# In situ topotactic transition to porous crystals boosts methane photooxidation

**DOI:** 10.1038/s41467-026-75390-1

**Published:** 2026-07-13

**Authors:** Yongchun Xiao, Xiuli Hu, Siu Wing Or, Kui Xie

**Affiliations:** 1 College of Materials Science and Engineering, Hunan University, Changsha, China; 2 Department of Electrical and Electronic Engineering, The Hong Kong Polytechnic University, Hung Hom, Kowloon, Hong Kong, China; 3 School of Mechanical Engineering, Shanghai Jiao Tong University, 800 Dongchuan Road, Minhang District, Shanghai, China; 4 Fujian Institute of Research on the Structure of Matter, Chinese Academy of Sciences, Fuzhou, Fujian, China; 5 Shandong Provincial Key Laboratory of Monocrystalline Silicon Semiconductor Materials and Technology, College of Chemistry and Chemical Engineering, Dezhou University, Dezhou, China; 6 Changxing Ocean Laboratory, Shanghai Jiao Tong University, 1896 Changtao Road, Chongming District, Shanghai, China; 7 Shanghai Changxing Ocean Laboratory, 1896 Changtao Road, Chongming District, Shanghai, China

**Keywords:** Solid-state chemistry, Energy

## Abstract

The rapid recombination of photogenerated electron-hole pairs represents a fundamental bottleneck in photocatalysis. Constructing porous single crystals is expected to resolve this issue by simultaneous optimization of light absorption, charge transport and mass diffusion. Here, we present a topotactic lattice contraction strategy for synthesizing porous single-crystalline zinc blende ZnO monoliths with exceptional crystallinity, phase purity and high porosity. In situ transmission electron microscopy reveals the atomic-scale formation process: preferential S/O migration along {222} channels enable epitaxial nucleation, while interfacial strain induces vacancy coalescence into interconnected nanopores. The low defect density in ZnO effectively suppresses photogenerated carrier recombination, exhibiting improved charge transport and prolonged carrier lifetime. Incorporating atomically dispersed Ru sites (0.6 wt%) further enhances charge separation efficiency, achieving 82% selectivity for methyl hydroperoxide in methane photooxidation while maintaining >85% activity over 40 hours. This work establishes a route to porous single crystals, advancing material design for prospective application in photocatalysis.

## Abstract (web summary)

Porous single crystals created via a topotactic transition uniquely integrate structural coherence with three-dimensional porosity, simultaneously optimizing light absorption, charge transport and mass diffusion for transformative photocatalysis.

## Introduction

Porous architectures with continuous solid-phase skeletons and interconnected fluid channels represent a paradigm shift in functional material design, achieving unprecedented surface-to-volume ratios and mass-transfer efficiencies that are revolutionizing catalysis, energy storage, and sensing technologies^1^. Their deployment in photocatalysis, however, is fundamentally constrained by the requirement for efficient long-range carrier transport with minimal dissipation^2,3^. In photocatalytic selective oxidation, for example, the activity relies critically on photogenerated holes (h^+^) and reactive oxygen species (e.g., ·O_2_- or ·OOH radicals), the latter arising from electron-driven O_2_ reduction^4^. The overall efficiency is therefore dictated by the separation, transport, and utilization of photogenerated charge carriers, and the central challenge is to design architectures that optimize these processes in concert. Conventional synthetic routes typically yield polycrystalline networks, in which grain boundaries and structural defects act as scattering and recombination centers, severely compromising carrier mobility and catalytic performance^5,6^.

Single crystals, with their extended periodic order and absence of grain boundaries, offer an ideal platform for efficient charge propagation^7^. Introducing porosity into such crystals could, in principle, combine coherent carrier transport with enhanced mass transfer and engineered surface reactivity—a combination pivotal for orchestrating complex catalytic pathways^8^. However, creating porous single crystals necessitates coevolution of a coherent single-crystal framework with a three-dimensional pore network; this inherently competing coupling imposes unprecedented challenges for materials synthesis. Epitaxial strategies with selective element removal to preserve lattice coherence during porogenesis are promising, yet fundamentally constrained by stringent lattice-matching requirements, thereby restricting material diversity^9,10^. Mechanistically, the formation of porous single crystals hinges on two coupled requirements: maintaining long-range lattice coherence to preserve single-crystalline properties, and precisely controlling lattice collapse through shrinkage or elemental extraction to generate porosity. Accordingly, surmounting the intrinsic limitations of epitaxy through dynamic reconstruction strategies that enable crystallographic inheritance beyond geometric matching constitutes a compelling frontier for materials synthesis.

**Fig. 1:**
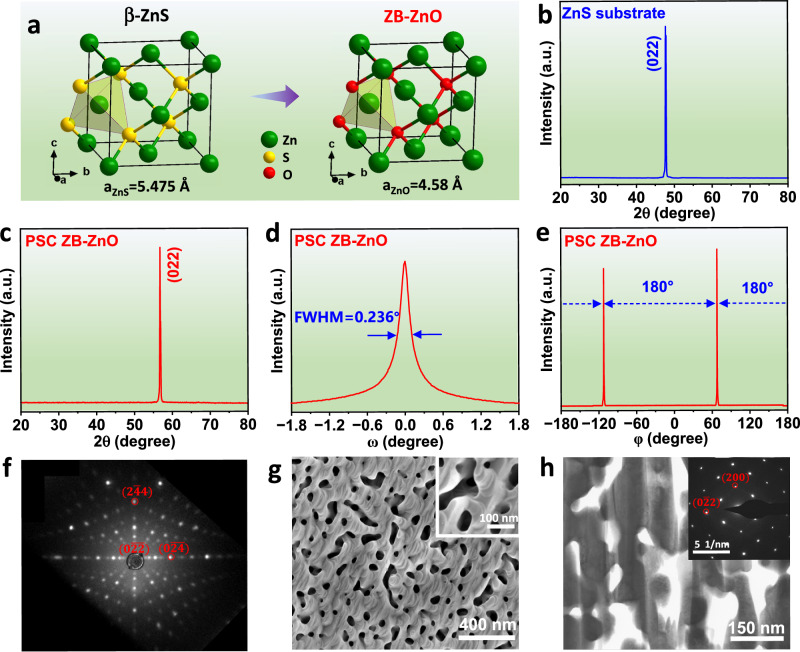
Structure analysis of PSC ZB-ZnO monolith derived from β-ZnS substrate. **a** Atomic-scale schematic illustrating the phase transition from cubic ZnS to ZB-ZnO, highlighting lattice reorganization during the sulfidation-to-oxidation process. **b** XRD patterns of cubic ZnS substrate. **c** XRD patterns of PSC BZ-ZnO. **d** High-resolution rocking curve of PSC ZB-ZnO(022) reflection with full-width at half-maximum (FWHM) of 0.236°, demonstrating exceptional crystallographic coherence. **e** XRD Φ-scan azimuthal dependence of (002) reflection displaying two-fold symmetry (180° periodicity). **f** Laue microdiffraction pattern revealing single-crystalline nature. **g** SEM image showing mesoporous architecture with crystal skeleton connectivity; inset is an enlarged view. **h** TEM image with inset FFT pattern confirming [022] zone axis orientation.

Kinetics-driven topotactic transitions represent a cornerstone of single-crystal transformations, enabling precise inheritance of lattice architecture through atomic-scale restructuring^11–13^. These registry-preserving phase transitions involve ordered atomic exchange and rearrangement at specific lattice sites, while maintaining invariant sublattice geometry and orientation^14,15^. Critically, deliberate lattice contraction can in situ generate porosity within the parent registry: Framework shrinkage accommodates excess volume through void formation rather than loss of crystallographic coherence^16^. Building upon this principle, we devise a topotactic contraction strategy that leverages crystallographic isomorphism between parent and product phases to program lattice shrinkage during isomorphic anion exchange, thereby driving vacancy clustering and establishing continuous 3D porosity while preserving the cation sublattice and monolithic single-crystal integrity. We demonstrate this strategy using wide-bandgap ZnO, whose suitable band structure enables the activation of molecular oxygen and water to generate essential reactive oxygen species essential for photocatalytic oxidation of hydrocarbon species, such as methane^17^. Our design involves a topotactic transformation from cubic β-ZnS (F-43m) to its oxide analog via a sulfur/oxygen anion exchange, targeting a controlled lattice contraction (5.41 Å → 4.58 Å), as illustrated in Fig. 1a.

Herein, we synthesize porous single-crystalline zincblende ZnO (PSC ZB-ZnO) monoliths and elucidate the dynamic in situ growth of porosity in single crystals via a topotactic lattice-contraction strategy. The resulting materials effectively suppress photogenerated carrier recombination, exhibiting prolonged carrier lifetime and enhanced charge transport. Further functionalization with atomically dispersed Ru sites significantly enhances carrier separation efficiency, steers O_2_ activation toward •OOH intermediates, achieving selective photocatalytic methane oxidation to methyl hydroperoxide (CH_3_OOH) with 82% selectivity and sustained operational stability over 40 h. This work establishes a topotactic route to porous single crystals, offering a versatile platform for the rational design of high-performance materials for advanced catalytic and energy applications.

## Results

### Synthesis and characterizations

To fabricate large-scale, grain-boundary-free porous ZnO single crystals, we employ a two-step synthesis strategy beginning with the growth of high-quality β-ZnS single crystals via chemical vapor transport (CVT)^18^. Precision orientation-controlled cutting and polishing yield a 10 × 10 × 0.5 mm β-ZnS substrate with single-crystalline perfection, as confirmed by XRD analysis along the <022> orientation (Fig. 1b), SAED patterns ([022] zone axis), and atomic-resolution TEM imaging (Supplementary Fig. 1). Subsequent solid-solid phase transformation under optimized conditions (O_2_/Ar atmospheres, 50–200 sccm, 450 °C–600 °C) converts β-ZnS into PSC ZB-ZnO while maintaining epitaxial registry, as evidenced by preserved (022) Bragg reflections (Fig. 1c) with macroscopic dimensional retention. Comprehensive structural characterizations revealed exceptional crystallinity (0.236° FWHM in rocking curve analysis, Fig. 1d) and two-fold symmetry of (001) planes (Fig. 1e), confirmed by Laue microdiffraction (Fig. 1f).

Microstructural analysis (Fig. 1g, h, Supplementary Fig. 2-5) demonstrates a homogeneous distribution of pores ( ~ 80 nm) within a coherent zinc-blende framework, resulting in a specific surface area of ~16 m²/g and a porosity of ~42%. 3D reconstruction analysis further confirms a continuous skeletal framework and an interconnected pore-throat network with an average-throat length of ~230 nm (Supplementary Fig. 6). Critically, SAED patterns along the [022] zone axis (inset Fig. 1h, and Supplementary Fig. 3) confirm cubic zincblende structure inheritance and crystallographic continuity. Rietveld refinement of XRD data (Supplementary Fig. 7 and Table S1) yields consistent cubic lattice parameters (a ≈ 4.584 Å, ±0.004 Å) across multiple synthesis batches. Remarkably, our multiscale analyses uncover a lattice inheritance mechanism where ZB-ZnO maintains atomic arrangement fidelity despite 15.3% lattice contraction (a = 5.40 Å → 4.58 Å). This topological reorganization preserves single-crystalline registry, far exceeding conventional epitaxial strain tolerances, presenting new opportunities to understand the dynamics of structural evolution in porous crystalline systems.

### Structure evolution

**Fig. 2:**
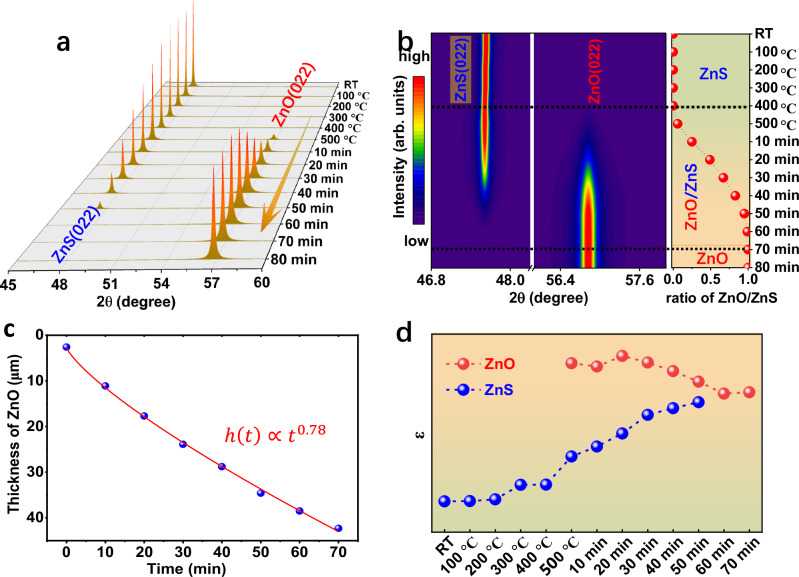
Temperature- and time-resolved phase evolution from β-ZnS to PSC ZB-ZnO. **a** Ex situ XRD patterns tracking the structural transformation from β-ZnS(022) to PSC ZB-ZnO(022) after programmed thermal and temporal treatment. **b** Integrated 2D-XRD mapping (left) and parameter-resolved phase evolution (right) for the β-ZnS(022) to PSC ZB-ZnO(022) evolution; the dashed line represents the critical point where phase transition begins or ends. **c** Time-Resolved Growth Kinetics of PSC ZB-ZnO on ZnS at 500 °C. **d** Strain evolution during growth of PSC ZB-ZnO grown on ZnS substrate.

To systematically investigate the structural evolution and kinetic mechanisms governing the β-ZnS to PSC ZB-ZnO transformation, we conduct ex situ X-ray diffraction (XRD) analyses during a precisely controlled heating process. As illustrated in Fig. 2a, the β-ZnS (022) reflection at 2θ = 46.8° exhibits remarkable stability from room temperature up to 400 °C, demonstrating the absence of phase transitions within this thermal range. A pivotal structural transition initiates at 500 °C, evidenced by the abrupt appearance of the PSC ZB-ZnO (200) diffraction peak at 2θ = 56.8°. The temporal evolution of the XRD patterns reveals a well-defined two-phase coexistence regime: the progressive intensification of the PSC ZB-ZnO peak occurs concomitantly with the gradual attenuation and eventual complete disappearance of the β-ZnS (022) signal over a 60-minute period - a characteristic signature of a diffusion-controlled solid-state reaction mechanism rather than an instantaneous phase transition. Complementary two-dimensional XRD mapping (Fig. 2b left) provides spatially resolved crystallographic insights, capturing the dynamic evolution of structural features with atomic-scale precision.

The structural characteristics of PSC ZB-ZnO reveal distinct differences from the parent β-ZnS phase, as evidenced by pronounced peak broadening in XRD patterns —a phenomenon we attribute to microstrain accumulation during lattice rearrangement. Quantitative analysis of the normalized intensity ratio (ZB-ZnO/β-ZnS) in Fig. 2b (right) reveals a nonlinear growth profile for PSC ZB-ZnO, which correlates with cross-sectional SEM observations showing time-dependent layer thickening. Kinetic analysis yields a growth exponent of h α t^0.77^ (R = 0.997), significantly deviating from Wagner’s parabolic law (h α t^0.5^) that characterizes conventional diffusion-limited oxide growth (Fig. 2c and Supplementary Fig. 8). We interpret this anomalous growth behavior as resulting from a complex interplay between increasing diffusional resistance and microstructural evolution. Specifically, the development of interconnected porosity establishes percolation pathways that temporarily counteract diffusion limitations, thereby enabling the observed superparabolic kinetics.

To investigate the porosity development in ZB-ZnO during thermal treatment under O_2_ (400 °C → 520 °C at 10 °C/min), we conduct in situ transmission electron microscopy (TEM) analysis using focused ion beam (FIB)-fabricated β-ZnS single-crystal lamellae mounted on MEMS heating chips (Supplementary Fig. 9). The sequential TEM images (Supplementary Fig. 10a–e) reveal distinct stages of porosity evolution: (1) At 400 °C, the surface remains smooth with minimal structural changes; (2) Initial pore nucleation emerges at 450 °C; (3) Progressive pore propagation occurs at 480 °C; (4) By 500 °C, pores coalesce into interconnected networks, culminating in a fully percolated 3D porous architecture at 520 °C through depthwise propagation. The accompanying schematic (Supplementary Fig. 10 f) illustrates the underlying mechanism: thermally activated anion exchange (S^2-^ → O^2-^) synchronizes sulfur release from the ZnS precursor with oxygen incorporation, creating vacancies whose aggregation, driven by significant lattice strain, generates porosity within the original crystal framework. This sequence of dynamics is corroborated at a larger scale by time-resolved FIB-SEM imaging of the transformation front, which visualizes the progressive growth of the porous framework. (Supplementary Fig. 11) Complementary strain analysis via the Williamson-Hall method provides further mechanistic insight. For single-crystal thin films, the absence of grain-boundary-induced size broadening simplifies the equation to *ε* = *β* / (4 tan*θ*) (Fig. 2d and Supplementary Fig. 12). The results show that β-ZnS exhibits progressive strain accumulation beyond 400 °C, indicating lattice distortion preceding the phase transition. In contrast, ZB-ZnO maintains relatively constant strain during initial growth (0–20 min at 500 °C) followed by strain relaxation. This delayed strain release correlates with the porous structure evolution, suggesting that void formation serves as a stress relief pathway during later growth stages.

### Atomic-scale topotactic transition

**Fig. 3:**
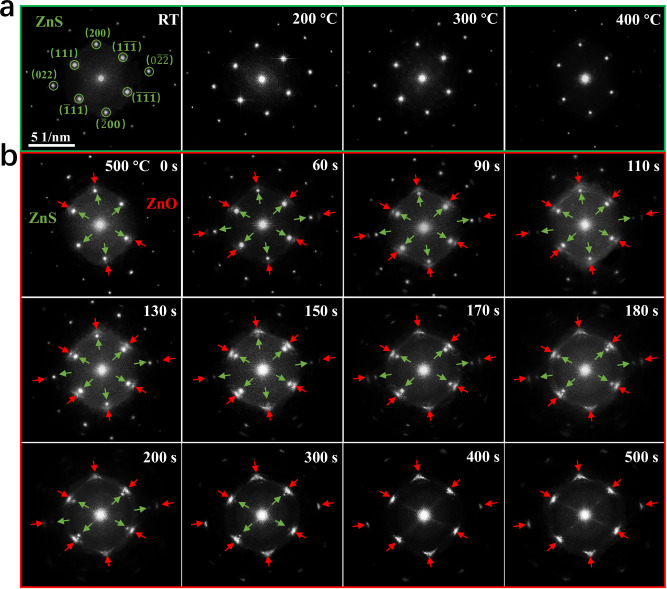
SAED pattern during topotactic transition from β-ZnS single crstal to PSC ZB-ZnO acquired via in situ TEM under 5×10⁻⁴ Torr O_2_. **a** SAED patterns exhibit no observable changes with temperature increase below 400 °C. **b** Sequential SAED evolution under isothermal oxidation (500 °C, 0 → 500 s). The green and red arrows represent the diffraction patterns of ZnS and ZnO, respectively.

To elucidate the atomic-scale mechanisms governing the β-ZnS→ZB-ZnO phase transition, we employ in situ TEM analysis of β-ZnS single-crystal lamellae subjected to controlled thermal oxidation (5×10^−4 ^Torr O_2_). Initial SAED characterization (Fig. 3a and Supplementary Fig. 13) confirms the remarkable thermal stability of β-ZnS below 400 °C, with sharp diffraction spots along the [$02\bar{2}$] zone axis persisting without peak broadening or additional reflections – consistent with XRD data that rules out phase transition or lattice distortion in this regime. The transformation dynamics become evident at 500 °C (Fig. 3b and Supplementary Fig. 14), where emerging ZB-ZnO diffraction spots (red arrows) progressively replace diminishing ZnS reflections (green arrows), providing direct evidence of the S^2-^ → O^2-^ anion exchange process. Notably, the ZB-ZnO spots exhibit anisotropic broadening, revealing interfacial microstrain accumulation during phase conversion. Crucially, the ZB-ZnO nuclei maintain precise crystallographic alignment with the parent matrix, as demonstrated by the co-oriented ZB-ZnO (200), (111), and (022) reflections. This epitaxial relationship confirms a topotactic transformation mechanism in which anion substitution occurs solely within the Zn2+ sublattice, preserving the original crystal orientation throughout the phase evolution.

Time-resolved SAED analysis at 500 °C reveals the isothermal oxidation kinetics through three distinct stages: (1) In the initial stage (0–90 s), rapid surface anion exchange drives epitaxial ZB‑ZnO nucleation on ZB‑ZnS with conserved orientation and weak emerging ZnO reflections while ZnS spots remain sharp; (2) During the intermediate stage (100–300 s), the ZnS diffraction spots weaken while the ZnO spots intensify and broaden with gradually slowing kinetics latter, indicating diffusion-limited growth and strain accumulation. (3) Beyond 400 s, the system reaches structural equilibrium with complete ZnS phase consumption and stabilized ZB-ZnO diffraction patterns. The absence of intermediate phases or amorphous domains throughout this process unequivocally demonstrates a direct substitutional transformation mechanism rather than dissolution-reprecipitation pathways. This kinetic evolution culminates in complete phase transformation at 400 s, with subsequent stabilization (500 s) marked by invariant ZB-ZnO diffraction intensities and positions—confirming the termination of the anion-exchange-driven reaction upon ZnS phase depletion.

**Fig. 4:**
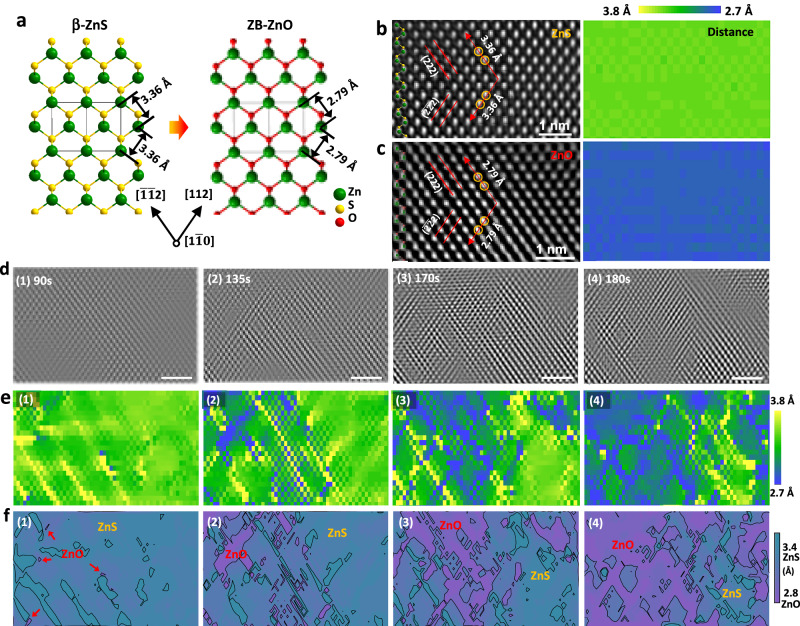
Topotactic transition process from β-ZnS to PSC ZB-ZnO captured by in situ HRTEM at 500 °C. **a** Atomic structure schematic diagram of the topological transition from cubic ZnS to ZB-ZnO. The structural transition preserves the cubic Zn sublattice while replacing S^2-^ with O^2-^. **b**,**c** Application results of the HRTEM image analyses for phase identification. (Left) HRTEM images of ZnS and ZB-ZnO with Zn-Zn interatomic spacing along the 〈112〉 direction measuring 3.36 Å and 2.79 Å, respectively. (Right) Interatomic spacing map of Zn-Zn_〈112〉_. **d** In situ HRTEM images of the atomic-scale transition process from cubic ZnS to ZB-ZnO at 500 °C. **e** Time-resolved spatiotemporal evolution of lattice dynamics showing the relative change in Zn-Zn_〈112〉_ interatomic spacing. **f** Contour plot of the Zn-Zn_〈112〉_ interatomic spacing, which was extracted from (**e**). Interatomic spacing of 2.8 Å corresponds to ZB-ZnO (pink reference).

In situ TEM directly captured the atomic-scale structural evolution during β-ZnS to ZB-ZnO transformation, revealing coherent lattice inheritance pathways. Figure 4a compares atomic configurations along the [$1\bar{1}0$] zone axis, showing the conserved face-centered cubic Zn²⁺ sublattice (cyan spheres) with S^2-^ → O^2-^ anion substitution (yellow to red spheres). This substitution induces a 15.3% contraction in Zn-Zn interatomic spacing along <112> (from 3.36 to 2.79 Å), consistent with lattice parameter reduction from 5.406 to 4.580 Å. The {222} crystallographic planes, with their expanded interplanar spacing, serve as preferential diffusion channels for anion exchange. A clean, electron-transparent β-ZnS specimen oriented along [$1\bar{1}0$] is prepared via low-energy FIB milling, enabling HRTEM characterization. Identically fabricated PSC ZB-ZnO thin film specimens allow comparative analysis. Figure 4b, c demonstrates HRTEM applications: (1) Phase discrimination through Zn-Zn <112> spacing measurements (3.36 Å for ZnS *vs*. 2.79 Å for ZnO); (2) Spatial mapping showing direct correspondence between interatomic spacing and phase distribution. The atomic-resolution imaging confirms the topotactic nature of the transformation, where anion substitution occurs within the preserved Zn^2+^ framework, maintaining crystallographic registry throughout the process.

The dynamic β-ZnS → ZB-ZnO topotactic transformation under controlled oxidation (500 °C, 5 × 10^−4 ^Torr O_2_) is systematically investigated through in situ HRTEM. Real-time observations (Fig. 4d and Supplementary Fig. 15) reveal the phase evolution mechanism, where structural similarities between parent β-ZnS and product ZB-ZnO phases necessitate advanced analytical techniques for phase discrimination. Spatiotemporal analysis of Zn-Zn <112> interatomic spacing evolution (Fig. 4e and Supplementary Fig. 16) provides direct evidence of the transformation process: At 90 s, elongated {111} interplanar expansion zones (yellow regions) emerges as rapid diffusion channels for S/O exchange, with peripheral lattice contraction domains ( ≈ 2.79 Å spacing, <1% area fraction) marking initial ZB-ZnO nucleation sites triggered by O^2-^ infiltration. By 135 s, propagating {222} interplanar channels (7% coverage) facilitates sulfur desorption and oxygen migration, enabling continuous ZB-ZnO nucleation/growth. At 170 s, these channels evolve into interconnected ZB-ZnO networks, while new expansion zones initiate secondary nucleation, achieving 80% phase conversion by 180 s.

Detailed Zn-Zn spacing contour analysis (Fig. 4f and Supplementary Fig. 17) identified three distinct regions: residual β-ZnS (blue, 3.4 Å spacing), ZB-ZnO domains (pink, 2.79 Å), and transitional zones with compressive strain driving vacancy coalescence into nanopores. Notably, regions exhibiting >3.4 Å spacing correlate with strain-engineered nanopores, demonstrating dynamic equilibrium between strain accumulation and pore-mediated energy dissipation at the atomic scale—providing mechanistic insight into the porosity formation process. The PSC ZB-ZnO formation mechanism involves a topotactic lattice contraction process governed by three key factors: (1) Selective S/O anion exchange preferentially along {222} diffusion highways; (2) Epitaxial ZB-ZnO nucleation at multiple sites within these channels, preserving parent-phase crystallographic orientation; (3) Interfacial strain-driven vacancy aggregation generating interconnected nanopores that ultimately form a strain-relieved architecture. Building on this mechanistic understanding, we further investigate the advantages of this unique PSC structure in optimizing charge transport and synergistically selective photocatalysis with Ru single atoms.

### Synthesis of Ru_1_/PSC ZB-ZnO for CH_4_ photooxidation

**Fig. 5:**
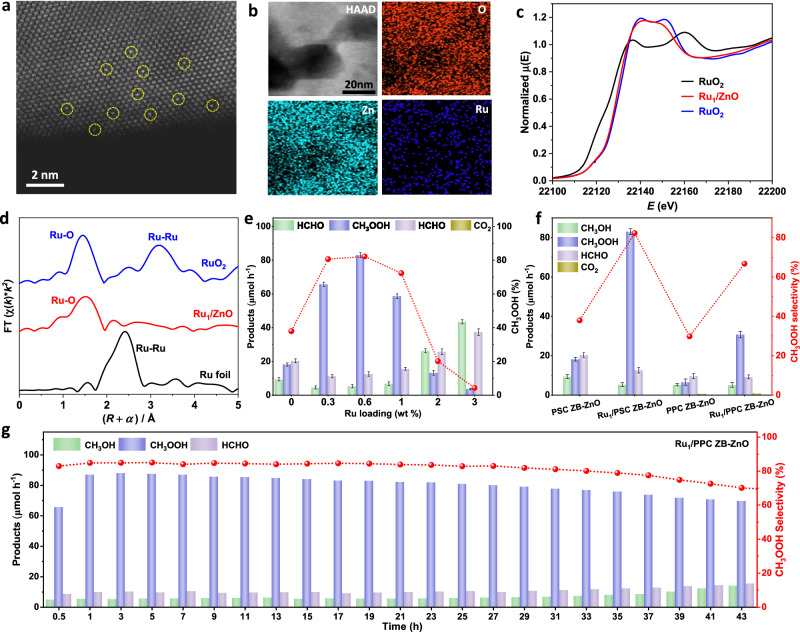
Structural characterization and photocatalytic methane oxidation performance of Ru_1_/ZB-ZnO catalysts. **a** AC-HAADF-STEM of Ru atoms in ZB-ZnO matrix, isolated Ru atoms are highlighted by dashed circles. **b** HAADF and elemental mapping images of PSC Ru_1_/ZB-ZnO. **c** Normalized Ru K-edge XANES spectra of Ru_1_/ZB-ZnO with RuO_2_ foil and Ru as references. **d** Fourier transform (FT) magnitudes of Ru K-edge EXAFS spectra in R space of Ru_1_/ZB-ZnO, RuO_2_, and Ru foil. **e** Dependence of photocatalytic methane oxidation performance on Ru loading content in Ru/ZB-ZnO catalysts. **f** Photocatalytic methane oxidation performance of PSC ZB-ZnO, Ru_1_/PSC ZB-ZnO, PPC ZB-ZnO, and Ru_1_/PPC ZB-ZnO. **g** Continuous stability tests on Ru_1_/ZB-ZnO. The reaction conditions in (**e–g**): CH_4_ (45 ml min^−1^) + air (5 mL min^−1^), and RH = 20%, 2 h, 100 mg catalysts, 69 °C, xenon lamp with AM 1.5 G filter, 1000 mW cm^-1^, 350–1000 nm. The error bars in (**e**,**f**) represent standard deviations obtained from three parallel experiments.

The 0.6 wt% Ru single atoms anchored on PSC ZB-ZnO (Ru_1_/PSC ZB-ZnO) were synthesized via atomic layer deposition (ALD). Aberration-corrected HAADF-STEM imaging (Fig. 5a) unambiguously reveals the atomic dispersion of Ru species, with isolated atoms distinctly marked by yellow dashed circles and a complete absence of clusters or aggregates. This uniform distribution is further substantiated by elemental mapping (Fig. 5b). XPS analysis resolves the chemical state of Ru, with deconvolution of the Ru 3d_5/2_ peak identifying Ru^4+^ species at 281.1 eV binding energy (Supplementary Fig. 18). XANES spectra position the Ru absorption edge between RuO_2_ and metallic Ru, closer to RuO_2_, while valence analysis quantifies the oxidation state at approximately +3.5 (Fig. 5c and Supplementary Fig. 19). EXAFS analysis exhibits no Ru-Ru scattering path at 2.2 Å, with wavelet transform (WT) contours resembling RuO_2_ (Fig. 5d and Supplementary Fig. 20). Quantitative fitting identifies a dominant Ru-O coordination peak at 2.006 Å with a coordination number of 4 (Supplementary Fig. 21 and Table S2), confirming Ru^4+^ single atoms stabilized through oxygen coordination in the PSC ZB-ZnO matrix.

Photocatalytic methane oxidation is conducted in a gas-solid flow reactor under visible light irradiation (AM 1.5 G filter, 1000 mW·cm^-2^, Supplementary Figs. 22–23). The 0.6 wt% Ru single-atom catalyst (Ru_1_/PSC ZB-ZnO) achieved a CH_3_OOH yield of 84.6 μmol·h^-1^—a 4.6-fold enhancement over pristine PSC ZB-ZnO—with high selectivity (82.5%) and a total C_1_ oxygenate yield of 100.93 μmol·h^-1^ (Fig. 5e and Supplementary Fig. 24–29). The high efficiency is further supported by an apparent quantum efficiency (AQE) of 15.3% at 365 nm (Supplementary Table S3). To decouple the role of single crystallinity, we synthesized porous polycrystalline controls (PPC ZB-ZnO and Ru_1_/PPC ZB-ZnO, Supplementary Fig.30 and Table S4). Both PSC ZB-ZnO and Ru_1_/PSC ZB-ZnO consistently outperform their polycrystalline counterparts (Fig. 5f), confirming that the porous single-crystalline framework critically enhances photocatalytic activity. The system also shows a near-linear dependence of CH_3_OOH on light intensity yield with stable selectivity (Supplementary Fig. 31), along with long-term operational stability (85% activity retention after 43 h, Fig. 5g). The stability of the Ru_1_ species was verified by post-reaction HAADF-STEM (Supplementary Fig. 32). The performance of Ru_1_/PSC ZB-ZnO demonstrates competitive selectivity, time yield and stability in CH_3_OOH synthesis compared to other catalysts (Supplementary Fig. 33 and Table S5–S7). Control experiments confirm that no products are detected in the absence of photocatalysts, light, or CH_4_ (Supplementary Fig. 34), indicating that all of the products originate from photocatalytic CH_4_ conversion.

To elucidate the origin of this performance difference, we analyzed the electronic structures of all catalysts. As shown by the UV-vis diffuse reflectance spectra (UV-vis DRS) and corresponding Kubelka-Munk plots (Supplementary Fig. 35), the band gaps are nearly identical between the porous single-crystal and polycrystalline ZnO supports (PSC ZB-ZnO: 3.16 eV; PPC ZB-ZnO: 3.17 eV). However, upon Ru modification, the bandgap slightly narrows (Ru_1_/PSC ZB-ZnO: 3.13 eV; Ru_1_/PPC ZB-ZnO: 3.14 eV), indicating that Ru incorporation induces a marginal red-shift of the absorption edge, which is beneficial for enhancing visible-light absorption. Through UPS, VB-XPS, and Mott-Schottky analysis (Supplementary Figs. 36–38), we construct the band structure diagram (Supplementary Fig. 39), which shows that single-crystalline and polycrystalline ZnO have similar band alignments, while Ru modification causes a minor negative shift of both the valence band (VB) and conduction band (CB). This optimized band alignment in Ru-modified ZnO facilitates more efficient charge separation and stronger redox driving forces. Based on the positions of VB/CB and the thermodynamic potentials of oxygen activation reactions (H_2_O/•OH: 2.380 V; O_2_/•OOH: 0.05 V), all four catalysts can generate •OH and •OOH active species via H_2_O oxidation and O_2_ reduction, respectively.

### Charge transport characteristics

**Fig. 6:**
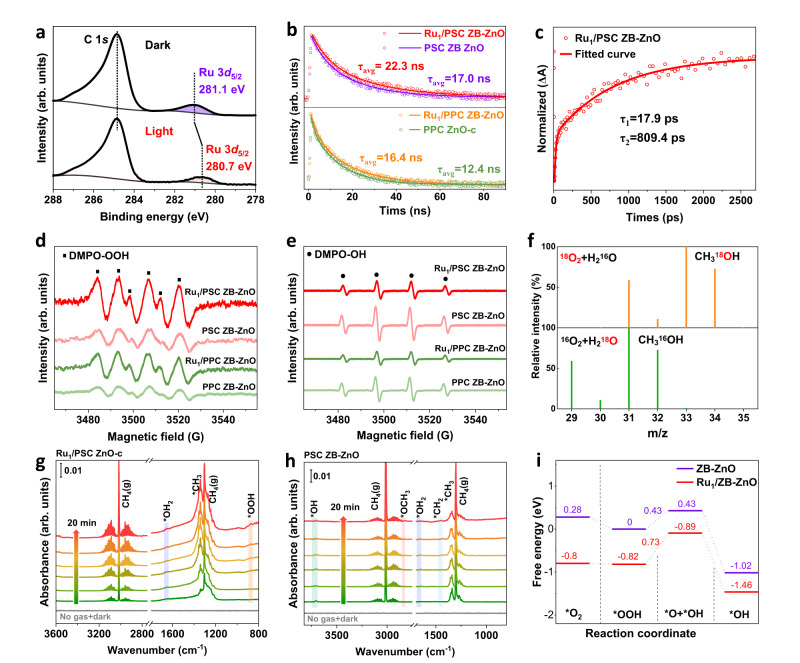
Mechanism of the selective CH_4_ photo-oxidation over the Ru_1_/PSC ZB-ZnO catalyst. **a** In situ Ru 3*d*_5/2_ XPS spectra of Ru/PSC ZB-ZnO in the dark and under light irradiation. **b** Time-resolved PL decay of Ru_1_/PSC ZB-ZnO and PSC ZB-ZnO; circles and lines represent measured and fitted data, respectively. **c** The normalized TAS decay kinetics of Ru_1_/PSC ZB-ZnO film probed at 412 nm excited by pump 360 nm. **d**,**e** EPR spectra of Ru_1_/PSC ZB-ZnO, PSC ZB-ZnO, Ru_1_/PPC ZB-ZnO and PPC ZB-ZnO in air-saturated water under light irradiation for 5 min. **f** GC-MS spectra of CH_3_OH reduced from CH_3_OOH over Ru_1_/PSC ZB-ZnO with ^18^O_2_ + H_2_^16^O or ^16^O_2_ + H_2_^18^O in photocatalytic CH_4_ oxidation. **g**,**h** In situ DRIFTS spectra for CH_4_ photooxidation over Ru_1_/PSC ZB-ZnO and PSC ZB-ZnO during light irradiation. **i** Calculated reaction energy of O_2_ adsorption and activation over Ru_1_/ZB-ZnO and ZB-ZnO catalysts.

To investigate charge transfer mechanisms, in situ X-ray photoelectron spectroscopy (XPS) was performed. Figure 6a reveals a distinct low-energy shift in the Ru 3*d*_5/2_ binding energy from 281.1 eV (dark) to 280.7 eV (light), confirming Ru atoms act as electron acceptors. Simultaneously, the O 1 *s* peak shifts oppositely under illumination (Supplementary Fig. 40), indicating hole accumulation on lattice oxygen. These results directly map the charge-separation pathway: electrons migrate to Ru acceptors for O_2_ reduction, while holes accumulate on framework oxygen for oxidation.

To probe charge carrier dynamics, photoluminescence (PL) and electrochemical analyses were conducted. The steady-state PL spectra (Supplementary Fig. S41) show that the PL emission intensity follows the trend: PPC ZB-ZnO > PSC ZB-ZnO > Ru_1_/PPC ZB-ZnO > Ru_1_/PSC ZB-ZnO. This indicates that the porous single-crystalline framework itself suppresses recombination relative to its polycrystalline counterpart by eliminating grain boundaries, while Ru single atoms as an effective electron sink further quench the PL signal. This synergistic suppression of recombination is quantitatively validated by Time-resolved PL decay measurements (Fig. 6b). The average carrier lifetime (τ_avg_) of Ru_1_/PSC ZB-ZnO is 22.3 ns, significantly longer than that of Ru_1_/PPC ZB-ZnO (16.4 ns). Even without Ru, pristine PSC ZB-ZnO exhibits a longer lifetime (τ_avg_ = 17.0 ns) than PPC ZB-ZnO (τ_avg_ = 12.4 ns), highlighting the intrinsic advantage of the single-crystalline framework in prolonging charge carrier lifetime. Ru modification further extends the lifetime, indicating enhanced survival of photogenerated carriers for surface reactions^19,20^. Electrochemical impedance spectroscopy (EIS, Supplementary Fig. 42) further reveals lower charge transfer resistance for single-crystalline ZnO compared to polycrystalline ZnO, with Ru-modified catalysts showing the lowest resistance. These results underscore a synergistic interplay: the single-crystalline structure provides a conductive, low-defect bulk transport pathway, while atomically dispersed Ru sites accelerate interfacial charge-transfer kinetics.

Femtosecond transient absorption spectroscopy (fs-TAS) reveals that the porous single-crystalline ZB-ZnO framework exhibits rapid charge transport, while Ru single atoms further enhance photogenerated carrier density and suppress recombination (Fig. 6c, Supplementary Figs. 43, 44 and Table S8)^21^. Specifically, Ru doping accelerates electron transfer to trap states by ~30% and extends the radiative recombination lifetime by ∼2.9 fold, confirming that atomically dispersed Ru sites simultaneously promote charge separation and prolong carrier availability for surface reactions^22,23^. These results underscore the synergistic enhancement in charge-carrier management achieved by integrating the single-crystalline architecture with Ru single-atom modification.

### Mechanism of photocatalytic CH_4_ mechanism

To probe the adsorption behaviors, temperature-programmed desorption (TPD) analyses were conducted (Supplementary Fig. S45). O_2_-TPD analysis reveals that Ru single atoms in Ru_1_/PSC ZnO significantly enhance O_2_ adsorption, indicating their role as preferential sites for O_2_ activation. CH_4_-TPD shows a prominent adsorption peak at 470 °C on ZnO for both samples, indicating that CH_4_ primarily adsorbs on ZnO surface, with Ru introduction only a weak secondary peak at 288 °C. H_2_O-TPD profiles are nearly identical, indicating that Ru does not alter water adsorption sites (such as surface-terminated Zn/O, surface hydroxyl species).

In situ EPR with DMPO reveals that Ru single atoms shift radical selectivity from •OH (dominant on pristine ZnO) to •OOH (prominent on Ru-loaded samples), a change attributed to Ru serving as preferential O_2_ adsorption sites (Fig. 6d, e)^23,24^. The porous single-crystal substrates (Ru_1_/PSC ZB ZnO and PSC ZB-ZnO) generate more intense •OOH (for Ru_1_) and •OH (for ZnO) signals than their porous polycrystalline counterparts (Ru_1_/PPC ZB ZnO and PPC ZB-ZnO), owing to their lower bulk defect density, which facilitates more efficient charge carrier transport to the surface for O_2_ activation. Thus, Ru single atoms together with the single-crystalline ZnO framework synergistically promote the •OOH pathway, steering selective oxidation toward CH_3_OOH while mitigating over-oxidation.

To further confirm the potential role of charge carriers and •OH species responsible for CH_4_ oxidation, quenching experiments were conducted using Na_2_C_2_O_4_, isopropanol and AgNO_3_ as scavengers of h^+^, •OH and e^-^respectively. As illustrated in Supplementary Fig. 46, the introduction of h⁺ and e^-^ scavenger nearly completely suppresses the reaction. When isopropanol is added as •OH quencher, the yield of products decreases to ca. 45% of the original level. These results indicate that •OH contributes to methane activation, while holes play the dominant role in the initial C–H bond cleavage. Moreover, electrons are essential for driving the reduction step that generates the key •OOH intermediate.

To definitively trace the origin of oxygen in CH_3_OOH, isotope labeling experiments were performed using either ^18^O_2_ or H_2_^18^O as the oxygen tracer, and the resulting oxygen-containing products were subsequently analyzed by gas chromatography-mass spectrometry (GC-MS)^25^. To prevent CH_3_OOH thermal decomposition during GC-MS analysis under aerobic conditions, we reduced CH_3_OOH to CH_3_OH with NaBH_4_ before analysis. As shown in Fig. 6f, the use of ^18^O_2_ and H_2_^16^O resulted in strong signal peaks for CH_3_^18^OH, whereas no CH_3_^18^OH was detected in reactions carried out with ^16^O_2_ and H_2_^18^O. This provides strong evidence that O_2_ is the source of oxygen for CH_3_OOH. Moreover, control experiments conducted in the absence of O_2_ yielded no product formation, which conclusively rules out any contribution from lattice oxygen (Supplementary Fig. 34).

To elucidate the intermediate species involved in the photocatalytic CH_4_ oxidation process, in situ diffuse reflectance infrared Fourier transform spectroscopy (DRIFTS) was performed under simulated reaction conditions (Supplementary Fig. 47). For the Ru_1_/PSC ZB-ZnO catalyst, distinct absorption bands were observed (Fig. 6g): (1) the *ν*(C − H) vibrational modes of gaseous CH_4_ at 1306 cm^-1^, (2) the δ(C − H) bending mode of adsorbed *CH_3_ species at 1341 cm^-1^ (intensity increasing with reaction time), and (3) a newly emerging band at 870 cm^-1^ attributed to the •OOH intermediate, likely originating from O_2_ reduction. The temporal enhancement of both •CH_3_ and •OOH signals suggests the progressive formation of CH_3_OOH through coupling between •CH_3_ (generated via C − H bond activation of CH_4_) and •OOH. In contrast, bare PSC ZB-ZnO exhibited markedly different behavior (Fig. 6h): (1) a prominent CH_3_ stretching vibration band at 2824 cm^-1^, which is assigned to surface-bound •OCH_3_ species; and (2) a distinct OH stretching vibration band at 3703 cm^-1^, indicative of adsorbed •OH radicals under illumination^26^. Additionally, the detection of •CH_2_ intermediates at 1447 cm^-1^ implies a propensity for deeper oxidation pathways in the absence of Ru cocatalysts^27^. Notably, the absence of the 3703 cm^-1^ (•OH) band on Ru_1_/ZB-ZnO confirms the suppression of •OH radical generation, which aligns with its enhanced selectivity toward partial oxidation products. Under CH_4_ atmosphere, both samples exhibit discernible *CH_3_ and *OCH_3_ signals upon irradiation (Supplementary Fig. 48), further confirming that the holes (O^−^) generated from photoexcitation of ZnO can effectively activate CH_4_ to form *CH_3_ on the surface. These in situ DRIFTS results collectively demonstrate that Ru single atoms effectively modulate surface reaction pathways by inhibiting •OH formation and stabilizing key intermediates (•CH_3_ and •OOH) for selective CH_4_ conversion to CH_3_OOH.

DFT calculations reveal the potential role of isolated Ru sites in modulating O_2_ photoreduction pathways (Fig. 6i). Using the dominant (222) surface of ZB-ZnO as the model (Supplementary Figs. 49–50, Supplementary Data 1), comparative analysis reveals that Ru_1_/ZB-ZnO enhances O_2_ chemisorption ( − 0.08 eV vs. 0.28 eV for pristine ZB-ZnO) and thermodynamically favors •OOH formation (ΔG = −0.02 eV vs. −0.28 eV). Additionally, Ru might raise the energy barrier for •OOH dissociation (0.73 eV vs. 0.43 eV), which could kinetically suppress of O-O bond cleavage and stabilizing •OOH intermediates. These theoretical results demonstrate that isolated Ru sites kinetically enhance •OOH selectivity and suppress overoxidation. It should be noted that these computational models involve necessary simplifications and cannot fully replicate the complexity of the material under realistic catalytic conditions.

According to the all above results, we propose a reaction mechanism for photocatalytic CH_4_ oxidation over Ru_1_/ZnO under O_2_, as depicted in Supplementary Fig. 51. Upon light irradiation, the electrons are excited from VB to CB of ZnO, and then transfer to the decorated Ru single atoms, wherein O_2_ is selectively reduced to •OOH Concurrently, the holes remaining in VB of ZnO activate adsorbed CH_4_ to form •CH_3_, which can then couple with •OOH to yield CH_3_OOH as the primary product. Additionally, •OH radicals generated by H2O oxidation may further promote CH4 activation, thereby contributing to the overall reaction pathway.

## Discussion

In summary, we have developed a topotactic lattice contraction strategy to synthesize centimeter-scale porous single crystals while preserving long-range crystallinity. Using β-ZnS to PSC ZB-ZnO transformation as a model, multiscale characterization reveals that S/O anion exchange along β-ZnS {222} diffusion pathways triggers ZnO nucleation and epitaxial expansion within the intact cation sublattice. The associated lattice contraction generates strain, which is relieved by vacancy aggregation into an interconnected nanopore network, yielding a coherent monolith with permeating porosity. The resulting PSC ZB-ZnO architecture effectively suppresses photogenerated carrier recombination, exhibiting prolonged carrier lifetime and enhanced charge transport. Incorporation of atomically dispersed Ru active sites (0.6 wt.%) within the ZB-ZnO framework further enhances charge separation and directs oxygen reduction selectively toward *OOH intermediates, thereby achieving 82% selectivity for methyl hydroperoxide (CH_3_OOH) with operational stability exceeding 40 h in methane photo-oxidation. Our study establishes a paradigm for PSC materials design through topotactic contraction, providing an advanced material platform for solar-driven high-efficiency synthesis technologies.

## Methods

### Crystal growth

High-quality ZnS single crystals grown via iodine vapor transport. ZnS powder (99.99%) was vacuum-annealed (1000 °C, 48 h) to form phase-pure ZnS. Crystal growth occurred in sealed quartz ampoules (850 °C source, 5 mg/cm^3^ I_2_, 99.999%) where optimized ampoule geometry (5 cm length, controlled diameter/taper) and thermal gradients enabled defect-free, large-area crystals at >1 mm/h rates. Then ZnS crystals were oriented along the (022) crystallographic plane, cut into 10 × 10 mm × (0.1–0.5 mm) slabs, and polished to sub-nanometer roughness for PSC ZnO synthesis. The ZnS substrates were loaded into a high-vacuum chamber ( ± 0.01 mbar precision) equipped with digitally calibrated mass flow controllers (MFCs, ±0.5% accuracy) for gas-phase anion exchange. Solid-solid conversion to PSC ZnO was achieved under controlled O_2_/Ar atmospheres (50–200 sccm, 5 N purity) at 450 °C–600 °C for 30–100 h, with dynamic pressure regulation (1–1000 mbar) to balance lattice reconstruction kinetics and defect annihilation. PPC ZnO was obtained under similar conditions, except using a gas flow rate of 2–5 LSM and a pressure of 2–5 bar.

### Synthesis of Ru_1_/PSC ZB-ZnO

Ru_1_/PSC ZB-ZnO was synthesized via atomic layer deposition (ALD) using bis(cyclopentadienyl) ruthenium(II) (Ru(Cp)_2_, 99.9%, HWRK Chem) as the Ru precursor and O_2_ (99.99%)plasma as the co-reactant. The ALD process was conducted in a custom ultrahigh-vacuum chamber (base pressure <1×10^-8 ^Torr) at a deposition temperature of 250 °C with the following cyclic sequence: (1) Ru precursor pulse, 0.8 s at 150 °C (vaporizer temperature) with Ar carrier gas. (2) Purge, 15 s Ar flow to remove physiosorbed precursors. (3) O_2_ plasma exposure, 10 s RF plasma (300 W, 13.56 MHz) in 20% O_2_/Ar mixture. (4) Final purge, 20 s Ar flow. Ru loadings of 0.3% and 0.6% were achieved by tuning the Ru precursor pulse duration (0.1 s vs. 0.8 s) within a single atomic layer deposition (ALD) cycle, while higher loadings (1-3%) were controlled by varying the ALD cycle count (10–30 cycles) at a fixed 0.1 s pulse. Post-synthesis annealing at 400 °C under Ar for 2 h enhanced Ru-O-Zn interfacial coordination.

### Characterizations

X-ray diffraction (XRD, Cu-Kα, Mniflex 600) is used to determine the phase structure and facet orientation. XRD Phi scan, omega scan (XRD-D8 Discover) and Laue diffraction patterns (Laue_V_674_AC_GigE) are used to characterize the single-crystalline features. Morphological evolution and microstructural features were examined by Field-emission scanning electron microscope (FE-SEM) (SU8010), complemented by atomic-resolution lattice imaging using aberration-corrected transmission electron microscopy (Cs-TEM, FEI Titan3 G2 60-300). Cross-sectional TEM specimens were prepared via focused ion beam milling (FIB, Zeiss Auriga Helios 650). Ruthenium (Ru) contents were measured through Inductively Coupled Plasma Mass Spectrometry (ICP-MS) (Agilent 8900). Brunauer-Emmett-Teller (BET) surface areas were collected by ASAP 2460 Micromeritics instrument. The photoluminescence (PL) spectrum was measured at room temperature using a spectrophotometer (Edinburgh Instruments, FLS920). UV-vis diffuse reflectance spectra (DRS) were recorded on a Hitachi U-3010 spectrometer (200–800 nm range) using α-Al_2_O_3_ as the reflectance standard, with optical band gaps derived from Kubelka-Munk transformations. Electrochemical impedance spectroscopy (EIS) and Mott-Schottky measurements were performed using a CHI660E electrochemical workstation. A conventional three-electrode configuration was employed, with the as-prepared sample serving as the working electrode, a platinum gauze as the counter electrode, and an Ag/AgCl (saturated KCl) electrode as the reference electrode. A 0.1 M Na_2_SO_4_ solution was used as the electrolyte. Mott–Schottky plots were recorded at frequencies of 500, 800, and 1100 Hz. In situ irradiation XPS was conducted on a ThermoFisher Scientific EscaLab QXi spectrometer under dark or light conditions. X-ray absorption spectroscopy (XAS) experiments at the Cu K-edge were conducted at the BL11B beamline station in the Shanghai Synchrotron Radiation Facility (SSRF).

### In situ TEM experiments

Real-time structural evolution was captured using an environmental TEM platform (Thermo Themis G3) equipped with a precision heating stage (DENS Wildfire). Specimens were subjected to thermal protocols (25 °C–600 °C, O_2_/Ar atmosphere) with controlled ramp rates (0.1 °C–0.5 °C·s^-1^). To minimize beam-induced artifacts, electron flux density was maintained below 50 pA·cm^-2^ through automated beam blanking during thermal transients. The in-situ TEM test for each slice is an independent heating process, from the initial temperature to the desired temperature.

### Photocatalytic activity tests

Methane oxidation reactions were conducted in a customized gas-phase continuous-flow photoreactor. The light source was a 300 W xenon lamp (CEL-HXUV300-S) equipped with AM 1.5 G filter, 1000 mW·cm^-2^). The light intensity was calculated using the five-point method (Supplementary Fig. S23). The catalytic system employed 100 mg photocatalyst placed in reaction cell, with precisely controlled gas streams (50 mL·min^-1^ total flow rate) containing CH_4_ (99.999%) and synthetic air (O_2_/N_2_ = 25 vol%). Humidity modulation (RH 25%) was achieved through calibrated bubbler systems maintained at 25.0 °C ± 0.1 °C. Gaseous products were detected via gas chromatography (GC, FULIGC9790II) equipped with a methanizer and a flame ionization detector. No gaseous products other than CO₂ (below the detection limit) were detected (Supplementary Fig. S28). The gaseous HCHO was cryogenically trapped in 50 mL deionized water (5 min at 0 °C) and subsequently analyzed via UV-vis spectrophotometry (Hitachi U4100). The chromogenic reaction was initiated by mixing 5 mL aliquot with 1 mL acetylacetone solution (0.25% v/v), followed by thermal treatment in boiling water (3 min). Absorbance at 413 nm was measured against HCHO calibration standards (Supplementary Fig. S26). Methyl hydroperoxide (CH_3_OOH) and methanol (CH_3_OH) concentrations were determined by ^1^H NMR spectroscopy (Bruker AVANCE III HD 400 MHz) using water suppression techniques. Samples were prepared by mixing 0.5 mL absorption solution with 0.1 mL D_2_O (99.9%) and 0.05 μL DMSO (internal standard, 99.9%). Calibration curves (concentration vs. DMSO area ratio) were prepared, and products were quantified by referencing ^1^H NMR signals to these curves (Supplementary Fig. 25).

The AQE of Ru1/ZnO photocatalyst was measured at different wavenumbers using an Xe lamp equipped with a band-pass filter as the light source. AQE was determined using the following equation:

1 $${AQE}=\frac{{N}_{{\mbox{e}}}}{N({\mbox{photons}})}\times 100\%$$

*N*_e_ represents the number of reacted electrons, *N*_*e*_ = *N*(CH_3_OOH) + 3×*N*(CH_3_OH) + 5×*N*(HCHO). Where *N*(CH_3_OOH), *N*(CH_3_OH), *N*(HCHO) denote the molar quantities of synthesized methyl hydroperoxide, methanol, and formaldehyde, respectively. *N*(photons) represents the number of reacted electrons and the number of incident photons during methane conversion.

### Fs-TAS

The fs-TAS measurements were performed using a Helios Fire spectrometer (Ultrafast Systems LLC). The pump beam (360 nm) was obtained by the transmitted pulse processed by a TOPAS optical parametric amplifier. A broadband white-light continuum probe spanning 390–500 nm was employed. Differential absorption (ΔA) signals were derived from alternating probe pulses (pump-blocked/unblocked states), with temporal resolution controlled via a motorized delay stage. Decay kinetics were modeled using biexponential fitting:

2 $$\Delta A={A}_{1}{e}^{-t/{\tau }_{1}}+{A}_{2}{e}^{-t/{\tau }_{2}}$$

where *τ*_1,2_ represent characteristic decay lifetimes and *A*_1,2_ their relative amplitudes.

### TPD test

The CH_4_-TPD experiments were performed on a AutoChem 2930. Prior to CH_4_ adsorption, the catalyst sample (0.1 g) was pretreated under a He flow at 200 °C for 60 min. After cooling to 50 °C, the sample was exposed to a CH_4_ flow for 60 min to achieve saturation adsorption, followed by purging with He to eliminate physically adsorbed species. Subsequently, the temperature was ramped from 50 °C to target temperature at a heating rate of 5 °C·min^-1^, and the desorbed signals were recorded. The sample was then cooled to room temperature at the same rate. The O_2_-TPD and H_2_O-TPD experiments were carried out with a similar process.

### EPR measurement

Electron paramagnetic resonance (EPR) spectroscopy was performed on a Bruker E580 X-band spectrometer (9.85 GHz microwave frequency, 1 G modulation amplitude) at 298 K using 5,5-dimethyl-1-pyrroline N-oxide (DMPO, 99%, Macklin) as the spin-trapping agent. For superoxide radical (•O_2_^-^) detection: 5 mg photocatalyst was dispersed in 4 mL methanol containing 50 mM DMPO, followed by 5 min photoirradiation under aerobic conditions with continuous stirring. Subsequently, 0.5 mL aliquots were rapidly transferred to quartz EPR tubes for measurement. Hydroxyl radical (•OH) identification followed an analogous protocol, employing an aqueous DMPO solution (10 mM) as the trapping matrix.

### Active species trapping experiment

To explore the potential role of active species responsible for CH_4_ oxidation, quenching experiments were conducted using Na_2_C_2_O_4_ (99.96%, aladdin), isopropanol (99.8%, macklin) and AgNO_3_ (0.1 g/ml, Nanjing reagent) as scavengers for photogenerated holes (h^+^), hydroxyl radicals (•OH) and electrons (e^-^), respectively. Typically, 20 mg of photocatalyst was suspended in 50 mL deionized water supplemented with 7.5 μmol of each scavenger^28^. After sealing the vessel and purging with the gas mixture (0.1 MPa air, 1.9 MPa CH_4_, the gases purity were the same as above), the reaction system was irradiated for 1 h.

### Isotope labeling experiments

To trace the origin of oxygen in CH_3_OOH, we conducted isotope experiments using ^18^O-labeled oxygen (^18^O_2_) or ^18^O-labeled water (H_2_^18^O, 99%) in the reaction. Typically, 100 mg of photocatalyst was employed in the reaction cell. The reactor was then degassed for 30 min to completely remove air, with precisely controlled gas streams (50 mL·min^-1^ total flow rate) containing CH_4_ (99.999%) and synthetic air (25 vol%^18^O_2_, or ^16^O_2_). H_2_^16^O or H_2_^18^O was introduced into the reaction system via methane bubbling. Then we reduced CH_3_OOH to CH_3_OH with NaBH_4_ (99.99%) before analysis. The products were measured by GC-MS (Agilent 7890B-5977B).

### In situ DRIFTS experiments

In situ FTIR spectroscopic analysis was conducted using a thermo scientific spectrometer. The sample was pretreated by Ar purging for 30 min to acquire background reference spectra. A humidified CH_4_/air mixture (saturated vapor introduced via deionized water bubbling) was subsequently delivered into the reaction chamber. Time-resolved spectra were collected under continuous illumination (Supplementary Fig. S47).

### Computational method

The Vienna Ab-initio Simulation Package (VASP)^29^, based on density functional theory, was employed for structural relaxation. The Perdew-Burke-Ernzerhof (PBE) functional within the generalized gradient approximation (GGA)^30^ was selected to describe exchange-correlation interactions. Projector augmented wave (PAW) potentials^31^ were used to represent ionic cores, and a plane-wave basis set with a kinetic energy cutoff of 450 eV was applied. A convergence threshold of 10^−5^ eV was set for electronic self-consistency. Geometry optimization was regarded as converged when all atomic forces were below 0.04 eV/Å. A 15 Å vacuum layer was placed along the z-axis to eliminate spurious interlayer interactions, while van der Waals interactions were corrected using Grimme’s DFT-D3 method^32^. A 2 × 2 × 1 Monkhorst-Pack grid was adopted for Brillouin zone sampling during structural relaxation. Spin polarization was introduced to characterize the magnetic properties of slab models.

Nørskov’s computational hydrogen electrode (CHE) approach^33,34^ was utilized to calculate the Gibbs free energy change (ΔG) of gas-phase species and surface-adsorbed intermediates. The corresponding formula is expressed as:

3 $$\Delta G=\Delta E+\Delta {E}_{{ZEP}}-T\Delta S$$

Here, ΔE refers to the total energy difference obtained from DFT calculations, ΔE_ZPE_ is the zero-point energy change, and ΔS represents the entropy correction. All calculations were carried out at a standard temperature T of 298.15 K.

## Supplementary information

Supporting Information

Description of Additional Supplementary File

Supplementary Data 1

Transparent Peer Review file

## Source data

Source data

## Data Availability

All reported data in this study are included in the paper and supplementary information. Atomic coordinates of the optimized structures are provided in Supplementary Data 1. Source data are provided with this paper.
