# Supplementary material for: In situ topotactic transition to porous crystals boosts methane photooxidation
Source: Nat Commun. 2026 Jul 13;17:8612. doi: 10.1038/s41467-026-75390-1 (PMC13486515; doi:10.1038/s41467-026-75390-1)
Supplement: Supplementary file 1 [file 41467_2026_75390_MOESM1_ESM.pdf]

Supplementary Information

In situ Topotactic Transition to Porous Crystals Boosts Methane Photooxidation

Yongchun Xiao [a, b, c, e] *, Xiuli Hu [d], Siu Wing Or [b], and Kui Xie [c, e, f, g] *

[a] College of Materials Science and Engineering, Hunan University, Changsha, Hunan, 410082, China

[b] Department of Electrical and Electronic Engineering, The Hong Kong Polytechnic University, Hung Hom, Kowloon, Hong Kong, China

[c] School of Mechanical Engineering, Shanghai Jiao Tong University, 800 Dongchuan Road, Minhang District, Shanghai 200240, China

[d] Shandong Provincial Key Laboratory of Monocrystalline Silicon Semiconductor Materials and Technology, College of Chemistry and Chemical Engineering, Dezhou University, Dezhou 253023, China

[e] Fujian Institute of Research on the Structure of Matter, Chinese Academy of Sciences, Fuzhou 350002, Fujian, China

[f] Changxing Ocean Laboratory, Shanghai Jiao Tong University, 1896 Changtao Road, Chongming District, Shanghai 200240, China

[g] Shanghai Changxing Ocean Laboratory, 1896 Changtao Road, Chongming District, Shanghai 200240, China

*Corresponding Author: ycxiao@hnu.edu.cn; xiekui@sjtu.edu.cn

Table of Contents

This PDF file includes:

Supplementary Figures 1–51

Supplementary Tables S1–S8

Supplementary References 1–25

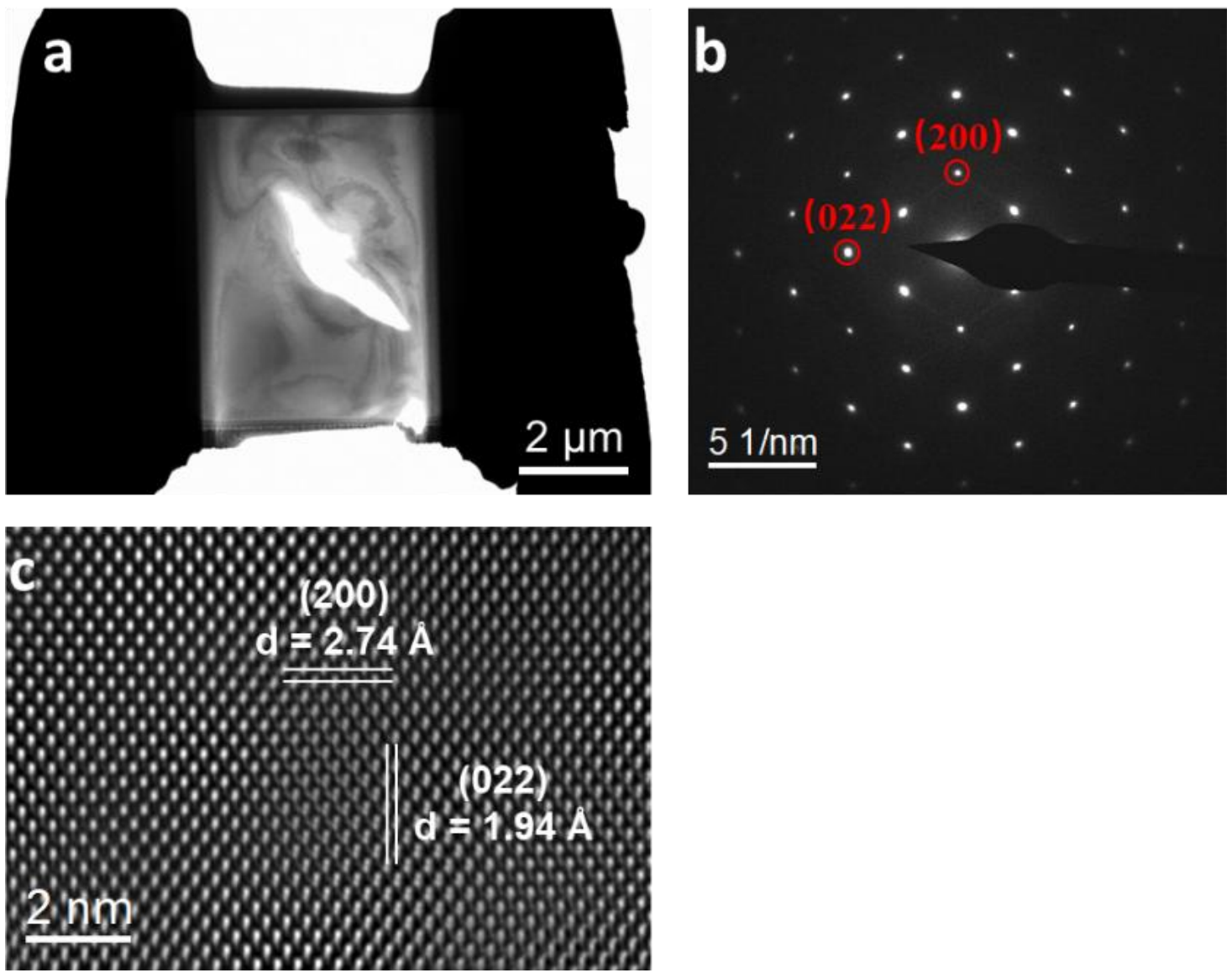

Figure S1. Structural characterization of β-ZnS. (a) Focused ion beam (FIB)-fabricated TEM slice. **(b)** Selected-area electron diffraction (SAED) pattern indexed to $[02\bar{2}]$ zone axis, with (200) and (022) reflections labeled. **(c)** Aberration- corrected high-resolution TEM image revealing atomic lattice fringes. Measured interplanar spacings: $d_{(200)}$ = 2.74 Å and $d_{(022)}$ = 1.94 Å.

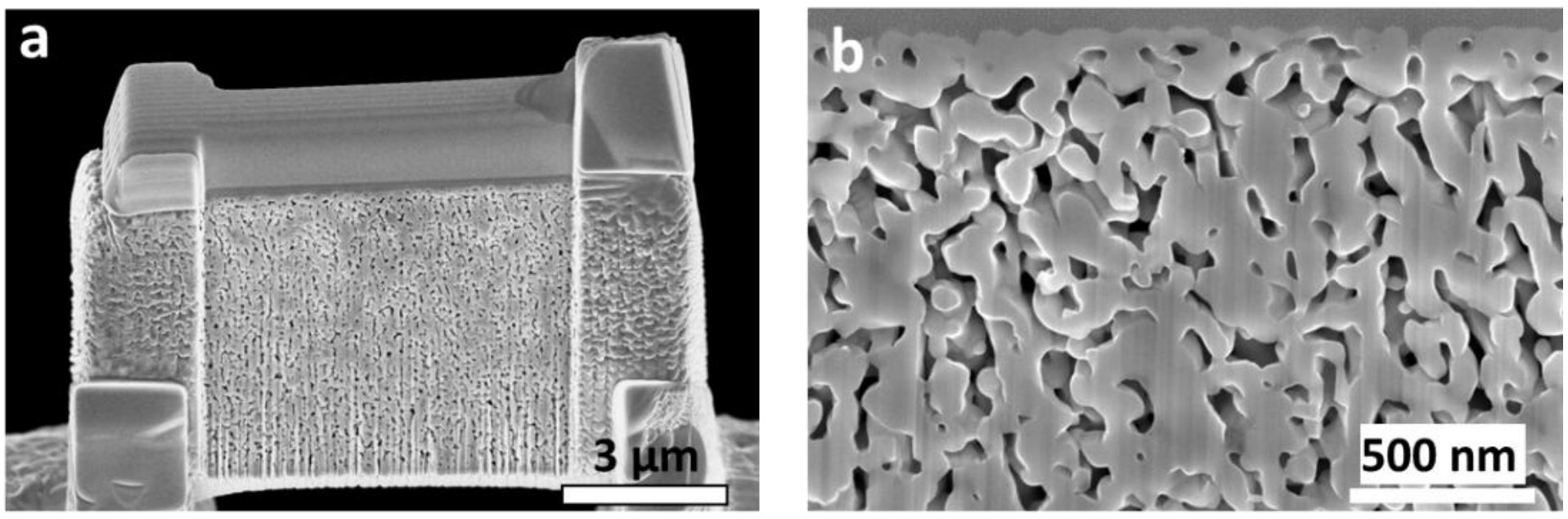

Figure S2. Sample preparation and cross-sectional structural characterization of PSC ZB-ZnO. (a) FIB-fabricated lamella prepared for subsequent TEM specimen thinning. (b) SEM image showing the three-dimensional porous architecture.

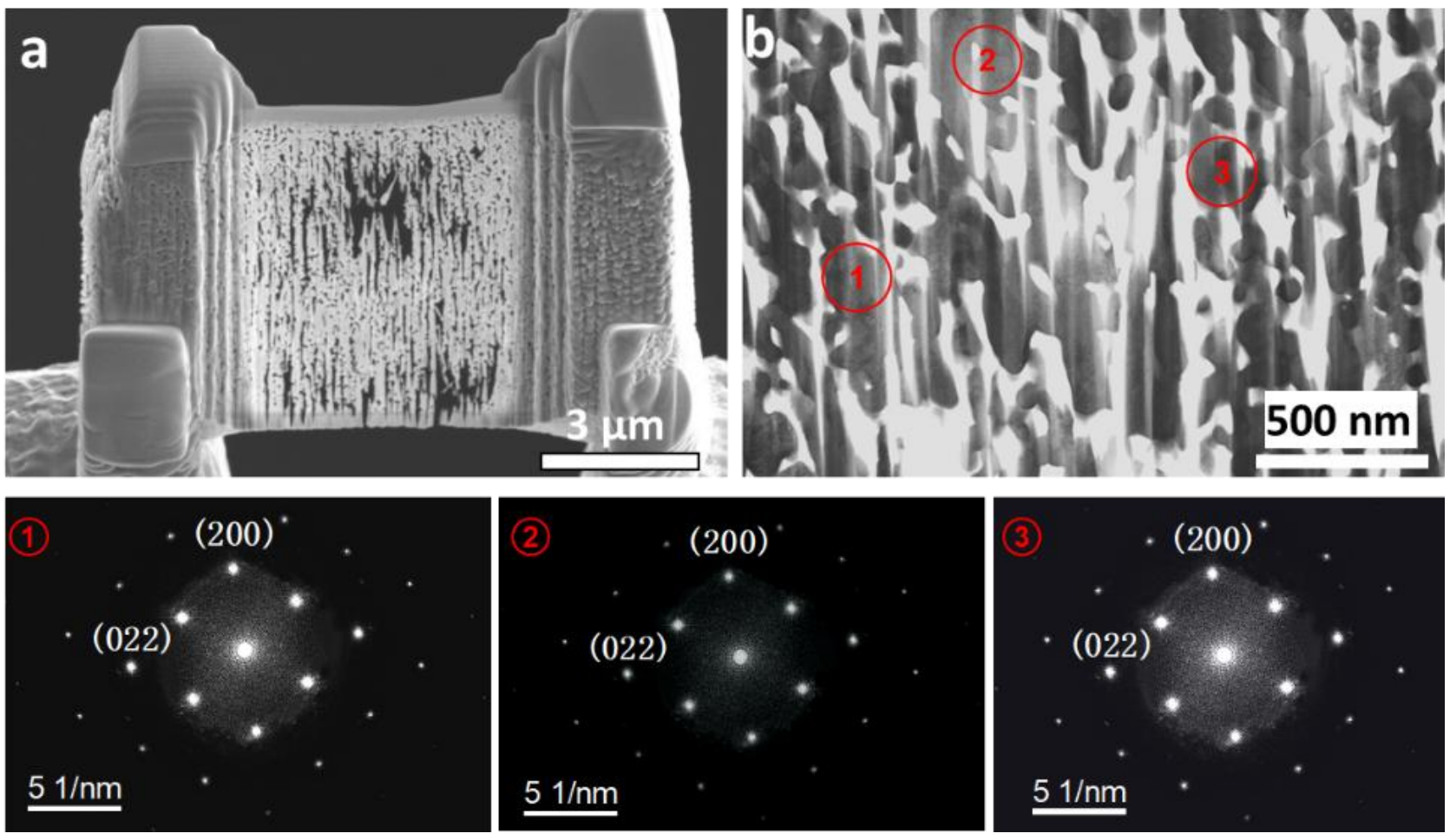

Figure S3. Microstructural characterization of PSC ZB-ZnO. (a) FIB-sectioned lamella prepared for TEM analysis. (b) STEM image revealing three-dimensional porosity. (1-3) SAED patterns acquired from distinct regions (circled in b), indexed to $[02\bar{2}]$ zone axis, with (200) and (022) reflections labeled.

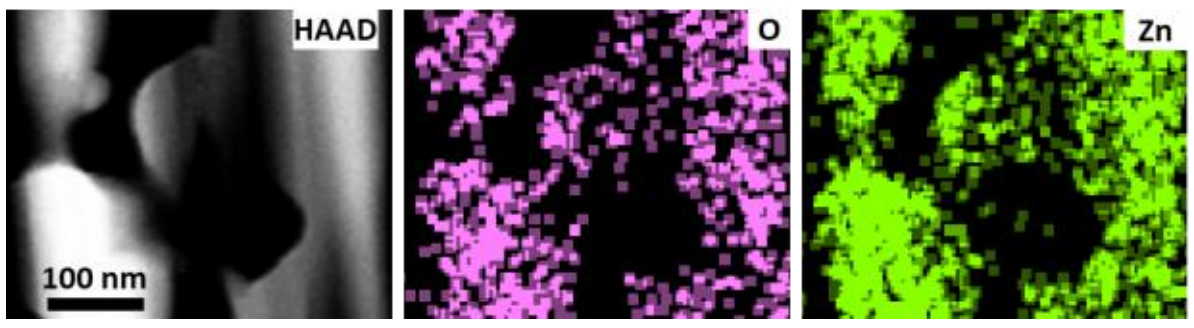

Figure S4. EDS mapping of the PSC ZB-ZnO. No residual S signals are detected.

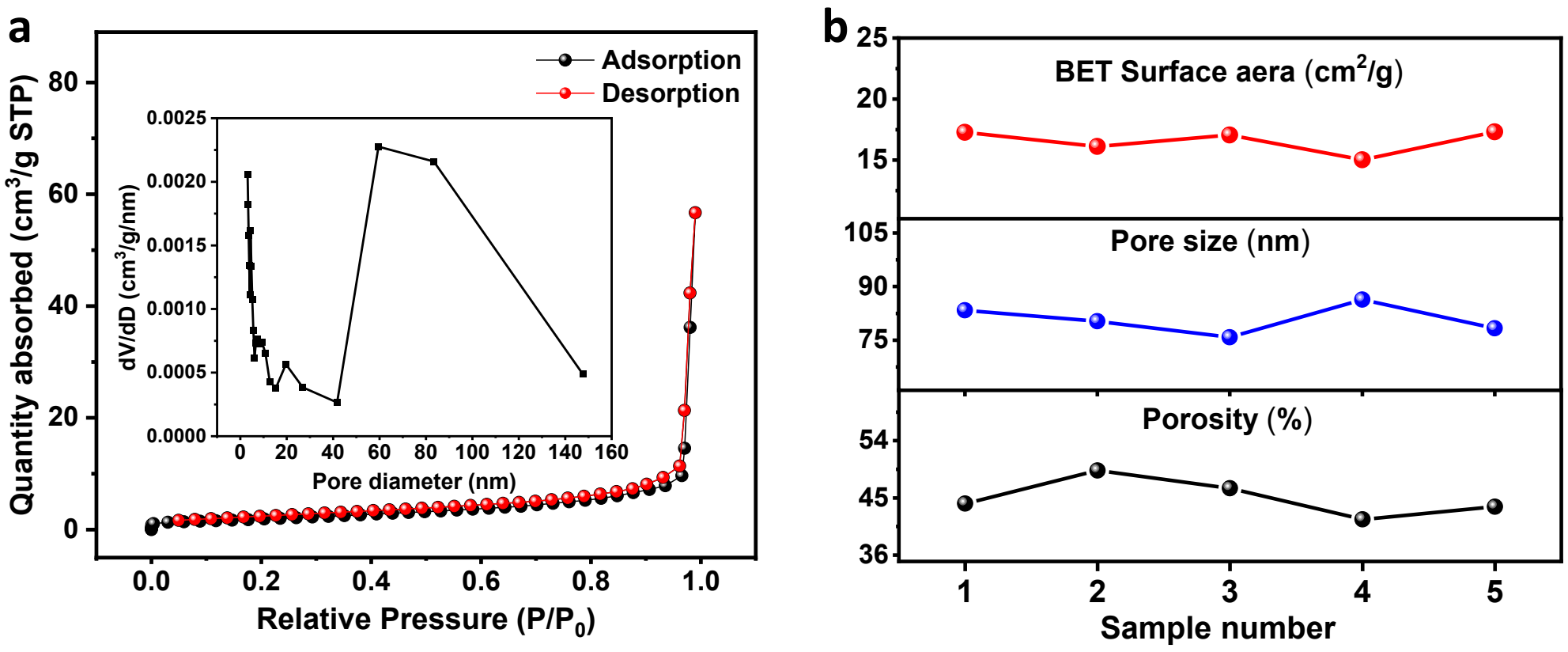

Figure S5. Pore structure characterization of PSC ZB-ZnO. (a) Nitrogen adsorption/ desorption isotherms with the corresponding pore-size distribution curve shown in the inset. (b) BET surface area, pore size and porosity of the synthesized samples.

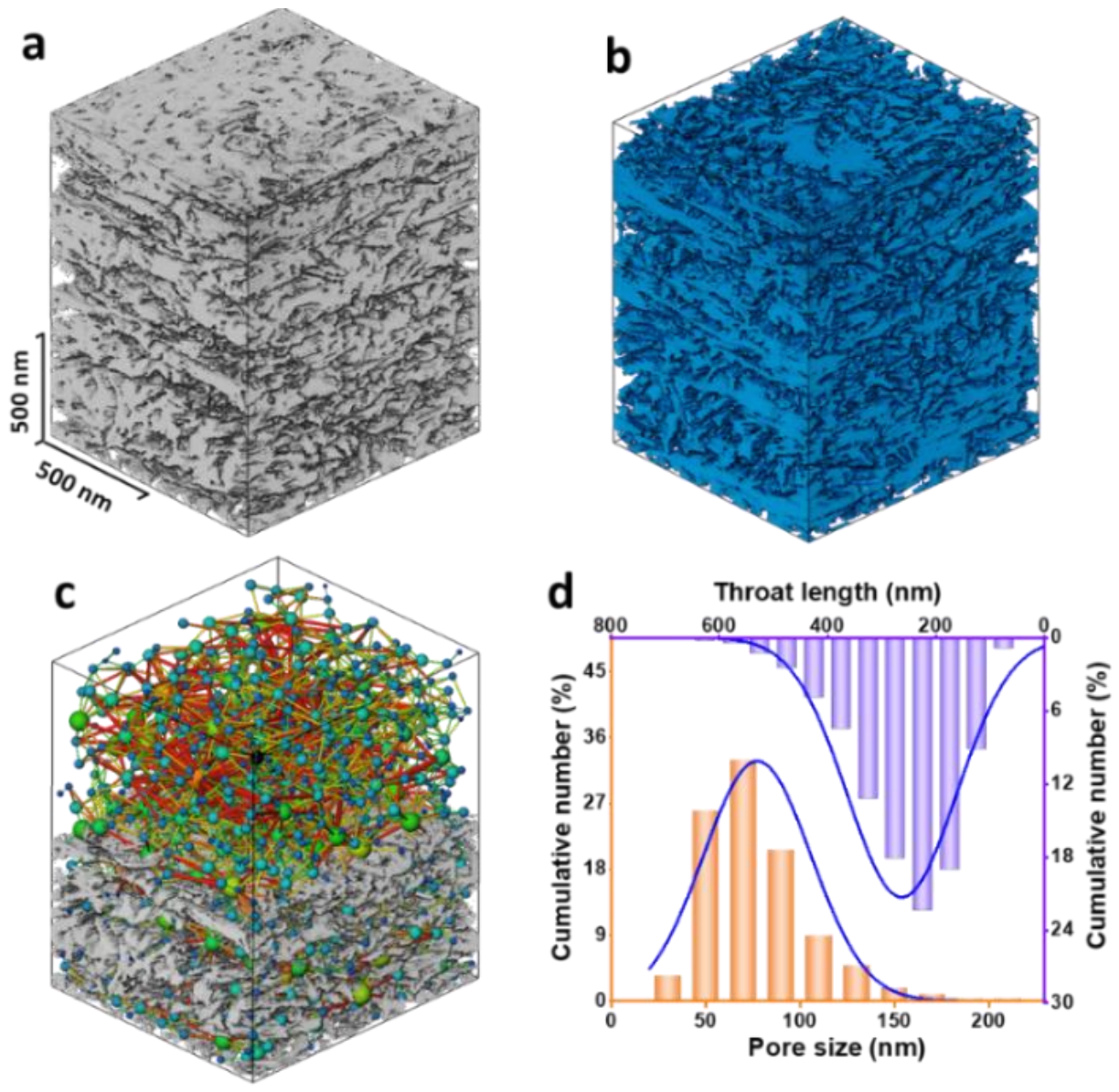

Figure S6. 3D dimensional reconstruction analysis of PSC ZB-ZnO: (a) skeletal framework structure, (b) pore system, (c) ball-stick model of interconnected pores and throats, (d) pore size and throat length distribution.

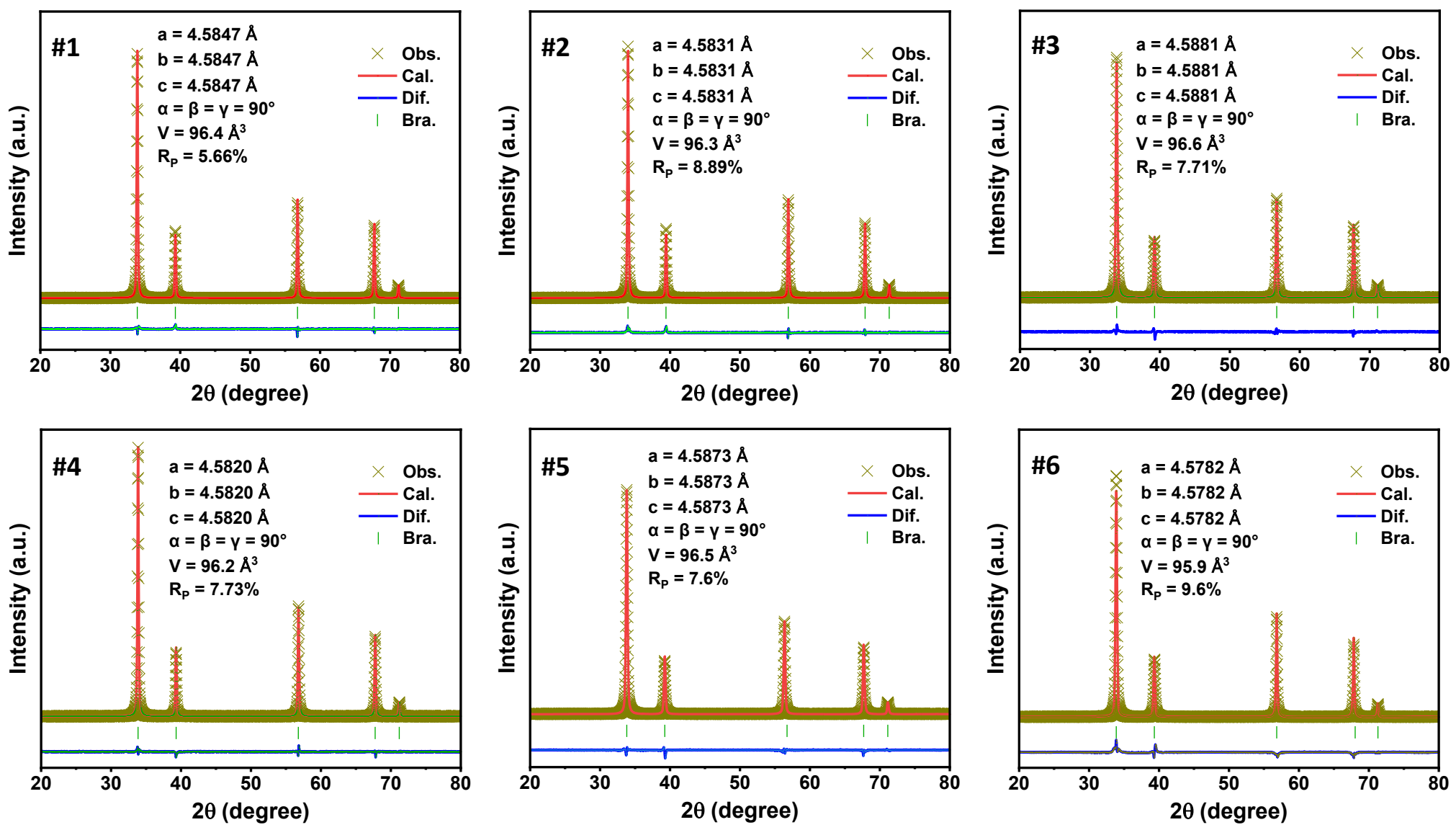

Figure 7. XRD Rietveld refinement plots for PSC ZB-ZnO samples from multiple independent synthesis batches.

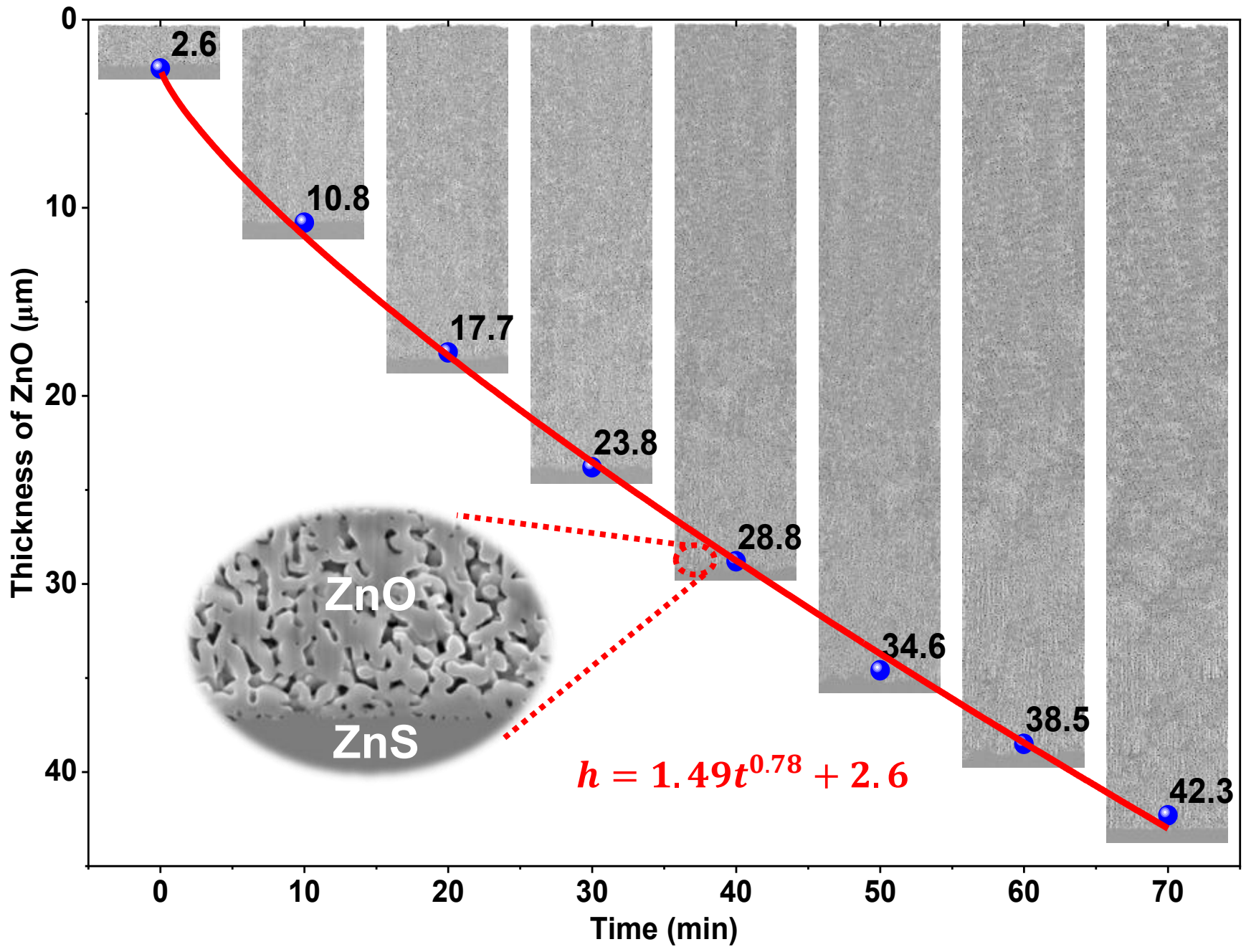

Figure S8. Cross-sectional morphology and time-resolved growth kinetics of ZB-ZnO on ZnS. Layer thickness (h) versus growth time (t) measured via 3D ion slicing (data points: 2.6, 10.8, 17.7, 23.8, 28.8, 34.6, 38.5, 42.3 nm). Solid curve: Power-law fit h = $1.49t^{0.78} + 2.6$ (R^2>0.99). Inset represents ZnO/ZnS interfacial microstructure.

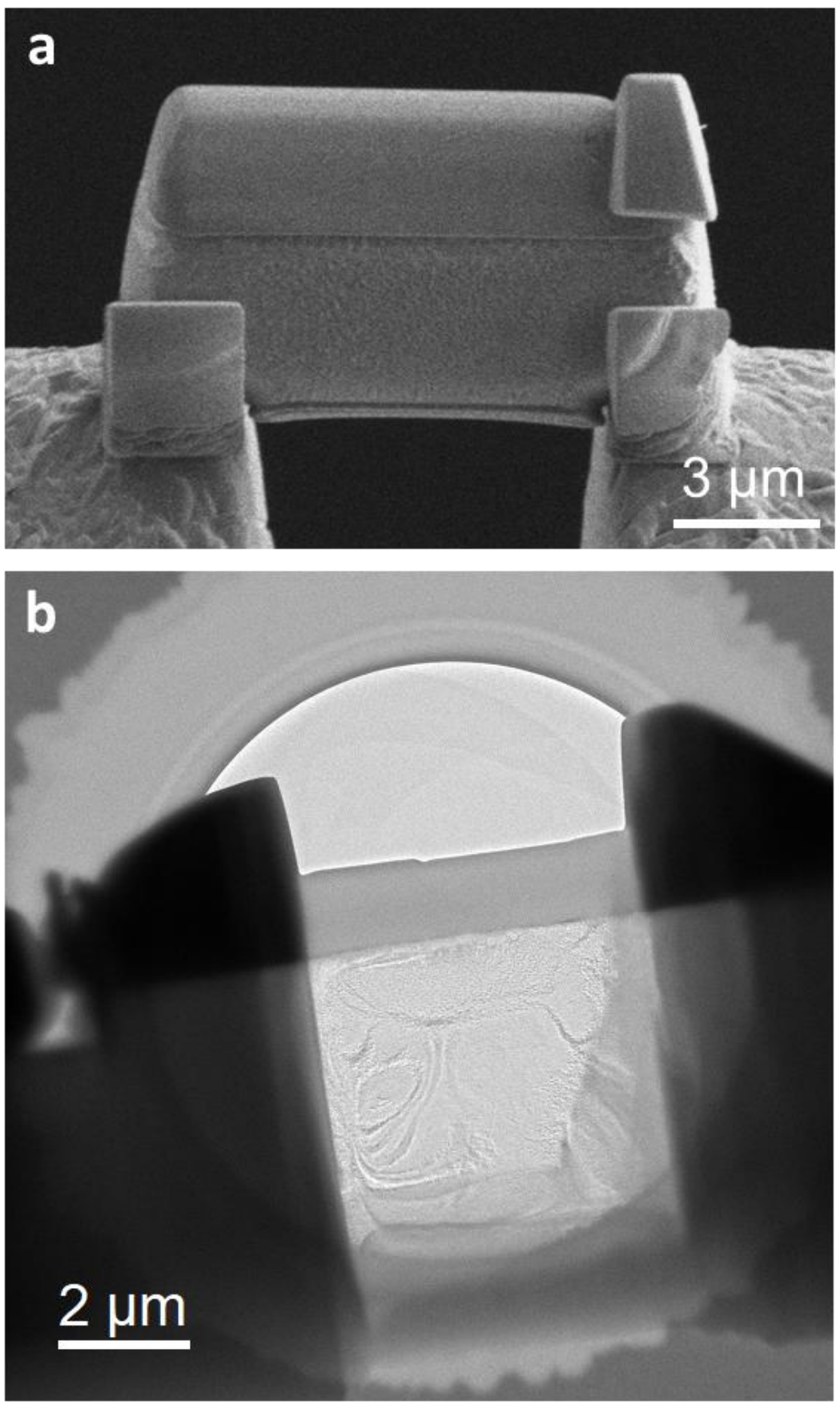

Figure S9. In situ TEM specimen preparation. (a) FIB-fabricated ZnS lamella. (b) Lamella mounted on a DENS solutions Wildfire heating chip via Pt deposition, enabling atomic-resolution observation under thermal stimuli.

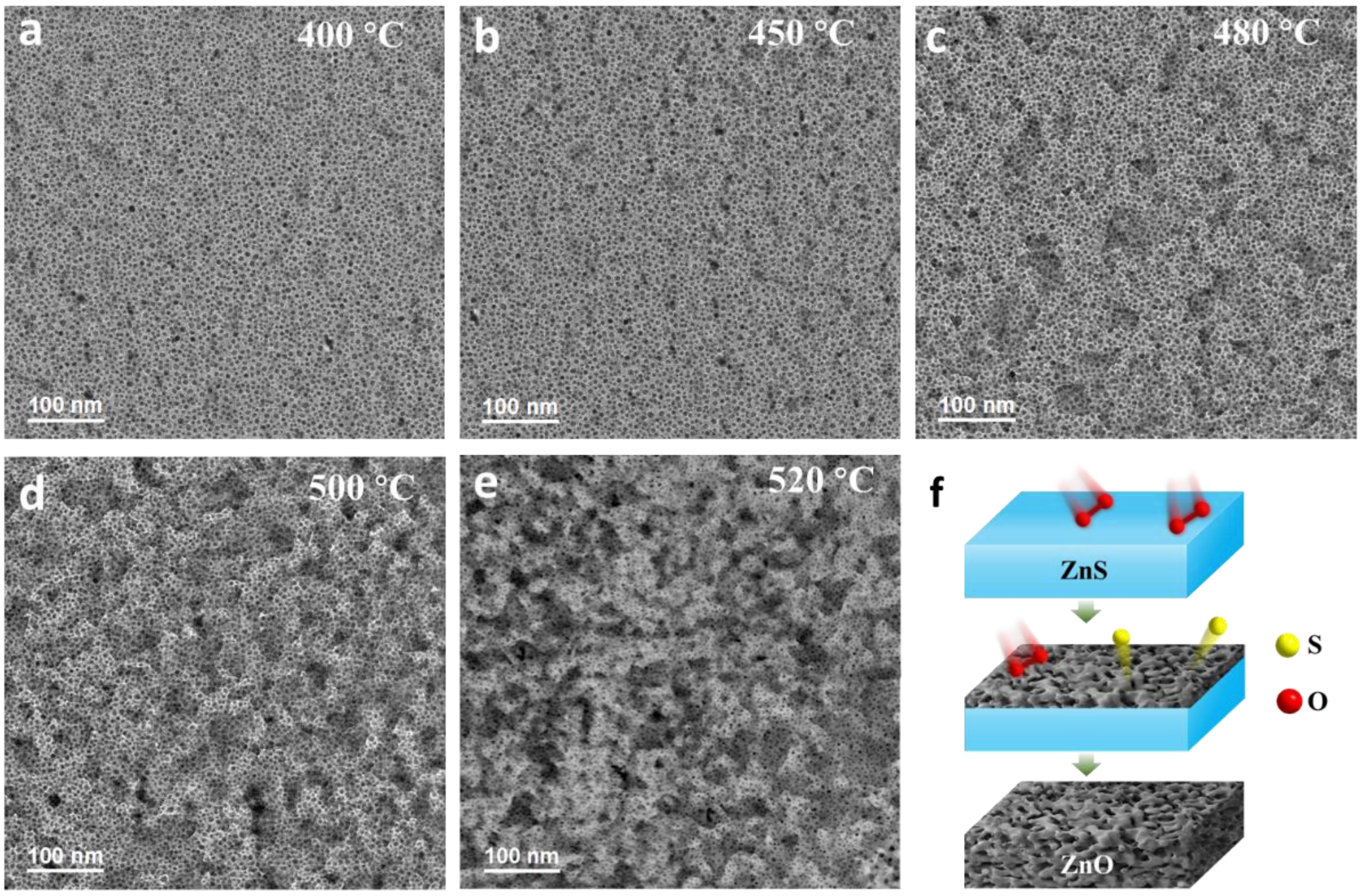

Figure S10. In situ TEM observation of pore evolution in ZB-ZnO under thermal treatment. (a-e) Sequential images showing pore formation in O_2 atmosphere during continuous heating (460→520 °C at 10 °C/min). (f) Schematic illustration of the formation of porous ZnO structures.

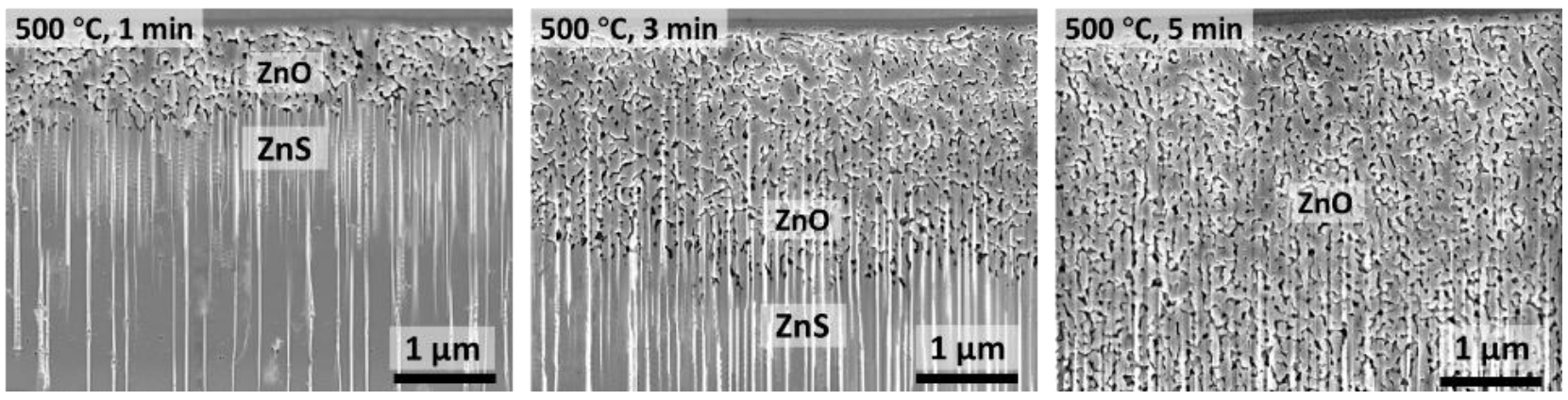

Figure S11. Time dependent FIB-SEM images of PSC ZB-ZnO grown on ZnS. At 1 min, a distinct interface between ZnS (precursor) and newly formed porous ZnO is observed, indicating the initiation of the topotactic lattice contraction process. By 3 minutes, the ZnS core has become partially consumed, and a continuous porous structure composed of ZnO continues to grow downward. At 5 minutes, the ZnS has almost completely transformed into PSC ZnO throughout the observed area.

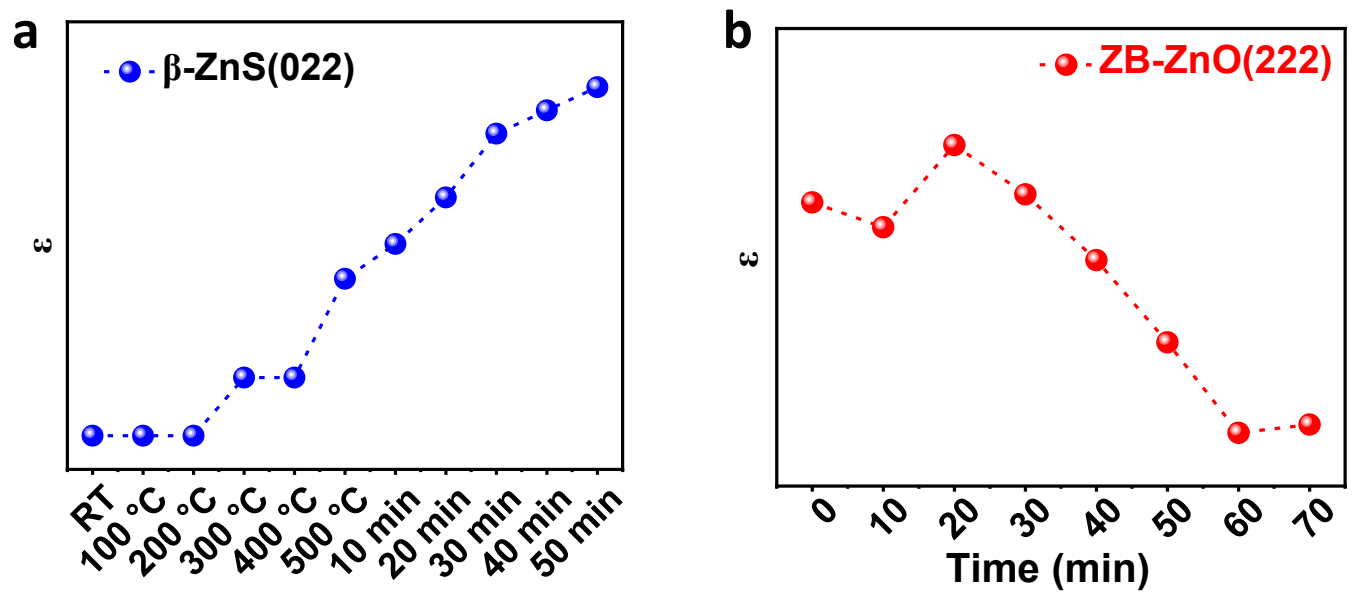

Figure S12. Strain evolution of (a) ZnS and (b) PSC ZB-ZnO during the transition.

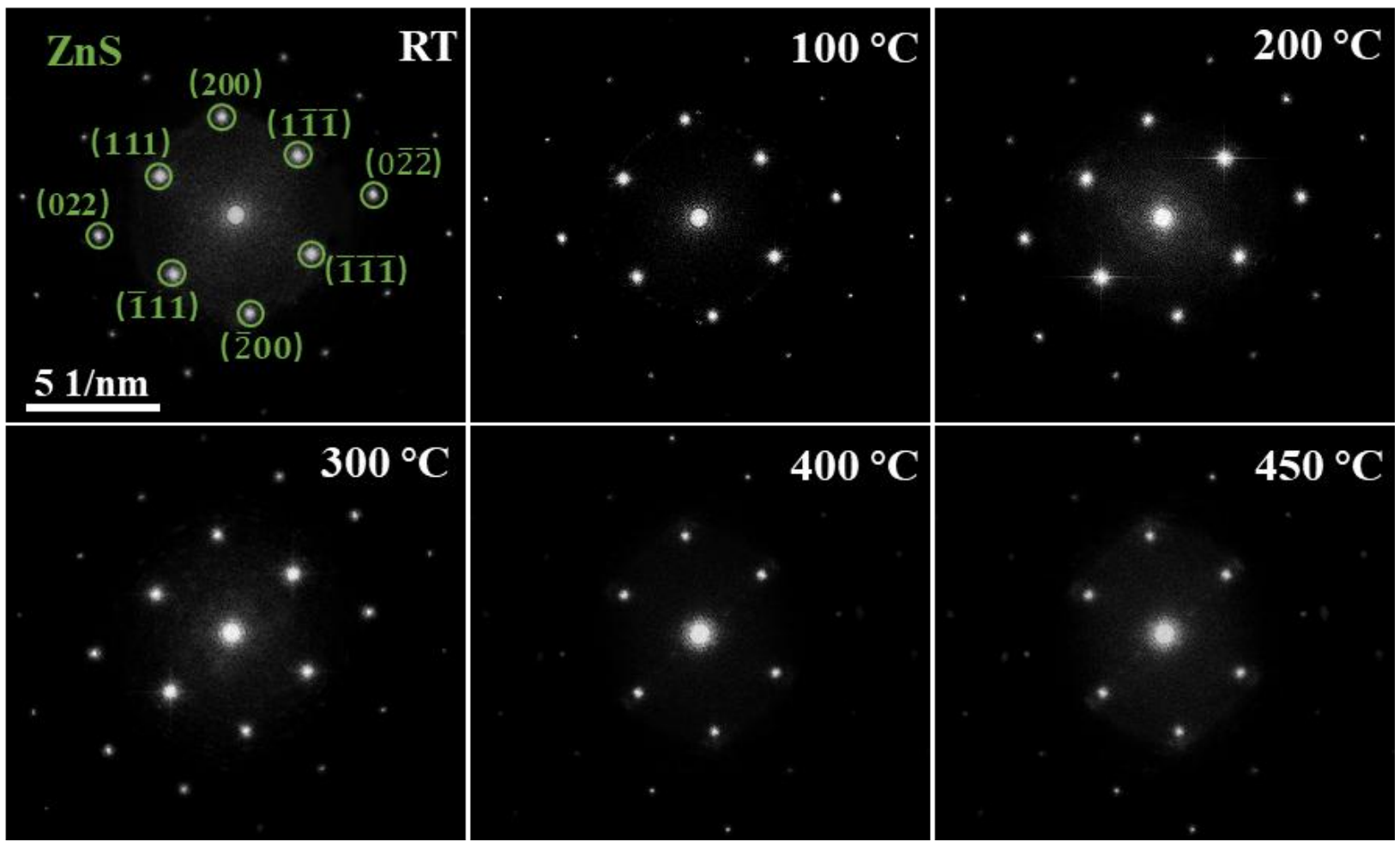

Figure S13. SAED pattern of β-ZnS single crystal acquired via in situ TEM during thermal process under 5×10^{-4} Torr O_2. No observable changes with temperature increase below 450 °C.

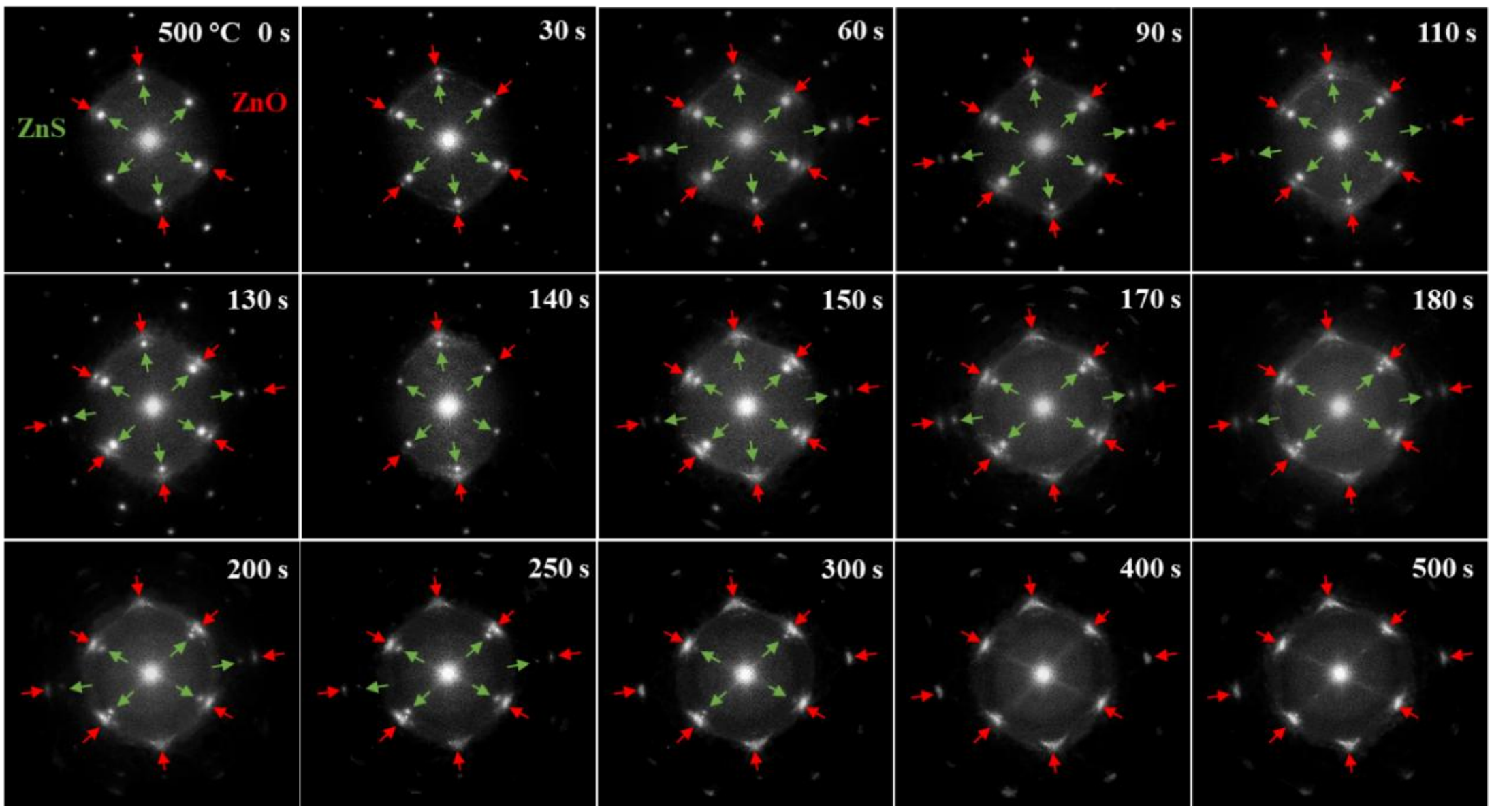

Figure S14. SAED pattern during topotactic transition from β-ZnS single crystal to ZB-ZnO single crstal acquired via in situ TEM under 5×10^{-4} Torr O_2. Sequential SAED evolution under isothermal oxidation (500 °C, 0→500 s).

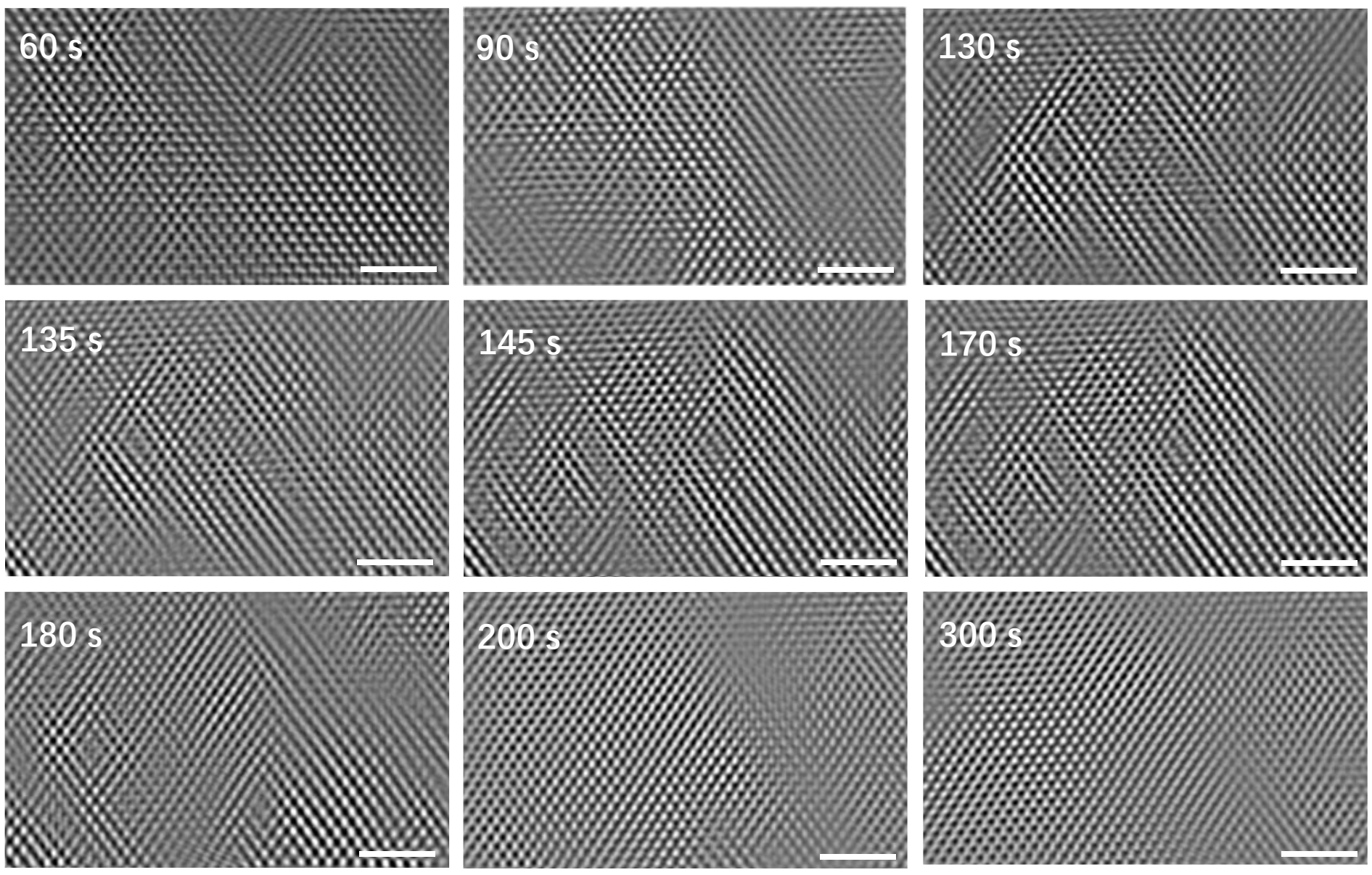

Figure S15. In situ HRTEM images of the topotactic transition process from cubic ZnS to ZB-ZnO at 500 °C, the scalebar is 2 nm.

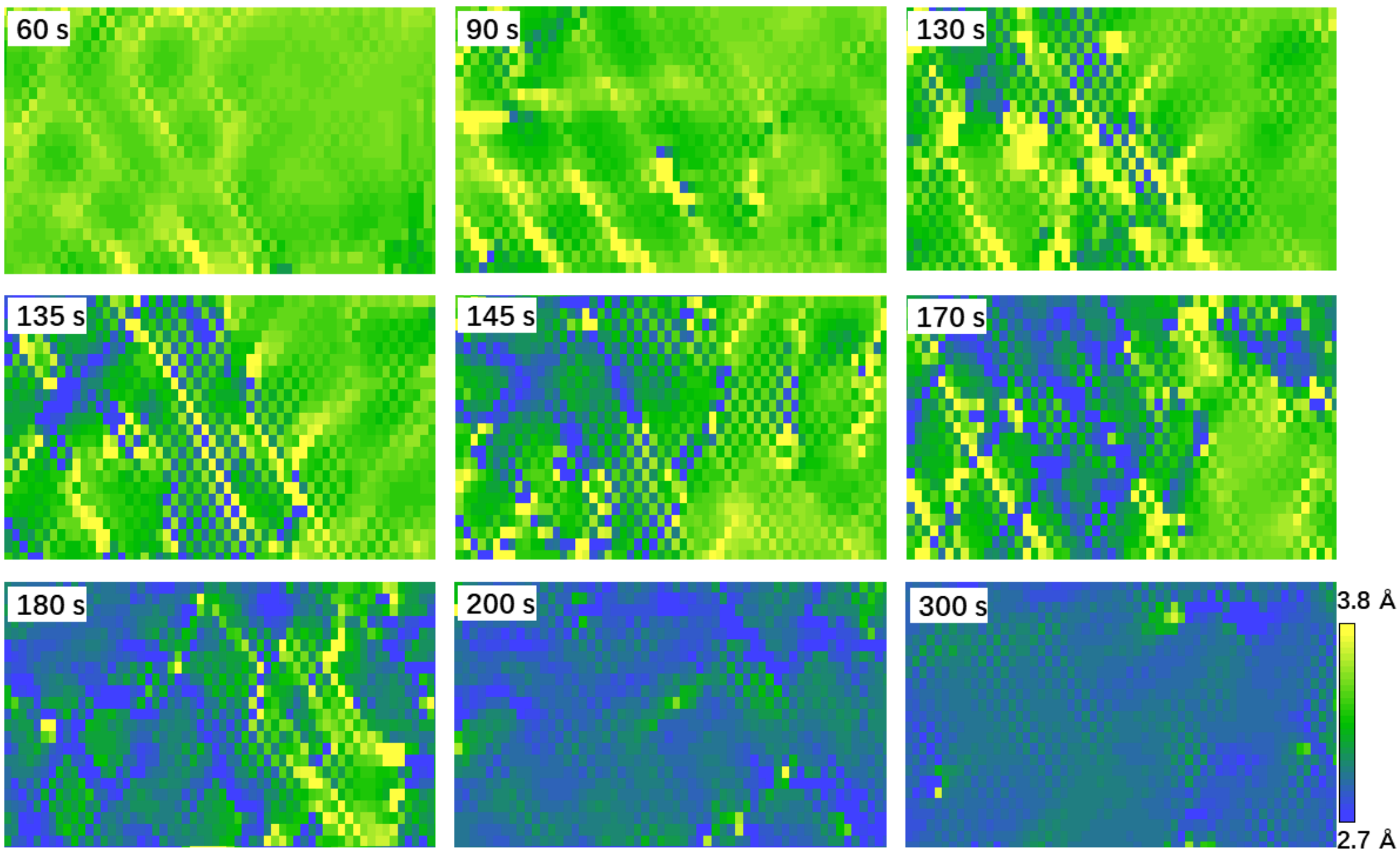

Figure S16. Spatiotemporal evolution of lattice dynamics. Time-resolved evolution of interatomic spacing ($\Delta d/d_0$) along the ⟨112⟩ direction showing: (i) Preferential expansion of {111} planes (yellow, $\Delta d/d_0 > +4.8\%$), forming percolative networks within 60 s, (ii) Multi-site lattice contraction (blue, $\Delta d/d_0 < -2.3\%$) nucleating along pre-expanded pathways at 90 s, (iii) Contraction front propagation through pre-established expanded channels, culminating in global lattice collapse (>300 s).

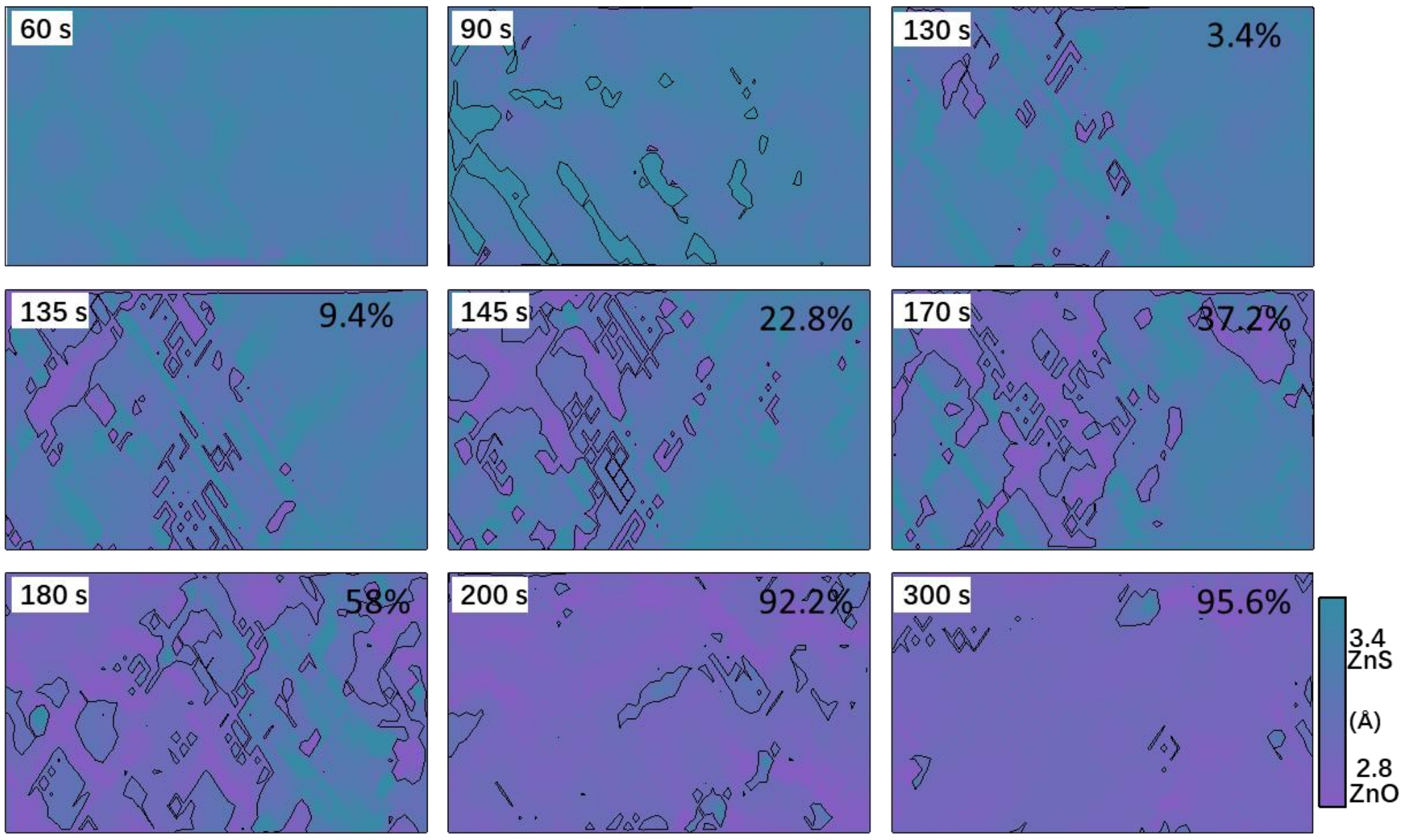

Figure S17. Contour plot of the interatomic spacing along the ⟨112⟩ direction, which was extracted from Figure S9. Interatomic spacing of 2.8 Å corresponds to ZB-ZnO (pink reference). Multi-site nucleation initiates at 90 s and propagates anisotropically along ⟨112⟩, reaching near-complete phase transformation by 300 s.

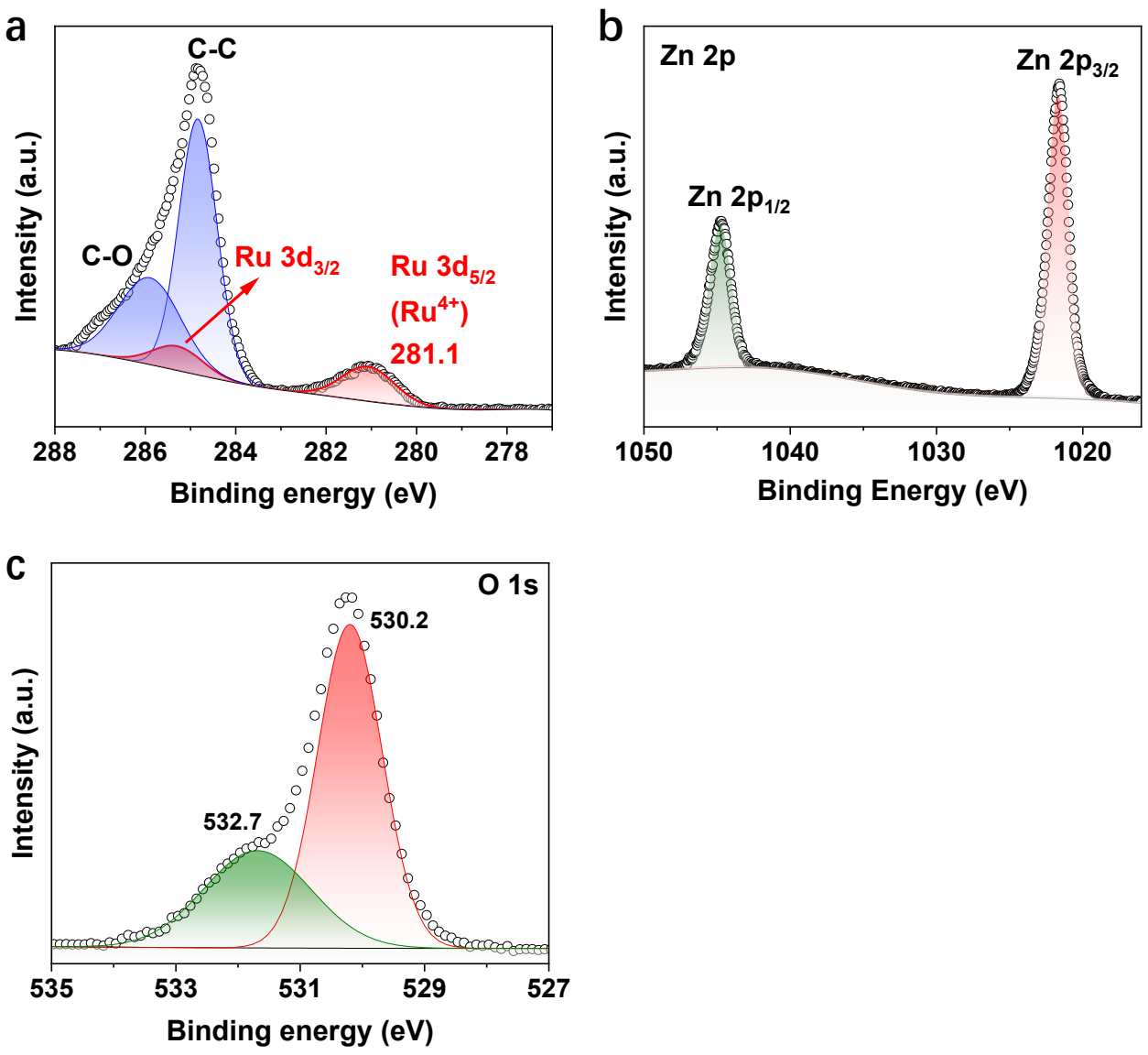

Figure S18. X-ray photoelectron spectrum of Ru_1/ZB-ZnO. (a) Ru 3d spectrum showing Ru^{4+} species (Ru $3d_{3/2}$ at 281.1 eV, red arrow) with characteristic spin-orbit splitting ($3d_{5/2}$: 285.3 eV). (b) Zn 2p doublet ($2p_{3/2}$: 1021.5 eV, $2p_{1/2}$: 1044.6 eV). (c) O 1s deconvolution revealing lattice oxygen (520.2 eV) and surface hydroxyl groups (532.7 eV). All spectra charge-referenced to C 1s (284.8 eV).

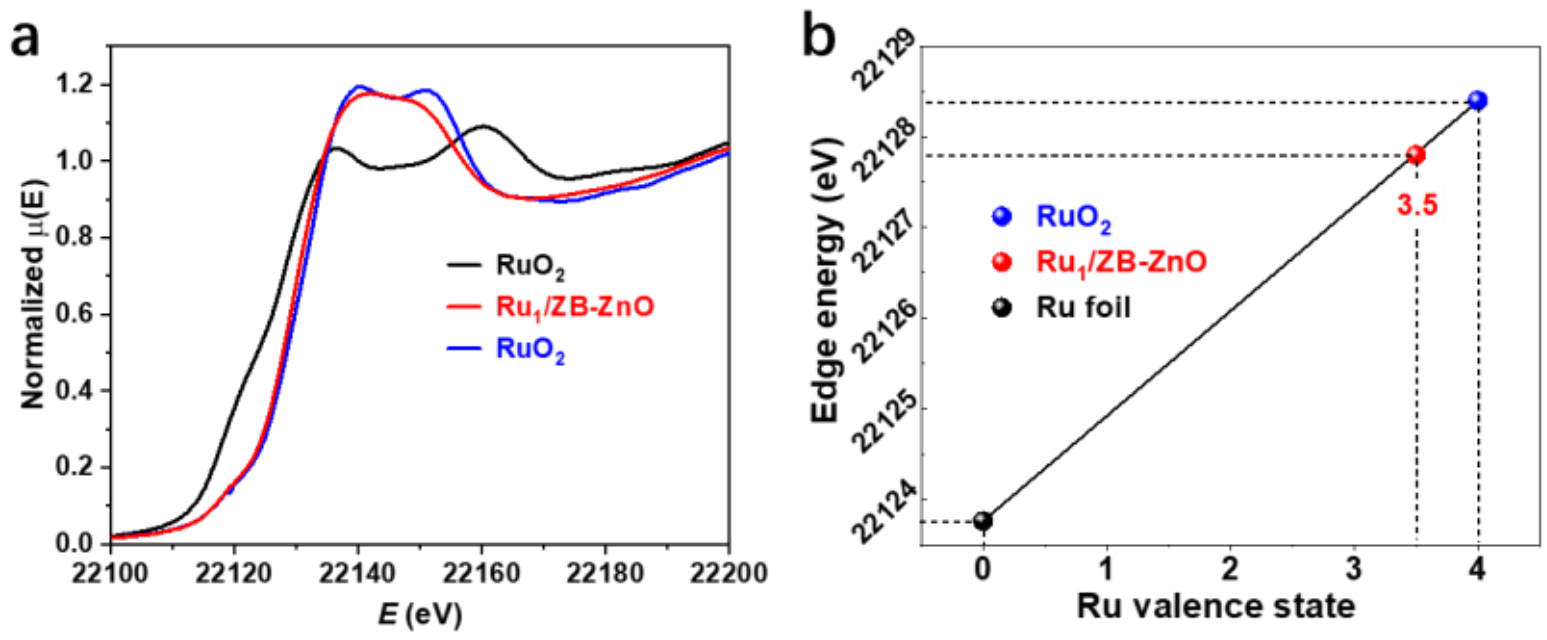

Figure S19. Normalized Ru K-edge XANES and valence-dependent edge energy analysis of Ru_1/ZB-ZnO with RuO_2 foil and Ru as references. (a) Normalized XANES spectra showing Ru/ZB-ZnO (red) exhibits intermediate absorption features between Ru foil (black) and RuO_2 (blue). (b) Linear correlation between Ru valence state and edge energy shows that Ru_1/ZB-ZnO (red dot) exhibits a valence state of approximately +3.5.

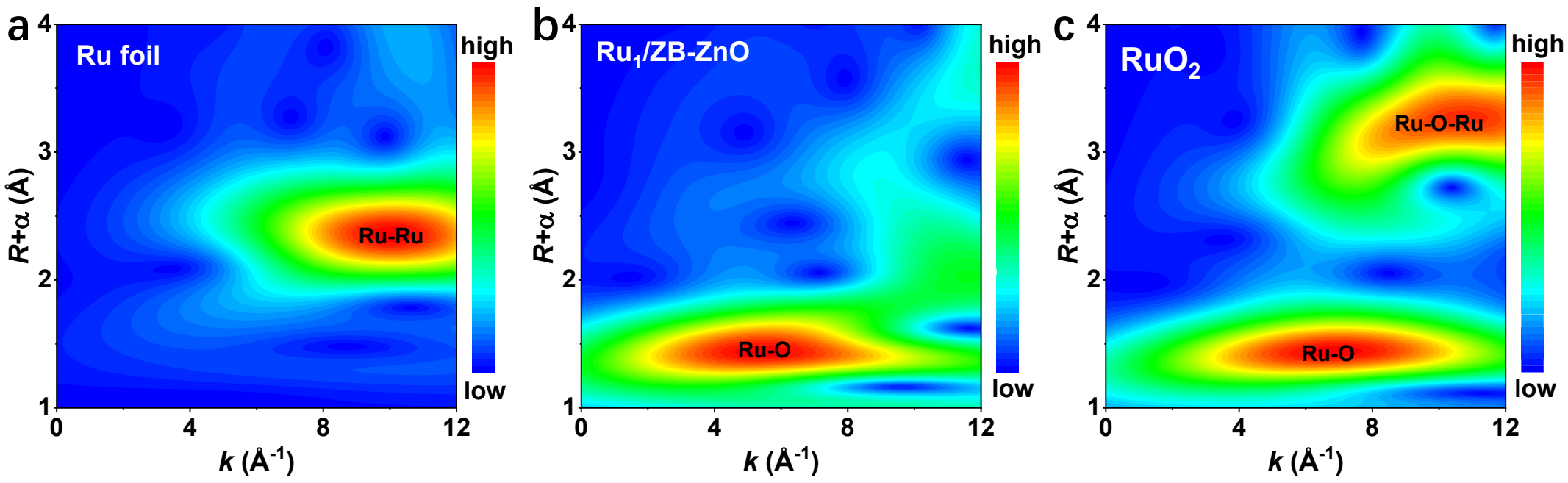

Figure S20. Wavelet transforms for the k^3-weighted EXAFS signals of Ru. (a) Ru foil. (b) Ru_1/ZB-ZnO. (c) RuO_2.

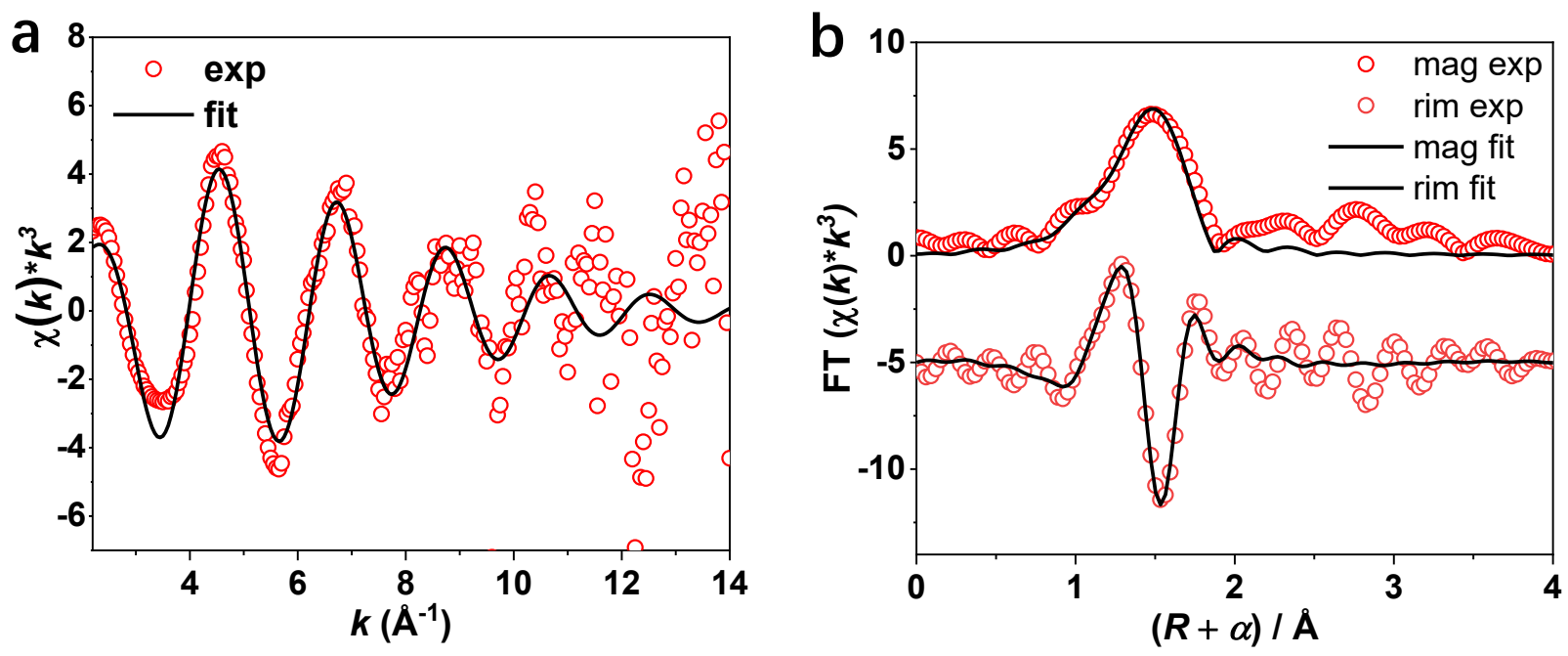

Figure S21. Ru K-edge EXAFS analysis of Ru_1/ZB-ZnO. (a) Ru K-edge EXAFS (cycles) and the fit curves (line) Ru_1/ZnO, shown in k^3 weighted k-space. (b) Fourier transform magnitude (circle) and imaginary component (circles) in R-space with fit (line), revealing dominant Ru-O bond at 1.49 Å (no Ru-Ru contribution). Data: k^3-weighted, not phase-corrected.

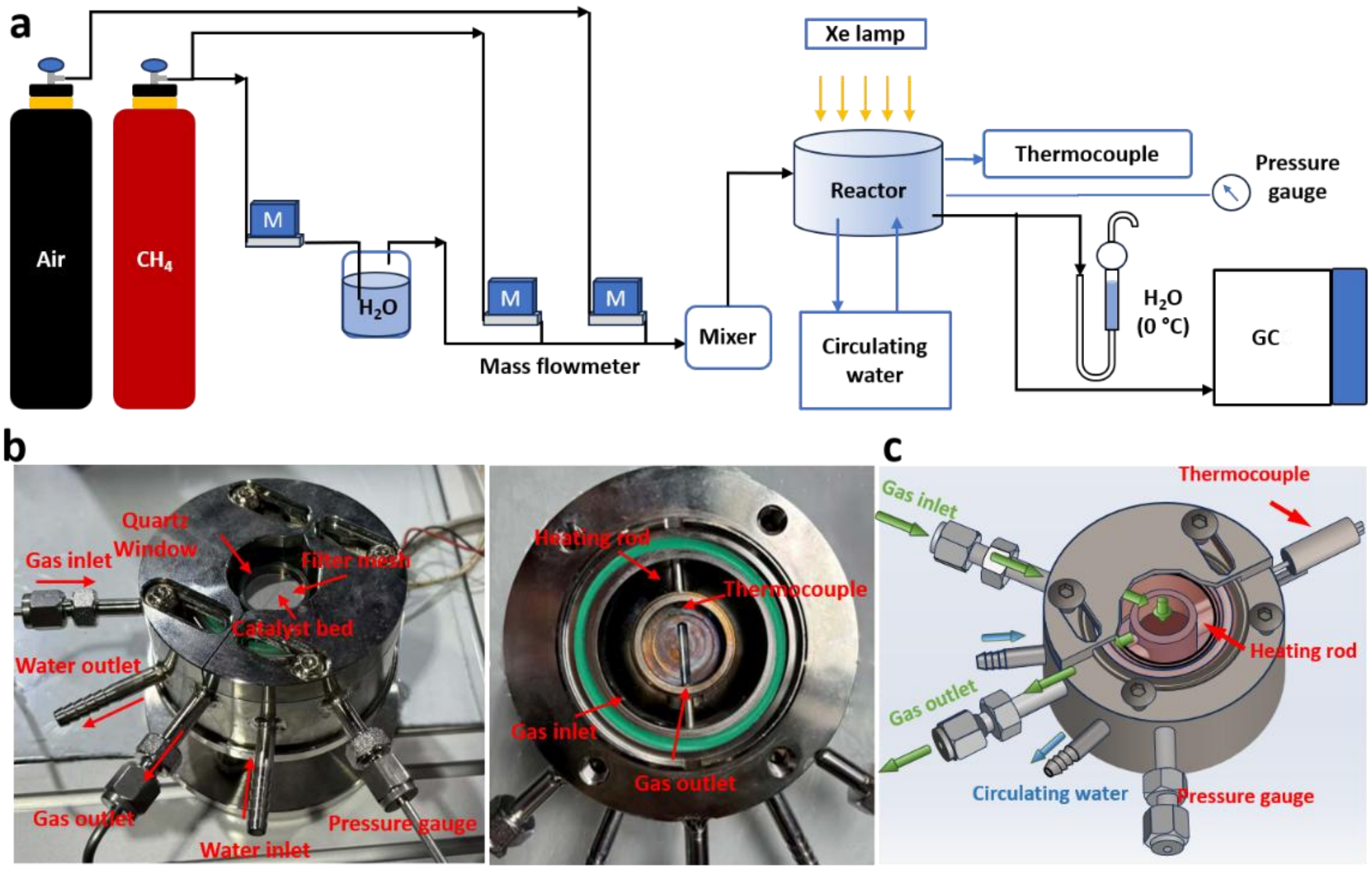

Figure S22. Equipment diagram. a, Schematic diagram of the experimental setup. **b,** Physical photo of the continuous-flow gas-solid photoreactor for photocatalytic oxidation of CH_4. **c,** Schematic diagram of the photoreactor.

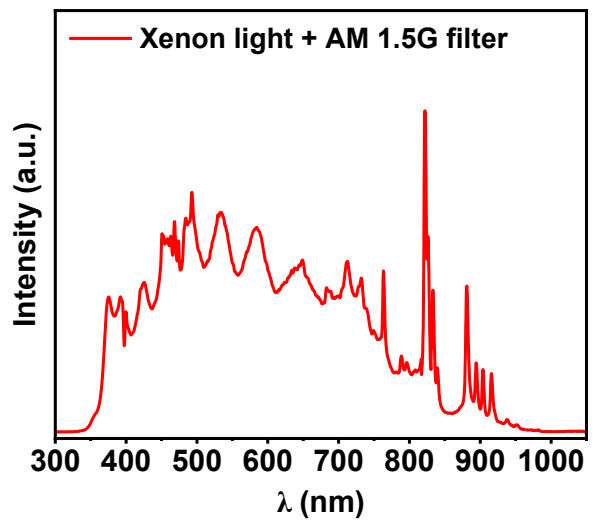

Figure S23. The spectrum of xenon lamp light with 1.5G filter.

The light intensity is calculated as follows:

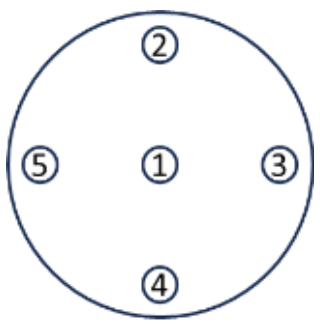

$$E = \frac{\bar{E}_{center}}{3} + \frac{2*(\bar{E}_{edge})}{3} = \frac{\bar{E}_{center}}{3} + \frac{2*(E_{edge2}+E_{edge3}+E_{edge4}+E_{edge5})}{3*4}$$

$\bar{E}_{center}$: The light intensity value at the center (spot 1)

E_{edge2}、E_{edge3}、E_{edge4}、E_{edge5}： The light intensity values at the edge (spot 2、3、4、5)

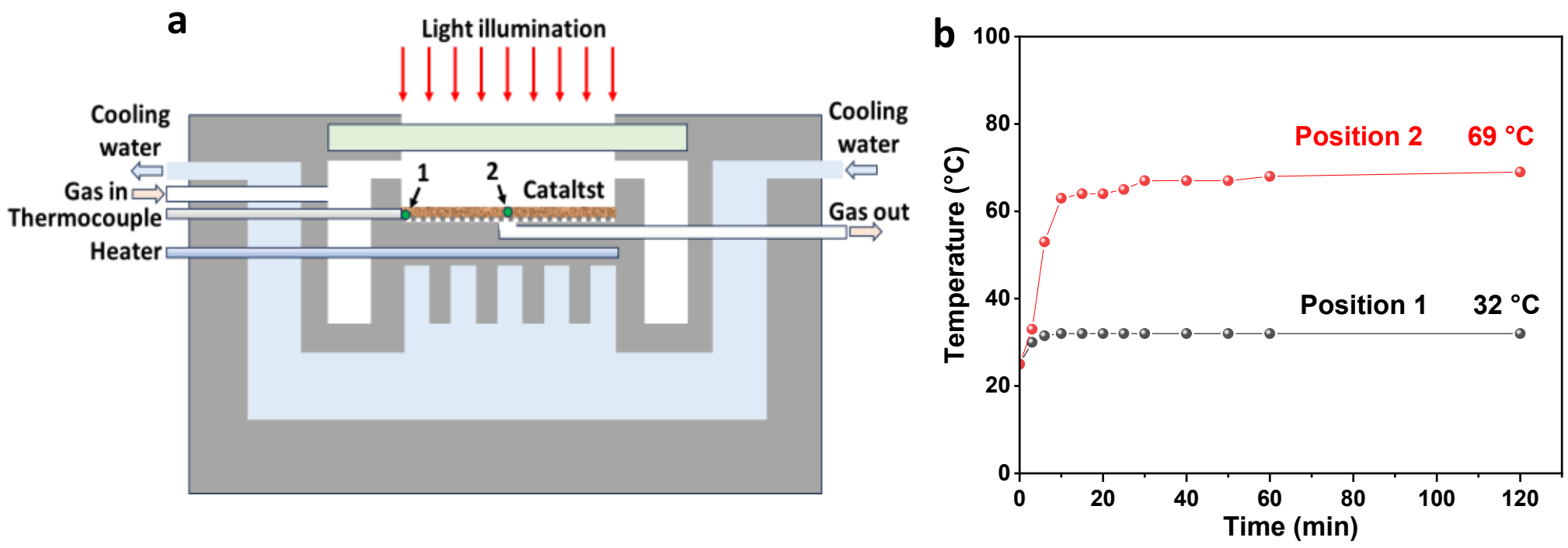

Figure S24. Catalyst surface temperature detection in gas−solid photoreactor equipped with a circulating water system. **a,** Schematic diagram of test positions. **b,** The temperature of different positions detected by a thermometer under light at different reaction time during photothermal catalytic oxidation. The temperature at the interface between the catalyst and the reactor wall (position 1) stabilized at 32°C after more than ten minutes, indicating that the circulating water effectively reduces the temperature around the reactor wall to a low level. The temperature near the upper surface at the center of the catalyst bed (Position 2) stabilized at 79 °C with the cooling system in operation under our photooxidation conditions.

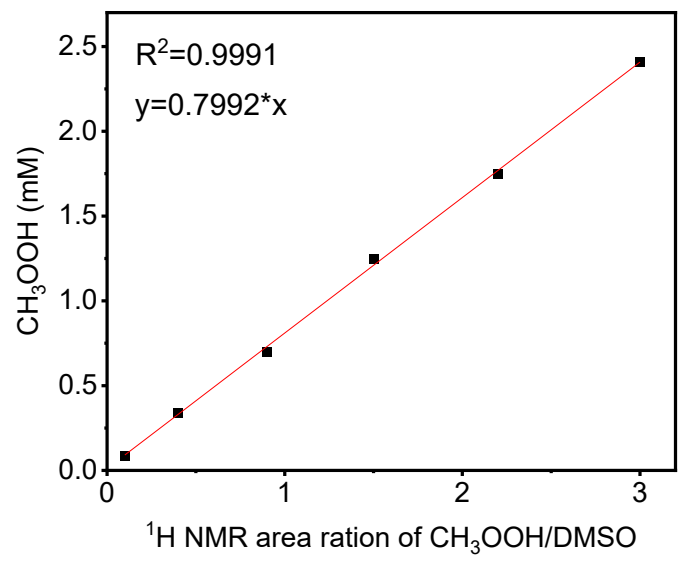

Figure S25. ^{1}H NMR calibration curve of CH_3OOH using DMSO as an internal standard. Given the identical methyl proton count in both CH_3OH and CH_3OOH, the calibration curve used for CH_3OOH quantification applies equally to CH_3OH.

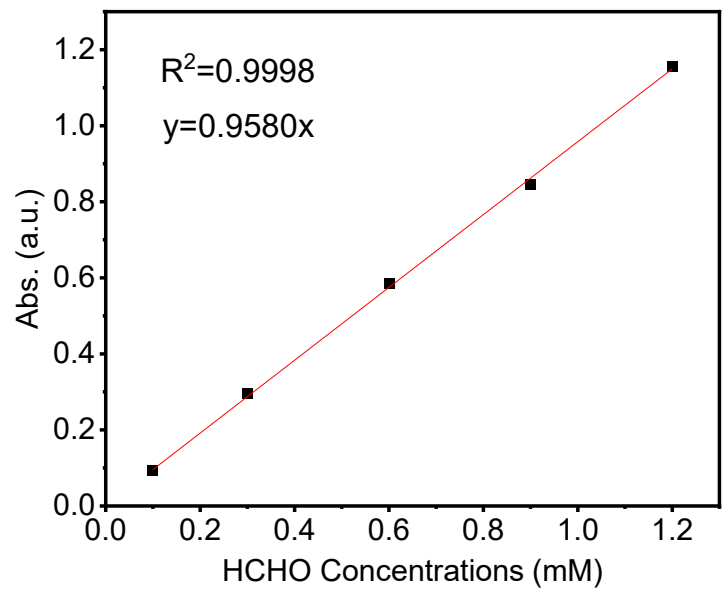

Figure S26. Calibration curve of HCHO. Calibration curve for quantification of HCHO by the colorimetric method.

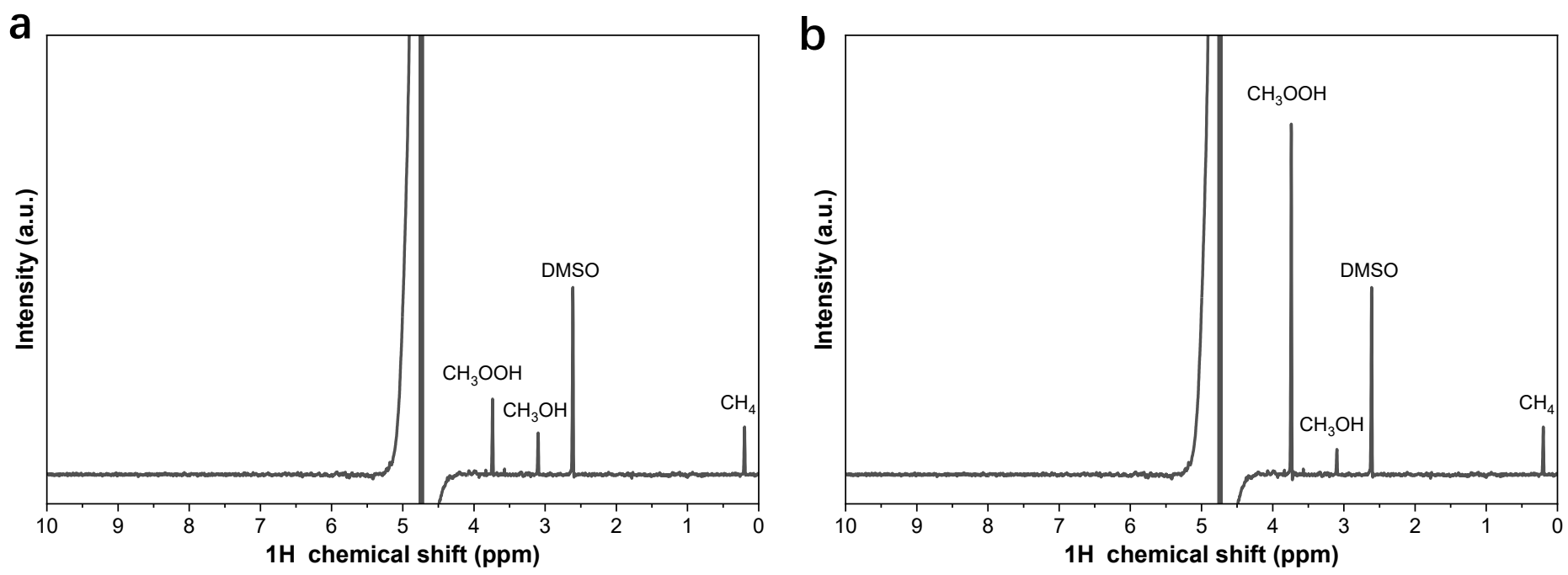

Figure S27. ^{1}H NMR spectrum of the liquid product obtained from photocatalytic methane oxidation over (a) ZB-ZnO and (b) 0.6wt % Ru_1/ZB-ZnO for 2 h.

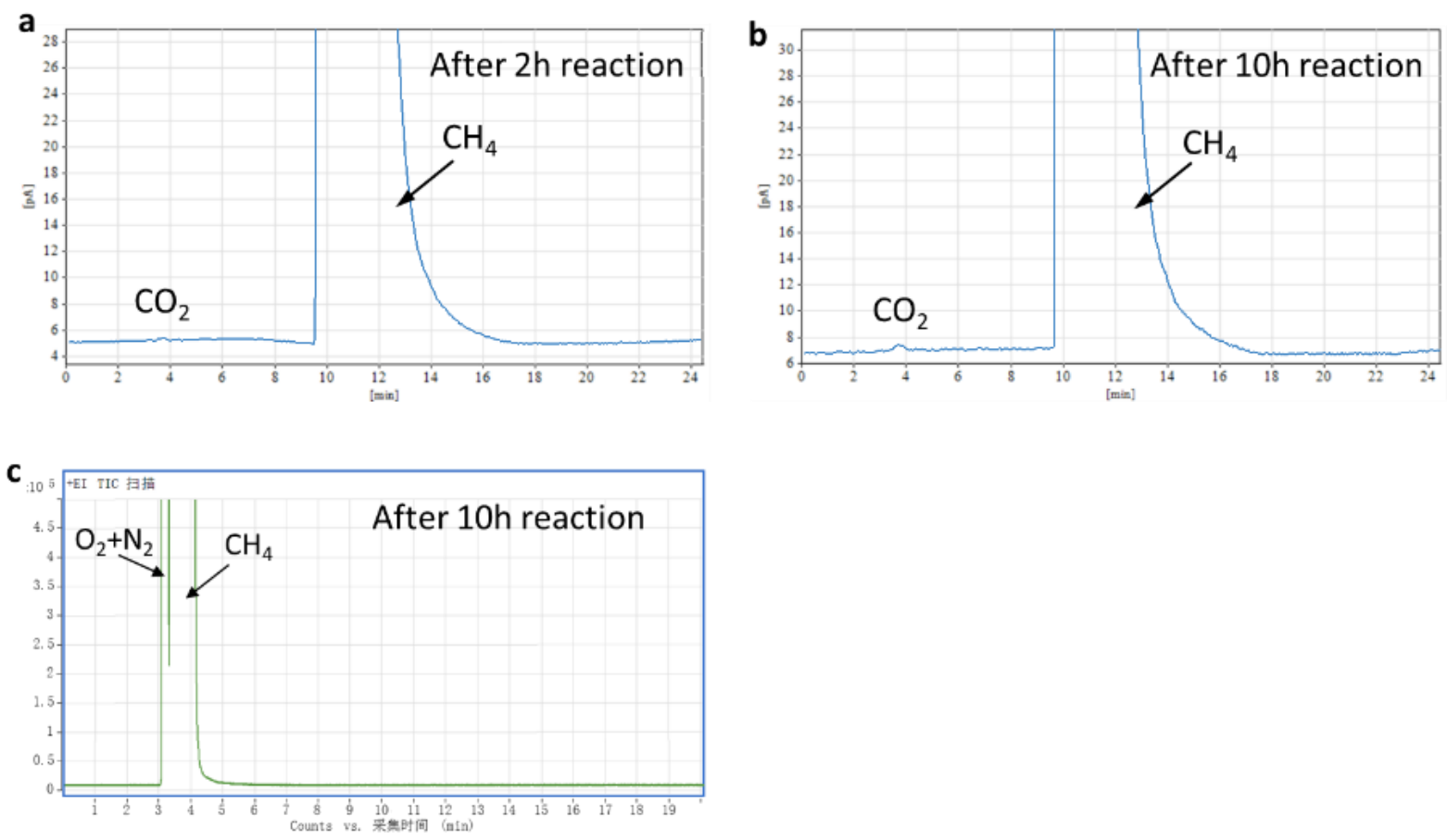

Figure S28. Original GC-FID spectras for outlet gas after photocatalytic methane oxidation over Ru_1/PSC ZB-ZnO for (a) 2 h and (b) 10 h reaction. (c) GC-MS total ion chromatograms (TICs) confirmed no co-eluting peaks or unresolved species after 10 hours reaction.

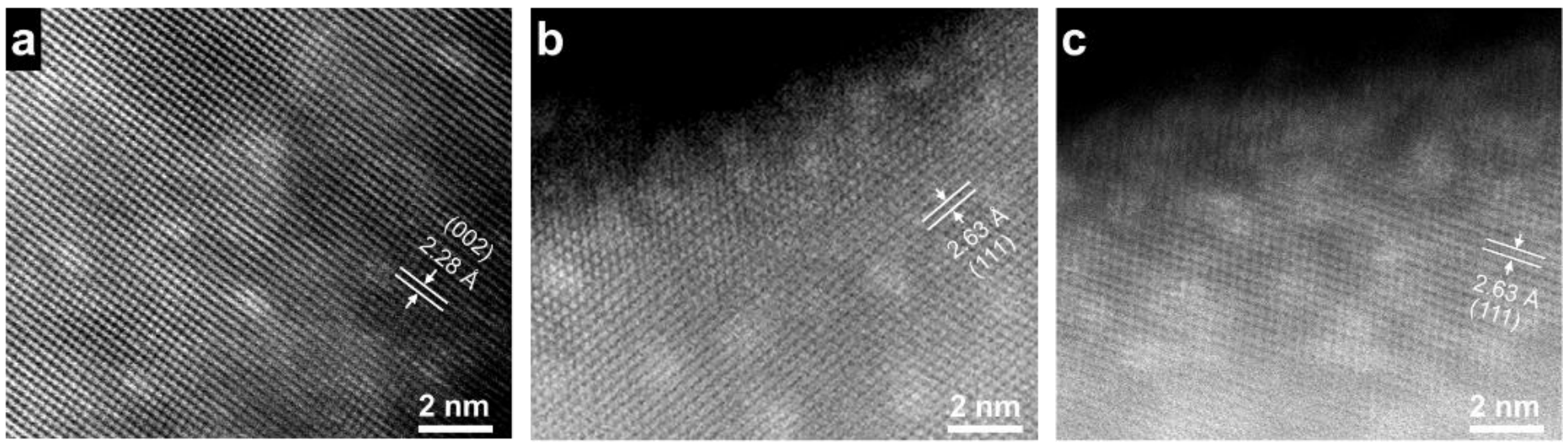

Figure 29. HAADF-STEM characterization on PSC ZnO-c samples with (a)1.0 wt%, (b)2.0 wt%, and (c)3 wt% Ru loadings. Higher Ru loadings (2-3 wt%) lead to Ru nanoparticle formation, altering product distribution toward CH_3OH and HCHO. Ru clusters become clearly visible at the 1.0 wt% loading. With the Ru loading further increased to 2.0 wt% and 3.0 wt%, the clusters grow distinctly larger and begin to exhibit structural features characteristic of nanoparticles, demonstrating a pronounced tendency toward nanoparticle formation with increased loading.

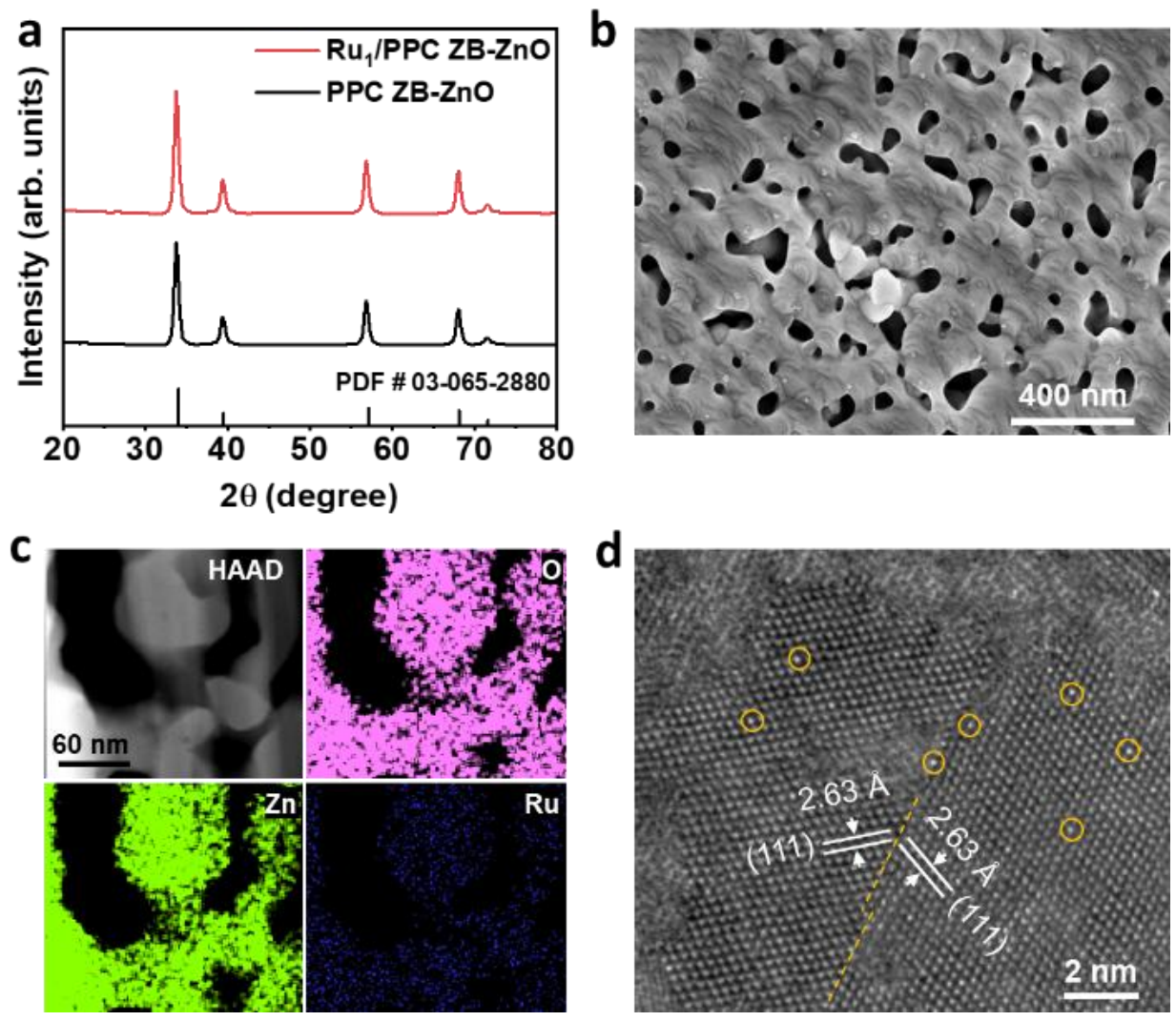

Figure S30. Structural and Morphological Characterization of Ru_1/PPC ZnO-c. (a) XRD patterns of Ru_1/PPC ZnO-c and PPC ZnO-c, where diffraction peaks are indexed to PDF card #03-065-2880, confirming the cubic phase of the as prepared material. (b) SEM image of Ru_1/PPC ZnO-c, exhibiting a porous architecture with uniform pore distribution. (c) Elemental mapping of Ru_1/PPC ZnO-c, illustrating the homogeneous dispersion of Ru, Zn, and O species throughout the porous matrix. (d) HAADF STEM Image of Ru_1/PPC ZnO-c.

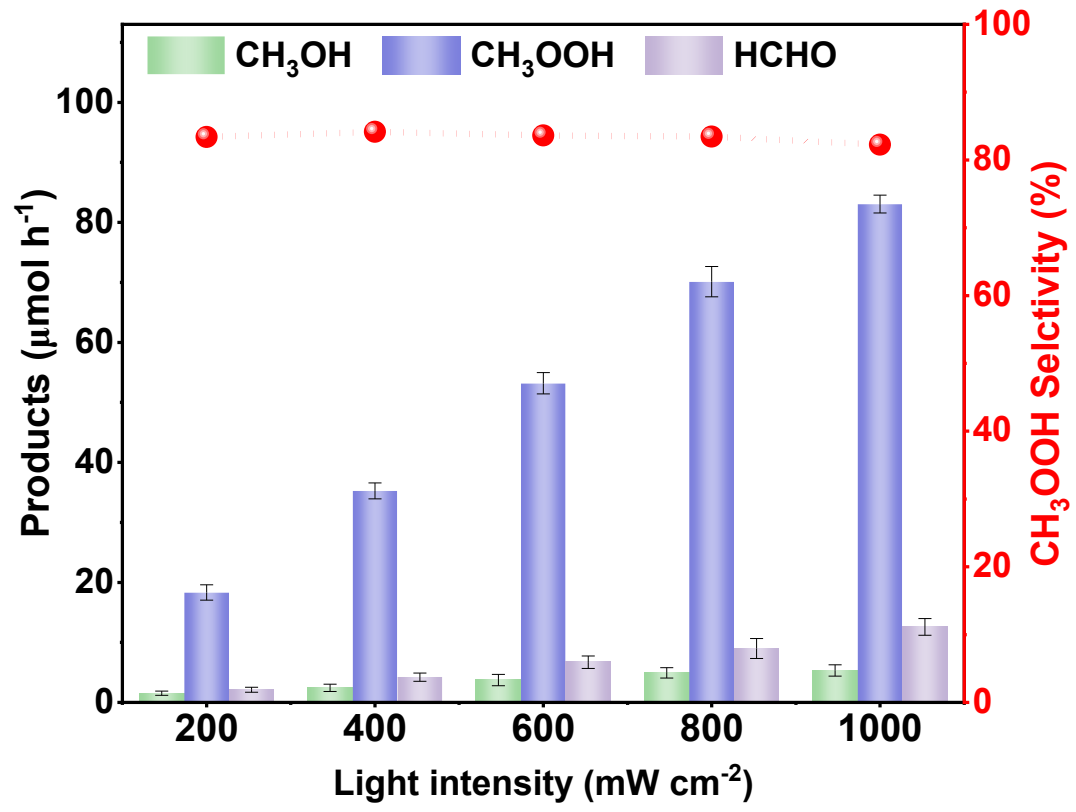

Figure S31. Photocatalytic activity of Ru_1/PSC ZB-ZnO with different light intensity. The error bars represent standard deviations obtained from three parallel experiments.

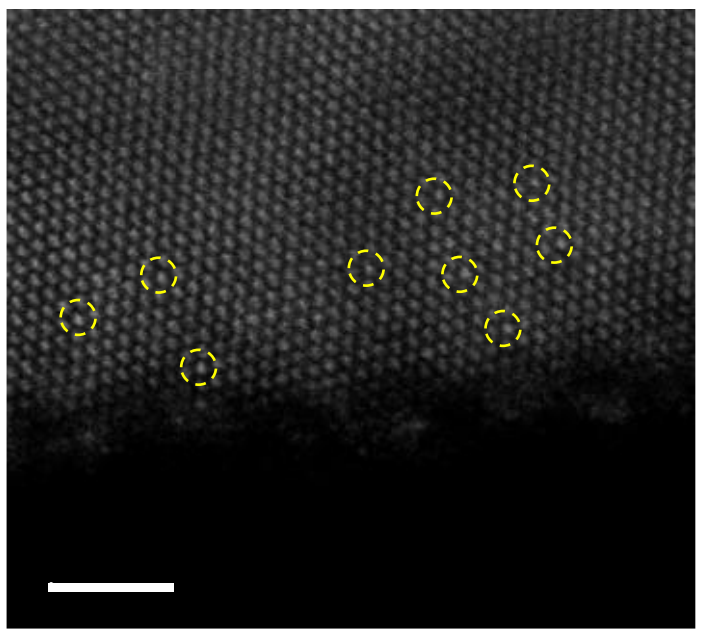

Figure S32. Post-reaction atomic-scale characterization of Ru_1/ZB-ZnO. HAADF-STEM confirm persistent single-atom dispersion of Ru (yellow circles) on ZnO after catalytic reaction. No Ru clusters/nanoparticles observed.

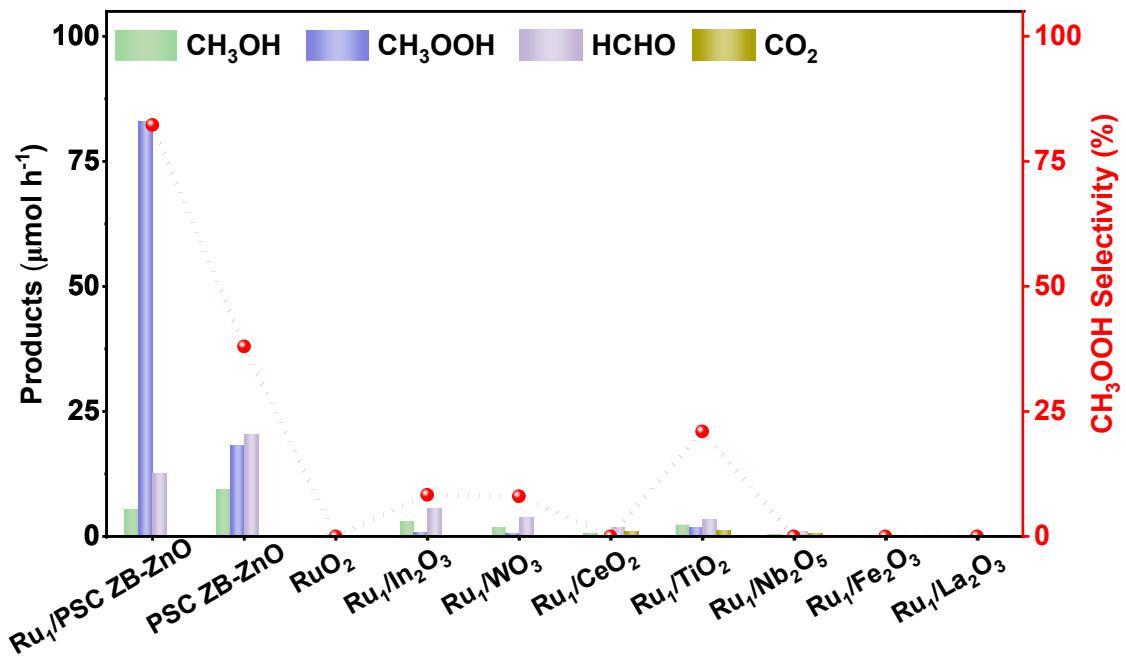

Figure S33. Performance evaluation of Ru_1/PSC ZB-ZnO and other Ru-based catalysts in the photocatalytic methane oxidation reaction.

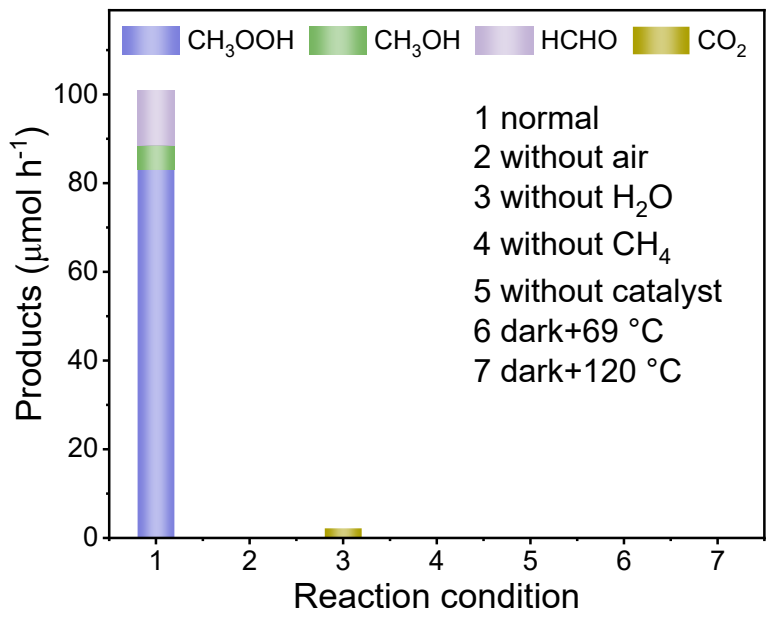

Figure 34. Control experiments screening the necessity of key reaction components. Production rates obtained over Ru_1/PSC ZB-ZnO under different conditions. Reaction conditions: 45 mL min^{-1} CH_4 and 5 mL min^{-1} air (25 vol.% O_2/N_2), RH = 20%, 50 °C, 300 W Xe lamp with AM 1.5G filter, 1000 mW cm^{-2}. The results demonstrate that significant product formation occurs exclusively under light irradiation with all reaction components present. The negligible activity observed in the dark (even with system temperature rise to 120 °C), under visible light, in the absence of methane, or without catalyst, systematically rules out thermal effects. O_2 and H_2O is essential for initiating methane oxidation.

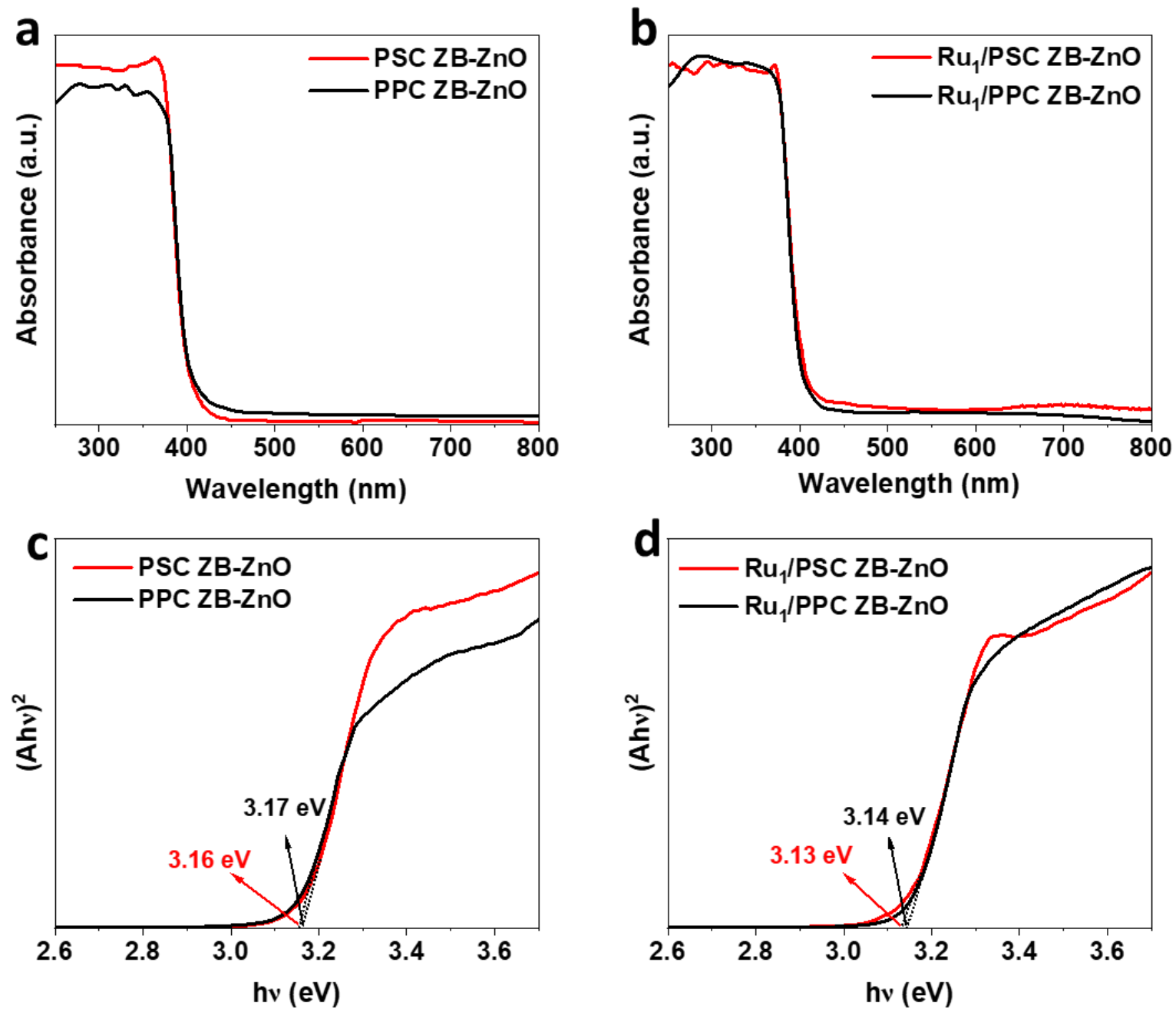

Figure S35. Photophysical properties of ZnO based catalysts with/without Ru modification. (a) UV−visible diffuse reflectance spectra of PSC ZB-ZnO, PPC ZB-ZnO, and PPC ZB-ZnO. (b) UV−visible diffuse reflectance spectra of Ru_1/PPC ZB-ZnO. (c-d) Transformed Kubelka-Munk function versus photon energy. The determined bandgaps are E_g = 3.16 eV (PSC ZB-ZnO), 3.17 eV (PPC ZB-ZnO), 3.13 eV (Ru_1/PSC ZB-ZnO), and 3.14 eV (Ru_1/PPC ZB-ZnO), highlighting the influence of Ru incorporation and support (PPC vs PSC) on the electronic structure of ZnO.

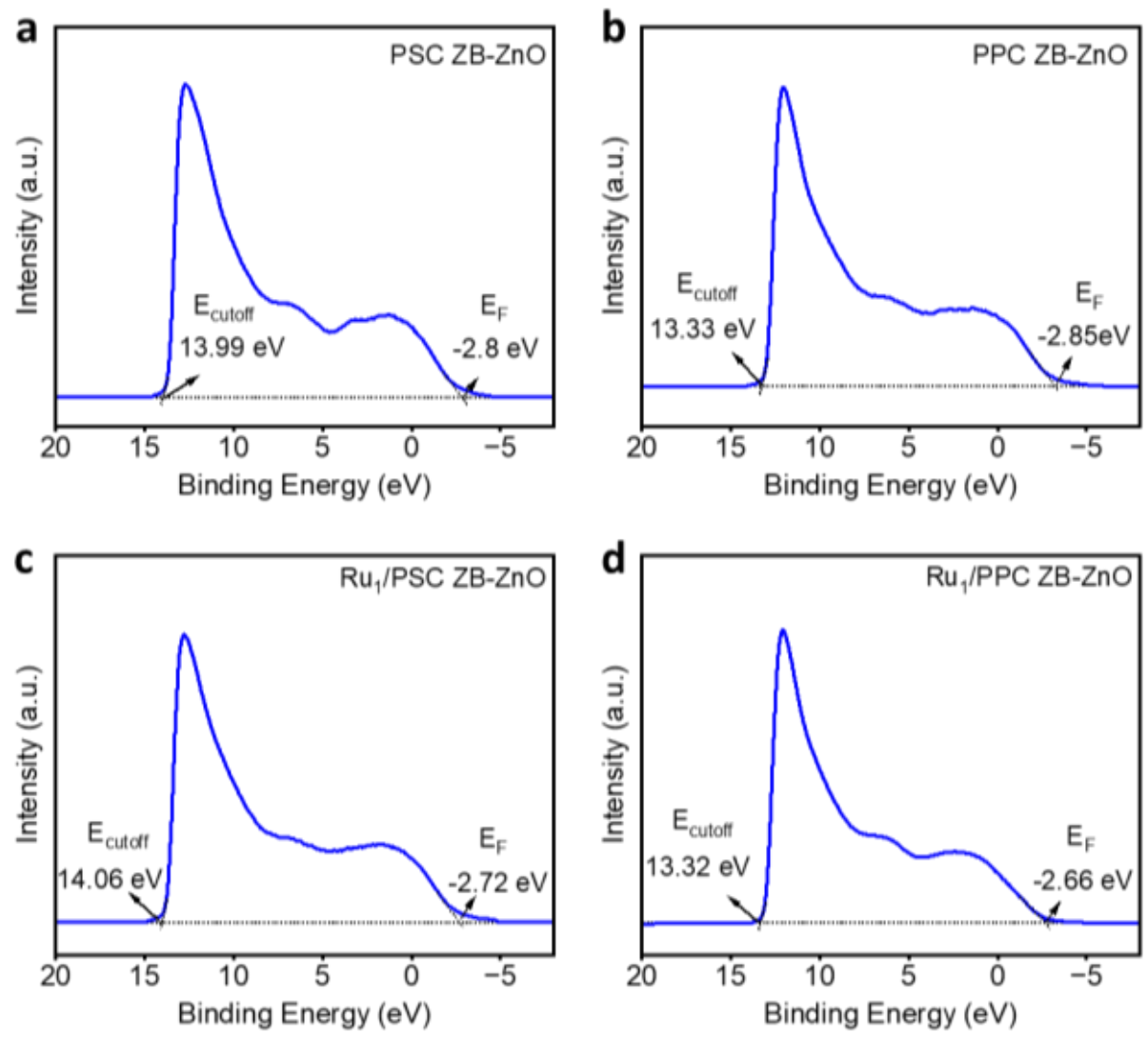

Figure S36. The UPS spectra of (a) PSC ZB-ZnO, (b) PPC ZB-ZnO, (c) Ru_1/PSC ZB-ZnO, and (d) Ru_1/PPC ZB-ZnO, respectively. Thie electron work function of the analyzer was using the formula: $\Phi = h\nu - (E_{cutoff} - E_F)$ ($h\nu$ =21.22 eV).

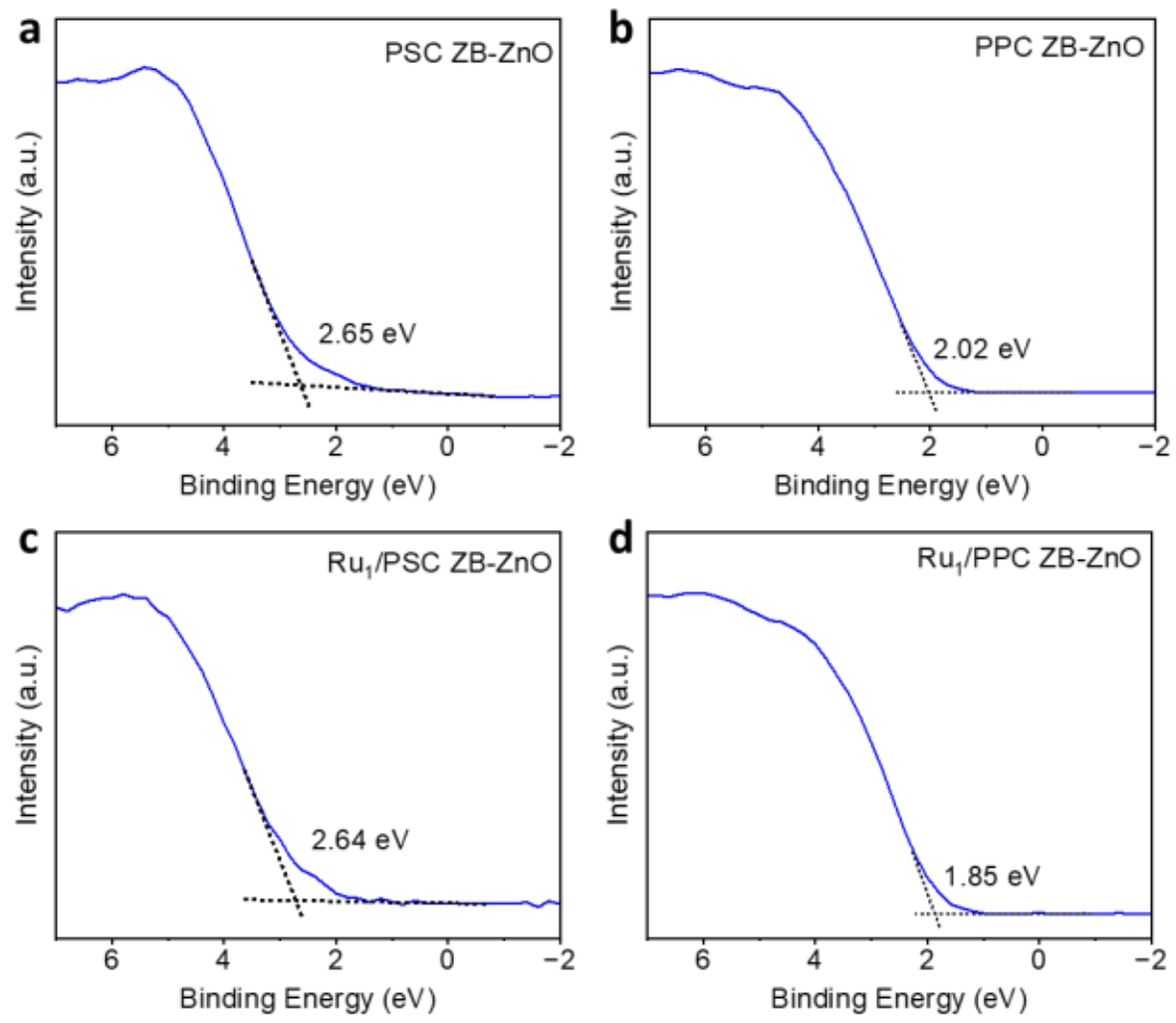

Figure S37. VB-XPS spectrum of (a) PSC ZB-ZnO, (b) PPC ZB-ZnO, (c) Ru_1/PSC ZB-ZnO, and (d) Ru_1/PPC ZB-ZnO, respectively. E_{VB} vs. NHE (V) was calculated using the formula $E_{VB} = \Phi + VB - 4.44$.

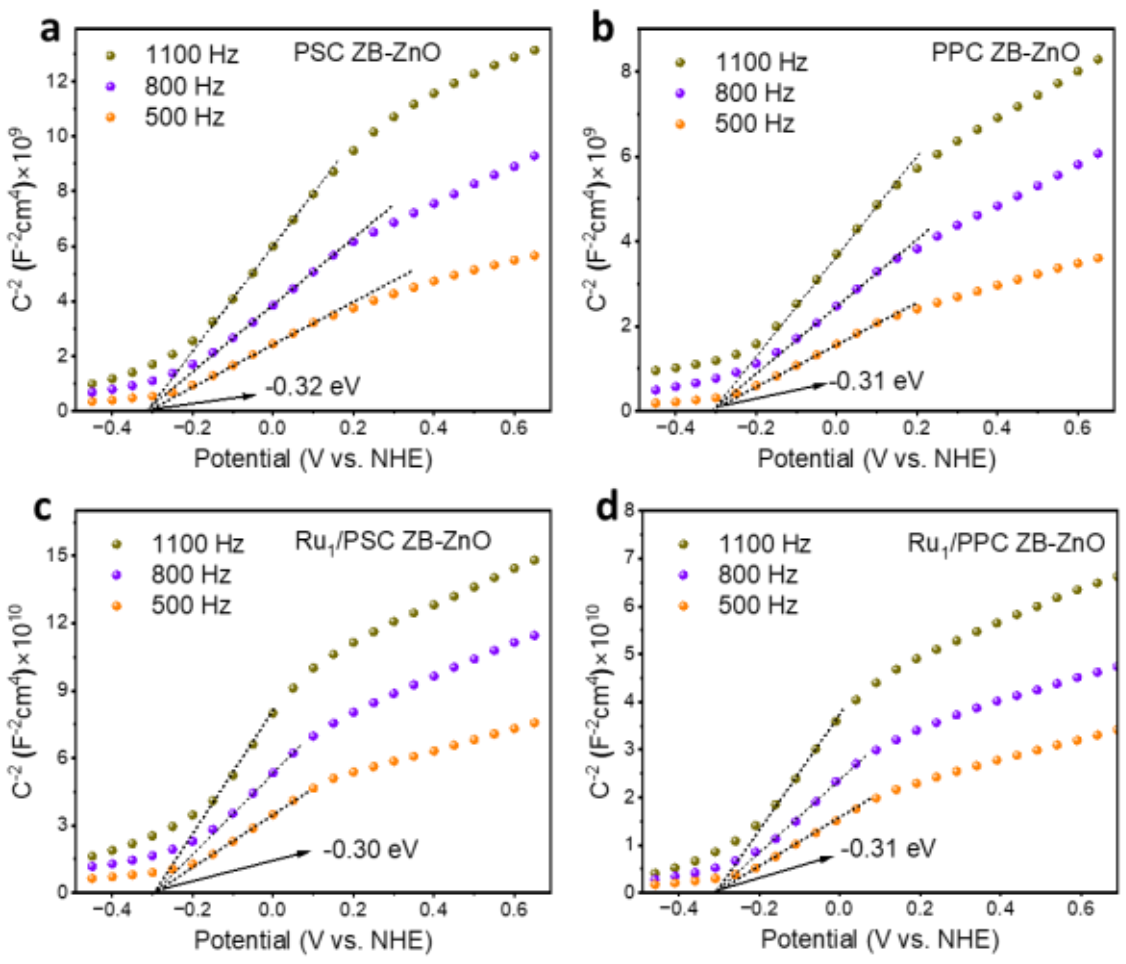

Figure S38. Mott Schottky plots of (a) PSC ZB-ZnO, (b) PPC ZB-ZnO, (c) Ru_1/PSC ZB-ZnO, and (d) Ru_1/PPC ZB-ZnO, respectively. All the samples showed the positive slope of the Mott-Schottky plot, implying n type semiconductors. The flat band potential (E_{fb}) were determined by extending the linear portion of the MS plots. In general, the conduction band potential (E_{CB}) was considered to be ~0.2 eV negative than the E_{fb}.

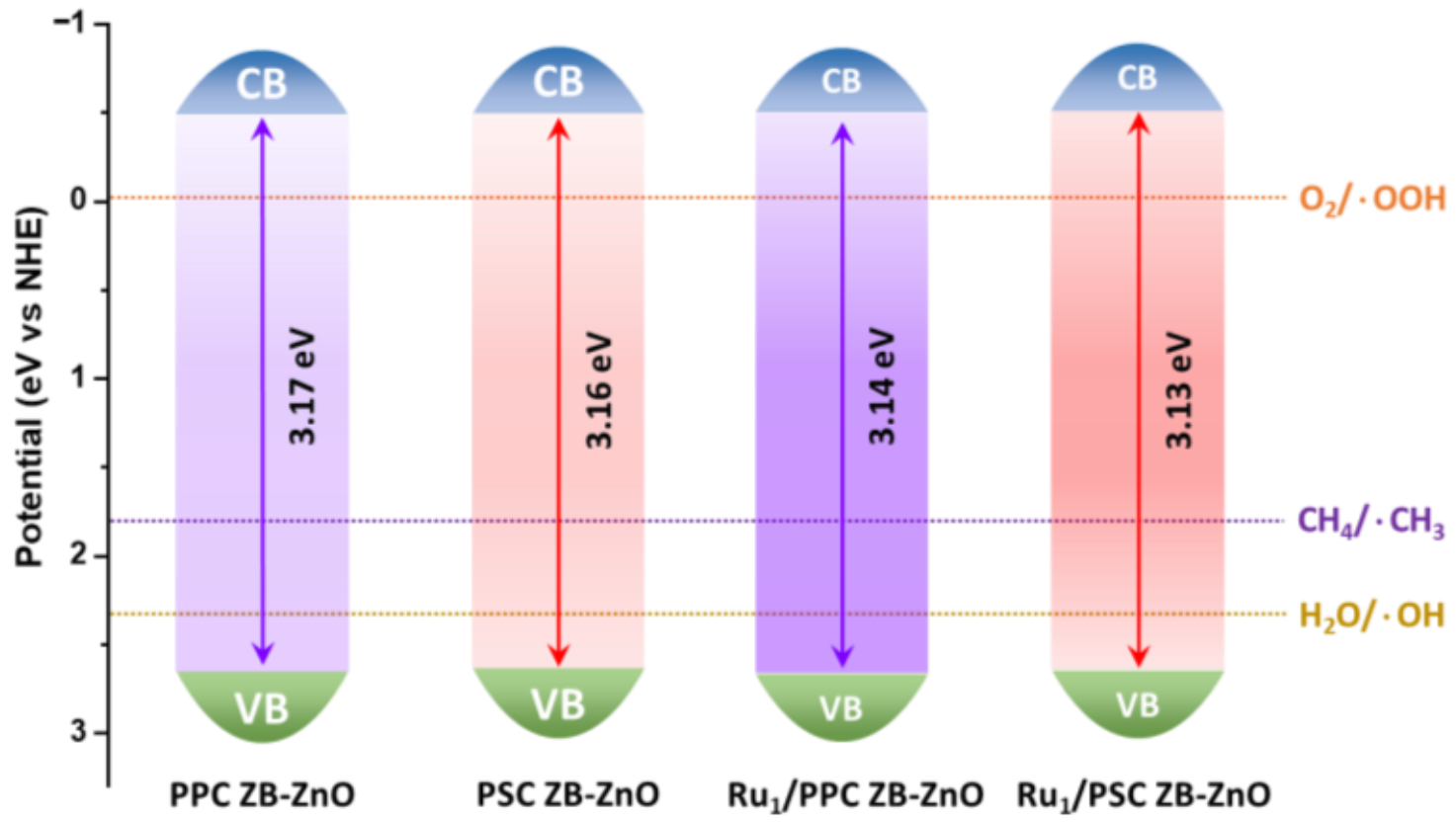

Figure S39. Schematic illustration of the band structures of PPC ZB-ZnO, PSC ZB-ZnO, Ru_1/PPC ZB-ZnO, and Ru_1/PSC ZB-ZnO. determined by VB-XPS valence spectra, UPS spectrums and Mott−Schottky plots.

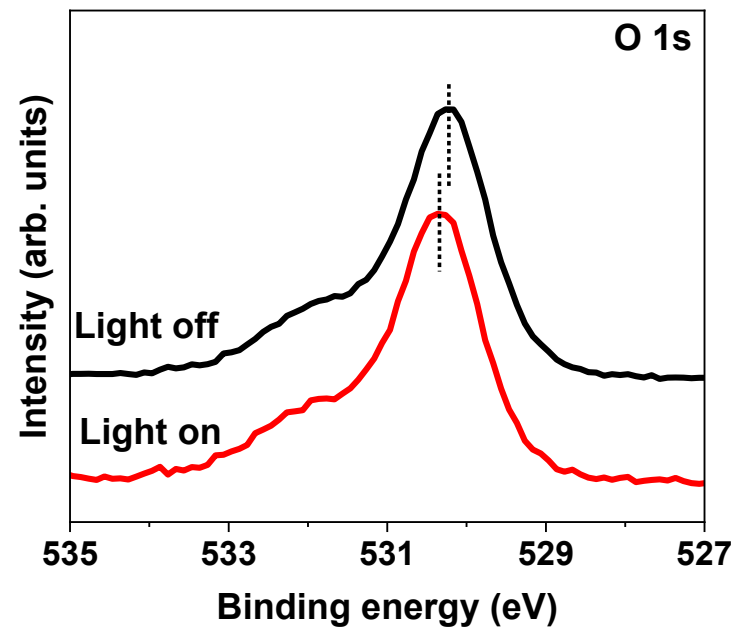

Figure S40. In situ O 1*s* XPS spectra of Ru_1/ZB-ZnO in dark and under light irradiation.

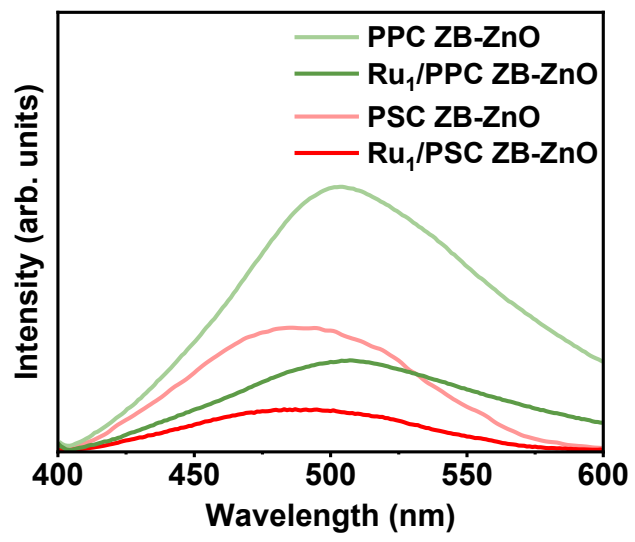

Figure S41. Steady-state PL characterizations of ZnO samples, with and without Ru single-atom modification. Ru modification enhances the charge separation efficiency of ZnO-based photocatalysts, while the single-crystalline structure synergistically suppresses the recombination of photo-generated carriers.

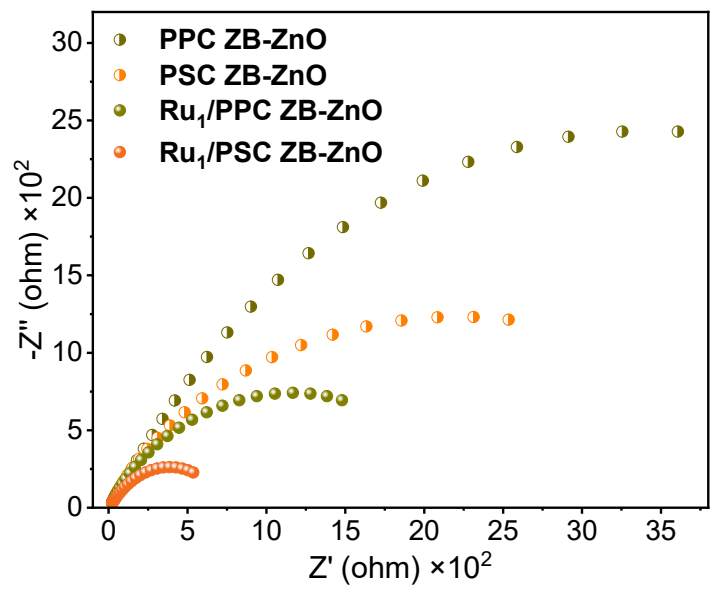

Figure S42. Electrochemical impedance spectroscopy (EIS) of single-crystal and polycrystalline ZnO with and without Ru single-atom modification. The analysis demonstrates that that single-crystalline ZnO (PSC ZB-ZnO) possesses a substantially lower charge-transfer resistance than its polycrystalline counterparts (PPS ZB-ZnO). Moreover, this advantage is further accentuated upon Ru modification. Specifically, the Ru-modified systems display a notably smaller semicircle radius in the Nyquist plots, evidencing a significant further reduction in charge-transfer resistance. This underscores a synergistic interplay, where the single-crystal morphology provides an efficient conductive pathway, and the atomic-level Ru incorporation actively enhances the interfacial charge-transfer kinetics.

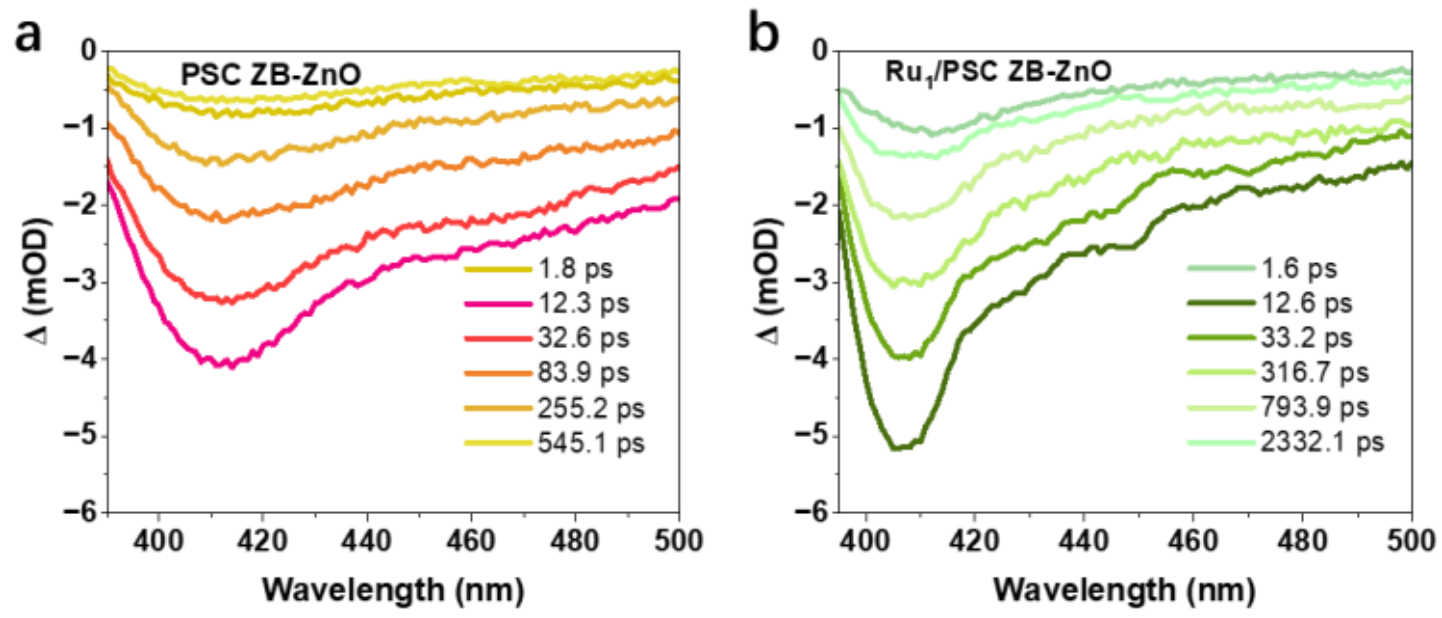

Figure S43. Femtosecond transient absorption (fs-TA) spectra of (a) PSC ZB-ZnO and (b) Ru_1/PSC ZB-ZnO (pump beam: 360 nm).

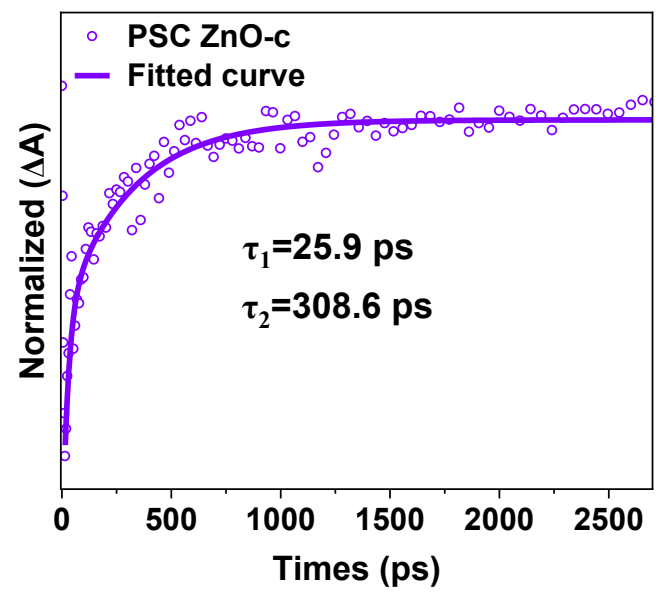

Figure S44. The normalized TAS decay kinetics of PSC ZB-ZnO probed at 412 nm excited by pump 360 nm.

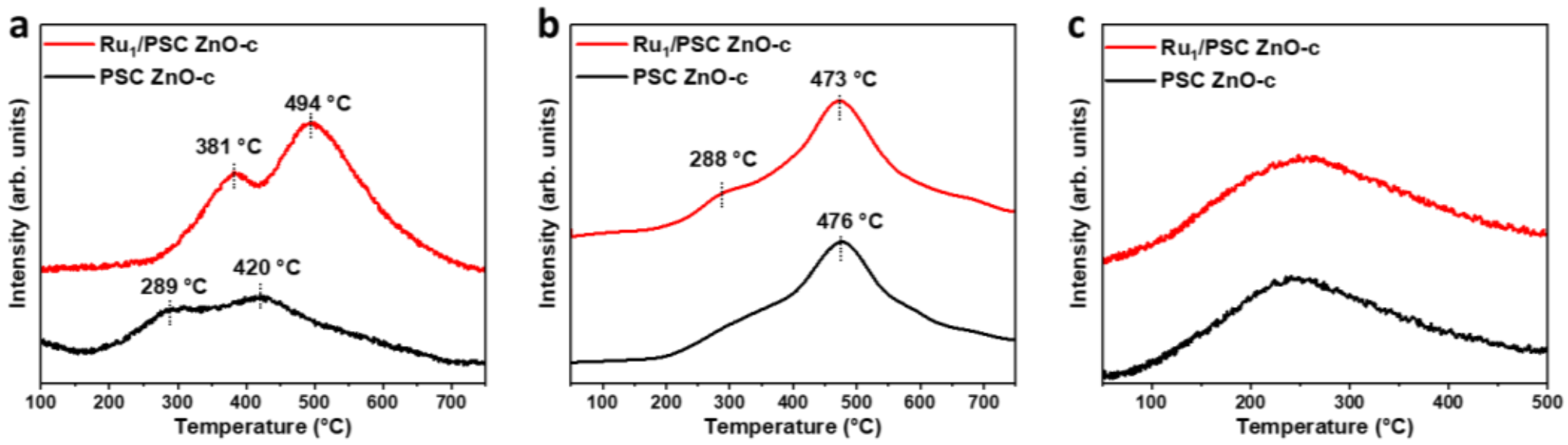

Figure S45. Temperature-programmed desorption (TPD) analyses. **a-c,** O_2-TPD, CH_4-TPD, H_2O-TPD spectra of Ru_1/PSC ZB-ZnO and PSC ZB-ZnO.

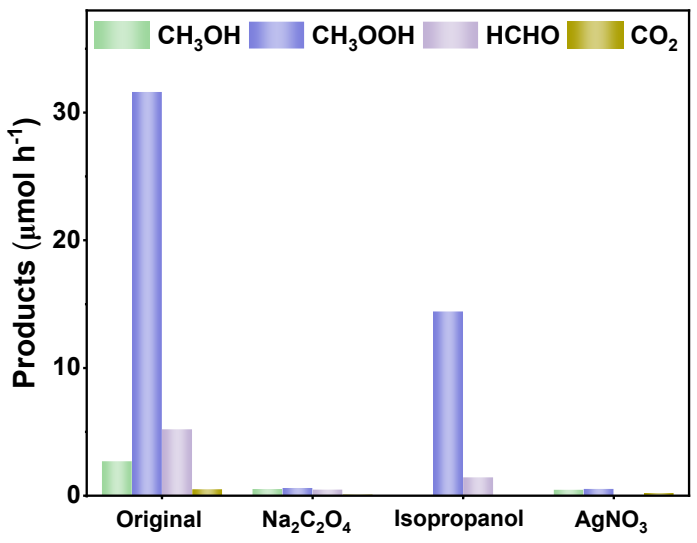

Figure S46. Active species trapping experiments for photocatalytic CH_4 oxidation. Influence of the scavengers of the photocatalytic CH_4 oxidation over PSC Ru_1/ZB-ZnO. $Na_2C_2O_4$ as h^+ capture agent, isopropanol as ·OH capture agent, $AgNO_3$ as e^- capture agent. Batch reactor, 0.1 MPa air (25 vol.% O_2 and N_2), 1.9 MPa CH_4, 50 mL H_2O, 1 h reaction time.

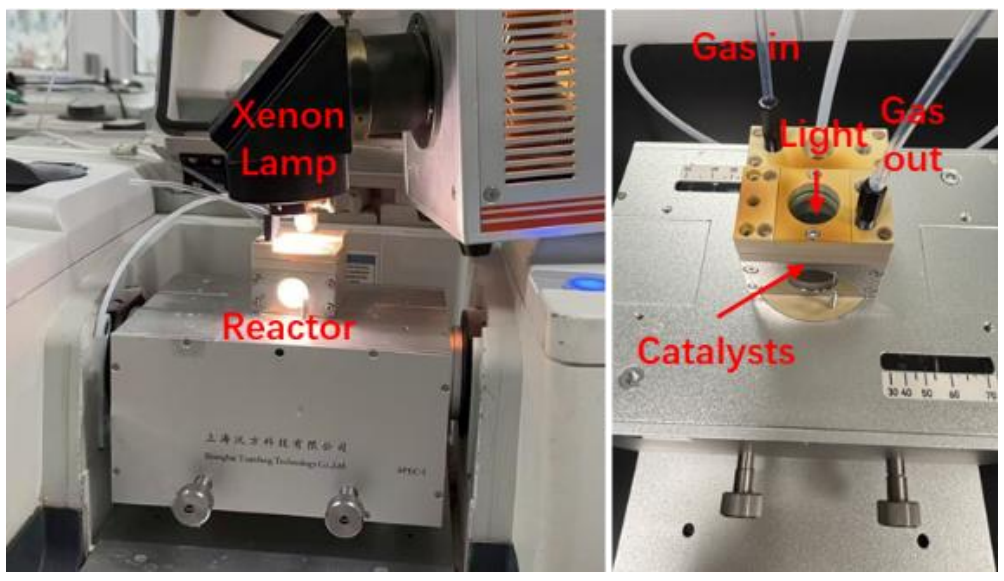

Figure S47. Schematic illustration of the in-situ infrared reaction setup.

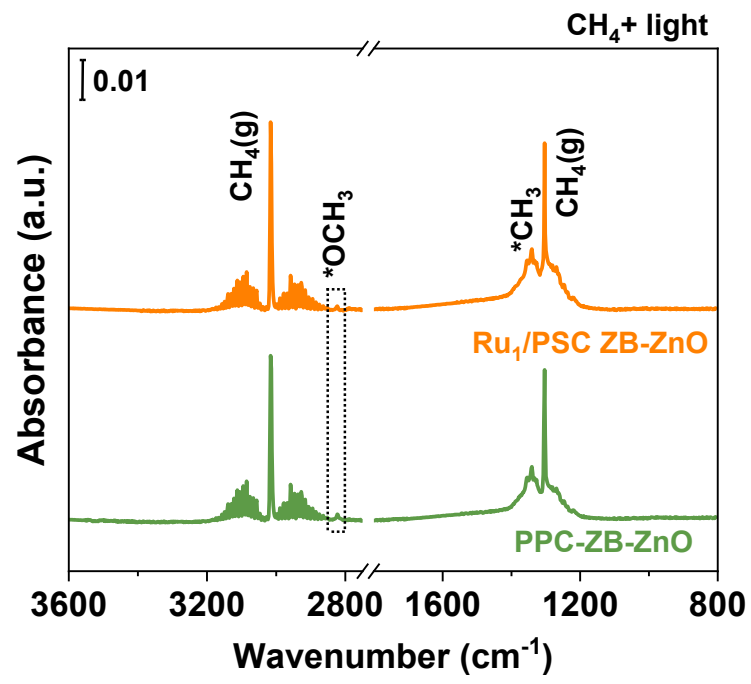

Figure S48. In situ diffuse reflectance infrared Fourier transform spectroscopy (DRIFTS) for CH_4 photooxidation over Ru_1/PSC ZB-ZnO and PSC ZB-ZnO under light and CH_4.

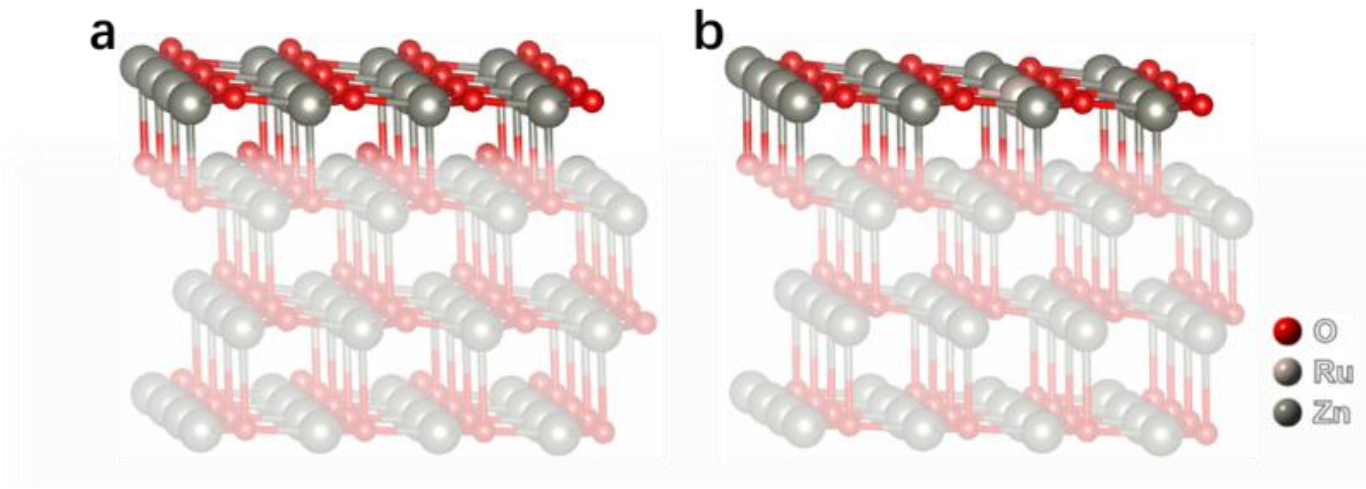

Figure S49. Calculation model of (a) ZnO(222) and (b) Ru_1/ZnO(222) for ZB-ZnO and Ru_1/ZB-ZnO, respectively. Pink, gray and red spheres in the model represent Ru, Zn and O atoms, respectively.

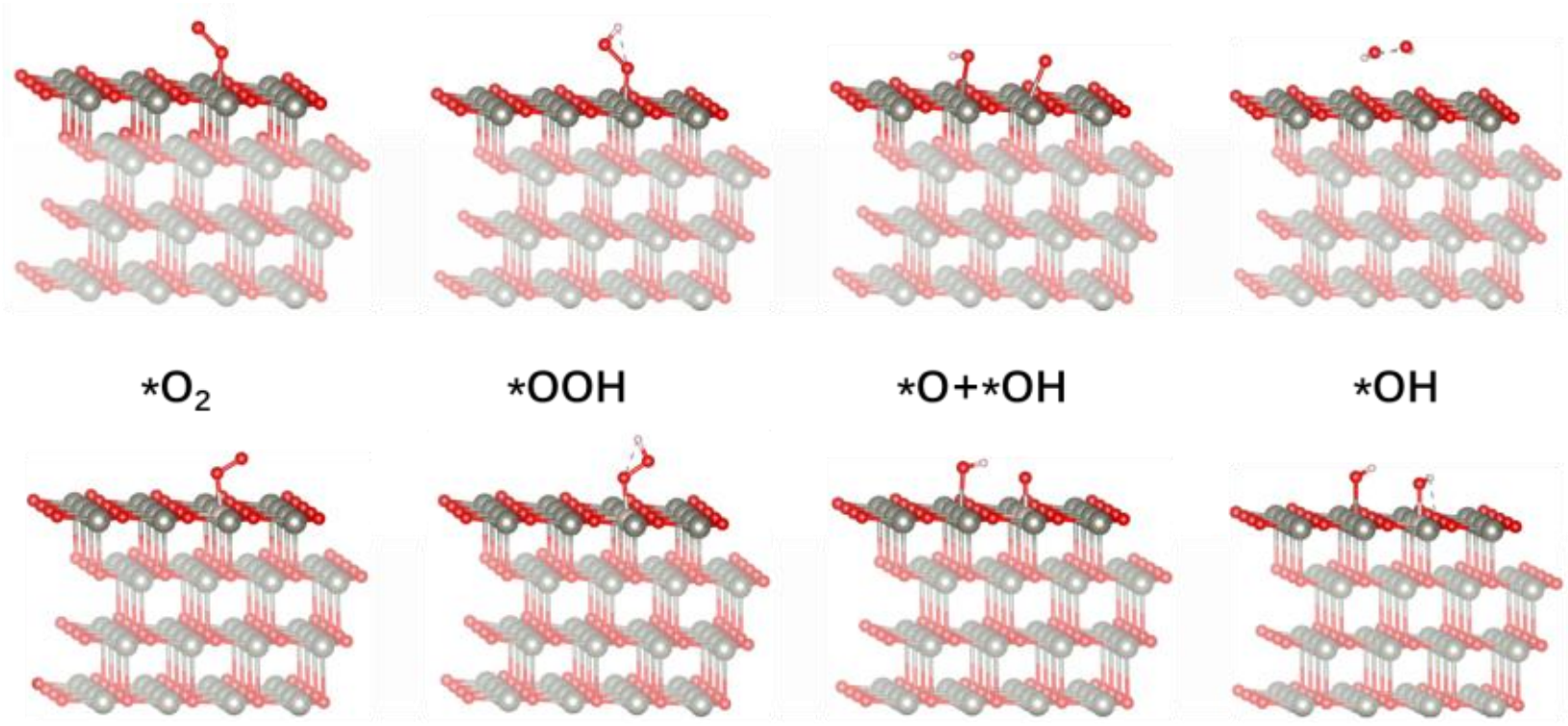

Figure S50. Scheme of O_2 reduction intermediates on ZB-ZnO(222) (top) and Ru_1/ZB-ZnO(222) (bottom). Pink, gray, red, and white spheres in the model represent Ru, Zn, O, and H atoms, respectively.

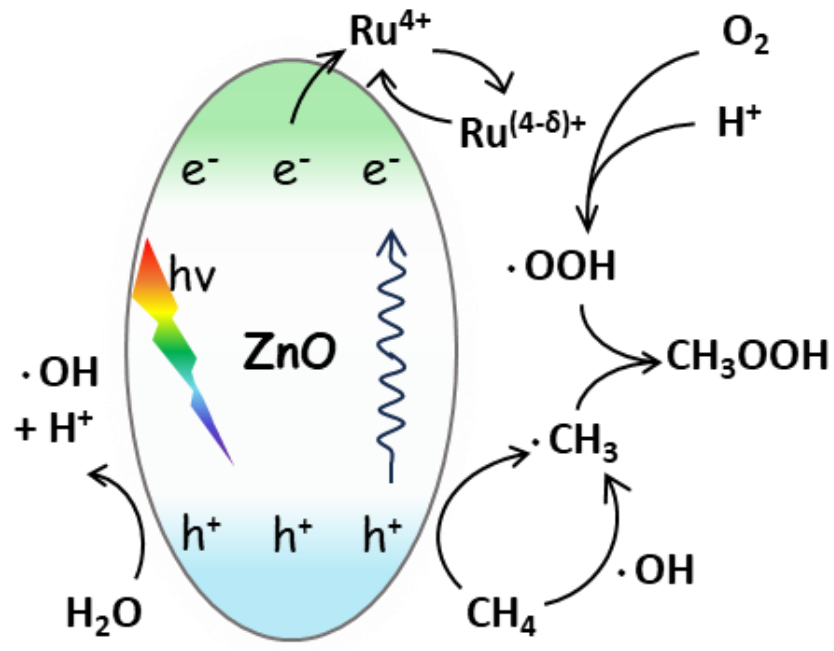

$$H_2O + h^+ \rightarrow \cdot OH + H^+$$

$$O_2 + e^- + H^+ \rightarrow \cdot OOH$$

$$CH_4 + h^+ \rightarrow \cdot CH_3 + H^+$$

$$\text{or } CH_4 + \cdot OH \rightarrow \cdot CH_3 + H_2O$$

$$\cdot CH_3 + \cdot OOH \rightarrow CH_3OOH$$

Figure S51. Schematic illustration of Photocatalytic CH_4 Oxidation on Ru_1/ZB-ZnO.

Table S1. Summary of the results obtained from rietveld XRD studies: lattice parameters (a, b, c), unit cell volume (V), and R_p factor.

Sample	a (Å)	b (Å)	c (Å)	V (Å^3)	R_p (%)	Phase
#1	4.5847	4.5847	4.5847	96.4	5.66	Cubic
#2	4.5831	4.5847	4.5847	96.3	8.89	Cubic
#3	4.5881	4.5881	4.5881	96.6	7.71	Cubic
#4	4.5820	4.5820	4.5820	96.2	7.73	Cubic
#5	4.5873	4.5873	4.5873	96.5	7.60	Cubic
#6	4.5782	4.5782	4.5782	95.9	9.60	Cubic

Table S2. Curvefit Parameters[a] for Ru K-edge EXAFS for various

Sample	Path	N	S_0^2	R / Å	d^b / Å	ΔE_0 / eV	σ^2 / Å
Ru_1/ZnO	Ru-O	4.4(5)	1	2.01(1)	1.982	0±1	0.006(2)

[a] S_0^2 was fixed as 1. ΔE_0 was refined as a global fit parameter, returning a value of (2 ±1) eV. Data ranges: $2.5 \leq k \leq 12$ Å^{-1}, $1.0 \leq R \leq 2.5$ Å. The number of variable parameters is 4, out of a total of 9.1 independent data points. R factor for this fit is 1.2%. [b] The distances for Ru-O is from the crystal structure of RuO_2.

Table S3. The amount of formed liquid oxygenates, the light intensity, and calculated AQE at different wavelengths (an example of measurement).

Wavelength (nm)	Light Intensity (W/cm^2)	CH_3OOH (μmol)	CH_3OH (μmol)	HCHO (μmol)	N(photons) (mol)	AQE (%)
365	0.018	55.63	4.62	5.06	3.73×10^{20}	15.3
380	0.021	58.84	3.77	5.59	4.54×10^{20}	13.0
420	0.026	2.10	0.15	0.11	6.21×10^{20}	0.3

Take AQE at 365 nm of Ru_1/ZB-ZnO as an example, AQE calculated from Equation:

$$AQE = \frac{N_e}{N(\text{photons})}\times100\% = \frac{N_A\times(N(CH_3OOH) + 3\times N(CH_3OH) + 5\times N(HCHO))}{N(\text{photons})}\times100\%$$

$$= \frac{6.02\times10^{23}\times(55.63 + 3\times4.62 + 5\times5.06)\times10^{-6}}{3.73\times10^{20}}\times100\%$$

$$=15.3\%$$

Table S4. Physicochemical properties of ZnO-based catalysts.

Catalysts	BET surface area (m^2/g)	Ru loading (wt%)
PSC ZB-ZnO	16.1	0
PPC ZB-ZnO	16.6	0
Ru_1/PSC ZB-ZnO	15.6	0.30
Ru_1/PPC ZB-ZnO	15.7	0.32

Table S5: Performance comparison of photocatalysis. Representative works on the selective photocatalytic conversion of CH_4 to CH_3OOH.

Catalysts	Reactor type	Reactants	Light	CH_3OOH (μmol/h)	Selectivity (%)	Ref.
Ru_1/PSCZB-ZnO	flow	45 mL min^{-1} CH_4 and 5 mL min^{-1} air (25 vol.% O_2/N_2), RH = 20%; Cat.: 100 mg, 69 °C	1000 mW cm^{-2}	83.0	82.3	**This work**
b-TiO_2	batch	P_{CH4} = 0.1 MPa; 20 mL 50 mM H_2O_2, 1 h; Cat.: 2 mg	300 mW cm^{-2}	1.5	100	1
Ru_1O_x/ZnO	batch	P_{CH4} = 2 MPa; P_{O2} = 0.1 MPa; 10 mL of H_2O, 1 h; Cat.: 10 mg, 25 °C	150 mW cm^{-2}	3.2	90.9	2
0.5Ru-ZnO	batch	P_{CH4} = 2 MPa; P_{O2} = 0.1 MPa; 180 mL of H_2O, 1h; Cat.: 10 mg, 25 °C	365 nm LED (75 mW cm^{-2})	100.2	86.1	3
$Pd_{0.5}$-def-WO_3	batch	P_{CH4} = 1.9 MPa; P_{O2} = 0.1 MPa; 100 mL of H_2O, 2h; Cat.: 20 mg, 25 °C	100 mW cm^{-2}	92.9	80.3	4
Pd-def-In_2O_3	batch	P_{CH4} = 1.9 MPa; P_{O2} = 0.1 MPa; 50 mL of H_2O, 3 h; Cat.: 20 mg, 25 °C	LED lamp (421 nm)	36	60	5
ZnO(Zni)-350	batch	P_{CH4} = 1.9 MPa; P_{O2} = 0.2 MPa; 180 mL H_2O, 2 h; Cat.: 10 mg, 30 °C	550 mW cm^{-2}	18.6	60.4	6
$Au_{0.2}Cu_{0.15}$-ZnO	batch	P_{CH4} = 1.9 MPa; P_{O2} = 0.1 MPa; 100 mL of H_2O, 2h; Cat.: 20 mg, 25 °C	150 mW cm^{-2}	124.7	56.9	7
Au/ZnO	batch	P_{CH4} = 2.9 MPa r; P_{O2} = 0.1 MPa; 30 mL of H_2O; 2 h; Cat.: 10 mg, 25 °C	320 nm - 780 nm; 300 mW·cm^{-2}	59.4	54	8
$Au_{0.15}$/ZnO	batch	P_{CH4} = 1.5 MPa; P_{O2} = 0.5 MPa;100 mL of H_2O; 2 h; Cat.: 10 mg, 30 °C	100 mW cm^{-2}	5.4	52.8	9
Cu-W-TiO_2	batch	P_{CH4} = 2 MPa; P_{O2} = 0.2 MPa; 80 mL of H_2O, 2 h; Cat.: 10 mg, 25 °C	350 - 760 nm, 200 mW cm^{-2})	77.5	47	10
0.1 wt % Au/ZnO	batch	P_{CH4} = 2 MPa; P_{O2} = 0.1 MPa;100 mL of H_2O; 2 h; Cat.: 10 mg, 25 °C	300-500 nm, 100 mW cm^{-2}	62.7	47	11
1%Cu/ZnO	batch	P_{CH4} = 1.9 MPa; P_{O2} = 0.1 MPa; 80 mL of H_2O; 2 h; Cat.: 5 mg, 30 °C	16 A	40.5	42	12
Au/CoO_x/TiO_2	batch	P_{CH4} = 2 MPa; P_{O2} = 0.1 MPa; 180 mL of H_2O; 2 h; Cat.: 10 mg, 25 °C	450 mW cm^{-2}	10.4	39	13
Au_1Pd_9-ZnO	batch	P_{CH4} = 1.9 MPa; P_{O2} = 0.1 MPa; 75 mL of H_2O; 1h; Cat.: 10 mg, 25 °C	350 mW cm^2	40	34.8	14
Au-Pd/TiO_2	batch	P_{CH4} = 3 MPa; P_{O2} = 1 MPa; 30 mL of H_2O, 1h; Cat.: 5 mg, 42 °C	250 mW cm^{-2}	31	31	15

Table S6: Performance comparison of photocatalysis. Representative works on the selective photocatalytic oxidation of CH_4 to C_1 oxygenates at high density light.

Catalysts	Reactor type	Reactants	Light	Oxygenates (μmol/h)	Selectivity (%)	AQE (%)	Ref.
Ru_1/PSCZB-ZnO	flow	45 mL min^{-1} CH_4 and 5 mL min^{-1} air (25 vol.% O_2/N_2), RH = 20%; Cat.: 100 mg	350-1000 nm; 1000 mW cm^{-2}	100.9	99.9	15.3 (365 nm)	**This work**
FeO_x/ZnO	batch	P_{CH4} = 3 MPa; 5 mM H_2O_2; 90 mL of H_2O, 3 h; Cat.: 5 mg	full spectrum, 1500 mW cm^{-2}	20.7	99.9	/	16
Ag_1-ZnO	flow	45 mL min^{-1} CH_4 and 5 mL min^{-1} air (25 vol.% O_2/N_2), RH = 20%; Cat.: 4 mg	200 - 1200 nm; 1000 mW cm^{-2}	124	73.9	20.19 (365 nm)	17
1wt.%Pd-ZnO	flow	50 mL min^{-1} CH_4 and 10 mL min^{-1} air (25 vol.% O_2/N_2); Cat.: 0.5 mg	full spectrum, 600 mW cm^{-2}	3.3	100	12.7 (350 nm)	18
0.5Ru-ZnO	batch	P_{CH4} = 2 MPa; P_{O2} = 0.1 MPa; 180 mL of H_2O, 1h; Cat.: 10 mg	365 nm LED (75 mW cm^{-2})	476.2	95.4	/	3
ZnO(Zni)-350	batch	P_{CH4} = 1.9 MPa; P_{O2} = 0.2; 180 mL of H_2O, 2 h; Cat.: 10 mg	550 mW cm^{-2}	28.9	98.6	5.7 (365 nm)	6
Au/CoO_x/TiO_2	batch	P_{CH4} = 2 MPa; P_{O2} = 0.1; 180 mL of H_2O, 2 h; Cat.: 10 mg	450 mW cm^{-2}	25.4	95	1.2 (368 nm)	13
Ni-NC/TiO_2	batch	P_{CH4} = 2 MPa; P_{O2} = 0.1 MPa; 100 mL of H_2O, 4h; Cat.: 10 mg	300-500 nm, 450 mW cm^{-2})	50.2	93	1.9 (360 nm)	19
3.2%Ag/TiO_2 {001}	batch	P_{CH4} = 2 MPa; P_{O2} = 0.1 MPa; 100 mL of H_2O, 2h; Cat.: 10 mg	300-500 nm, 450 mW cm^{-2}	54.1	90	/	20
Au_1Pd_9-ZnO	batch	P_{CH4} = 1.9 MPa; P_{O2} = 0.1 MPa; 75 mL of H_2O, 1h; Cat.: 10 mg	350 mW cm^2	101.3	86.75	16.5 (380 nm)	14
Au/ZnO	batch	P_{CH4} = 29 bar; P_{O2} = 1 bar; 30 mL of H_2O; 2 h; Cat.: 10 mg	320-780 nm; 300 mW·cm^{-2}	106.2	96.6	6.4 (365 nm)	8
Au_1/In_2O_3	batch	P_{CH4} = 2 MPa; P_{O2} = 1 MPa; 180 mL of H_2O, 7h; Cat.: 10 mg	300-1100 nm, 250 mW cm^{-2}	18.8	95	/	21

Table S7. Comparison of catalytic performance. Representative works on photocatalytic CH_4 conversion to C_1 oxygenates in flow reactor.

Catalysts	Reactants	T (°C)	Light	Oxygenates (μmol/h)	Selectivity (%)	Ref.
Ru_1/PSC ZB-ZnO	45 mL min^{-1} CH_4 and 5 mL min^{-1} air (25 vol.% O_2/N_2), RH = 20%; Cat.: 100 mg	69	350 - 1000 nm; 1000 mW cm^{-2}	100.9	99.9	**This work**
Ag_1-ZnO	45 mL min^{-1} CH_4 and 5 mL min^{-1} air (25 vol.% O_2/N_2), RH = 20%; Cat.: 4 mg	228	200 - 1200 nm; 1000 mW cm^{-2}	124	73.9	17
PMOF-RuFe(OH)	O_2 + H_2O, ~1 bar, 3ml H_2O; Cat.: 10 mg	R.T.	400–780 nm	88.1	100	22
1wt.%Pd-ZnO	50 mL min^{-1} CH_4 and 10 mL min^{-1} air (25 vol.% O_2/N_2); Cat.: 0.5 mg	35.3	full spectrum, 600 mW cm^{-2}	3.3	100	18
90% A-TiO_2	Cat.: 10 mg; P_{CH4} = 20 bar; P_{O2} = 5 bar; 180 mL of H_2O, 3 h	25	300-1100 nm, 130 mW cm^{-2}	34	99.6	23
MoO_3 (5 wt.%)/SiO_2	Cat.: 25 mg; 7.4 mmol h^{-1} CH_4, (CH_4: O_2: He= 6: 2: 25);	220	high-pressure mercury vapour lamp (200 W)	6	100	24
MoO_3 (5 wt.%)/ZnO	Cat.: 250 mg; 7.5 mmol h^{-1} CH_4, (CH_4: O_2: He = 3:1:10);	220	high-pressure mercury vapour lamp (200 W)	4.9	79.2	25

Table S8. Fitted lifetime from normalized TAS decay kinetics of PSC ZB-ZnO and Ru_1/PSC ZB-ZnO.

Sample	A_1	τ_1 (ps)	A_2	τ_2 (ps)	τ_{avg} (ps)
PSC ZB-ZnO	1.2	25.9	0.56	308.6	265.5
Ru_1/PSC ZB-ZnO	0.48	17.9	0.48	809.4	792.3

Reference

[1] Sun Y. X., Bo C. L., Cheng Z. J., Zhang X. Y., Liu J. J., Piao L. Y. Tandem photo-oxidation of methane to methanol at room temperature and pressure over Pt/TiO_2. *Nano Res.*, **16**, 12942−12948 (2023).

[2] Gong H. T. *et al.* selective photocatalytic aerobic oxidation of methane to methyl hydroperoxide by ZnO-loaded single-atomic ruthenium oxide catalyst. *J. Chem. Soc. Chem. Comm.,* **147**, 9134-9146 (2025).

[3] Xu Y. X. *et al.* Efficient methane oxidation to formaldehyde via photon–phonon cascade catalysis. *Nat. Sustain.* **7**, 1171–1181 (2024).

[4] Wang K. R., Luo L., Wang C., Tang J. W. Photocatalytic methane activation by dual reaction sites co-modified WO_3. *Chinese J. Catal.* **46**, 103-112 (2023).

[5] Luo L. et al. Synergy of Pd atoms and oxygen vacancies on In_2O_3 for methane conversion under visible light. *Nat. Commun.* **13**, 2930 (2022).

[6] Xiao Z. et al. Interstitial zinc defects enriched ZnO tuning O_2 adsorption and conversion pathway for superior photocatalytic CH_4 oxygenation. *ACS Catal.* **14**, 9104-9114 (2024).

[7] Luo L. *et al.* Binary Au-Cu reaction sites decorated ZnO for selective methane oxidation to C_1 oxygenates with nearly 100% selectivity at room temperature. *J. Chem. Soc. Chem. Comm.,* **144**, 740-750 (2022).

[8] Zhou Q. *et al.* Selective photocatalytic oxidation of methane to methanol by constructing a rapid O_2 conversion pathway over Au-Pd/ZnO. *ACS Catal.* **14**, 955-964 (2024).

[9] Zhou W. C., *et al.* Highly selective aerobic oxidation of methane to methanol over gold decorated zinc oxide photocatalysis. *J. Mater. Chem. A* **8**, 13277-13284 (2020).

[10] Huang M. *et al.* Selective photocatalytic oxidation of methane to oxygenates over Cu-W-TiO_2 with significant carrier traps. *ACS Catal.* **12**, 9515-9525 (2022).

[11] Song H. *et al.* Direct and selective photocatalytic oxidation of CH_4 to oxygenates with O on cocatalysts/ZnO at room temperature in water. *J. Chem. Soc. Chem. Comm.,* **141**, 20507-20515 (2019).

[12] Cui Y. F. *et al.* Largely promoted C-H activation in methane with O_2 via d-Orbital hybridization induced by CuO_x supported on ZnO. *ACS Catal.* **15**, 1607-1615 (2025).

[13] Song H. *et al.* Selective photo-oxidation of methane to methanol with oxygen over dual-cocatalyst-modified titanium dioxide. *ACS Catal.* **10**, 14318-14326 (2020).

[14] Chen F. *et al.* Defective ZnO nanoplates supported AuPd nanoparticles for efficient photocatalytic methane oxidation to oxygenates. *Adv. Energy Mater.* **14**, (2024).

[15] Cai X. J., Fang S. Y., Hu Y. H.. Unprecedentedly high efficiency for photocatalytic conversion of methane to methanol over Au-Pd/TiO_2 - what is the role of each component in the system? *J. Mater. Chem. A* **9**, 10796-10802 (2021).

[16] Hao Y. D., *et al.* Fabrication of FeO_3 sites in FeO_x/ZnO for efficient and selective photo-catalytic oxidation of methane to liquid oxygenates. *Cell Rep. Phys. Sci.* **3**, 100909 (2022).

[17] Wang Y. X., Zhang Y. Y., Wang X. Q., Liu Y, Wu ZB. Photothermal direct methane conversion to formaldehyde at the gas-solid interface under ambient pressure. *Nat. Commun.* **16**, 2550 (2025).

[18] Peng H. *et al.* Continuous flow photosynthesis of methanol from methane by plasmonic charge accumulation. *Nat. Commun.* **17**, 344 (2025).
[19] Song H., *et al.* Atomically dispersed nickel anchored on a nitrogen-doped carbon/TiO_2 composite for efficient and selective photocatalytic CH_4 oxidation to oxygenates. *Angew. Chem. Int. Edit.* **62**, e202215057 (2023).
[20] Feng N. D., *et al.* Efficient and selective photocatalytic CH_4 conversion to CH_3OH with O_2 by controlling overoxidation on TiO_2. *Nat. Commun.* **12**, 4652 (2021).
[21] Jiang Y. H., *et al.* Enabling specific photocatalytic methane oxidation by controlling free radical type. *J Chem Soc Chem Comm,* **145**, 2698-2707 (2023).
[22] An B. *et al.* Direct photo-oxidation of methane to methanol over a mono-iron hydroxyl site. *Nat. Mater.* **21**, 932-938 (2022).
[23] Jiang Y. H., *et al.* Elevating photooxidation of methane to formaldehyde via TiO_2 crystal phase engineering. *J. Chem. Soc. Chem. Comm.* **144**, 15977-15987 (2022).
[24] Suzuki T., Wada K., Shima M., Watanabe Y. Photoinduced partial oxidation of methane into formaldehyde on silica-supported molybdena. *J. Chem. Soc. Chem. Comm.*, **15**, 1059-1060 (1990).
[25] Wada K., Yoshida K., Watanabe Y., Suzuki T. The selective photooxidation of methane and ethane with oxygen over zinc-oxide and molybdena-loaded zinc-oxide catalysts. *J. Chem. Soc. Chem. Comm.*, **10**, 726-727 (1991).
