# Supplementary material for: In situ topotactic transition to porous crystals boosts methane photooxidation
Source: Nat Commun. 2026 Jul 13;17:8612. doi: 10.1038/s41467-026-75390-1 (PMC13486515; doi:10.1038/s41467-026-75390-1)
Supplement: Supplementary file 2 [file 41467_2026_75390_MOESM2_ESM.pdf]

Description of additional supplementary files

File Name: Supplementary Data 1

Description: The atomic coordinates of the optimized computational models presented in Figure 6i.
