# Supplementary material for: In situ topotactic transition to porous crystals boosts methane photooxidation
Source: Nat Commun. 2026 Jul 13;17:8612. doi: 10.1038/s41467-026-75390-1 (PMC13486515; doi:10.1038/s41467-026-75390-1)
Supplement: Supplementary file 3 [file 41467_2026_75390_MOESM4_ESM.pdf]

nature portfolio

Peer Review File

In situ Topotactic Transition to Porous Crystals Boosts Methane Photooxidation

Corresponding Author: Professor Kui Xie

Parts of this Peer Review File have been redacted as indicated to remove third-party material.

This file contains all reviewer reports in order by version, followed by all author rebuttals in order by version.

Version 0:

Reviewer comments:

Reviewer #1

(Remarks to the Author)
This manuscript reported a method for preparing centimeter-sized porous single-crystal ZnO (ZB-ZnO) through a topological lattice contraction strategy, and combines it with atomically dispersed Ru sites for the selective photo-oxidation of methane. Though the overall design of this study is excellent and the data is abundant, there is still room for improvement in the in-depth interpretation of the mechanism and the presentation of some data. Besides, the authors devoted a great deal of effort to revealing the atomic-scale structural evolution and pore formation mechanism from β-ZnS to ZB-ZnO. Unfortunately, I believe this manuscript could be more suitable for the Journals focusing on materials. Major concerns are as follows:
1. Lack of quantitative characterization of pore structure: The article presented the pore morphology through SEM/TEM, but lacked key quantitative parameters of the porous structure, such as BET surface area, porosity, and pore size distribution. These data are crucial for evaluating quality transport performance and correlating with catalytic activity.
2. Fairness of catalytic performance comparison: The authors compared Ru1/ZB-ZnO with pure ZB-ZnO and found a significant improvement in performance. However, a crucial control experiment is missing: a performance comparison with Ru single-atom-loaded porous polycrystalline ZnO (or commercial ZnO) having a similar specific surface area and Ru loading. This would help clarify whether the performance enhancement mainly stems from the "single-crystal nature", "porous structure", or "Ru species itself", thereby highlighting the unique advantages of the porous single-crystal support.
3. The mechanism chain for the selective formation of CH3OOH needs to be refined: DRIFTS detected *CH3 and *OOH, and it was speculated that they couple to form CH3OOH. Although this is reasonable, it lacks direct evidence. Suggestion: By conducting isotope labeling experiments (such as with 18O2) and combining with mass spectrometry to detect 18O in the products, it can be clearly confirmed that the oxygen atoms in CH3OOH originate from gaseous O2 rather than lattice oxygen or water, thereby strongly supporting the proposed reaction pathway involving *OOH.
4. Figure 5e indicates that 0.6 wt% is the optimal loading amount; a higher loading will lead to the formation of nanoparticles. It is suggested that HAADF-STEM characterization be conducted on samples with 1-3 wt% loading to visually demonstrate the transition from single atoms to nanoparticles.
5. Although TRPL and TAS have demonstrated enhancement of interfacial charge separation, the oxidation ability of surface holes has not been directly evaluated. It is suggested to conduct additional hole capture experiments.
6. Figure S8 has its title format messed up. Please adjust it.
7. The last sentence on page 1, "and achieve", has a grammatical error. It should be "and achieving". Please review the entire text.
8. In the photocatalysis test, the light source intensity "1000 mW·cm2" is extremely high. Is this considered as quasi-sunlight? Please confirm or explain whether this intensity has been calibrated with a filter.

Reviewer #2

(Remarks to the Author)
In this manuscript, the authors report a topotactic lattice contraction strategy to fabricate porous single-crystalline (PSC) zincblende-ZnO (ZB-ZnO) monoliths from β-ZnS. Using in situ transmission electron microscopy, they directly observed the dynamic formation of PSC ZB-ZnO at atomic resolution, unveiling preferential anion migration along {222} channels and strain-driven vacancy aggregation that leads to nanopore generation while maintaining single-crystalline order. Following the incorporation of atomically dispersed Ru sites, the resulted catalyst presents a 82% selectivity for methyl hydroperoxide

(CH_3OOH) in methane oxidation under visible light (AM 1.5G), along with a good stability of 40 hours (<15% activity decay). This work shows an important strategy to synthesize single crystals with porosity, opening new frontiers in synthetic chemistry and material property engineering. The reviewer would suggest to accept it after addressing the following points:

1. While XRD 2θ patterns alone cannot unambiguously confirm single crystallinity, complementary techniques such as Φ-scanning are critical. Cubic ZB-ZnO should exhibit four-fold symmetry (Figure 1a), yet the Φ-scan shows only two-fold periodicity (90° intervals, see in the Figure 1e)). Please clarify whether this discrepancy arises from specific crystallographic orientation constraints or measurement methodology, and explain how to comprehensively verify the ZnO single crystallinity.

2. Hexagonal wurtzite is the thermodynamically stable phase of ZnO. Why does a cubic phase, instead of hexagonal, form when ZnS is oxidized? Is sulfur completely removed after the phase transformation? In the manuscript, the transition temperature from β-ZnS to cubic ZnO was recorded at 500°C. Would a higher temperature trigger structural reorganization?

3. Figure 4 illustrates the topological transformation process from β-ZnS to PSC ZB-ZnO observed via in situ high-resolution transmission electron microscopy (in situ HRTEM). The temporal resolution of this phase evolution depends on the spatial statistical distribution of the Zn–Zn atomic spacing along the ⟨112⟩ crystallographic direction. How is the spatial distribution of this atomic spacing precisely statistically analyzed?

4. Based on Figure 5e, it can be observed that the loading amount of Ru significantly impacts the catalytic performance. The selectivity for methyl hydroperoxide initially increases then decreases with increasing Ru loading levels. What causes this trend? Is the structural evolution of Ru species the primary factor? Will the porous structure change under different loading amounts?

5. Figure 5 presents the catalyst's exceptional performance in methane oxidation to methyl hydroperoxide, with an extremely high stability. Suggest authors to discuss this merit with detailed information. In addition, could the porous single-crystal framework inhibit Ru leaching?

Reviewer #3

(Remarks to the Author)
The authors successfully transformed β-ZnS into porous single-crystal zinc blende ZnO (ZB-ZnO) under an O_2/Ar atmosphere and subsequently loaded Ru to enable selective photocatalytic oxidation of methane, a process with considerable practical application potential. Crystal structure characterization techniques, including ex-situ XRD, Laue microdiffraction, selected area electron diffraction, and in-situ high-resolution transmission electron microscopy, were employed to elucidate the lattice reorganization mechanism during the solid-state phase transition. Furthermore, the study investigates the reaction mechanism of methane oxidation on Ru-loaded ZB-ZnO. This manuscript presents notable innovation and promising applications in solid material design. Nevertheless, it requires further strengthening in establishing clear structure-activity relationships, enhancing data depth, and improving the logical coherence of the narrative, to meet the publication standards of high-impact journals. The following are the review comments.

1.This manuscript suffers from structural flaws and an absence of thematic coherence. While the initial sections focus extensively on the synthesis mechanism of porous single-crystalline ZB-ZnO, the subsequent discussion fails to adequately elucidate its role in determining photocatalytic performance and underlying reaction mechanisms. Furthermore, the introduction fails to establish a logical connection between the scientific background and the functional significance of the material. The primary objective of the study requires clarification: is the work intended to present a novel synthetic approach, an advancement in mechanistic understanding, or a significant improvement in catalytic performance?

2.The manuscript describes lattice reorganization but lacks dynamic visualization of phase evolution. Include operando or time-resolved imaging, or 3D tomography (e.g., FIB-SEM) to show pore connectivity and crystallographic consistency.

3.What are the characteristics or advantages of ZnO compared to other types of photocatalytic materials? Why is ZnO chosen for photocatalytic methane oxidation? In addition, in the aspect of photocatalytic methane oxidation, what advantages do porous single-crystal ZB-ZnO or Ru/ZB-ZnO have over amorphous ZnO or Ru/amorphous ZnO? The key parameters - band structure, charge separation efficiency, charge transfer kinetics and surface/interface properties should be analyzed. Key data - UV-Vis DRS (Tauc plot), flat band potential, EIS, work function and steady-state PL must be provided to evaluate the photoelectric response and electronic structure of the materials.

4. Is the term "topological transformation" used in the manuscript scientifically reasonable? Have the proposed topological properties been strictly supported by structural evidence? Moreover, the reproducibility of this phase transition mechanism has not yet been confirmed. The authors should provide statistical data of crystallographic parameters (such as lattice constants and phase purity) from multiple synthesis batches, with deviations within an acceptable range, to demonstrate its robustness.

5.The long-term stability of atomically dispersed Ru species under operational conditions is a key concern. The authors should provide evidence, such as post-reaction HAADF-STEM or XAS, to confirm that Ru remains as isolated atoms without aggregation during prolonged catalytic cycling.

6.The mechanistic discussion of hydrogen and oxygen transfer pathways lacks definitive experimental support. Isotope labeling experiments (e.g., using $^{18}O_2$ or CD_4) are recommended to trace the origin of oxygen and hydrogen atoms in the

methyl hydroperoxide product and to validate the proposed reaction mechanism.

7.The photocatalytic performance measurements in this manuscript were conducted under a light intensity of 1000 mW/cm², which is ten times the intensity of sunlight. Therefore, the light source should not be described as AM 1.5G (100 mW/cm²). Under such high-intensity illumination, significant heating of the catalyst surface is expected, which raises concerns about potential thermal contributions to the observed reactivity. As a result, it becomes difficult to distinguish whether the catalytic process is purely photocatalytic or involves photo-thermal effects. To clarify the reaction mechanism, direct measurement of the catalyst surface temperature during irradiation is essential. Furthermore, a rigorous evaluation should be provided to demonstrate that the observed methane oxidation is driven solely by photocatalytic processes, rather than by thermally activated pathways.

8.Please provide a clear photo of the gas-phase continuous-flow photoreactor used in this research so that readers can have a more intuitive understanding of the application situation.

9.The most valuable products in methane selective oxidation are typically multi-carbon compounds formed via C–C coupling reactions. In contrast, all products reported in this study are single-carbon (C1) species, and the formation rates of the three detected products under 10-sun illumination are all below 100 μmol/h. Does this level of performance demonstrate competitiveness under comparable irradiation conditions? To enable a rigorous comparison, please provide a detailed summary table of relevant studies, including key experimental parameters such as light intensity, reaction temperature, catalyst loading, photocatalytic reactor configuration, and gas composition.

10.What specific reactions do photogenerated electrons and holes undergo at the surface of the photocatalyst during the photocatalytic process? While theoretical calculations can provide valuable insights, they are insufficient as standalone evidence. It is essential to support mechanistic interpretations with robust experimental data to reliably elucidate the reaction pathways of charge carriers.

11.What roles do Ru and ZnO play individually in the photocatalytic process? Is there evidence of a synergistic interaction between Ru and ZnO under illumination? Furthermore, what are the specific adsorption sites on the catalyst surface for CH4, H2O, and O2, respectively? To gain mechanistic insight into the reaction pathways and surface processes, comprehensive in-situ spectroscopic investigations—such as in-situ DRIFTS, XPS, or Raman spectroscopy—should be conducted to dynamically monitor the evolution of surface species and intermediate states during the catalytic cycle.

12.Please provide the original gas chromatography (GC) data and gas chromatography-mass spectrometry (GC-MS) data for the photocatalytic gaseous products to ensure that all potential by-products are fully accounted for and not overlooked.

13.In Figure 6 and Table S3, the normalized TAS decay kinetics fitting lifetimes of ZB-ZnO and Ru1/ZB-ZnO are opposite.

Version 1:

Reviewer comments:

Reviewer #1

(Remarks to the Author)
The authors have addressed most of the concerns.
The light intensity is 1000 mW/cm2, which is relatively high. Evaluating the photocatalytic performance in terms of apparent quantum efficiency is more reasonable. How about the apparent quantum efficiency of Ru1/PSCZB-ZnO?

Reviewer #2

(Remarks to the Author)
The revision dramatically improves the quality of this work and clarity of the presentation. The reviewer does not have other comment and would suggest to accept it.

Reviewer #3

(Remarks to the Author)
The authors have responded well to the majority of comments from reviewers. And the manuscript is nearly ready for acceptance. Please address one remaining point:
Generally, photocatalyst surface temperature will rises significantly under strong light irradiation (1000 mW/cm2). Thus, the observed proportionality between photocatalytic efficiency and irradiation intensity alone does not confirm a purely photocatalytic mechanism (without photothermal effect). Please clarify how surface temperature was measured. A thermocouple measures ambient temperature near the catalyst, not the actual irradiated surface temperature. Use an infrared thermometer to measure the transient surface temperature immediately after prolonged illumination, values often exceed 32 °C. This clarification will strengthen the mechanistic interpretation without affecting the manuscript’s quality or impact.

Version 2:

Reviewer comments:

Reviewer #1

(Remarks to the Author)
The authors have significantly improved the quality of this work. I have no further comments and recommend acceptance of the manuscript.

Reviewer #3

(Remarks to the Author)
The authors have replied well to these comments and suggestions. It is suggested that the manuscripts be received directly.

Open Access This Peer Review File is licensed under a Creative Commons Attribution 4.0 International License, which permits use, sharing, adaptation, distribution and reproduction in any medium or format, as long as you give appropriate credit to the original author(s) and the source, provide a link to the Creative Commons license, and indicate if changes were made.
In cases where reviewers are anonymous, credit should be given to 'Anonymous Referee' and the source.
The images or other third party material in this Peer Review File are included in the article's Creative Commons license, unless indicated otherwise in a credit line to the material. If material is not included in the article's Creative Commons license and your intended use is not permitted by statutory regulation or exceeds the permitted use, you will need to obtain permission directly from the copyright holder.
To view a copy of this license, visit https://creativecommons.org/licenses/by/4.0/

Response to reviewers

Response to Reviewer #1

General comments: This manuscript reported a method for preparing centimeter-sized porous single-crystal ZnO (ZB-ZnO) through a topological lattice contraction strategy, and combines it with atomically dispersed Ru sites for the selective photo-oxidation of methane. Though the overall design of this study is excellent and the data is abundant, there is still room for improvement in the in-depth interpretation of the mechanism and the presentation of some data. Besides, the authors devoted a great deal of effort to revealing the atomic-scale structural evolution and pore formation mechanism from β-ZnS to ZB-ZnO. Unfortunately, I believe this manuscript could be more suitable for the Journals focusing on materials. Major concerns are as follows:

Response: We sincerely thank you for your thoughtful comments, which have significantly contributed to enhancing our manuscript. In response, we have meticulously revised the main text to deepen the interpretation of the mechanisms and refine the data presentation.

1. We now already explicitly compare porous single-crystalline ZnO (PSC ZB-ZnO) with porous polycrystalline ZnO (PPC ZB-ZnO) as parallel platforms. While UV–vis DRS, UPS, VB-XPS, and Mott–Schottky analyses (**Supplementary Figs. 35–39**) confirm their similar band structures, the key distinction lies in their charge dynamics. As evidenced by steady-state/time-resolved PL, EIS, and fs-TAS (**Fig. 6a–c, Supplementary Figs. 41–44**), PSC ZnO inherently suppresses charge recombination and enables rapid carrier transport. The introduction of Ru single atoms further enhances this system by acting as efficient electron traps, prolonging carrier lifetimes and reducing interfacial resistance.

2. A suite of *in situ* spectroscopic investigations delineates the reaction pathway. *In situ* XPS (**Fig. 6d, Supplementary Fig. 40**) demonstrate that photoexcited electrons accumulate on Ru sites and holes localize on ZnO lattice oxygen. TPD experiments (**Supplementary Fig. S45**) identifies selective adsorption: CH_4 on ZnO and O_2 on Ru sites. *In situ* EPR (**Fig. 6e–f**) demonstrates that Ru addition switches the dominant radical from •OH (on ZnO) to •OOH (on Ru-containing catalysts). This is directly linked to the product *via* ^{18}O-labelling (**Fig. 6f**), confirming that the oxygen atom in CH_3OOH comes from gaseous O_2. Quenching experiments (**Supplementary Fig. S46**) verify the dominant role of photogenerated holes (h^+) in methane activating. *In situ* DRIFTS (**Fig. 6g–h, Supplementary Fig. S47**) shows that on Ru_1/PSC ZB-ZnO the bands assigned to adsorbed $*CH_3$ and •OOH grow concurrently, consistent with a surface coupling of $•CH_3$ (generated by hole oxidation of CH_4 on ZnO) with Ru-derived •OOH to form CH_3OOH. Finally, DFT calculations provided a thermodynamic explanation for the selective generation of CH_3OOH on Ru_1/PSC ZB-ZnO. In summary, we could obtain the catalytic mechanism diagram: Upon light irradiation, the electrons are excited from VB to CB of ZnO, and then transfer to the decorated Ru single atoms, wherein O_2 is selectively reduced to •OOH. Concurrently, the holes remaining in VB of ZnO activate adsorbed CH_4 to form $•CH_3$, which can then couple with •OOH to yield CH_3OOH as the primary product. Additionally, the •OH radicals generated from H_2O oxidation may further promote CH_4 activation, contributing to the overall reaction pathway.

3. To strengthen structural coherence, we have systematically reorganized the manuscript to establish clear logical links from synthesis, atomic-scale transformation mechanism, and charge transport

properties to catalytic function. The role of the porous single-crystal (PSC) architecture is now explicitly decoupled through controlled comparisons with porous polycrystalline counterparts. Key evidence includes prolonged carrier lifetime (TRPL), reduced charge-transfer resistance (EIS), and enhanced radical generation (EPR) for PSC samples, directly attributing the superior activity to grain-boundary elimination and long-range crystallographic order. This revised manuscript presents a unified structural–mechanistic framework, which, combined with its innovative synthesis and exceptional performance, underscores the scientific significance of the work and its alignment with the interdisciplinary scope of *Nature Communications*.

Comment 1: Lack of quantitative characterization of pore structure: The article presented the pore morphology through SEM/TEM, but lacked key quantitative parameters of the porous structure, such as BET surface area, porosity, and pore size distribution. These data are crucial for evaluating quality transport performance and correlating with catalytic activity.

Response: We sincerely appreciate the reviewer's insightful comment regarding the quantitative characterization of pore structure. We have conducted comprehensive N_2 physisorption measurements on PSC ZB-ZnO (**Supplementary Figure S5**). The results indicate that PSC ZB-ZnO possesses a specific surface area of ~16 m^2/g, a pore structure centered around ~82 nm, and a porosity of ~42%. To further elucidate the three-dimensional pore architecture, we conducted a detailed reconstruction analysis (as shown in **Supplementary Figure S6**). The 3D reconstruction reveals the skeletal framework, continuous pore system, and interconnected pore-throat network, with an average pore diameter of ~80 nm and throat length of ~230 nm. These structural features confirm a well-connected porous architecture, which is favorable for efficient mass transport.

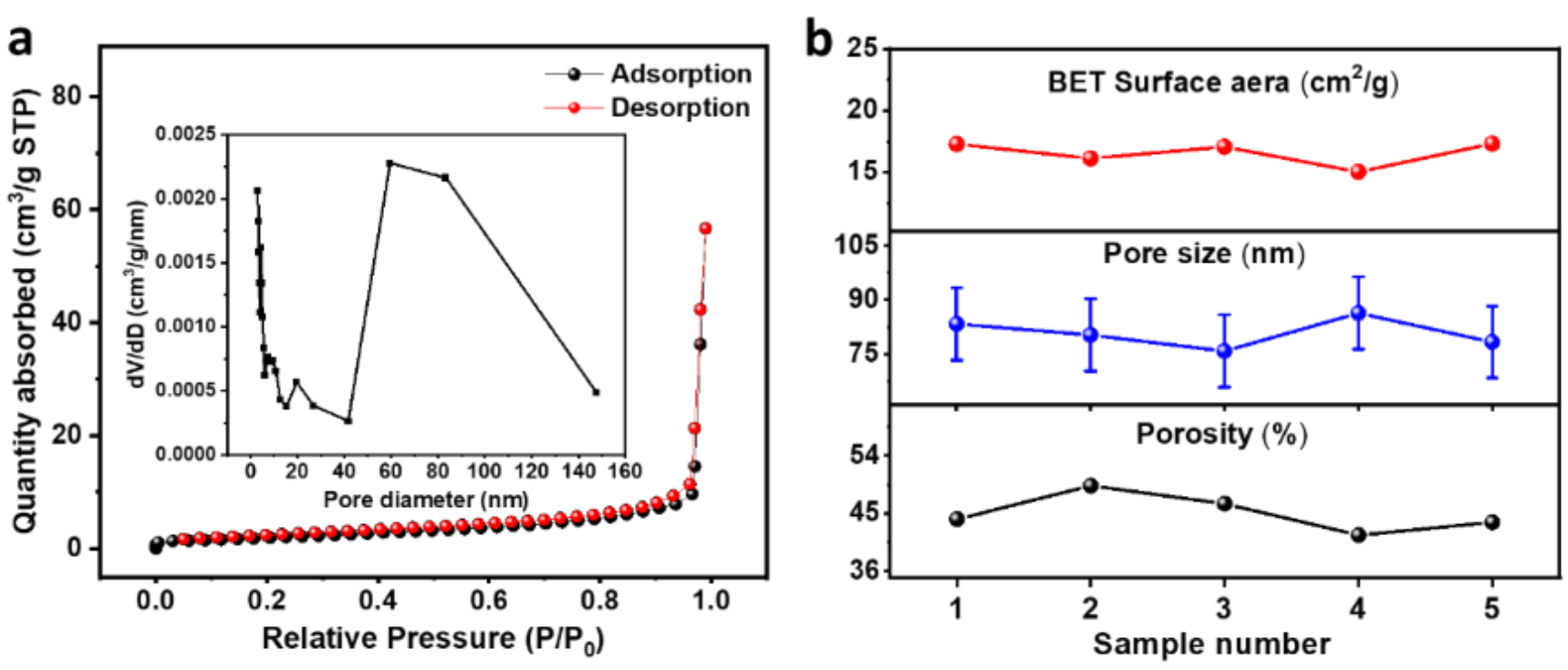

Supplementary Figure S5. Pore structure characterization of PSC ZB-ZnO. (a) Nitrogen adsorption/ desorption isotherms with the corresponding pore-size distribution curve shown in the inset. (b) BET surface area, pore size and porosity of the synthesized samples.

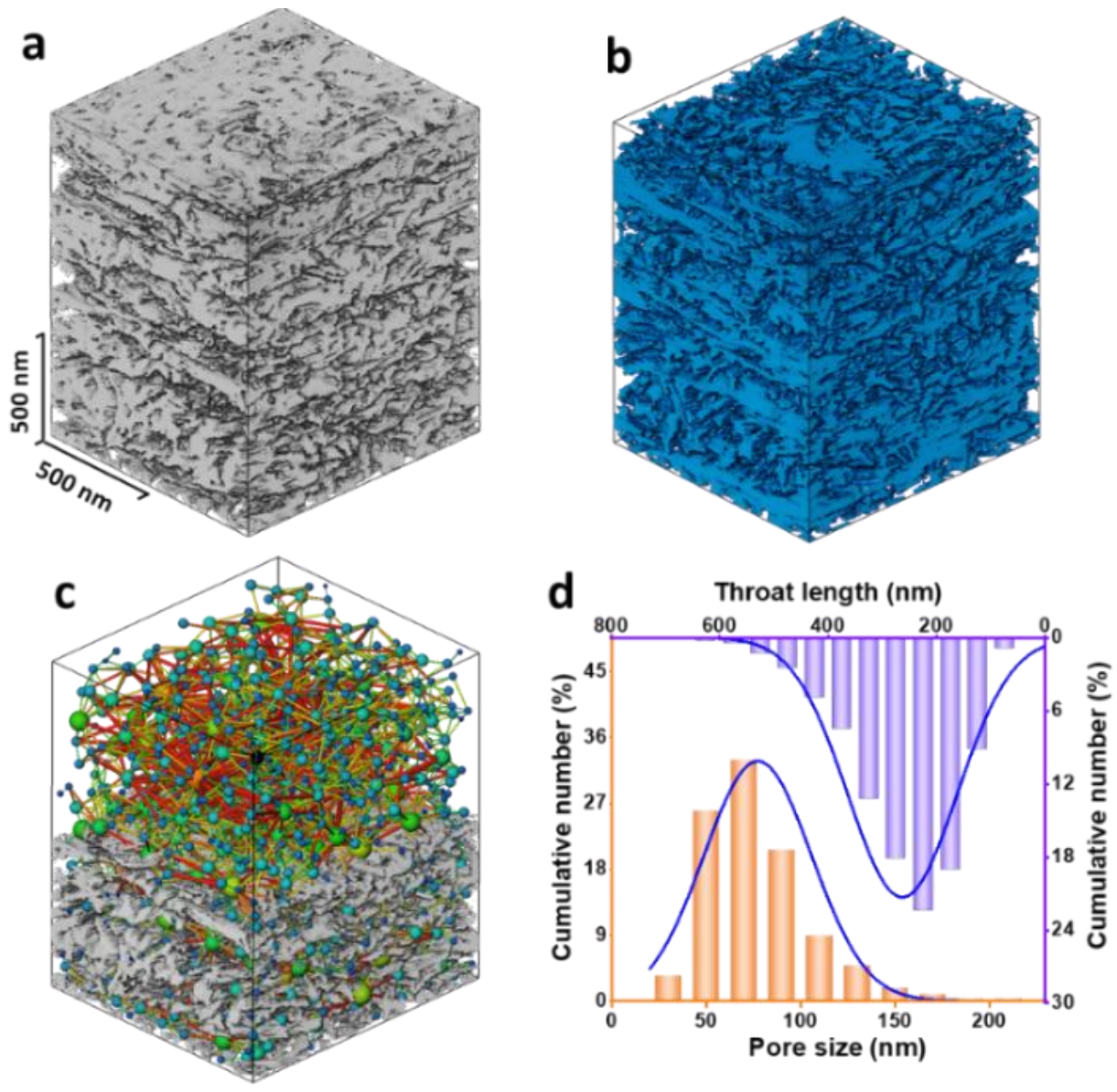

Supplementary Figure S6. 3D reconstruction analysis of PSC ZnO: (a) skeletal framework structure, (b) pore system, (c) ball-stick model of interconnected pores and throats, (d) pore size and throat length distribution.

Comment 2: Fairness of catalytic performance comparison: The authors compared Ru_1/ZB-ZnO with pure ZB-ZnO and found a significant improvement in performance. However, a crucial control experiment is missing: a performance comparison with Ru single-atom-loaded porous polycrystalline ZnO (or commercial ZnO) having a similar specific surface area and Ru loading. This would help clarify whether the performance enhancement mainly stems from the "single-crystal nature", "porous structure", or "Ru species itself", thereby highlighting the unique advantages of the porous single-crystal support.

Response: Thank you for raising this critical point regarding the catalytic performance comparisons. We have conducted supplementary experiments by synthesizing porous polycrystalline ZB-ZnO (PPC ZB-ZnO) and Ru single-atom-modified porous polycrystalline ZB-ZnO (Ru_1/PPC ZB-ZnO), as characterized in **Supplementary Figure S30**. As shown in **Table S3**, all samples exhibit comparable BET surface areas (15.7 -16.7 m^2/g). Meanwhile, Ru_1/PSC ZB-ZnO and Ru_1/PPC ZB-ZnO presents similar Ru loadings (0.32 vs. 0.30 wt%). The photocatalytic methane oxidation performance (**Fig. 5f**) reveals that porous single-crystalline structure (PSC ZB-ZnO, Ru_1/PSC ZB-ZnO) exhibit superior performance to porous polycrystalline structure (PPC ZB-ZnO, Ru_1/PPC ZB-ZnO). Moreover, Ru atoms modification further enhances the activity and markedly improves the selectivity toward CH_3OOH.

To elucidate the origin of this performance difference, we analyzed the electronic structures of all catalysts. As shown by the UV-vis diffuse reflectance spectra (UV-vis DRS) and corresponding Kubelka-Munk plots (**Supplementary Figure 35**), the bandgaps are nearly identical between the porous single-crystal and polycrystalline ZnO supports (PSC ZB-ZnO: 3.16 eV; PSC ZB-ZnO: 3.17 eV). However, upon Ru modification, the bandgap slightly narrows (Ru_1/PSC ZB-ZnO: 3.13 eV; Ru_1/PPC ZB-ZnO: 3.14 eV), indicating that Ru incorporation induces a marginal red-shift of the absorption edge, which is beneficial for enhancing visible-light absorption. Through UPS, VB-XPS, and Mott-Schottky analysis (**Supplementary Figure 36-38**), we constructed the band structure diagram (**Supplementary Figure**

39), which shows that single-crystalline and polycrystalline ZnO have similar band alignments, while Ru modification causes a minor negative shift of both the valence band (VB) and conduction band (CB). This optimized band alignment in Ru modified ZnO facilitates more efficient charge separation and stronger redox driving forces. Based on the positions of VB/CB and the thermodynamic potentials of oxygen activation reactions (H_2O/•OH: 2.380 V; O_2/•OOH: 0.05 V), all four catalysts can generate •OH and •OOH active species *via* H_2O oxidation and O_2 reduction, respectively.

To probe charge carrier dynamics, photoluminescence (PL) and electrochemical analyses were conducted. The steady-state PL spectra (Supplementary **Fig. S41**) show that the PL emission intensity follows the trend: PPC ZB-ZnO > PSC ZB-ZnO > Ru_1/PPC ZB-ZnO > Ru_1/PSC ZB-ZnO. This indicates that the porous single-crystalline framework itself suppresses recombination relative to its polycrystalline counterpart by eliminating grain boundaries, while Ru single atoms as an effective electron sink further quenches the PL signal. This synergistic suppression of recombination is quantitatively validated by Time-resolved PL decay measurements (**Fig. 6b**). The average carrier lifetime (τ_{avg}) of Ru_1/PSC ZB-ZnO is 22.3 ns, significantly longer than that of Ru_1/PPC ZB-ZnO (16.4 ns). Even without Ru, pristine PSC ZB-ZnO exhibits a longer lifetime (τ_{avg} = 17.0 ns) than PPC ZB-ZnO (τ_{avg} = 12.4 ns), highlighting the intrinsic advantage of the single-crystalline framework in prolonging charge carrier lifetime. Ru modification further extends the lifetime, indicating enhanced survival of photogenerated carriers for surface reactions. Electrochemical impedance spectroscopy (EIS, Supplementary **Fig. 42**) further reveals lower charge transfer resistance for single-crystalline ZnO compared to polycrystalline ZnO, with Ru-modified catalysts showing the lowest resistance. These results underscore a synergistic interplay: the single-crystalline structure provides a conductive, low-defect bulk transport pathway, while atomically dispersed Ru sites accelerate interfacial charge-transfer kinetics.

In summary, the superior performance of Ru_1/PSC ZB-ZnO stems from a powerful synergy: the porous single-crystal support inherently minimizes bulk recombination and facilitates efficient charge transport, while the Ru single atoms further boost charge separation and direct surface reaction pathways. This combination is uniquely enabled by our topotactically synthesized porous single-crystalline architecture. Moreover, this advantage is further accentuated upon Ru modification. Specifically, the Ru-modified systems display a notably smaller semicircle radius in the Nyquist plots, evidencing a significant further reduction in charge-transfer resistance. This underscores a synergistic interplay, where the single-crystal morphology provides an efficient conductive pathway, and the atomic-level Ru incorporation actively enhances the interfacial charge-transfer kinetics.

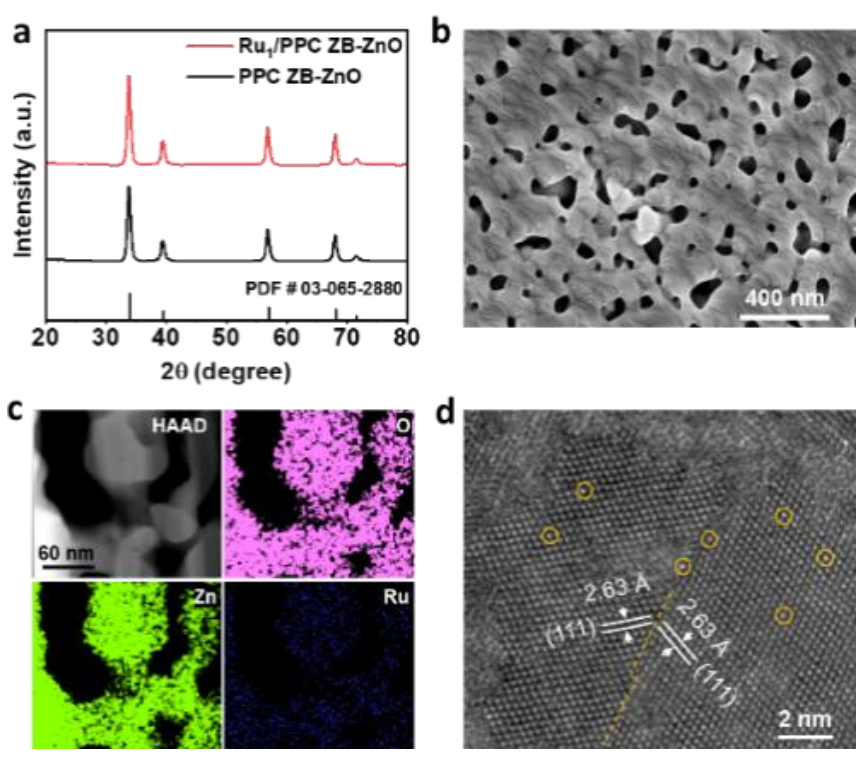

Supplementary Figure S30. Structural and Morphological Characterization of Ru_1/PPC ZB-ZnO. (a)

XRD patterns of Ru_1/PPC ZB-ZnO and PPC ZB-ZnO, where diffraction peaks are indexed to PDF card #03-065-2880, confirming the cubic phase of the as prepared material. (b) SEM image of Ru_1/PPC ZB-ZnO, exhibiting a porous architecture with uniform pore distribution. (c) Elemental mapping of Ru_1/PPC ZB-ZnO, illustrating the homogeneous dispersion of Ru, Zn, and O species throughout the porous matrix. (d) HAADF STEM Image of Ru_1/PPC ZB-ZnO.

Table S3. Physicochemical properties of ZnO-based catalysts.

Catalysts	BET surface area (m^2/g)	Ru loading (wt%)
PSC ZB-ZnO	16.1	0
PPC ZB-ZnO	16.6	0
Ru_1/PSC ZB-ZnO	15.6	0.30
Ru_1/PPC ZB-ZnO	15.7	0.32

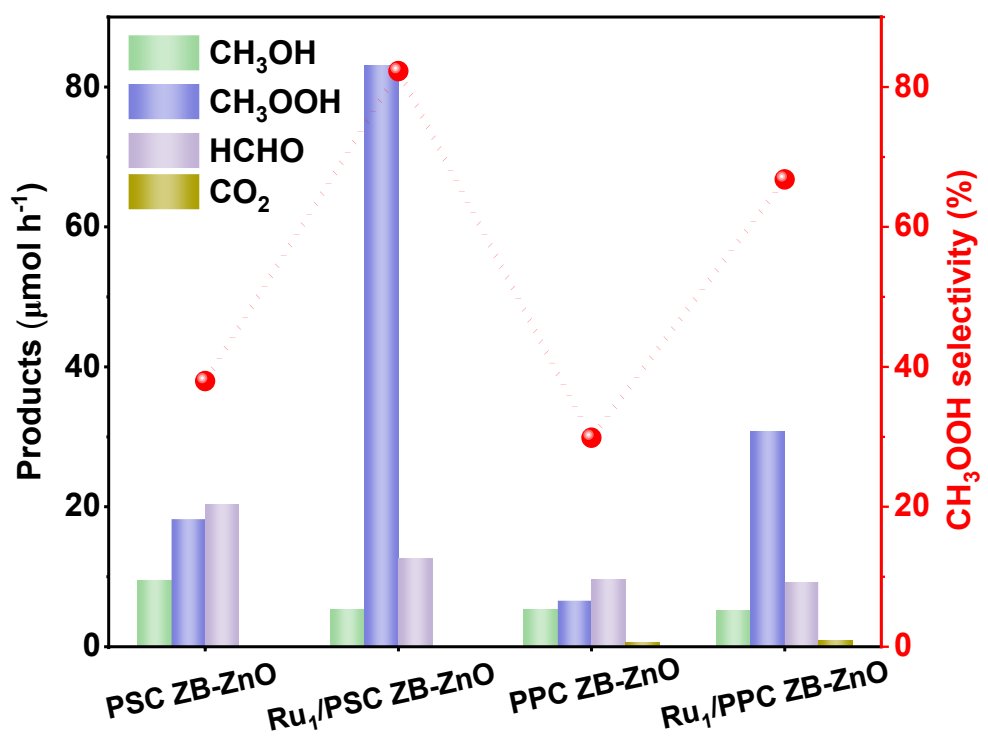

Fig. 5f. Photocatalytic methane oxidation performance of PSC ZB-ZnO, Ru_1/PSC ZB-ZnO, PPC ZB-ZnO, and Ru_1/PPC ZB-ZnO.

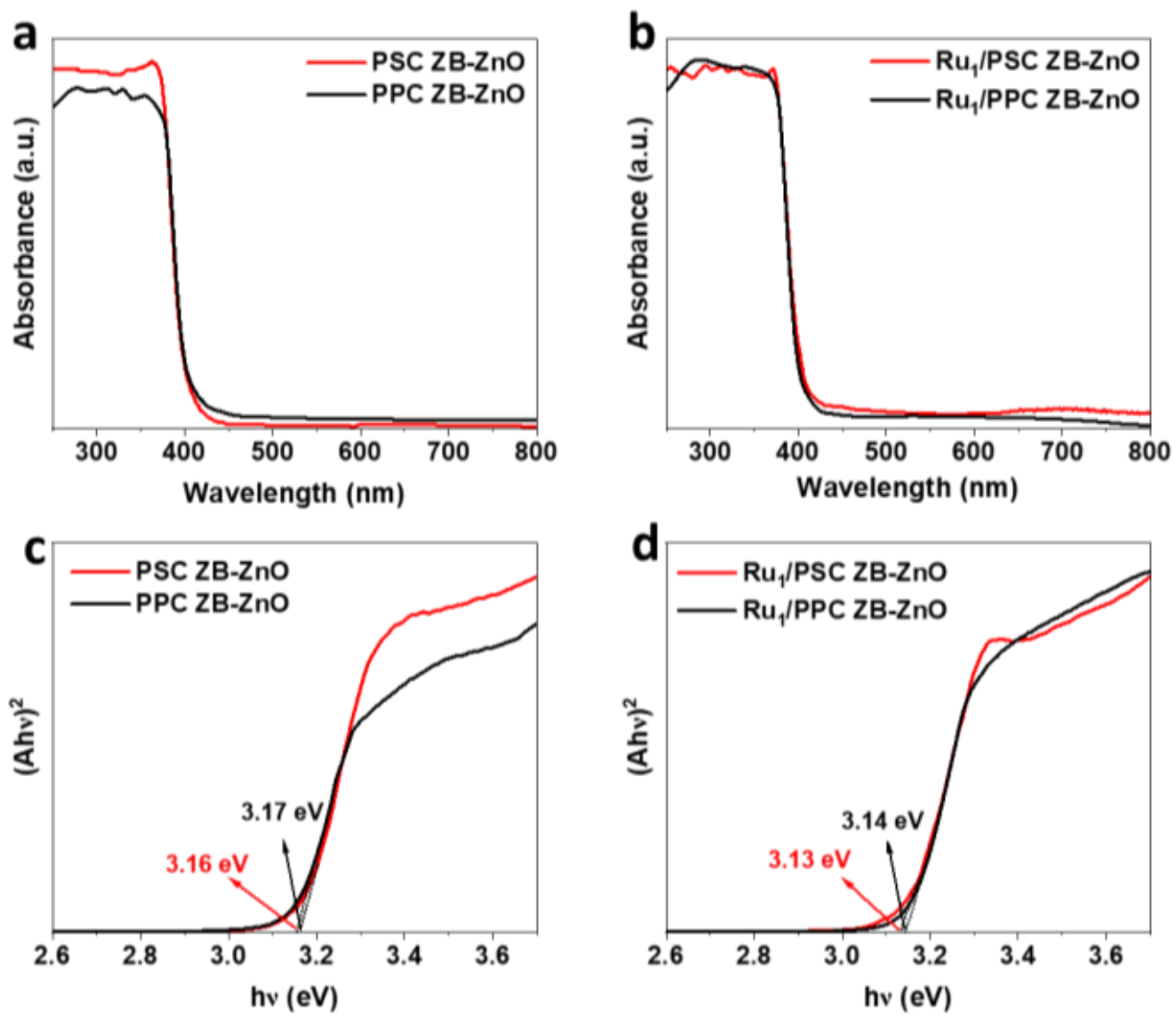

Supplementary Figure S35. Photophysical properties of ZnO based catalysts with/without Ru

modification. (a) UV−visible diffuse reflectance spectra of PSC ZB-ZnO, PPC ZB-ZnO, and PPC ZnO-h. (b) UV−visible diffuse reflectance spectra of Ru_1/PPC ZnO-h. (c-d) Transformed Kubelka-Munk function versus photon energy. The determined bandgaps are E_g = 3.16 eV (PSC ZB-ZnO), 3.17 eV (PPC ZB-ZnO), 3.13 eV (Ru_1/PSC ZB-ZnO), and 3.14 eV (Ru_1/PPC ZB-ZnO), highlighting the influence of Ru incorporation and support (PPC vs PSC) on the electronic structure of ZnO.

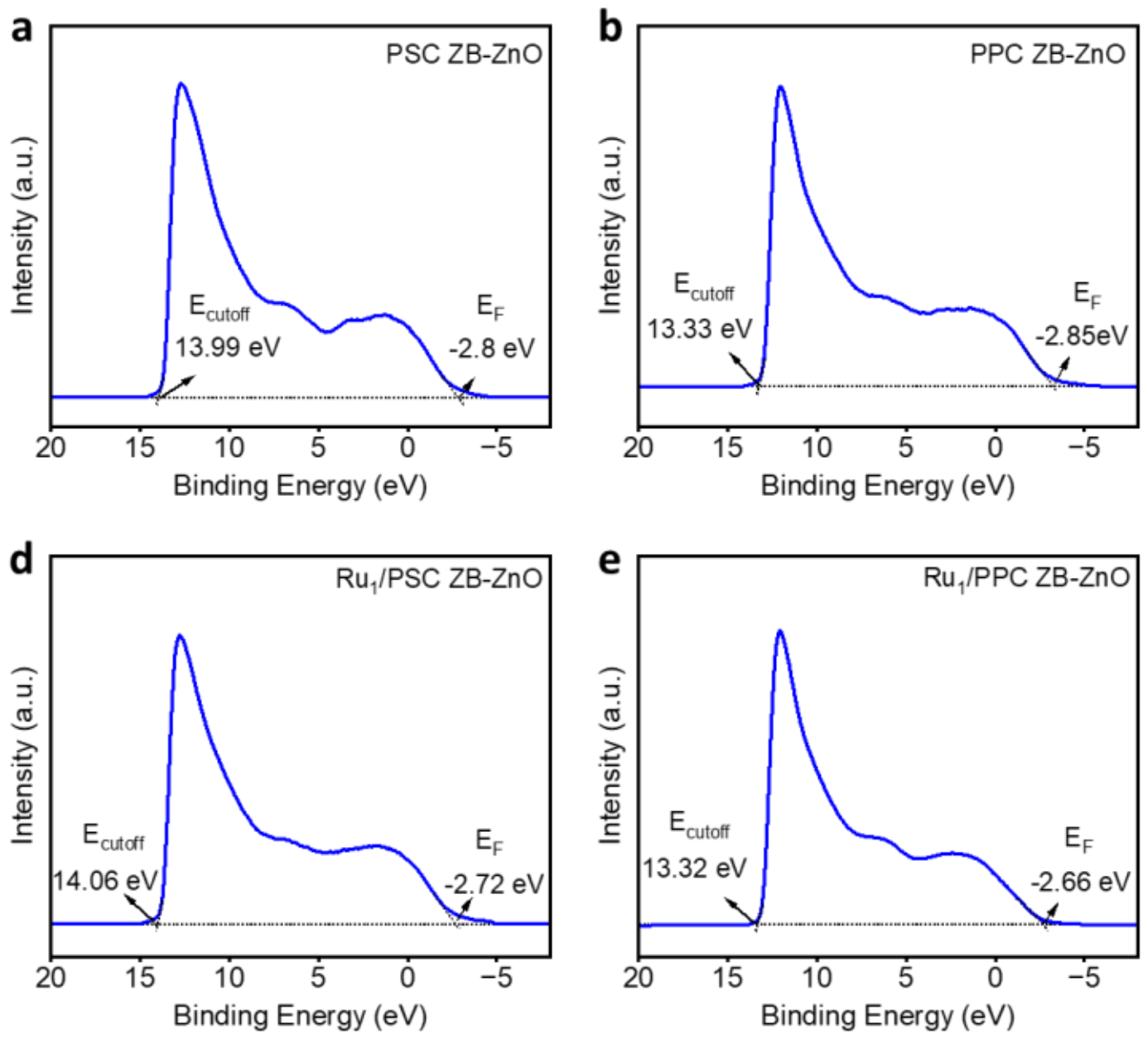

Supplementary Figure S36. UPS spectrums of ZB-ZnO based catalysts. Thie electron work function of the analyzer was using the formula: $\Phi = h\nu - (E_{cutoff} - E_F)$ ($h\nu$ =21.22 eV).

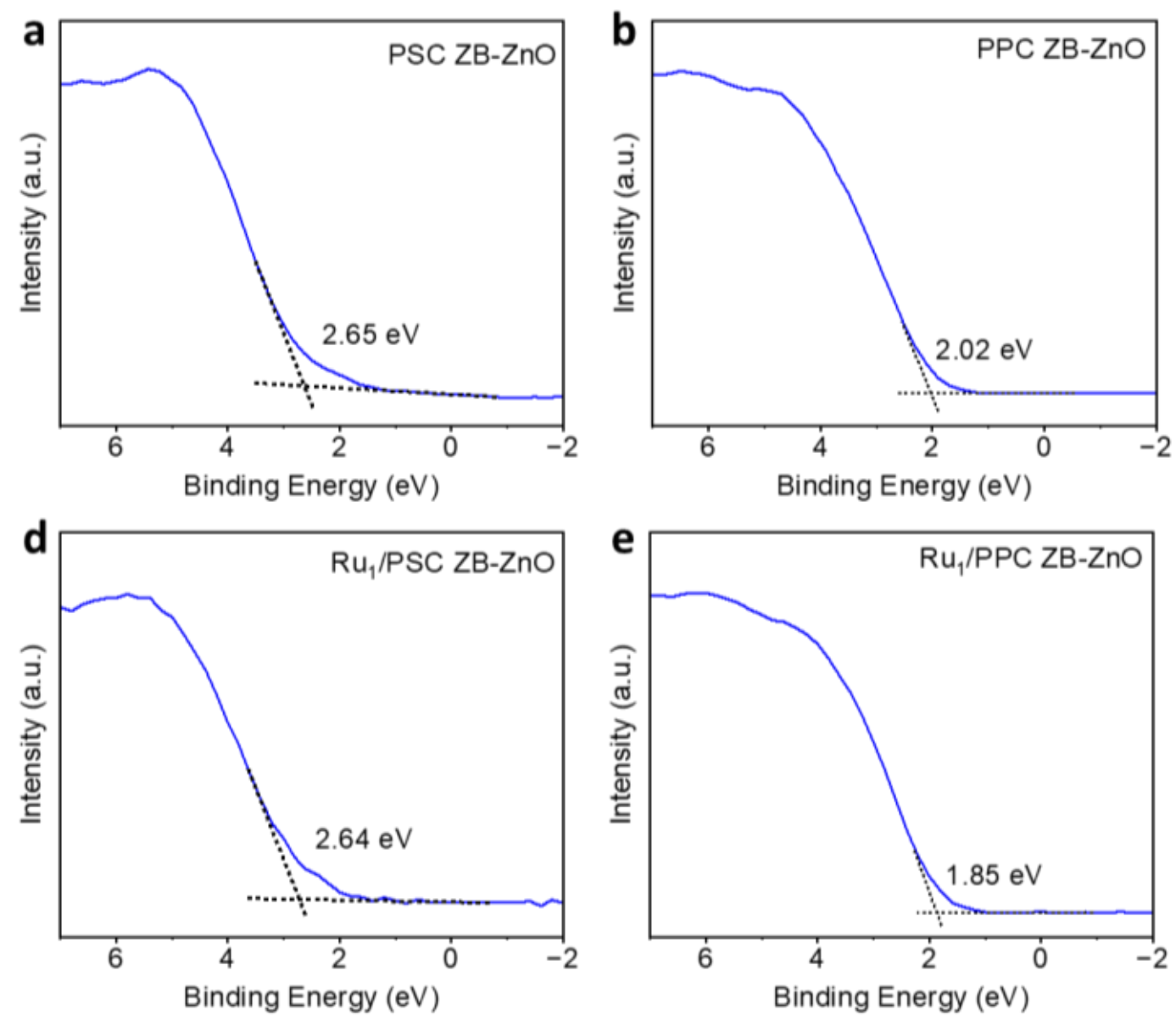

Supplementary Figure S37. VB-XPS spectrum of ZB-ZnO based catalysts. E_{VB} vs. NHE (V) was calculated using the formula $E_{VB} = \Phi + VB - 4.44$.

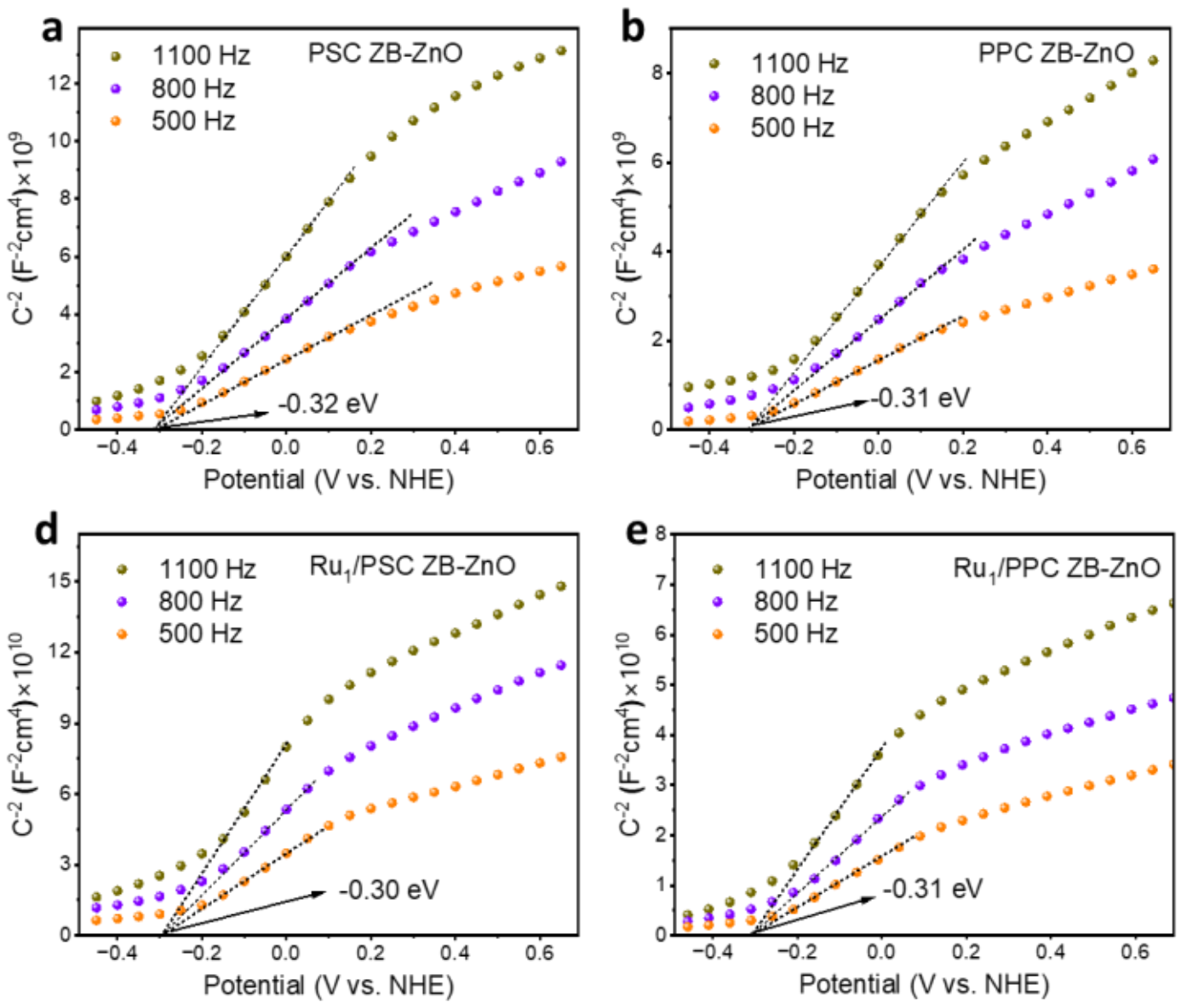

Supplementary Figure S38. Mott Schottky plots of ZB-ZnO based catalysts. All the samples showed the positive slope of the Mott-Schottky plot, implying n type semiconductors. The flat band potential (E_{fb}) were determined by extending the linear portion of the MS plots. In general, the conduction band potential (E_{CB}) was considered to be ~0.2 eV negative than the E_{fb}.

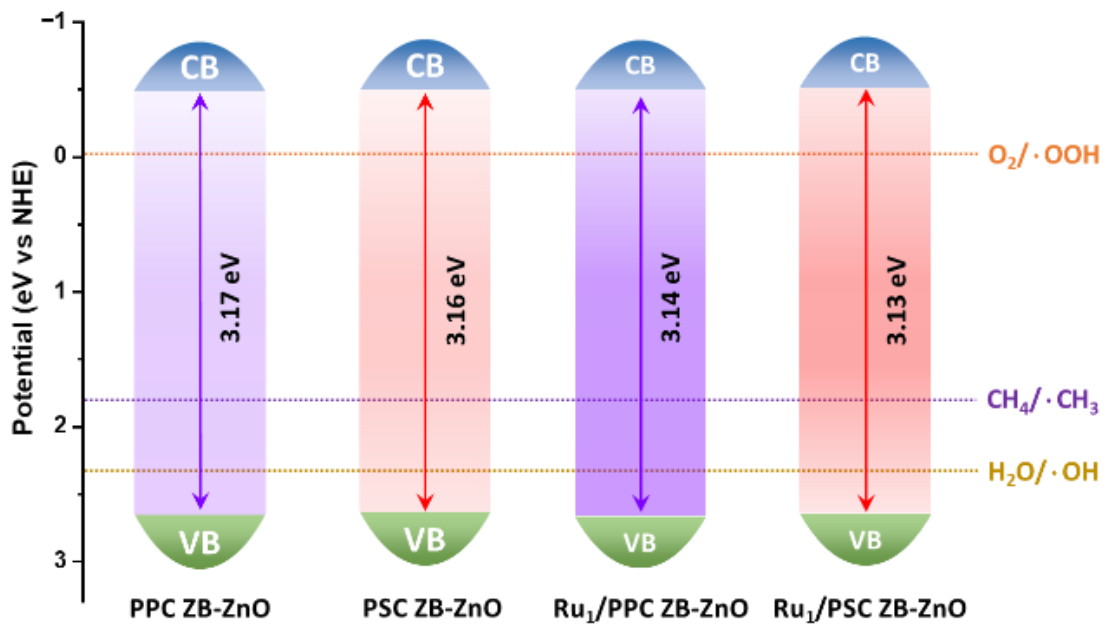

Supplementary Figure S39. Schematic illustration of the band structures of PPC ZB-ZnO, PSC ZB-ZnO, Ru_1/PPC ZB-ZnO, and Ru_1/PSC ZB-ZnO. determined by VB-XPS valence spectra, UPS spectrums and Mott−Schottky plots.

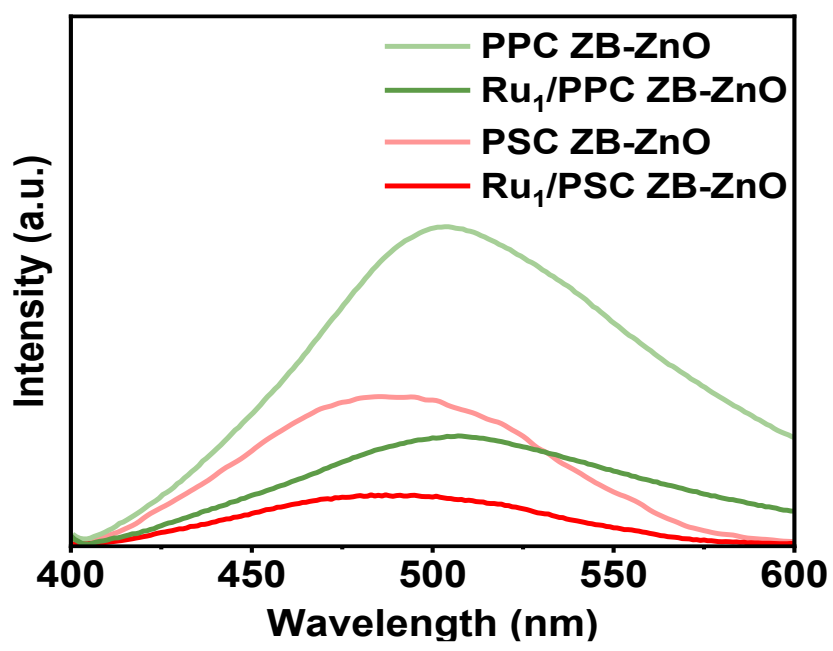

Supplementary Figure S41. Steady-state PL characterizations of ZnO samples, with and without Ru single-atom modification. Ru modification enhances the charge separation efficiency of ZnO-based

photocatalysts, while the single-crystalline structure synergistically suppresses the recombination of photo-generated carriers.

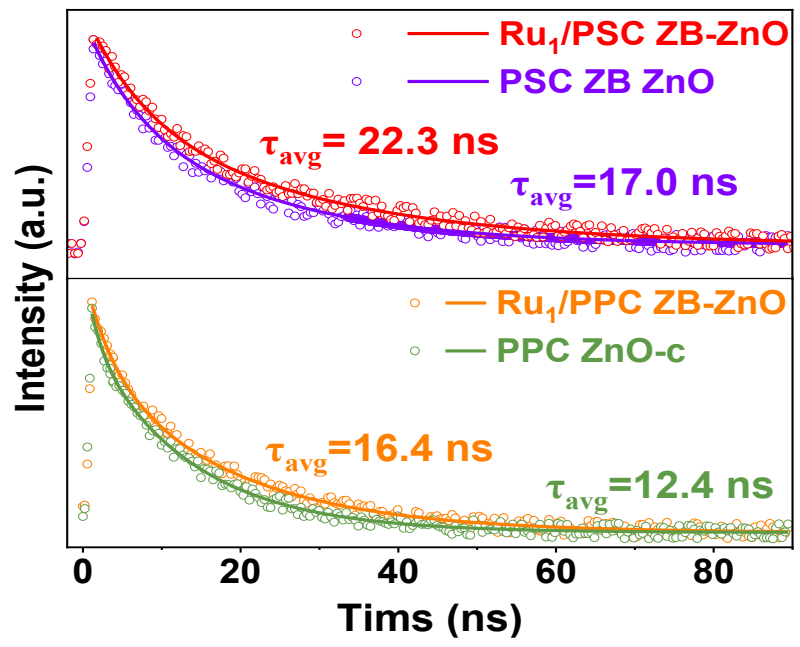

Fig. 6b. Time-resolved PL decay of PSC ZB-ZnO and PPC ZB-ZnO, with and without Ru single-atom modification. The average carrier lifetime (τ_{avg}) for Ru_1/PSC ZB-ZnO, Ru_1/PPC ZB-ZnO, PSC ZB-ZnO, PPC ZB-ZnO are 17.4, 12.6, 14.33, 12.63 ns, respectively.

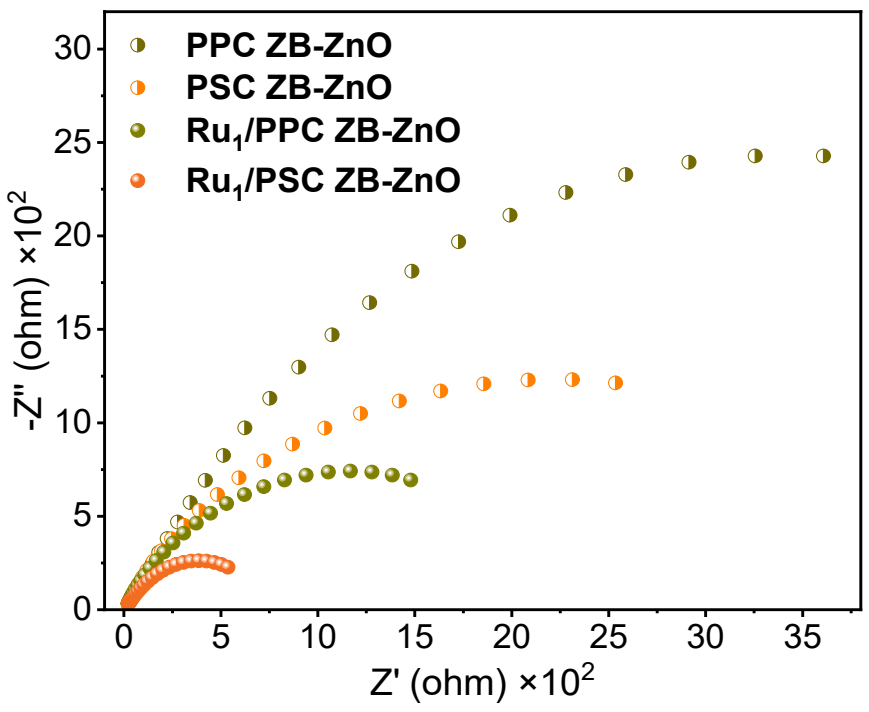

Supplementary Figure S42. Electrochemical impedance spectroscopy (EIS) of single-crystal and polycrystalline ZnO with and without Ru single-atom modification. EIS were performed in a 0.1 M Na_2SO_4.aqueous. The analysis demonstrates that that single-crystal ZnO (PSC ZB-ZnO) possesses a substantially lower charge-transfer resistance than its polycrystalline counterparts (PPS ZB-ZnO).

Comment 3: The mechanism chain for the selective formation of CH_3OOH needs to be refined: DRIFTS detected $*CH_3$ and *OOH, and it was speculated that they couple to form CH_3OOH. Although this is reasonable, it lacks direct evidence. Suggestion: By conducting isotope labeling experiments (such as with $^{18}O_2$) and combining with mass spectrometry to detect ^{18}O in the products, it can be clearly confirmed that the oxygen atoms in CH_3OOH originate from gaseous O_2 rather than lattice oxygen or water, thereby strongly supporting the proposed reaction pathway involving *OOH.

Response: We appreciate your insightful suggestion to strengthen the mechanistic evidence for CH_3OOH formation. To definitively trace the origin of oxygen in CH_3OOH, we conducted isotope experiments using ^{18}O-labeled oxygen ($^{18}O_2$) or ^{18}O-labeled water ($H_2{}^{18}O$) in the reaction and analyzed the oxygenated products by gas chromatography-mass spectrometry (GC-MS). To prevent CH_3OOH thermal decomposition during GC-MS analysis under aerobic conditions, we reduced CH_3OOH to CH_3OH with $NaBH_4$ before analysis. As shown in **Fig. 6f**, the use of $^{18}O_2$ and $H_2{}^{16}O$ resulted in strong signal peaks for $CH_3{}^{18}OH$, whereas no $CH_3{}^{18}OH$ was detected in reactions carried out with $^{16}O_2$ and $H_2{}^{18}O$. This provides strong evidence that O_2 is the source of oxygen for CH_3OOH. Moreover, control experiments

conducted in the absence of O_2 yielded no product formation, which conclusively rules out any contribution from lattice oxygen (**Supplementary Fig. S34**).

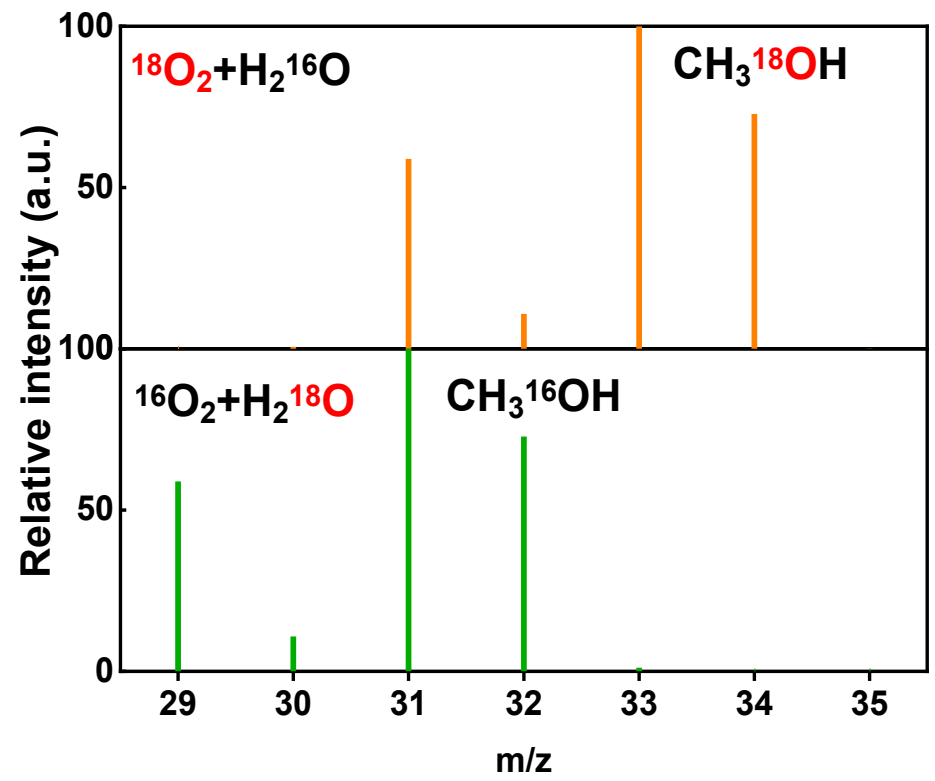

Fig. 6f. GC-MS spectra of CH_3OH generated over Ru_1/PSC ZB-ZnO with $^{18}O_2 + H_2{}^{16}O$ or $^{16}O_2 + H_2{}^{18}O$ in photocatalytic CH_4 oxidation.

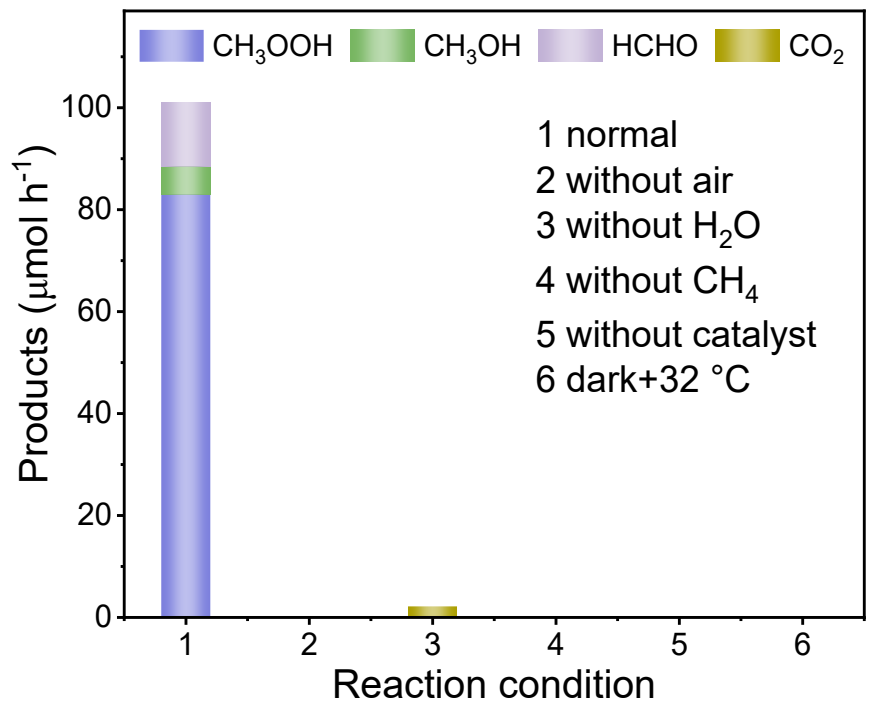

Supplementary Figure S34. Control experiments screening the necessity of key reaction components. Production rates obtained over Ru_1/PSC ZB-ZnO under different conditions. Reaction conditions: 45 mL min^{-1} CH_4 and 5 mL min^{-1} air (25 vol.% O_2/N_2), RH = 20%, 50 °C, 300 W Xe lamp with AM 1.5G filter, 1000 mW cm^{-2}. The results demonstrate that significant product formation occurs exclusively under light irradiation with all reaction components present. The negligible activity observed in the dark (even with system temperature rise to 32 °C), under visible light, in the absence of methane, or without catalyst, systematically rules out thermal effects. O_2 and H_2O is essential for initiating methane oxidation.

Comment 4: Figure 5e indicates that 0.6 wt% is the optimal loading amount; a higher loading will lead to the formation of nanoparticles. It is suggested that HAADF-STEM characterization be conducted on samples with 1-3 wt% loading to visually demonstrate the transition from single atoms to nanoparticles.

Response: Thank you for this valuable suggestion to visually demonstrate the transition from single atoms to nanoparticles at higher Ru loadings. To address this, we have conducted HAADF-STEM characterization on PSC ZB-ZnO samples with 1.0 wt%, 2.0 wt%, and 3 wt% Ru loadings, with results now integrated into **Supplementary Figure S29**. As shown in the figures, Ru clusters become clearly visible at the 1.0 wt% loading. With the Ru loading further increased to 2.0 wt% and 3.0 wt%, the clusters grow distinctly larger and begin to exhibit structural features characteristic of nanoparticles, demonstrating a pronounced tendency toward nanoparticle formation with increased loading.

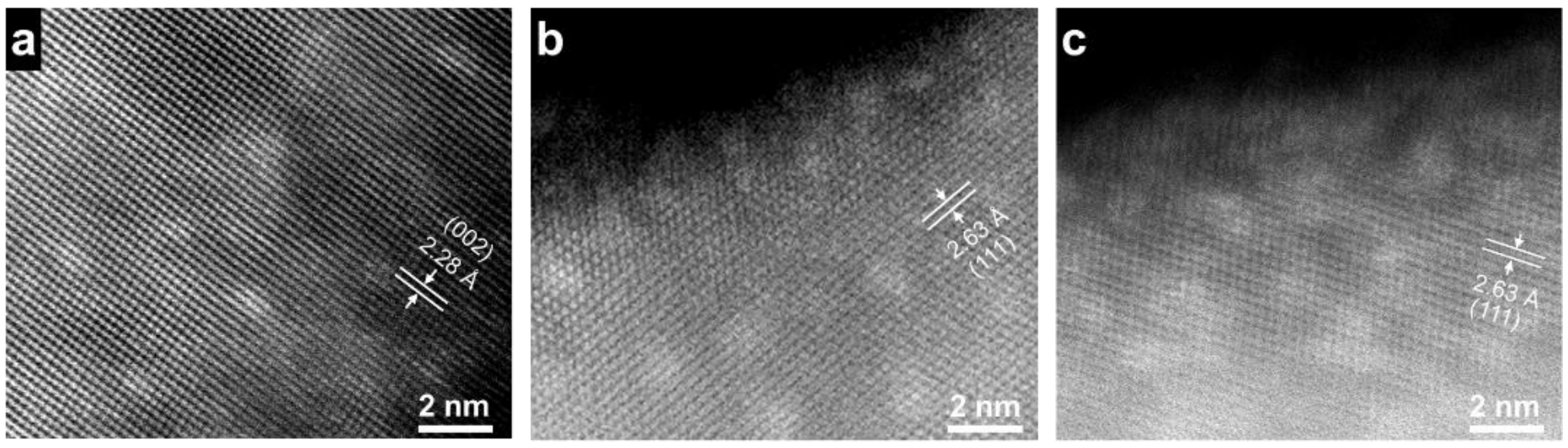

Supplementary Figure S29. HAADF-STEM characterization on PSC ZB-ZnO samples with (a)1.0 wt%, (b)2.0 wt%, and (c)3 wt% Ru loadings.

Comment 5: Although TRPL and TAS have demonstrated enhancement of interfacial charge separation, the oxidation ability of surface holes has not been directly evaluated. It is suggested to conduct additional hole capture experiments.

Response: We appreciate your astute suggestion to directly evaluate the oxidative capability of photogenerated holes. We have conducted complementary active species quenching experiments direct assessment of active species activity strengthens the mechanistic framework.

To further confirm the potential role of charge carriers and •OH species responsible for CH_4 oxidation, quenching experiments were conducted using $Na_2C_2O_4$ and isopropanol as scavengers of h^+ and •OH, respectively. As illustrated in Supplementary **Fig. S46**, when the h^+ scavenger is introduced, the reactions are nearly completely suppressed. When introducing isopropanol as •OH quencher, the yield of products decreases to ca. 45% of the original level, suggesting that •OH also plays an important role in the activation of methane, but photogenerated holes (h^+) play a dominant role in methane activation.

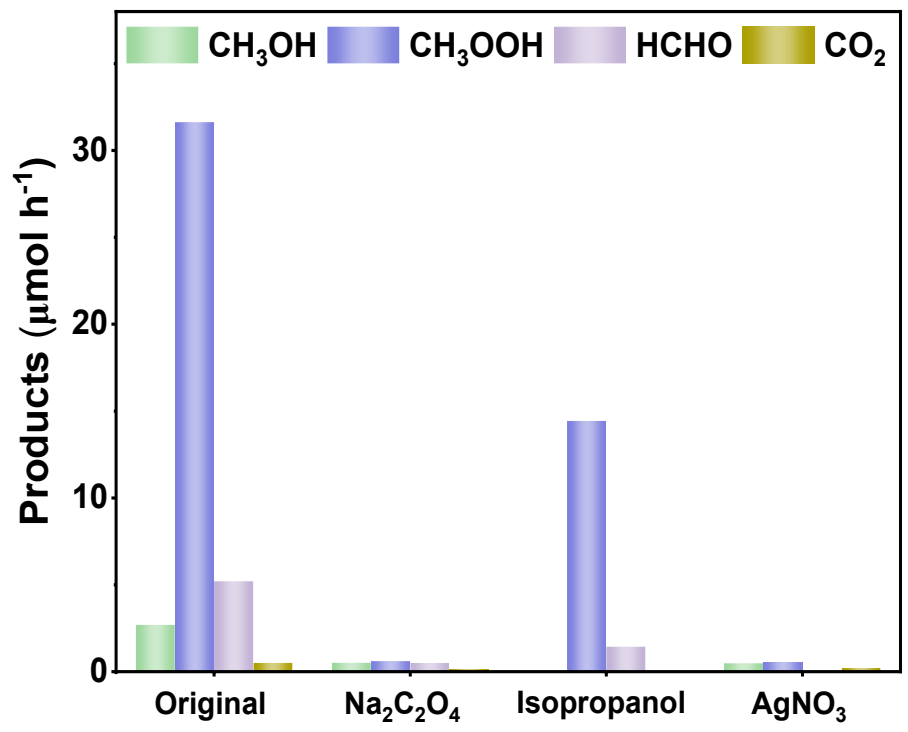

Supplementary Figure S46. Active species trapping experiments for photocatalytic CH_4 oxidation. Influence of the scavengers of the photocatalytic CH_4 oxidation over PSC Ru_1/ZB-ZnO. $Na_2C_2O_4$ as h^+ capture agent, isopropanol as ·OH capture agent, $AgNO_3$ as e^- capture agent. Batch reactor, 0.1 MPa air (25 vol.% O_2 and N_2), 1.9 MPa CH_4, 50 mL H_2O, 1 h reaction time.

Comment 6: Figure S8 has its title format messed up. Please adjust it.

Response: We sincerely thank you for pointing out the formatting issue. The title of the new **Supplementary Figure S12** has been corrected accordingly, as shown below:

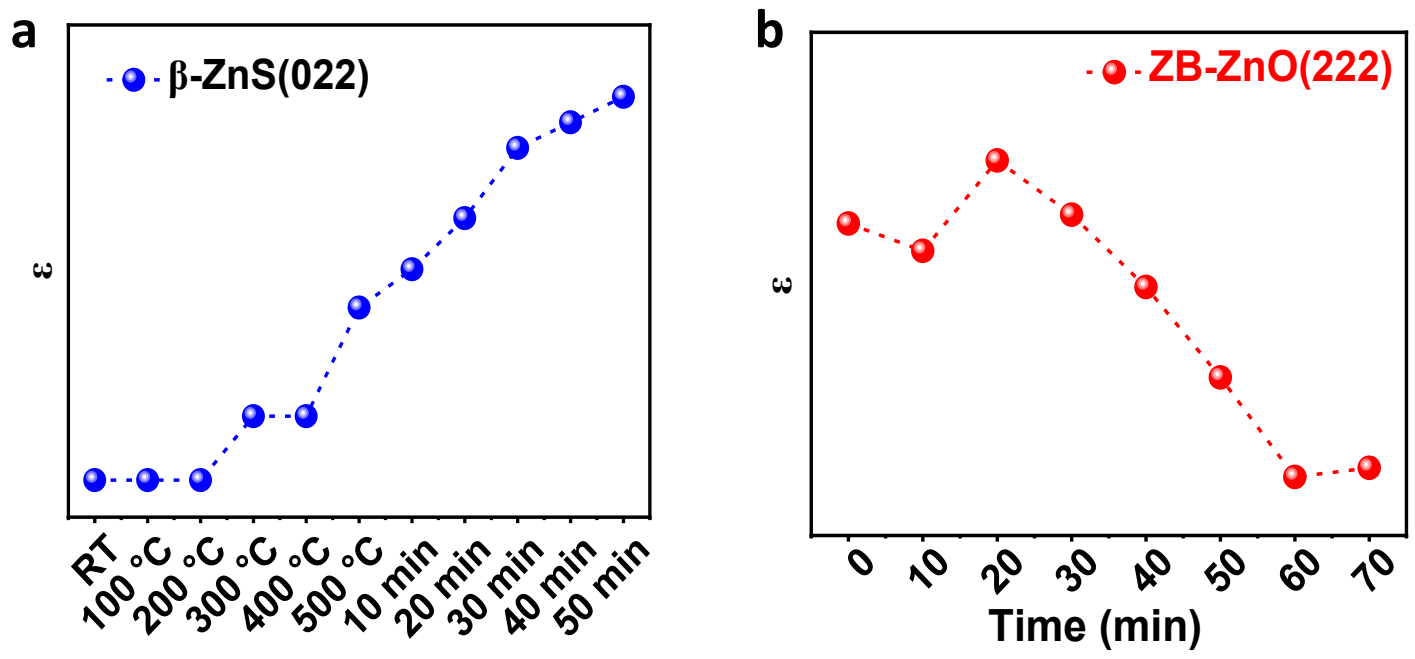

Supplementary Figure S12. Strain evolution during the growth of PSC ZB-ZnO on ZnS substrate.

Comment 7: The last sentence on page 1, "and achieve", has a grammatical error. It should be "and achieving". Please review the entire text.

Response: Thank you for identifying this grammatical inconsistency. As you pointed out, we have corrected "and achieve" to "and achieving" in the concluding sentence of the Introduction. Following your suggestion, we have conducted a thorough proofread of the manuscript and made necessary corrections for clarity and grammar.

Comment 8: In the photocatalysis test, the light source intensity "1000 mW·cm^{-2}" is extremely high. Is this considered as quasi-sunlight? Please confirm or explain whether this intensity has been calibrated with a filter.

Response: We sincerely appreciate your insightful comment regarding the light source intensity used in our photocatalysis experiments. In our experiment, the light source was a xenon lamp equipped with an AM 1.5G filter to simulate the solar spectral distribution. **Supplementary Figure S23** shows the xenon lamp light source spectral distribution with AM 1.5G filter. The power density was increased to 1000 mW·cm^{-2} and was carefully calibrated using a standard silicon photodetector power meter. The use of 1000 mW·cm^{-2} was a deliberate choice for our system, where photocatalytic reaction kinetics scale linearly with light intensity. This approach is well-established for accelerating performance evaluation. We acknowledge that describing this condition simply as "AM 1.5G" may be misleading, as it conflates spectral quality with intensity. We have amended the manuscript to accurately state, like "300 W Xe lamp with AM 1.5G filter, 1000 mW cm^{-2}."

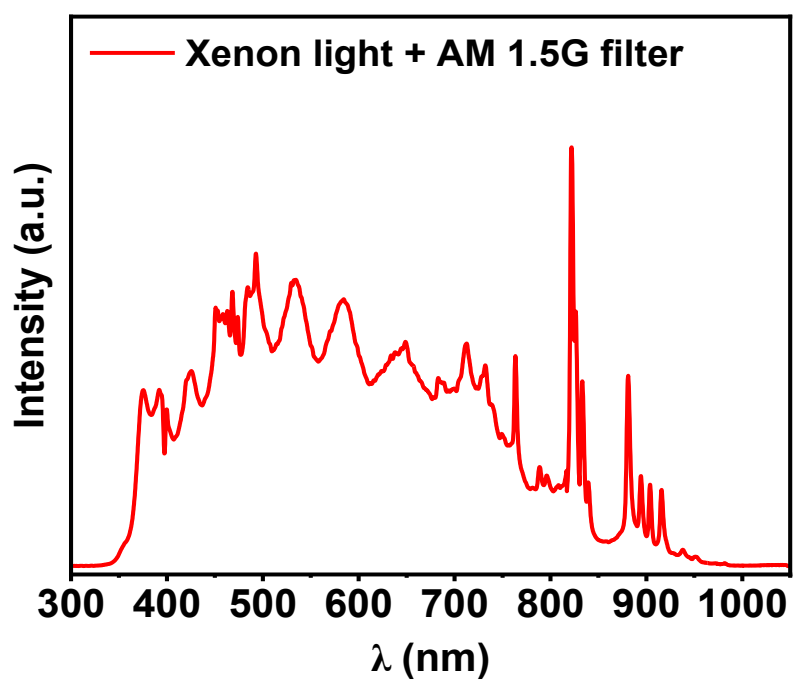

Supplementary Figure S23. The spectrum of Xenon lamp light with AM 1.5G filter.

Response to Reviewer #2

General comments: In this manuscript, the authors report a topotactic lattice contraction strategy to fabricate porous single-crystalline (PSC) zincblende-ZnO (ZB-ZnO) monoliths from β-ZnS. Using *in situ* transmission electron microscopy, they directly observed the dynamic formation of PSC ZB-ZnO at atomic resolution, unveiling preferential anion migration along {222} channels and strain-driven vacancy aggregation that leads to nanopore generation while maintaining single-crystalline order. Following the incorporation of atomically dispersed Ru sites, the resulted catalyst presents a 82% selectivity for methyl hydroperoxide (CH_3OOH) in methane oxidation under visible light (AM 1.5G), along with a good stability of 40 hours (<15% activity decay). This work shows an important strategy to synthesize single crystals with porosity, opening new frontiers in synthetic chemistry and material property engineering. The reviewer would suggest to accept it after addressing the following points:

Response: We sincerely appreciate your positive assessment of our work and the constructive suggestions. We are particularly encouraged by their acknowledgment that this work establishes a meaningful advancement not only for porous single-crystal synthesis but also for functional applications in catalysis and materials engineering, opening new frontiers for designing complex multifunctional crystalline architectures.

Comment 1: While XRD 2θ patterns alone cannot unambiguously confirm single crystallinity, complementary techniques such as Φ-scanning are critical. Cubic ZB-ZnO should exhibit four-fold symmetry (Figure 1a), yet the Φ-scan shows only two-fold periodicity (90° intervals, see in the Figure 1e). Please clarify whether this discrepancy arises from specific crystallographic orientation constraints or measurement methodology, and explain how to comprehensively verify the ZnO single crystallinity.

Response: We sincerely appreciate your insightful comment regarding the symmetry discrepancy observed in the Φ-scan analysis. You correctly note that cubic zinc blende ZnO should exhibit four-fold symmetry when rotating along the [001] axis and scanning the (220) plane (as schematically illustrated in **Fig. R1a**). However, in our study, the synthesized ZB-ZnO crystals were oriented along the (220) crystallographic plane. Consequently, the Φ-scan was performed with the [220] direction as the rotation axis while scanning the (001) reflection, which inherently displays two-fold symmetry due to the crystallographic constraints of this specific orientation (**Fig. R1b** and **Fig. 1e**). The observed symmetry is a direct consequence of the selected crystal orientation.

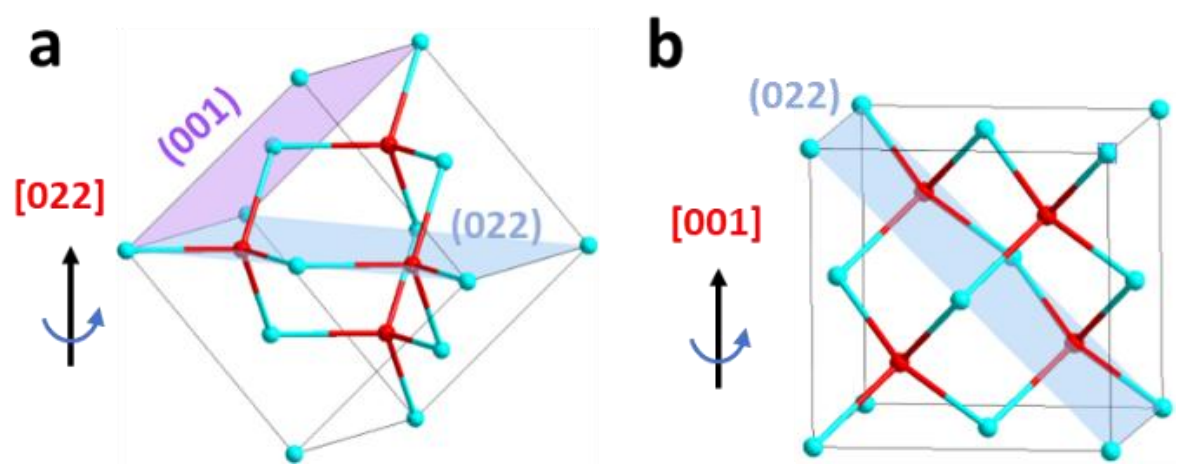

Fig. R1. Schematic diagram of ZB-ZnO rotation along the [022] and [001] axes.

To comprehensively validate the single crystallinity of ZB-ZnO, we employed complementary analytical techniques. First, XRD analysis (**Fig. 1c**) showed a single-crystal diffraction peak corresponding to the ZnO (022) plane. Building on this, rocking curve analysis (**Fig. 1d**) demonstrated

high crystallographic coherence with a narrow FWHM of 0.236°, while XRD φ-scan mapping (**Fig. 1e**) further confirmed the three-dimensional symmetry characteristic of single crystals. In parallel, Laue microdiffraction (**Fig. 1f**) revealed sharp, well-defined diffraction spots without polycrystalline signatures. Additionally, selected-area electron diffraction (SAED) patterns obtained from multiple regions along the [022] zone axis (**Supplementary Fig. S3**) consistently exhibited single-crystalline diffraction features. Critically, these cross-validated experimental results collectively provide definitive structural evidence that single-crystalline integrity is preserved throughout the porous architecture.

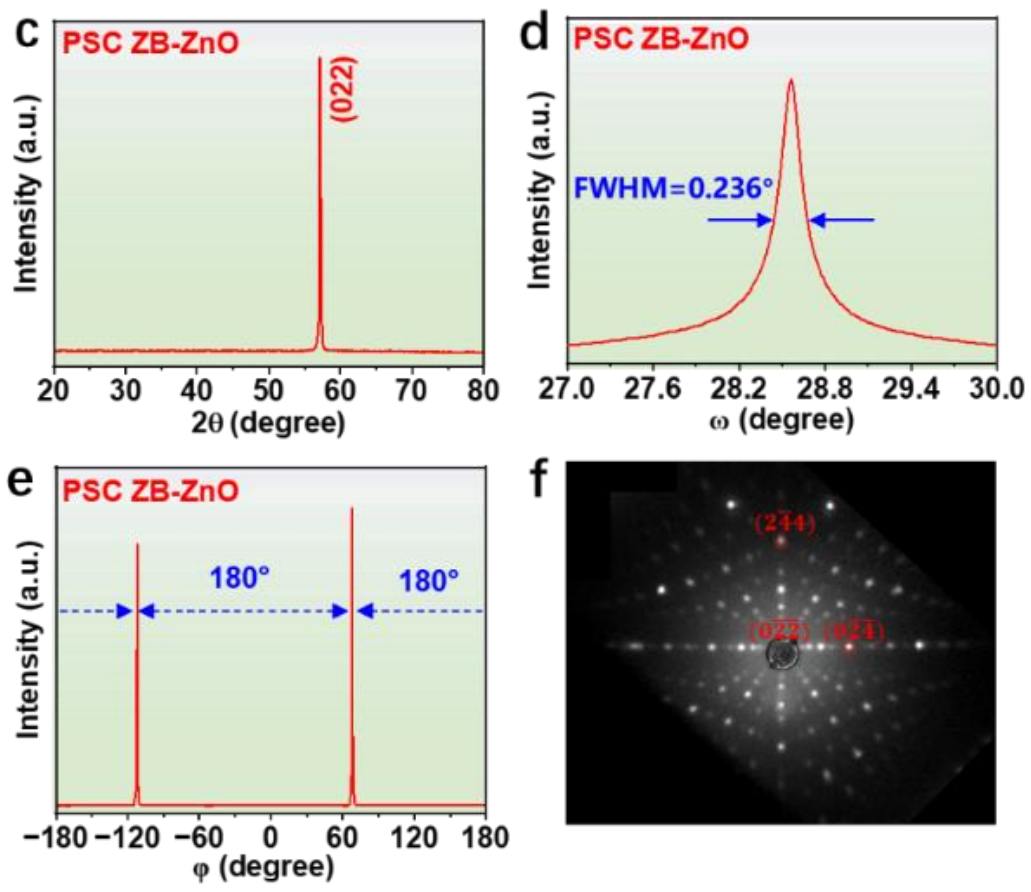

Fig. 1. **c,** XRD patterns of PSC BZ-ZnO. **d,** High-resolution rocking curve of PSC ZB-ZnO (022) reflection with full-width at half-maximum (FWHM) of 0.236°, demonstrating exceptional crystallographic coherence. **e,** XRD phi scan of (001) planes on a sample rotating around its [022] axis shows peaks spaced 180° apart. **f,** Laue microdiffraction pattern revealing single-crystalline nature.

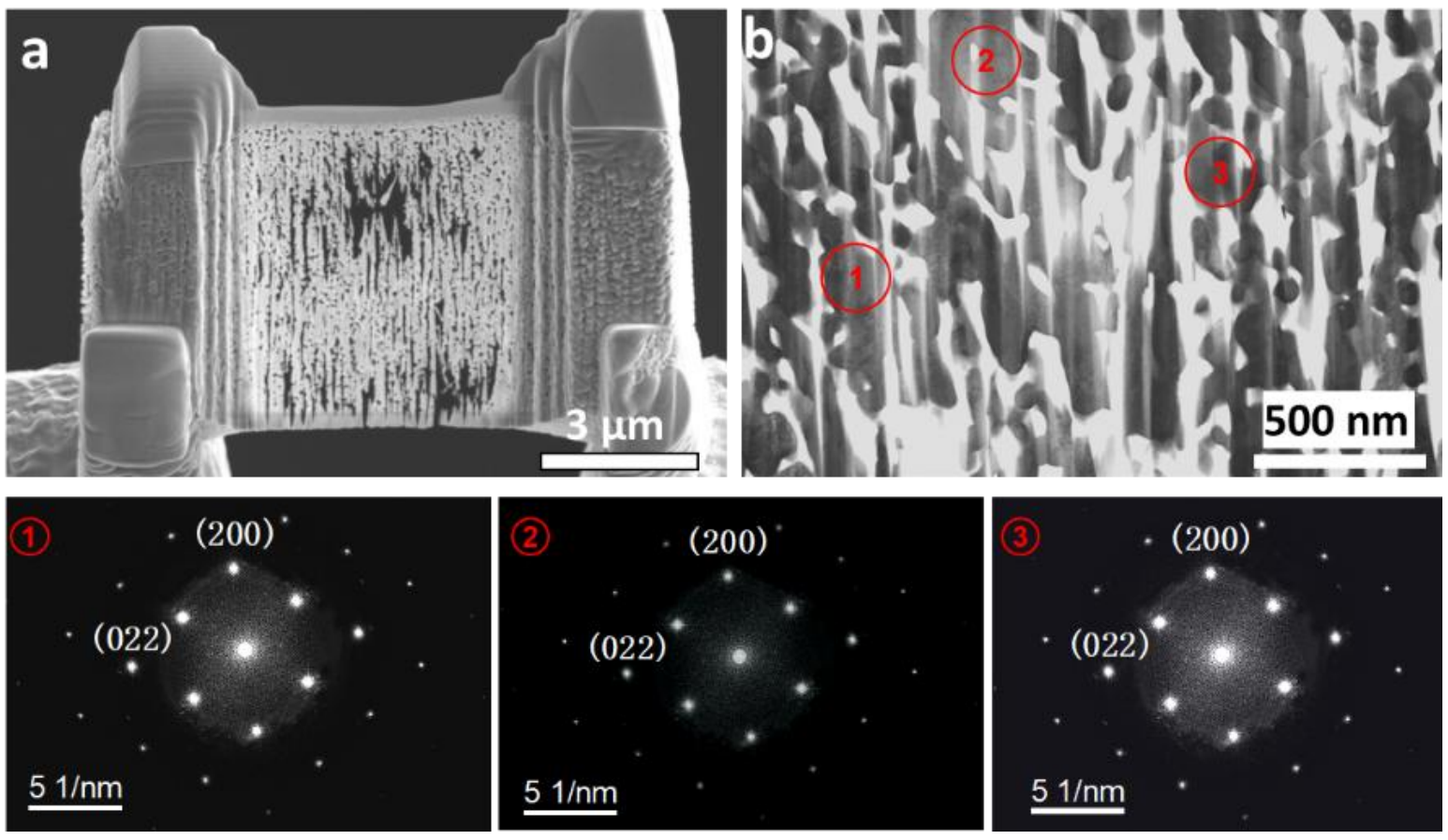

Figure S3. Microstructural characterization of ZB-ZnO PSCs. (a) FIB-sectioned lamella prepared for TEM analysis. (b) STEM image revealing three-dimensional porosity. (1-3) SAED patterns acquired from distinct regions (circled in b), indexed to $[02\bar{2}]$ zone axis, with (200) and (022) reflections labeled.

Comment 2: Hexagonal wurtzite is the thermodynamically stable phase of ZnO. Why does a cubic phase, instead of hexagonal, form when ZnS is oxidized? Is sulfur completely removed after the phase transformation? In the manuscript, the transition temperature from β-ZnS to cubic ZnO was recorded at 500°C. Would a higher temperature trigger structural reorganization?

Response: We sincerely appreciate your insightful comments regarding the phase and transformation

mechanism in our study. We appreciate your expertise and have addressed your questions below with supporting experimental evidence from our manuscript and supplementary data.

(a) Kinetics-driven topotactic transformation to cube PSC ZnO: Based on the comprehensive in situ TEM characterization and multiscale analysis presented in our work, the formation of the cubic zinc-blende ZnO phase instead of the thermodynamically stable hexagonal wurtzite phase during the oxidation of β-ZnS is attributed to a kinetics-driven topotactic transformation mechanism that preserves the parent cubic lattice registry. Our real-time atomic-scale observations reveal that the anion exchange ($S^{2-} \rightarrow O^{2-}$) preferentially proceeds along the {222} crystallographic planes of β-ZnS, which serve as fast diffusion channels, enabling epitaxial nucleation of ZB-ZnO while maintaining the coherence of the Zn^{2+} cation sublattice. This lattice inheritance, coupled with the anisotropic diffusion and the strain-induced vacancy coalescence, directs the phase evolution toward the cubic structure rather than allowing reconstruction into the hexagonal phase, demonstrating that under our controlled oxidation conditions, the kinetics of the topotactic transformation override thermodynamic stability, resulting in a cubic ZnO phase with exceptional crystallinity and interconnected porosity, which is pivotal for achieving the enhanced charge separation and photocatalytic performance highlighted in our study.

(2) Complete Sulfur Removal and Phase Purity: EDS mapping (**Supplementary Fig. S2**) reveals no residual sulfur signal in the final PSC ZnO, while XRD Rietveld refinement (**Supplementary Fig. S14**) identifies exclusively the cubic ZnO phase, with no detectable signatures of ZnS. Together, these results consistently confirm the complete removal of sulfur and the phase-pure conversion, supporting the effectiveness and reliability of the topotactic transformation process.

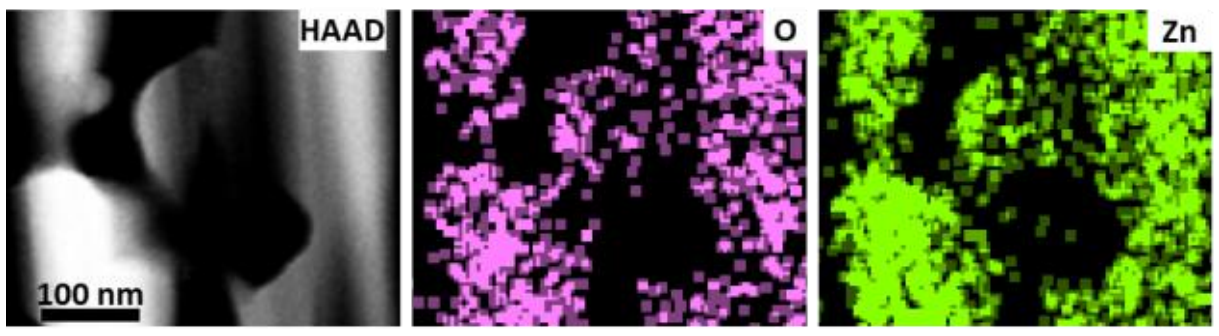

Figure S4. EDS mapping of the PSC ZB-ZnO. No residual S signals are detected.

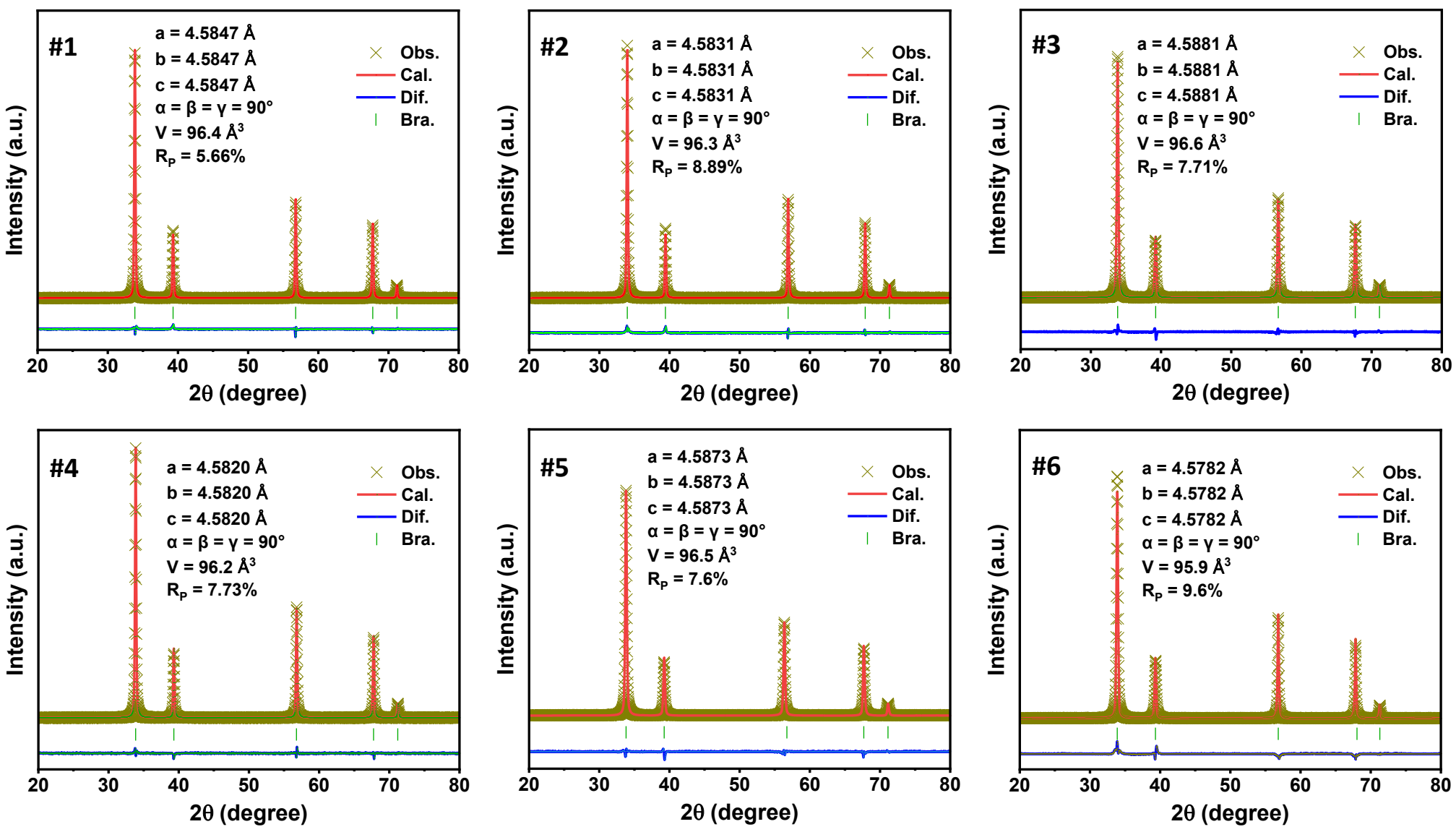

Figure S7. XRD rietveld refinement plots of different samples from six independent batches under

identical conditions.

Table S1. Summary of the results obtained from rietveld XRD studies: lattice parameters (a, b, c), unit cell volume (V), and R_p factor.

Sample	a (Å)	b (Å)	c (Å)	V (Å^3)	R_p (%)	Phase
#1	4.5847	4.5847	4.5847	96.4	5.66	Cubic
#2	4.5831	4.5847	4.5847	96.3	8.89	Cubic
#3	4.5881	4.5881	4.5881	96.6	7.71	Cubic
#4	4.5820	4.5820	4.5820	96.2	7.73	Cubic
#5	4.5873	4.5873	4.5873	96.5	7.60	Cubic
#6	4.5782	4.5782	4.5782	95.9	9.60	Cubic

(3) High-Temperature Effects: We agree that elevated temperatures can induce structural transformations. Our initial observations do indicate a trend towards the hexagonal wurtzite phase at higher temperatures (e.g., 800–900 °C). The detailed mechanism and kinetics of this thermally-driven phase reorganization represent a significant and complex direction for our future work.

Comment 3: Figure 4 illustrates the topological transformation process from β-ZnS to PSC ZB-ZnO observed via in situ high-resolution transmission electron microscopy (in situ HRTEM). The temporal resolution of this phase evolution depends on the spatial statistical distribution of the Zn–Zn atomic spacing along the ⟨112⟩ crystallographic direction. How is the spatial distribution of this atomic spacing precisely statistically analyzed?

Response: Thank you for this insightful comment regarding the quantitative analysis of Zn-Zn atomic spacing distributions presented in **Fig. 4** This analysis is indeed crucial for mapping the spatiotemporal evolution of the phase transformation at the atomic scale.

To achieve precise and statistically robust quantification of the Zn-Zn atomic spacing distribution along the ⟨112⟩ direction across the in situ HRTEM image sequences, we employed the specialized software tool CalAtom. CalAtom is specifically designed for the quantitative analysis of atomic columns in (scanning) transmission electron microscopy ((S)TEM) images at atomic resolution. As detailed in the dedicated methodology paper "CalAtom: A software for quantitatively analysing atomic columns in a transmission electron microscope image" (Ultramicroscopy, 202, 2019, 114-120) This software enables the accurate extraction of critical parameters, including atomic column positions, shapes, local atomic environments, and elemental composition information.

In our study, the application of CalAtom followed a rigorous workflow: HRTEM frames underwent noise reduction and contrast optimization, after which template-matching algorithms identified Zn atomic columns with high fidelity. Sub-pixel centroid positioning enabled calculation of Zn-Zn spacings across the field of view, generating spatial maps and contour plots at each timestep. Statistical analysis (histograms, mean/σ) revealed temporal-spatial evolution of spacings from β-ZnS to ZB-ZnO. Results were validated against lattice constants, FFTs, and SAED patterns.

Therefore, the spatial distribution of the Zn-Zn ⟨112⟩ atomic spacing presented in Figure 4 is the result of a rigorous, software-assisted quantitative analysis. CalAtom enabled us to move beyond qualitative

observation, providing statistically significant data on the nanoscale distribution of phases (ZnS vs. ZB-ZnO) and strain fields based on precise atomic position measurements. This approach is essential for resolving the complex kinetics of the topotactic transformation.

Comment 4: Based on Figure 5e, it can be observed that the loading amount of Ru significantly impacts the catalytic performance. The selectivity for methyl hydroperoxide initially increases then decreases with increasing Ru loading levels. What causes this trend? Is the structural evolution of Ru species the primary factor? Will the porous structure change under different loading amounts?

Response: Thank you for your insightful observation regarding the relationship between Ru loading levels and catalytic performance. The observed trend—where methyl hydroperoxide (CH_3OOH) selectivity initially increases (0–0.6 wt% Ru) then declines (0.6–3 wt% Ru)—is indeed primarily governed by the structural evolution of Ru species from atomically dispersed sites to nanoparticles, which directly modulates the oxygen activation pathway.

At the optimal loading of 0.6 wt%, aberration-corrected HAADF-STEM and EXAFS confirm Ru exists as isolated single atoms with Ru-O_4 coordination. This structure stabilizes end-on O_2 adsorption, facilitating selective reduction to •OOH intermediates and thus achieving high CH_3OOH selectivity. In contrast, at higher loadings (1–3 wt%), Ru aggregates into nanoclusters or nanoparticles, as evidenced by HAADF-STEM (**Supplementary Figure S29**). This may promote side-on O_2 adsorption and favors complete O-O bond cleavage, thereby shifting the product distribution toward methanol and formaldehyde. (literature support: Y.H. J. et al., J. Am. Chem. Soc. 2023, 145, 2698-2707).

Importantly, this selectivity trend is driven exclusively by Ru speciation, not by changes in the ZnO support. The porous architecture of PSC ZB-ZnO remains invariant across all loadings, as confirmed by identical BET surface from N_2 physisorption (**Supplementary Table S3**).

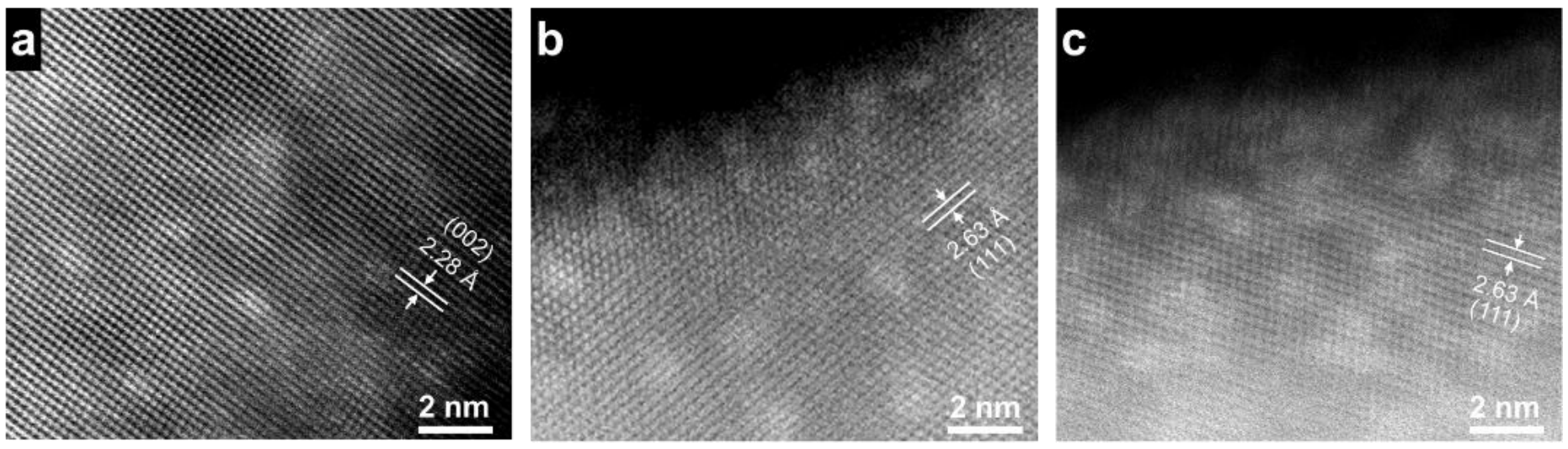

Figure S29. HAADF-STEM characterization on PSC ZB-ZnO samples with (a)1.0 wt%, (b)2.0 wt%, and (c)3 wt% Ru loadings.

Table S3. Physicochemical properties of ZnO-based catalysts

Catalysts	BET surface area (m^2/g)	Ru loading（wt%)
PSC ZB-ZnO	16.1	0
PPC ZB-ZnO	16.6	0
Ru_1/PSC ZB-ZnO	15.6	0.30
Ru_1/PPC ZB-ZnO	15.7	0.32

Comment 5: Figure 5 presents the catalyst's exceptional performance in methane oxidation to methyl hydroperoxide, with an extremely high stability. Suggest authors to discuss this merit with detailed

information. In addition, could the porous single-crystal framework inhibit Ru leaching?

Response: Thank you for highlighting the exceptional performance and stability of our Ru_1/ZB-ZnO catalyst. The outstanding selectivity (82.5%) for methyl hydroperoxide (CH_3OOH) and remarkable stability (>85% activity retention over 40 h) originate from the synergistic interplay between the robust porous single-crystalline (PSC) ZnO framework and the atomically dispersed, strongly anchored Ru sites.

First, the PSC architecture synthesized via topotactic contraction preserves long-range crystallographic coherence without grain boundaries. This eliminates defect-induced recombination centers and structural degradation pathways, while the interconnected pores ensure efficient mass transport, preventing pore blockage and reactant/product accumulation that typically deactivate catalysts.

Second, the atomic dispersion of Ru is crucial. Ru single atoms are stabilized via Ru–O coordination (Ru-O = 2.006 Å, EXAFS) within the ZnO lattice. This configuration stabilizes Ru^{4+} sites against dissociation or agglomeration, as confirmed by post-reaction HAADF-STEM (**Supplementary Figure S32**). Under reaction conditions, Ru acts as an electron reservoir, maintaining a stable oxidation state (~+3.5, XANES) and facilitating reversible charge transfer (evidenced by in situ XPS shift), kinetically disfavoring dissolution. This configuration directs selective O_2 reduction to •OOH (evidenced by EPR, isotope experiments), suppressing non-selective overoxidation pathways, while also enhancing charge separation (prolonged carrier lifetime). Together, the rigid single-crystal matrix provides structural stability, and the firmly integrated Ru sites ensure catalytic integrity, collectively explaining the high selectivity and long-term stability observed.

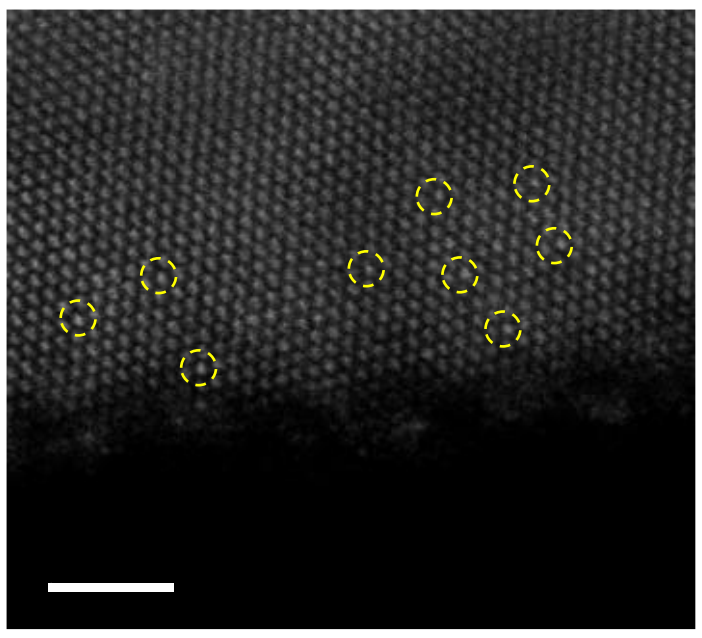

Figure S32. Post-reaction atomic-scale characterization of Ru_1/ZB-ZnO. HAADF-STEM confirm persistent single-atom dispersion of Ru (yellow circles) on ZnO after catalytic reaction. No Ru clusters/nanoparticles observed.

Response to Reviewer #3

General comments: The authors successfully transformed β-ZnS into porous single-crystal zinc blende ZnO (ZB-ZnO) under an O_2/Ar atmosphere and subsequently loaded Ru to enable selective photocatalytic oxidation of methane, a process with considerable practical application potential. Crystal structure characterization techniques, including ex-situ XRD, Laue microdiffraction, selected area electron diffraction, and in-situ high-resolution transmission electron microscopy, were employed to elucidate the lattice reorganization mechanism during the solid-state phase transition. Furthermore, the study investigates the reaction mechanism of methane oxidation on Ru-loaded ZB-ZnO. This manuscript presents notable innovation and promising applications in solid material design. Nevertheless, it requires

further strengthening in establishing clear structure-activity relationships, enhancing data depth, and improving the logical coherence of the narrative, to meet the publication standards of high-impact journals. The following are the review comments.

Response: Thank you for your thorough evaluation of our manuscript and for your positive feedback on the innovative aspects and application potential of our work. We sincerely appreciate the time and effort you dedicated to the review, as well as your constructive suggestions, which have helped us significantly improve the scientific rigor and impact of the study. Below, we detail our modifications to address these points comprehensively.

Comment 1: This manuscript suffers from structural flaws and an absence of thematic coherence. While the initial sections focus extensively on the synthesis mechanism of porous single-crystalline ZB-ZnO, the subsequent discussion fails to adequately elucidate its role in determining photocatalytic performance and underlying reaction mechanisms. Furthermore, the introduction fails to establish a logical connection between the scientific background and the functional significance of the material. The primary objective of the study requires clarification: is the work intended to present a novel synthetic approach, an advancement in mechanistic understanding, or a significant improvement in catalytic performance?

Response: We sincerely thank you for careful reading and constructive comments, which are invaluable for strengthening our manuscript. In the revision, we have therefore reorganized the manuscript around a single overarching objective: to demonstrate that a rationally engineered porous single-crystalline semiconductor can fundamentally overcome charge-recombination bottlenecks in photocatalysis and, when coupled with atomically dispersed Ru sites, can direct oxygen activation toward highly selective methane photo-oxidation to CH_3OOH.

(1) Clarified the primary objective and thematic logic: The Introduction now builds a logical progression from (i) the universal bottleneck of charge recombination in photocatalysis, to (ii) the conceptual advantage of PSC materials—combining a **low-defect, single-crystalline matrix** for superior charge transport with a 3D porous network for surface site engineering and reactant diffusion, to (iii) the specific synthetic challenge of achieving such architectures. The final paragraph explicitly states our work's goal: to develop a topotactic contraction strategy for PSCs, atomically decipher the pore-formation mechanism, demonstrate the intrinsic charge-transport benefits of the single-crystalline ZnO framework, and functionally validate the platform via Ru-site-engineered selective methane photo-oxidation to CH_3OOH production.

(2) Strengthened the link between structure and function: The revised manuscript elucidates the role of PSC ZB-ZnO by introducing direct comparisons with porous polycrystalline controls (PPC ZB-ZnO). Key evidence includes prolonged carrier lifetimes (TRPL: 17.0 ns for PSC vs. 12.4 ns for PPC), reduced charge-transfer resistance (EIS), and enhanced radical generation (EPR) due to grain-boundary elimination. These structural advantages—exceptional crystallinity (0.236° FWHM) and interconnected porosity—are explicitly linked to superior photocatalytic performance: higher CH_3OOH yield (84.6 $\mu mol \cdot h^{-1}$) and selectivity (82.5%). The strengthened structure-function correlation is further supported by in situ mechanistic studies showing efficient charge transport and tailored reaction pathways.

(3) Refined the balance between synthesis, mechanism, and application: The revised text presents

the topotactic lattice contraction as a **synthesis strategy** for PSCs. The in situ mechanistic studies provide **fundamental design rules** for preserving crystallinity during porogenesis. The photocatalytic methane oxidation serves as a **stringent functional test** that simultaneously validates the charge-transport advantage of the single-crystal framework and the product-selectivity control enabled by pore-hosted single-atom sites.

Comment 2: The manuscript describes lattice reorganization but lacks dynamic visualization of phase evolution. Include operando or time-resolved imaging, or 3D tomography (e.g., FIB-SEM) to show pore connectivity and crystallographic consistency.

Response: Thank you for this insightful suggestion regarding the visualization of dynamic phase evolution. We agree that comprehensive structural characterization is crucial, and we have indeed provided substantial operando, time-resolved, and 3D data to address precisely these aspects.

First, as noted by the reviewer, the main text details the pore formation mechanism via in situ anion exchange along {222} diffusion channels and strain-driven vacancy coalescence. This process is directly visualized in **Supplementary Figure S10**, which presents a sequential in situ TEM image series acquired during thermal treatment under O_2 from 400°C to 520°C. This series captures the key stages: initial surface stability, pore nucleation at 450°C, propagation and coalescence into an interconnected network by 500°C, and final formation of a fully percolated 3D porous architecture at 520 °C.

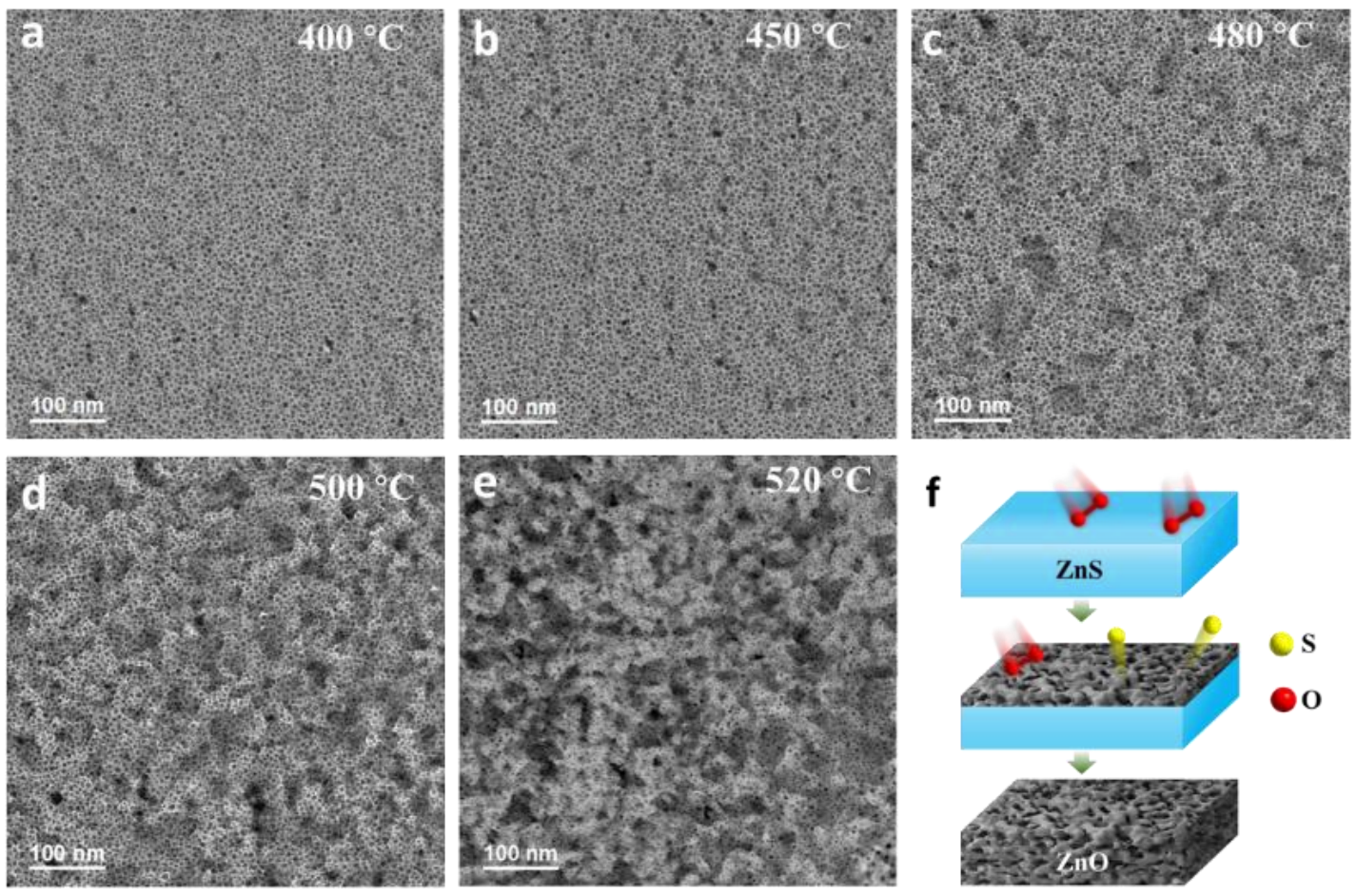

Figure S10. In situ TEM observation of pore evolution in ZB-ZnO under thermal treatment. (a-e) Sequential images showing pore formation in O_2 atmosphere during continuous heating (460→520 °C at 10 °C/min). (f) Schematic illustration of the formation of porous ZnO structures.

Second, to further solidify the dynamic visualization of the macroscopic phase transformation, we have now supplemented the manuscript with time-resolved FIB-SEM imaging (new **Supplementary Figure S11)**. These images track the in situ transformation at 500°C over 1, 3, and 5 minutes, clearly showing the progression of a sharp reaction front, the consumption of the ZnS precursor core, and the concurrent growth of the continuous porous ZnO framework, providing direct evidence of the transformation kinetics and interface movement.

Most importantly, to conclusively demonstrate the three-dimensional pore connectivity and crystallographic consistency of the final product—the porous single-crystalline (PSC) ZnO monolith—

we have performed 3D FIB-SEM tomography (new **Supplementary Figure S6**). The 3D reconstruction unambiguously reveals a continuous, coherent single-crystal skeleton permeated by an interconnected, hierarchical pore-throat network throughout the entire volume. Quantitative analysis of this network provides pore-size distribution and throat dimensions, which structurally rationalizes the enhanced mass transport properties. Therefore, the combination of atomic-scale in situ TEM, time-resolved cross-sectional FIB-SEM, and volumetric 3D FIB-SEM tomography collectively provides a multiscale, dynamic, and three-dimensional visualization of the phase evolution, pore formation, and the resulting structurally coherent porous architecture.

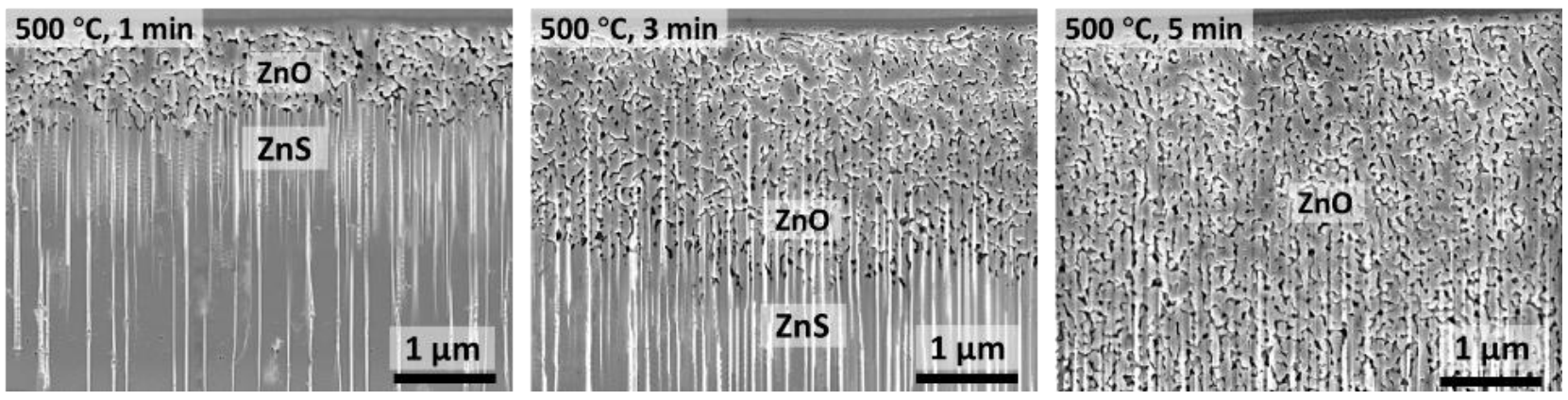

Figure S11. Time dependent FIB-SEM images of PSC ZB-ZnO grown on ZnS. At 1 min, a distinct interface between ZnS (precursor) and newly formed porous ZnO is observed, indicating the initiation of the topotactic lattice contraction process. By 3 minutes, the ZnS core has become partially consumed, and a continuous porous structure composed of ZnO continues to grow downward. At 5 minutes, the ZnS has almost completely transformed into PSC ZnO throughout the observed area.

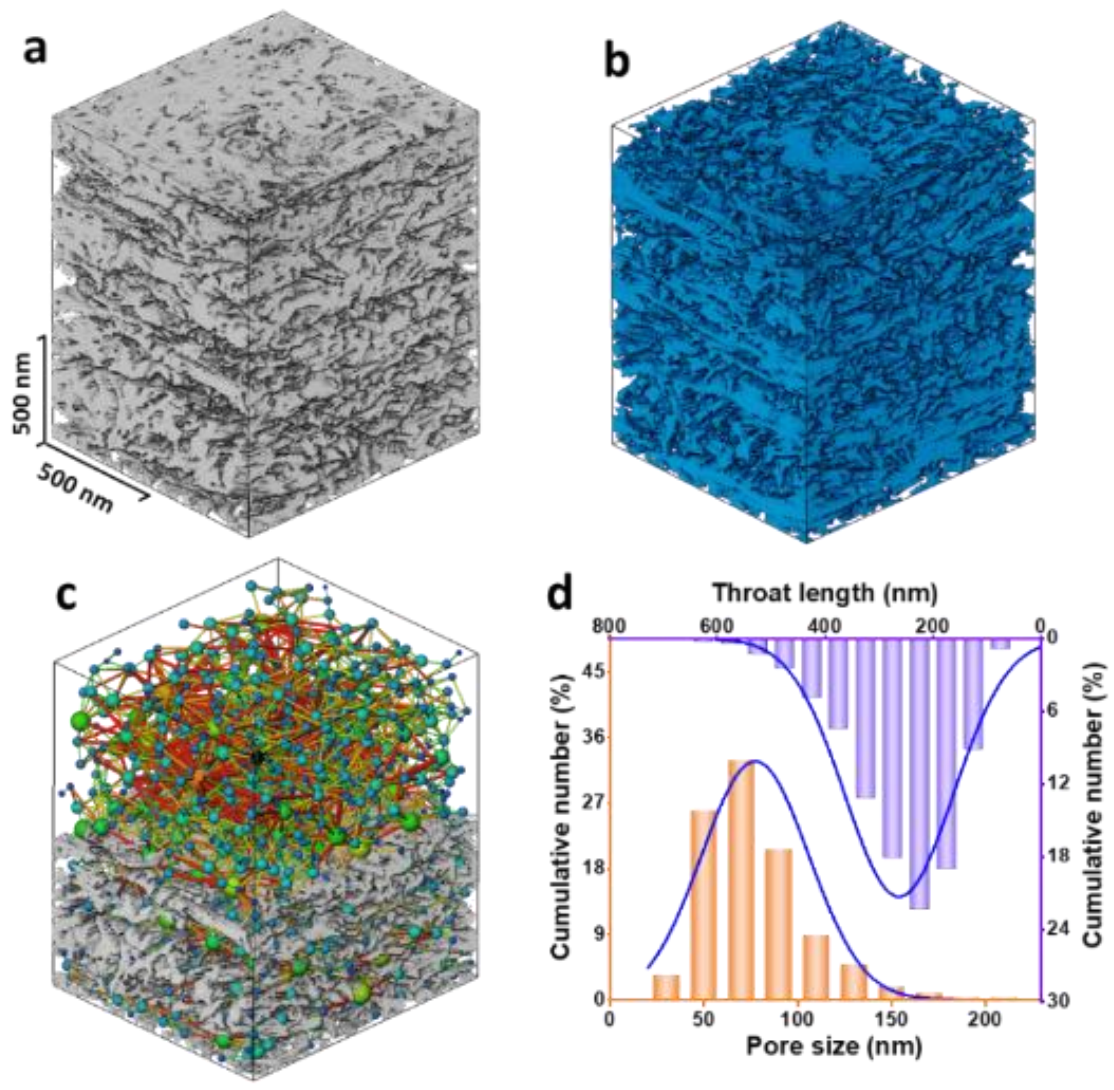

Figure S6. 3D reconstruction analysis of PSC ZnO: (a) skeletal framework structure, (b) pore system, (c) ball-stick model of interconnected pores and throats, (d) pore size and throat length distribution. The 3D reconstruction reveals the skeletal framework, continuous pore system, and interconnected pore-throat network. These structural features confirm a well-connected porous architecture, which is favorable for efficient mass transport.

Comment 3: What are the characteristics or advantages of ZnO compared to other types of photocatalytic materials? Why is ZnO chosen for photocatalytic methane oxidation? In addition, in the aspect of photocatalytic methane oxidation, what advantages do porous single-crystal ZB-ZnO or Ru/ZB-ZnO have over amorphous ZnO or Ru/amorphous ZnO? The key parameters - band structure, charge separation efficiency, charge transfer kinetics and surface/interface properties should be analyzed. Key

data - UV-Vis DRS (Tauc plot), flat band potential, EIS, work function and steady-state PL must be provided to evaluate the photoelectric response and electronic structure of the materials.

Response: We sincerely appreciate your insightful comments, which have greatly contributed to enhancing the quality of our manuscript.

(1) ZnO exhibits several distinct advantages over other photocatalytic materials, which align with the goals of our study on photocatalytic methane oxidation.

First, ZnO stands as the most widely employed photocatalysts for the photooxidation. It features a direct bandgap of approximately ~3.2 eV and exhibits high electron mobility, enabling efficient light absorption, generation and separation of charge carrier under UV and visible light irradiation. Importantly, the photocatalytic oxidation of hydrocarbon species relies critically on the formation of reactive oxygen species (e.g., $\cdot O_2^-$, ·OH or ·OOH radicals). This necessitates that a semiconductor possesses a conduction band minimum (CBM) more negative than the potential of $O_2/\cdot O_2^-$ (-0.33V versus NHE) a valence band maximum (VBM) more positive than the $H_2O/\cdot OH$ potential (+2.3 V versus NHE). ZnO satisfies both thermodynamic requirements, enabling effective activation of molecular oxygen and water to generate these essential oxidative intermediates. In contrast, other common photocatalytic materials such as CeO_2, CuO or $BiVO_4$ often struggle to simultaneously meet these stringent band-edge criteria, thereby limiting their efficacy in complete methane oxidation pathways. (see in the **Figure R1**, *Qi Li, et al, Angew. Chem. Int. Ed. 2022, 61, e202108069*).

PUBLISHED DATA FIGURE REDACTED

Figure R1. Illustration of the band structures of commonly used semiconductors and the redox potentials of diverse reactants. (Qi Li, et al, Angew. Chem. Int. Ed. 2022, 61, e202108069)

Second, ZnO serves as an excellent platform for constructing efficient heterojunctions and for metal co-catalyst loading, which further enhances its performance. The synergistic effects in these composites are multifaceted: the metal nanoparticles can act as electron sinks (or hole trap) to improve charge separation, their surface plasmon resonance can enhance light absorption into the visible range, and they can create new active sites at the metal-ZnO interface for specific reaction pathways. The ability of ZnO to form effective interfaces with both noble and non-precious metals greatly broadens its design flexibility and performance ceiling.

Third, ZnO is environmentally benign and structurally versatile, allowing for precise morphological control—as demonstrated in our work, where we engineered porous single-crystalline zinc blende ZnO (ZB-ZnO) via topotactic transformation from ZnS. This unique architecture integrates long-range

crystallinity with interconnected pores, mitigating grain-boundary recombination and enhancing mass transport—a limitation in polycrystalline. Our experimental results, as evidenced in **Figure S33**, demonstrates the high activity of our specifically designed PSC ZnO-based photocatalyst for methane oxidation, showcasing its practical efficacy. This performance is a direct consequence of the advantageous inherent properties of ZnO, which we have further optimized through Ru- modification strategies.

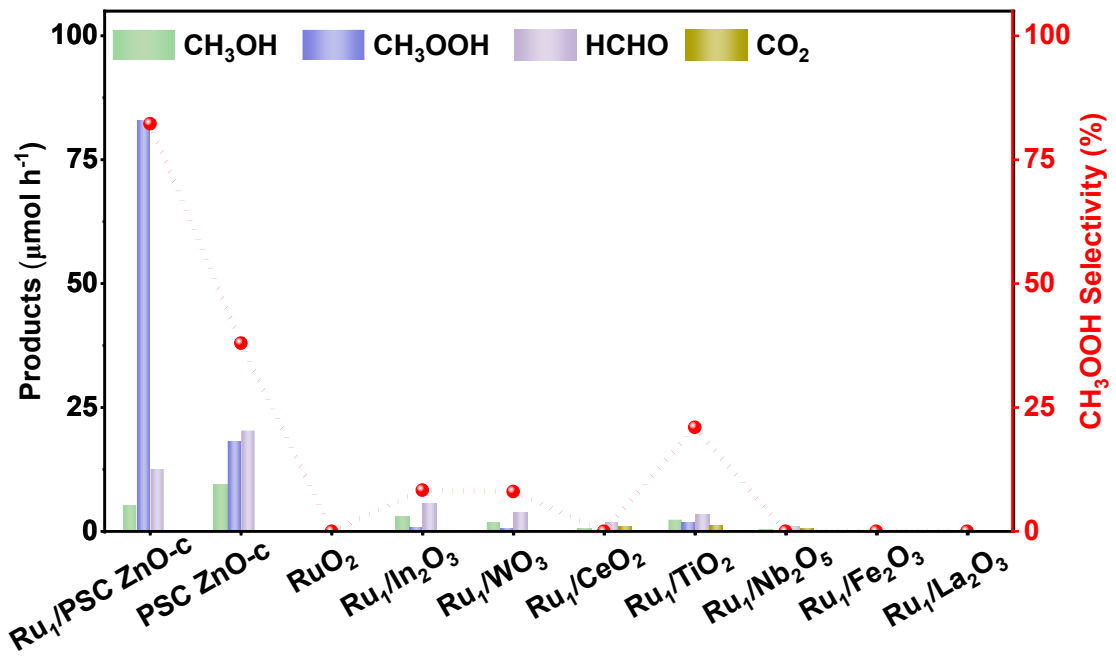

Figure S33. Performance evaluation of Ru_1/PSC ZnO and other Ru-based catalysts in the photocatalytic methane oxidation reaction.

(2) We fully acknowledge the importance of establishing a reasonable comparison system to highlight the intrinsic advantages of our porous single-crystalline ZB-ZnO (PSC ZB-ZnO), and we also agree that contrasting crystalline and amorphous phases is a significant and frontier topic in materials science. In the present study; however, our primary objective is to unambiguously elucidate the intrinsic role of long-range crystalline order in eliminating boundaries and reducing defects to enhance photocatalytic performance.

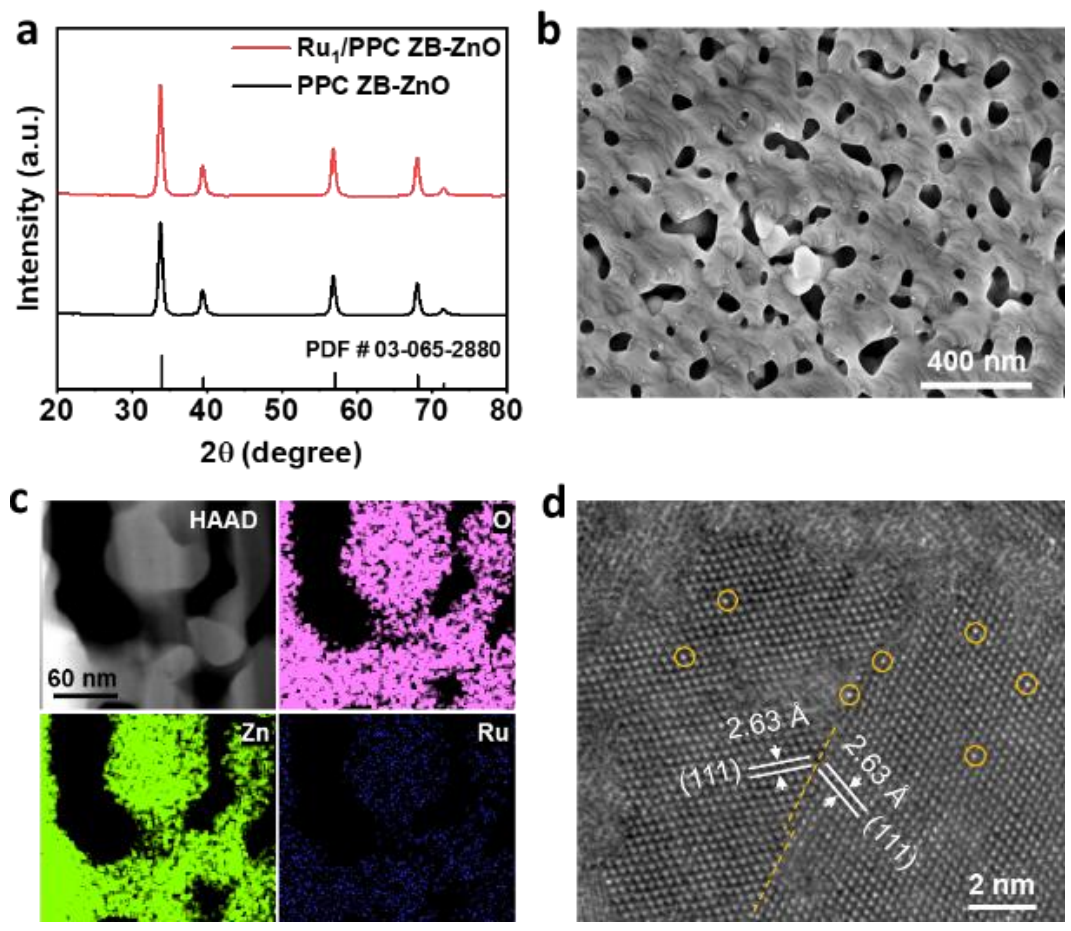

Figure S30. Structural and Morphological Characterization of Ru_1/PPC ZB-ZnO. (a) XRD patterns of Ru_1/PPC ZB-ZnO and PPC ZB-ZnO, where diffraction peaks are indexed to PDF card #03-065-2880, confirming the cubic phase of the as prepared material. (b) SEM image of Ru_1/PPC ZB-ZnO, exhibiting a porous architecture with uniform pore distribution. (c) Elemental mapping of Ru_1/PPC ZB-ZnO, illustrating the homogeneous dispersion of Ru, Zn, and O species throughout the porous matrix. (d) HAADF STEM Image of Ru_1/PPC ZB-ZnO.

For this reason, we deliberately selected porous polycrystalline ZB-ZnO (PPC ZB-ZnO) as the control

material, rather than amorphous ZnO. This choice was based on the need to maintain other structural and physicochemical parameters as similar as possible, thereby isolating the effect of crystallinity itself. As confirmed by the characterization data (**Supplementary Figure S30, Table S3**), PSC ZB-ZnO and PPC ZB-ZnO possess the same crystal phase, highly comparable pore architectures, and similar BET surface areas (15.6–16.6 m^2 g^{-1}), while Ru_1/PSC ZB-ZnO and Ru_1/PPC ZB-ZnO also exhibit nearly identical Ru loadings (0.32 vs 0.30 wt%). Such stringent control over morphology, porosity, surface area, and composition allows us to focus specifically on the impact of eliminating grain boundaries. In contrast, an amorphous ZnO reference would inevitably differ not only in long-range order but also in morphology, pore structure, defect density, and electronic properties, introducing multiple confounding factors and making it difficult to ascribe performance differences solely to crystallinity.

Table S3. Physicochemical properties of ZnO-based catalysts.

Catalysts	BET surface area (m^2/g)	Ru loading (wt%)
PSC ZB-ZnO	16.1	0
PPC ZB-ZnO	16.6	0
Ru_1/PSC ZB-ZnO	15.6	0.30
Ru_1/PPC ZB-ZnO	15.7	0.32

On this well-controlled basis, we systematically compared the photocatalytic methane oxidation performance and charge-carrier behaviors of PSC ZB-ZnO, PPC ZB-ZnO, and their Ru single-atom-modified counterparts (Ru_1/PSC ZB-ZnO and Ru_1/PPC ZB-ZnO). The photocatalytic results (**Fig. 5f**) clearly show that the porous single-crystalline structure (PSC ZB-ZnO, Ru_1/PSC ZB-ZnO) consistently outperforms the porous polycrystalline structure (PPC ZB-ZnO, Ru_1/PPC ZB-ZnO), and that Ru modification further enhances activity and significantly improves the selectivity toward CH_3OOH.

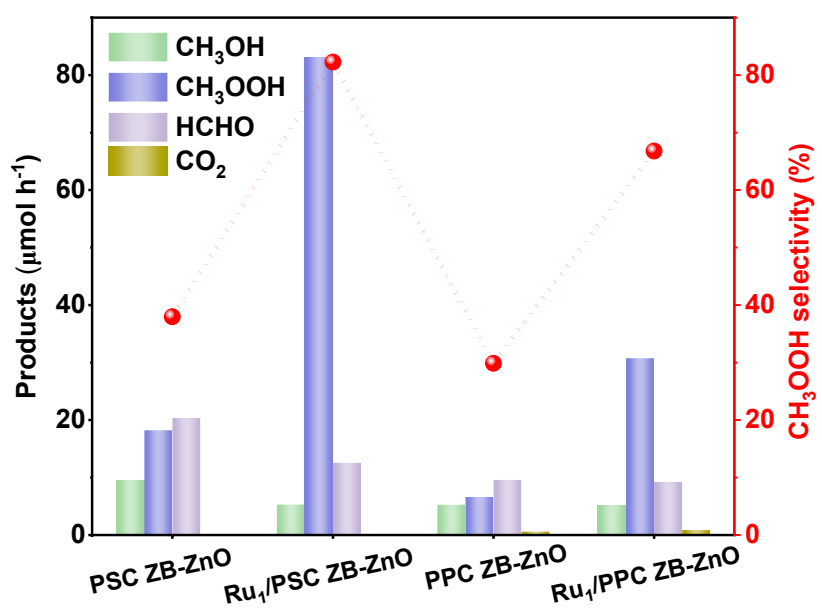

Fig. 5f. Photocatalytic methane oxidation performance of PSC ZB-ZnO, Ru_1/PSC ZB-ZnO, PPC ZB-ZnO, and Ru_1/PPC ZB-ZnO.

To elucidate the origin of these differences, we first examined the electronic structures. UV–vis diffuse reflectance spectra and Kubelka–Munk plots (**Supplementary Figure S35**) reveal that PSC ZB-ZnO and PPC ZB-ZnO have nearly identical bandgaps (3.16 vs 3.17 eV), indicating that the transition from polycrystalline to single-crystalline support does not substantially change the bandgap. Upon Ru incorporation, the bandgap is slightly narrowed (Ru_1/PSC ZB-ZnO: 3.13 eV; Ru_1/PPC ZB-ZnO: 3.14 eV), reflecting a marginal red-shift that is beneficial for visible-light absorption. Combined UPS, VB-XPS, and Mott–Schottky analyses (**Supplementary Figures S36–S38**) were used to construct the band

structure diagram (**Supplementary Figure S39**), showing that single-crystalline and polycrystalline ZnO possess similar band alignments, while Ru modification induces a small negative shift of both the valence band (VB) and conduction band (CB). This optimized band alignment in Ru-modified ZnO facilitates more efficient charge separation and provides stronger redox driving forces. Importantly, based on the VB/CB positions and the thermodynamic potentials for oxygen activation (H_2O/•OH: 2.380 V; O_2/•OOH: 0.05 V), all four catalysts are thermodynamically capable of generating •OH and •OOH radicals via H_2O oxidation and O_2 reduction, implying that their fundamental redox windows are comparable.

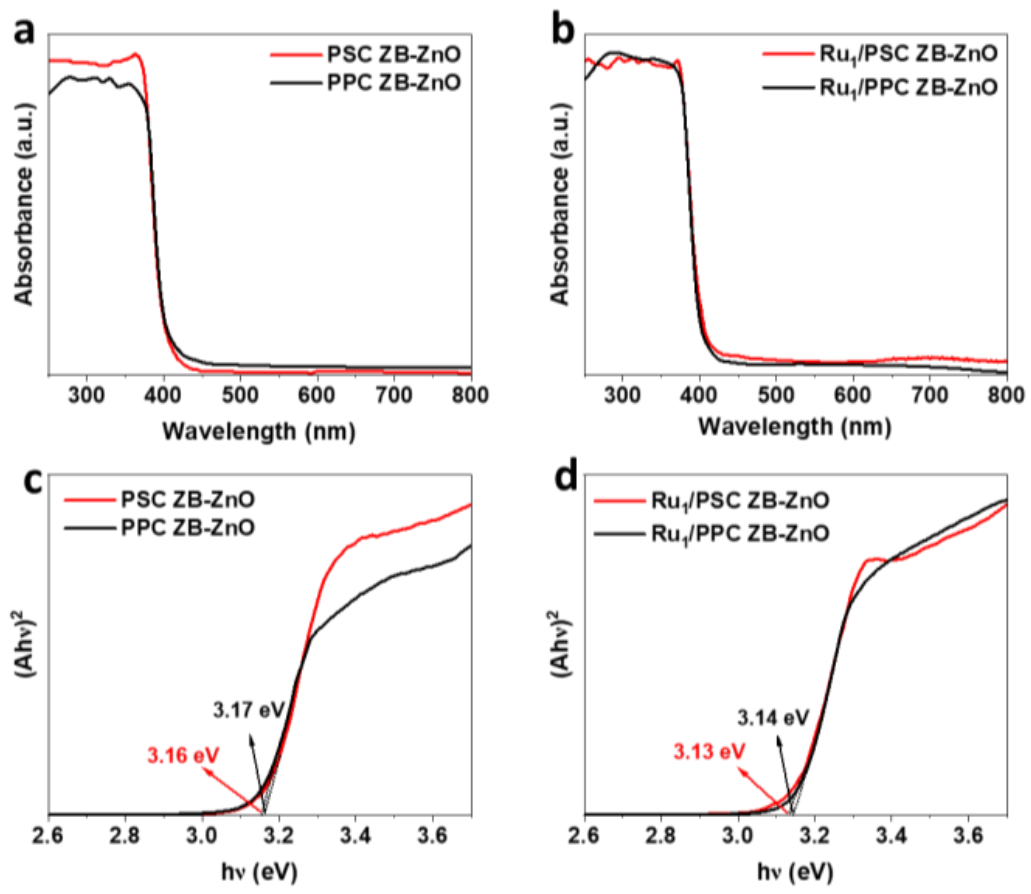

Figure S35. Photophysical properties of ZnO based catalysts with/without Ru modification. (a) UV−visible diffuse reflectance spectra of PSC ZB-ZnO, PPC ZB-ZnO, and PPC ZnO-h. (b) UV−visible diffuse reflectance spectra of Ru_1/PPC ZnO-h. (c-d) Transformed Kubelka-Munk function versus photon energy. The determined bandgaps are E_g = 3.16 eV (PSC ZB-ZnO), 3.17 eV (PPC ZB-ZnO), 3.13 eV (Ru_1/PSC ZB-ZnO), and 3.14 eV (Ru_1/PPC ZB-ZnO), highlighting the influence of Ru incorporation and support (PPC vs PSC) on the electronic structure of ZnO.

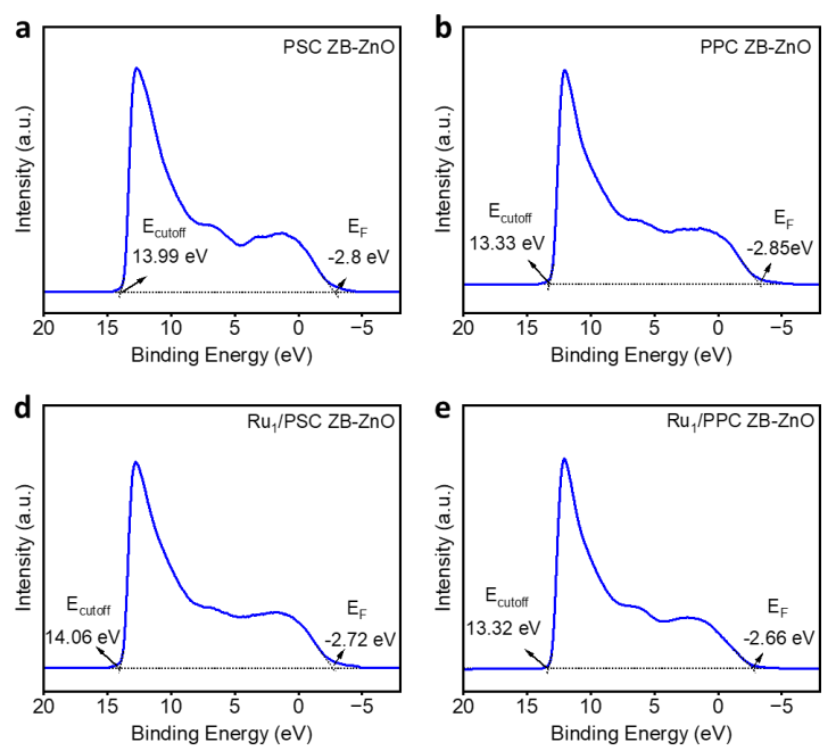

Figure S36. UPS spectrums of ZB-ZnO based catalysts. Thie electron work function of the analyzer was using the formula: $\Phi = h\nu - (E_{cutoff} - E_F)$ ($h\nu$ =21.22 eV).

Therefore, to pinpoint the decisive factor behind the superior performance of Ru_1/PSC ZB-ZnO, we turned to direct probes of charge separation and recombination. Steady-state PL spectra (**Supplementary Figure S41**) show that the emission intensity follows the order: PPC ZB-ZnO > PSC ZB-ZnO > Ru_1/PPC ZB-ZnO > Ru_1/PSC ZB-ZnO. This trend leads to two key conclusions: (i) even without Ru, the porous single-crystalline framework (PSC ZB-ZnO) intrinsically suppresses charge recombination relative to the polycrystalline analogue (PPC ZB-ZnO), which we attribute to the elimination of grain boundary trap states; and (ii) Ru single-atom modification further and strongly quenches the PL for both structures,

confirming its role as an efficient electron sink.

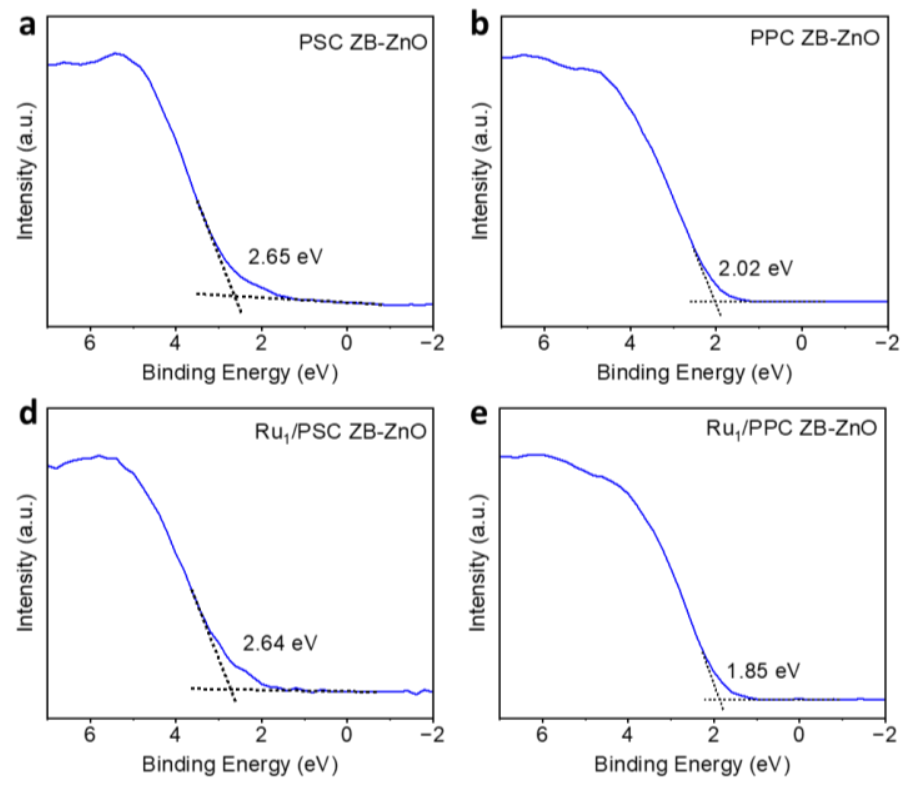

Figure S37. VB-XPS spectrum of ZB-ZnO based catalysts. E_{VB} vs. NHE (V) was calculated using the formula $E_{VB} = \Phi + VB - 4.44$.

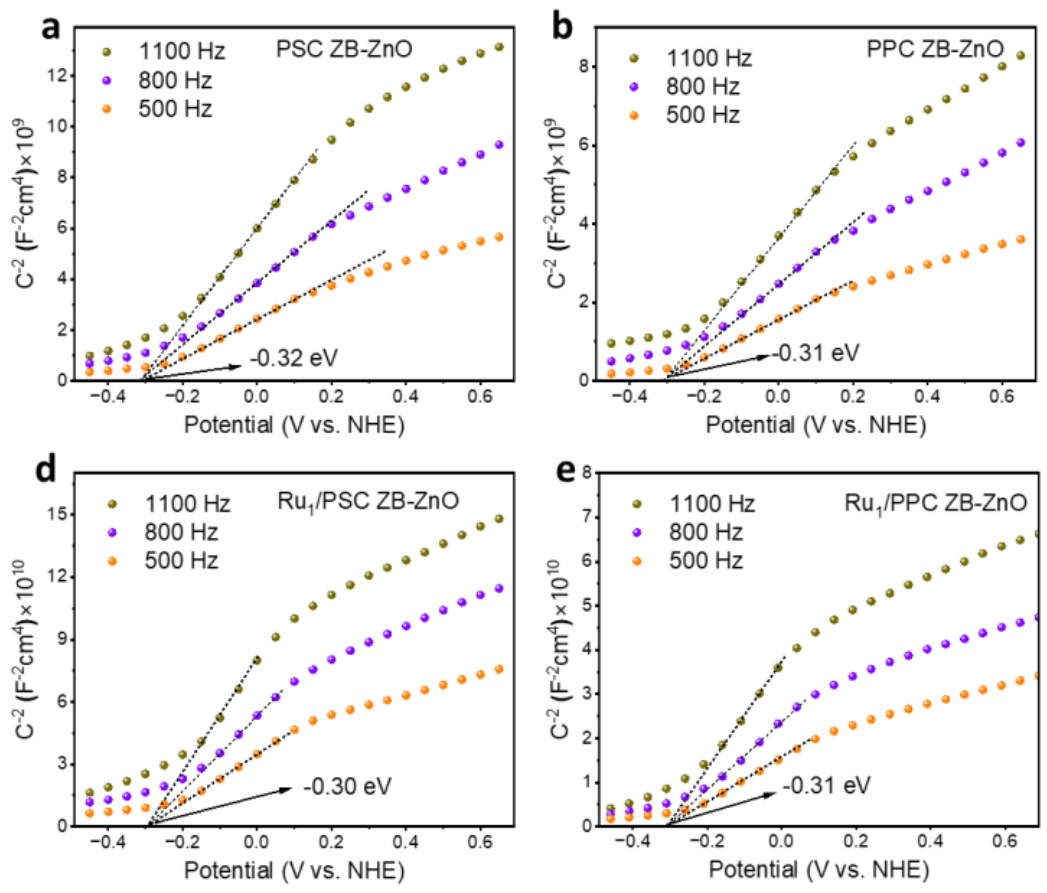

Figure S38. Mott Schottky plots of ZB-ZnO based catalysts. All the samples showed the positive slope of the Mott-Schottky plot, implying n type semiconductors. The flat band potential (E_{fb}) were determined by extending the linear portion of the MS plots. In general, the conduction band potential (E_{CB}) was considered to be ~0.2 eV negative than the E_{fb}.

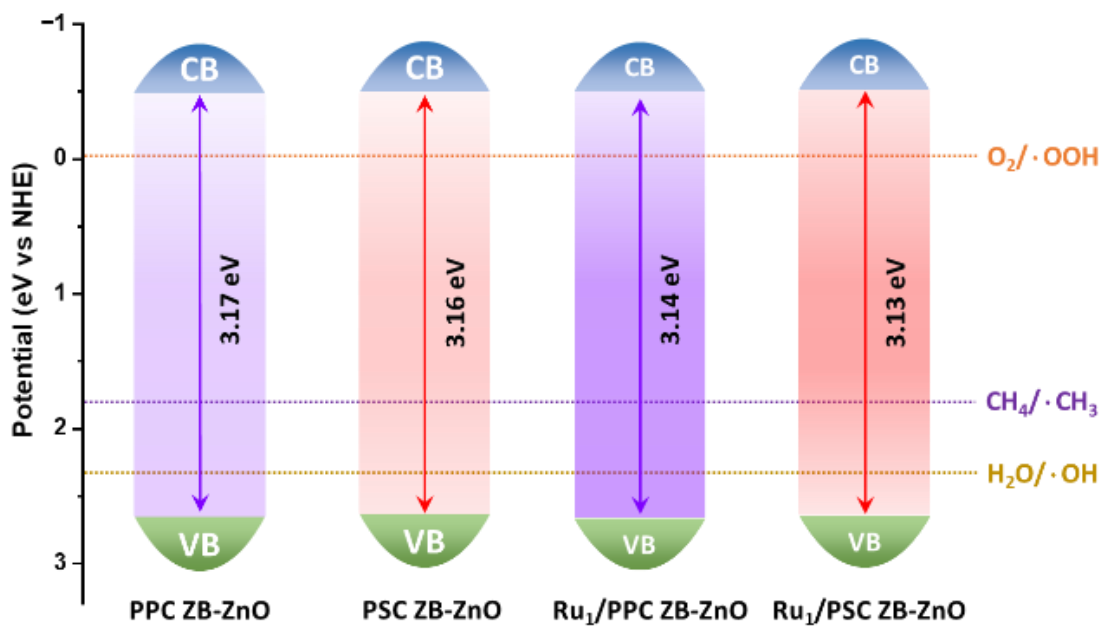

Figure S39. Schematic illustration of the band structures of PPC ZB-ZnO, PSC ZB-ZnO, Ru_1/PPC ZB-ZnO, and Ru_1/PSC ZB-ZnO. determined by VB-XPS valence spectra, UPS spectrums and Mott−Schottky plots.

Time-resolved PL decay measurements (**Fig. 6b**) quantify these effects: the average carrier lifetime (τ_{avg}) of Ru_1/PSC ZB-ZnO (22.3 ns) is significantly longer than that of Ru_1/PPC ZB-ZnO (16.4 ns), and,

notably, pristine PSC ZB-ZnO (τ_{avg} = 17.0 ns) also surpasses PPC ZB-ZnO (τ_{avg} = 12.4 ns). These results unequivocally demonstrate the intrinsic advantage of the single-crystalline matrix in prolonging charge-carrier lifetimes. Complementary electrochemical impedance spectroscopy (EIS, **Supplementary Figure S42**) further shows that Ru-modified catalysts have lower charge-transfer resistance than their unmodified counterparts, with Ru_1/PSC ZB-ZnO exhibiting the smallest semicircle and thus the lowest interfacial resistance. This highlights the synergistic interplay between the support and the single-atom sites: the porous single-crystalline framework provides a highly conductive, low-defect pathway for bulk charge transport, while the atomically dispersed Ru centers promote interfacial charge transfer and steer surface reaction pathways.

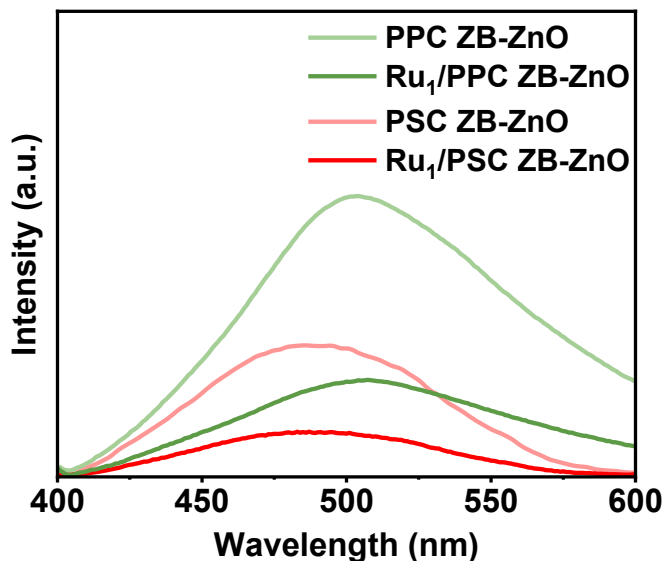

Figure S41. Steady-state PL characterizations of ZnO samples, with and without Ru single-atom modification. Ru modification enhances the charge separation efficiency of ZnO-based photocatalysts, while the single-crystalline structure synergistically suppresses the recombination of photo-generated carriers.

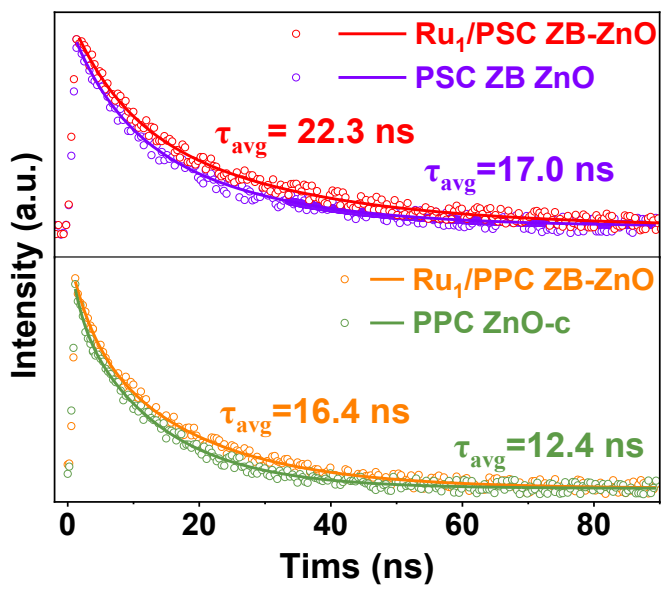

Fig. 6b. Time-resolved PL decay of PSC ZB-ZnO and PPC ZB-ZnO, with and without Ru single-atom modification. The average carrier lifetime (τ_{avg}) for Ru_1/PSC ZB-ZnO, Ru_1/PPC ZB-ZnO, PSC ZB-ZnO, PPC ZB-ZnO are 17.4, 12.6, 14.33, 12.63 ns, respectively.

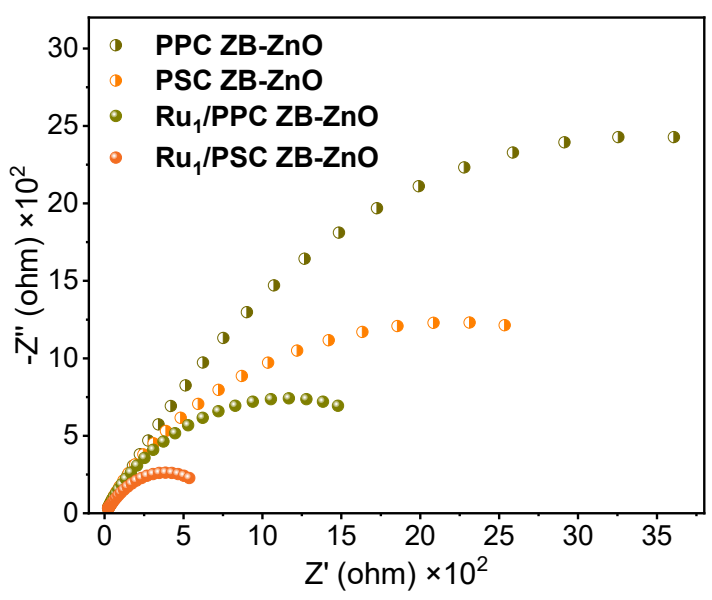

Figure S42. Electrochemical impedance spectroscopy (EIS) of single-crystal and polycrystalline ZnO with and without Ru single-atom modification. EIS were performed in a 0.1 M Na_2SO_4 aqueous. The analysis demonstrates that that single-crystal ZnO (PSC ZB-ZnO) possesses a substantially lower charge-transfer resistance than its polycrystalline counterparts (PPS ZB-ZnO). Moreover, this advantage is further accentuated upon Ru modification. Specifically, the Ru-modified systems display a notably

smaller semicircle radius in the Nyquist plots, evidencing a significant further reduction in charge-transfer resistance. This underscores a synergistic interplay, where the single-crystal morphology provides an efficient conductive pathway, and the atomic-level Ru incorporation actively enhances the interfacial charge-transfer kinetics.

In summary, by carefully choosing a porous polycrystalline ZnO with nearly identical phase, porosity, surface area, and Ru loading as the control, we ensured that the only substantial variable is the presence or absence of grain boundaries. The combined UV–vis DRS, UPS/VB-XPS/Mott–Schottky, PL (steady-state and time-resolved), and EIS analyses consistently show that long-range crystalline order with minimized grain boundaries is the key factor in enhancing charge separation, prolonging carrier lifetimes, and lowering charge-transfer resistance. Ru single-atom modification then builds on this foundation to further optimize band alignment and interfacial kinetics. This powerful synergy between the porous single-crystalline architecture and Ru single atoms underpins the markedly improved photocatalytic methane oxidation performance of Ru_1/PSC ZB-ZnO.

Comment 4: Is the term "topological transformation" used in the manuscript scientifically reasonable? Have the proposed topological properties been strictly supported by structural evidence? Moreover, the reproducibility of this phase transition mechanism has not yet been confirmed. The authors should provide statistical data of crystallographic parameters (such as lattice constants and phase purity) from multiple synthesis batches, with deviations within an acceptable range, to demonstrate its robustness.

Response: Thank you for raising these comments, and we appreciate the opportunity to clarify this point. In our work, the intended term is "**topotactic** transformation", refers to a structural phase change accomplished by the ordered loss/gain and rearrangement of atoms at specific positions in a lattice while remaining the same crystallographic frameworks with the parental phase (Z. Meng et al., Adv. Funct. Mater. 2023, 33, 2305225; Li et al., W. J. Kim, Nature 2023, 615, 237; K. Jia, et al., J. Am. Chem. Soc. 2023, 145,7288).

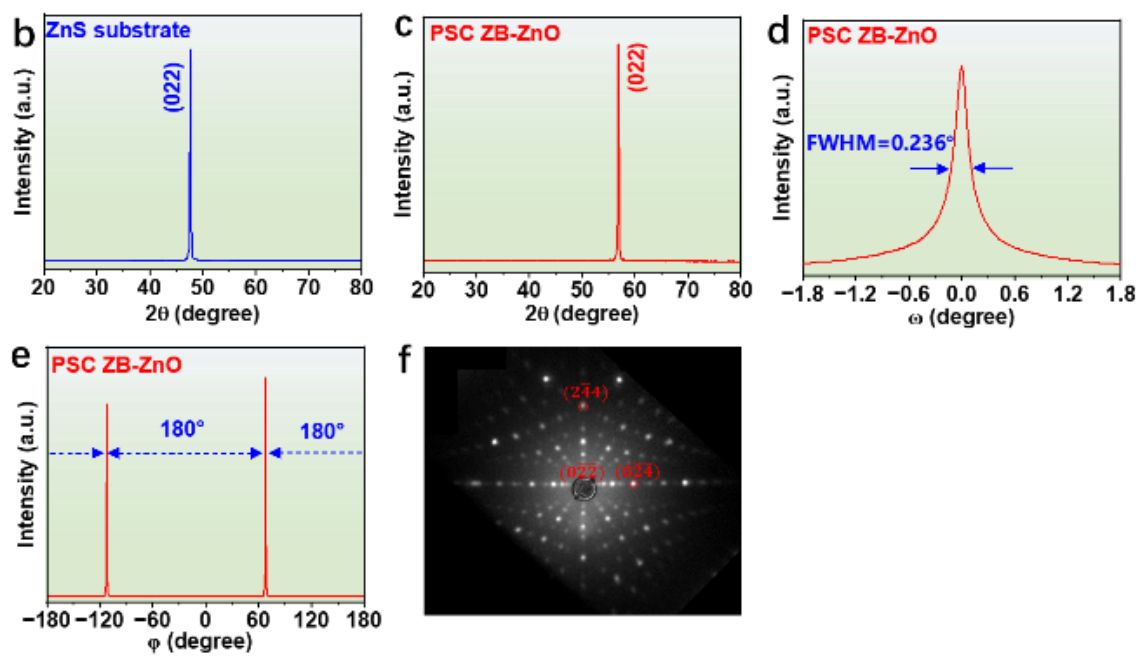

Fig. 1. Structure analysis of PSC ZB-ZnO monolith derived from β-ZnS substrate. **b**, XRD patterns of cubic ZnS substrate. **c**, XRD patterns of PSC BZ-ZnO. **d**, High-resolution rocking curve of PSC ZB-ZnO(022) reflection with full-width at half-maximum (FWHM) of 0.236°, demonstrating exceptional crystallographic coherence. **e**, XRD Φ-scan azimuthal dependence of (002) reflection displaying two-fold symmetry (180° periodicity). **f**, Laue microdiffraction pattern revealing single-crystalline nature.

(2) Direct crystallographic registry from SAED and in situ TEM. In situ SAED during the transformation (**Fig. 3b**) shows co-oriented diffraction spots from β-ZnS and ZB-ZnO, with ZB-ZnO (200), (111), and (022) reflections emerging in fixed orientation relative to the parent lattice. No ring-

like diffraction, intermediate phases, or misoriented domains are observed, ruling out dissolution–reprecipitation or random nucleation and confirming an epitaxial, topotactic mechanism.

In particular, a topotactic transformation is generally defined as a solid–solid reaction in which: **(1)** The parent and product phases maintain a crystallographic orientation relationship; **(2)** The cation (or anion) sublattice is largely preserved, with transformation proceeding via ordered substitution or rearrangement; **(3)** The overall lattice registry is retained, even though composition and lattice parameters may change. Our β-ZnS → PSC ZB-ZnO transformation fully satisfies this definition, and we have provided multiple, mutually consistent structural pieces of evidence to support this:

(1) Preservation of crystal orientation and single-crystal character. XRD Φ-scan, rocking curve, and Laue diffraction (**Fig. 1d–f**) confirm that the ZnO product remains single-crystalline with the same macroscopic orientation as the β-ZnS substrate. The (022) reflection of β-ZnS is preserved as the (022) family of planes in PSC ZB-ZnO (**Fig. 1b–c**), demonstrating crystallographic inheritance rather than random nucleation of new grains.

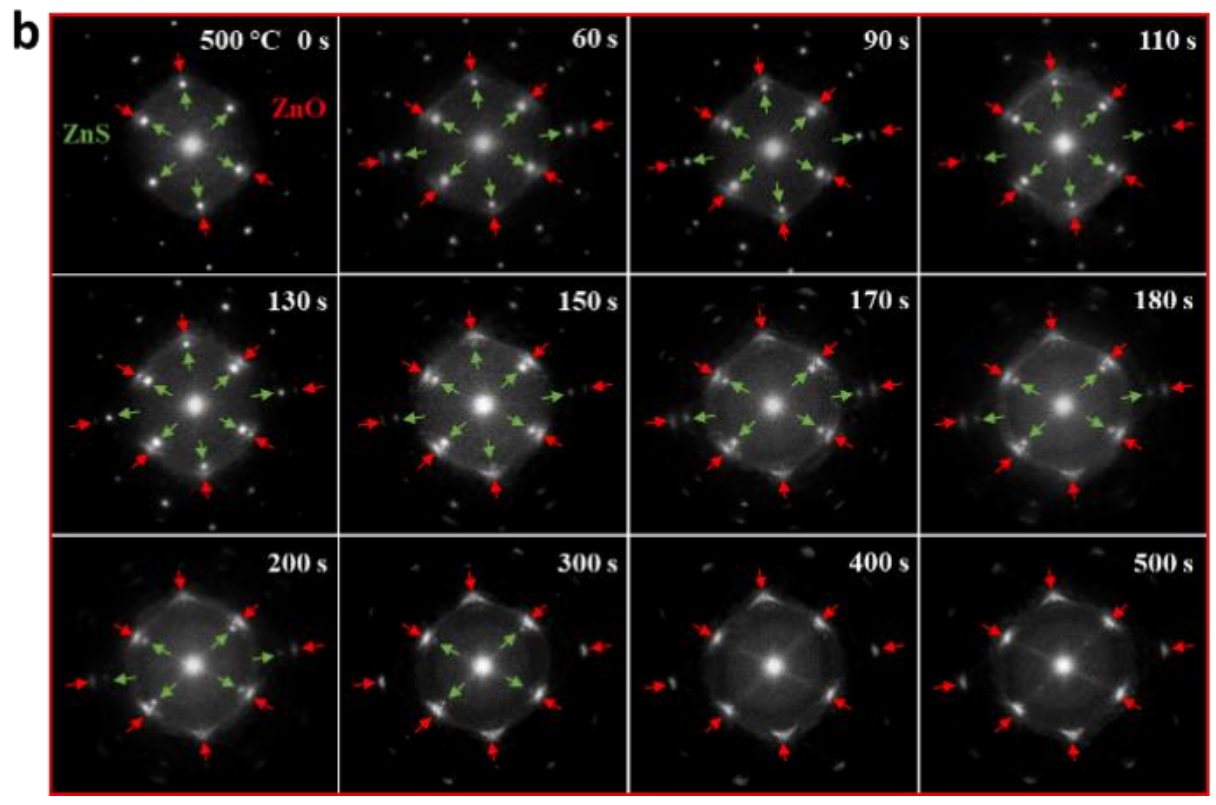

Fig. 3. SAED pattern during topotactic transition from β-ZnS single crstal to PSC ZB-ZnO acquired via in situ TEM under 5×10⁻⁴ Torr O_2. **b,** Sequential SAED evolution under isothermal oxidation (500°C, 0→500 s).

(3) Preservation of the Zn^{2+} sublattice with anion exchange. Atomic-resolution HRTEM and Zn–Zn interatomic spacing maps along the ⟨112⟩ direction (**Fig. 4b–c, e–f**) show that the Zn^{2+} fcc sublattice is preserved, while S^{2-} is replaced by O^{2-}. The Zn–Zn spacing contracts from 3.36 Å (β-ZnS) to 2.79 Å (ZB-ZnO), consistent with the lattice contraction from a = 5.406 Å to 4.580 Å, but the underlying lattice topology and orientation remain unchanged.

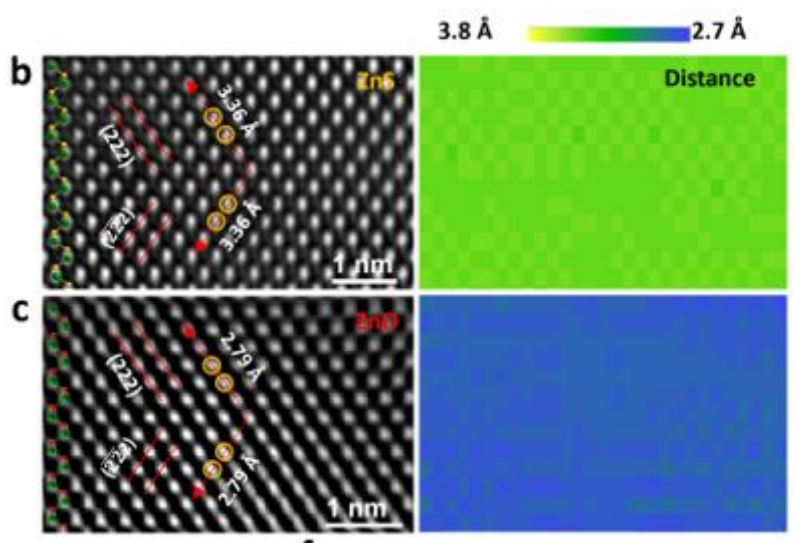

Fig. 4. Topotactic transition process from β-ZnS to PSC ZB-ZnO captured by in situ HRTEM at 500 °C. **b-c,** Application results of the HRTEM image analyses for phase identification. (Left) HRTEM images of ZnS and ZB-ZnO with Zn-Zn interatomic spacing along the ⟨112⟩ direction measuring 3.36 Å and

2.79 Å, respectively. (Right) Interatomic spacing map of $Zn\text{-}Zn_{(112)}$.

(4) Same space group and direct, registry-preserving phase change. Rietveld refinement (**Supplementary Fig. 7 and Table S1**) confirms that both β-ZnS and ZB-ZnO share the same cubic space group (F-43m), and that the transformation proceeds without intermediate phases or loss of long-range order. Ex situ XRD and in situ TEM show a direct β-ZnS → ZB-ZnO transformation via S/O anion exchange along {222} diffusion channels, again consistent with a classic topotactic process.

(5) Coupling of lattice contraction and porosity formation within a single-crystal framework. In situ TEM (**Supplementary Figs. 10, 15–17**) demonstrates that porosity forms through vacancy aggregation and strain relaxation inside the preserved single-crystal lattice, not through grain boundary formation or crystal fragmentation.

Taken together, these results rigorously support that the β-ZnS → PSC ZB-ZnO conversion is a topotactic transformation in the strict crystallographic sense: the product inherits the orientation and cation framework of the parent single crystal, with anion exchange and lattice contraction occurring in a registry-preserving manner, while simultaneously generating porosity.

In addition, we fully agree with you that demonstrating the reproducibility and robustness of the phase transition is essential. In response, we have conducted additional syntheses of porous single-crystal ZB-ZnO from at least six independent batches under identical conditions. For each batch, we performed XRD rietveld refinement to extract lattice constants and phase purity (**Supplementary S7**), and the results are summarized in the revised **Supplementary Table S1.** The lattice constants of ZB-ZnO across batches, consistently averages at ~4.584 Å with a minimal variation of ±0.004 Å, shows no secondary phases. The deviations in lattice parameters are well within the accepted range for high-quality crystals, demonstrating the high reproducibility and robustness of our topotactic transformation strategy.

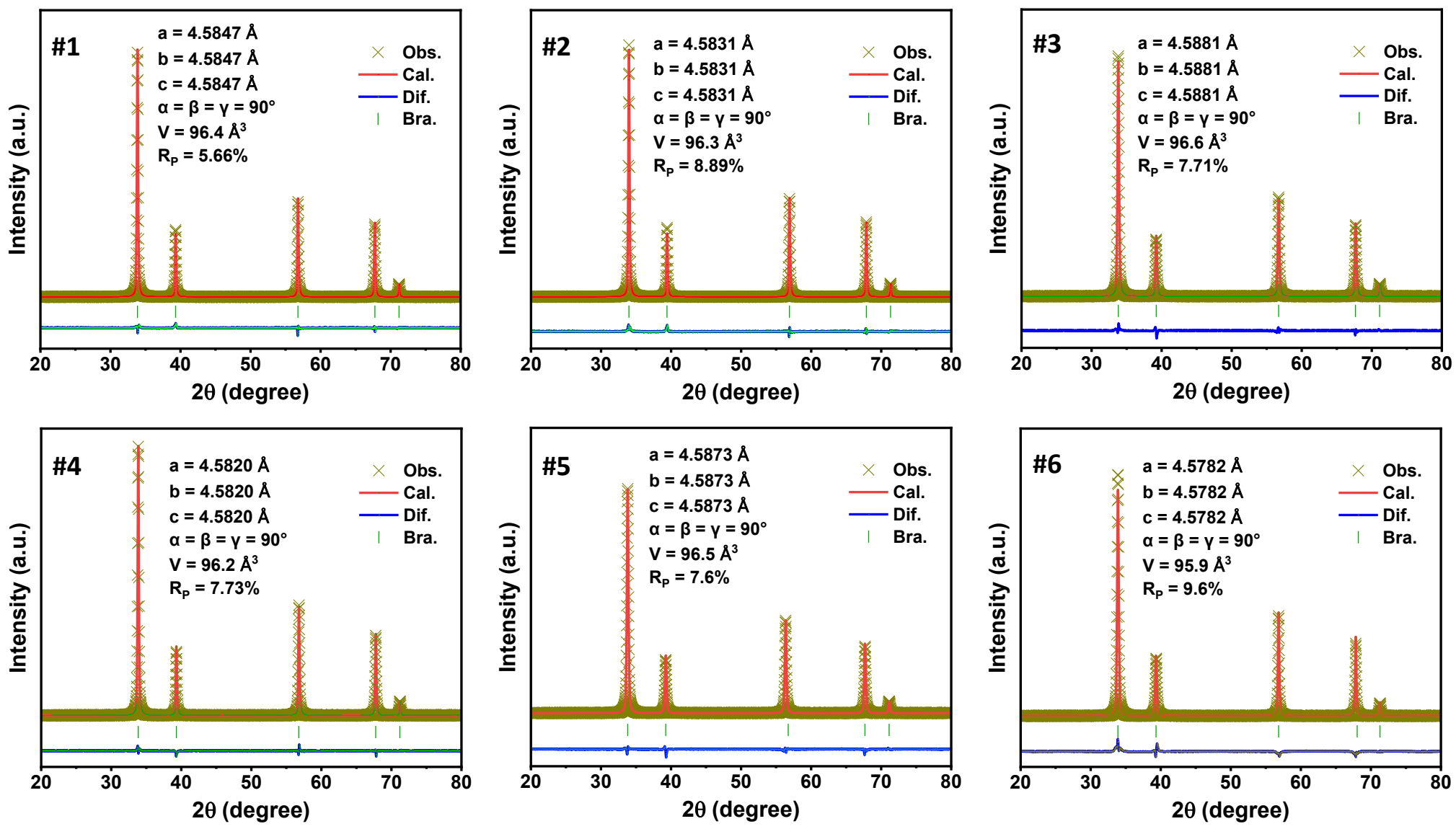

Figure S7. XRD rietveld refinement plots for different samples from multiple independent synthesis batches. under identical conditions.

Table S1. Summary of the results obtained from rietveld XRD studies: lattice parameters (a, b, c), unit

cell volume (V), and R_p factor.

Sample	a (Å)	b (Å)	c (Å)	V (Å^3)	R_p (%)	Phase
#1	4.5847	4.5847	4.5847	96.4	5.66	Cubic
#2	4.5831	4.5847	4.5847	96.3	8.89	Cubic
#3	4.5881	4.5881	4.5881	96.6	7.71	Cubic
#4	4.5820	4.5820	4.5820	96.2	7.73	Cubic
#5	4.5873	4.5873	4.5873	96.5	7.60	Cubic
#6	4.5782	4.5782	4.5782	95.9	9.60	Cubic

Comment 5: The long-term stability of atomically dispersed Ru species under operational conditions is a key concern. The authors should provide evidence, such as post-reaction HAADF-STEM or XAS, to confirm that Ru remains as isolated atoms without aggregation during prolonged catalytic cycling.

Response: Thank you for raising this critical concern regarding the stability of atomically dispersed Ru species under operational conditions. We fully agree that confirming the retention of single-atom dispersion after prolonged catalysis is essential for establishing the robustness of our catalyst design. As explicitly documented in **Supplementary Figure S32** (Post-reaction HAADF-STEM), isolated Ru atoms (highlighted by yellow circles) remain uniformly dispersed on the ZB-ZnO matrix after 43 hours of continuous photocatalytic methane oxidation, with no evidence of agglomeration into nanoparticles or clusters. This direct atomic-scale evidence confirms the exceptional stability of Ru single atoms anchored within the porous single-crystalline framework.

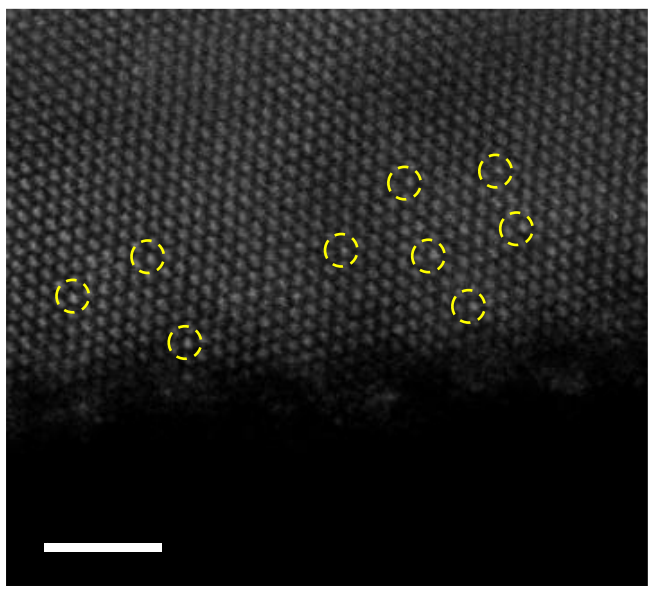

Figure S32: Post-reaction atomic-scale characterization of Ru_1/ZB-ZnO. HAADF-STEM confirm persistent single-atom dispersion of Ru (yellow circles) on ZnO after catalytic reaction. No Ru clusters/nanoparticles observed.

Comment 6: The mechanistic discussion of hydrogen and oxygen transfer pathways lacks definitive experimental support. Isotope labeling experiments (e.g., using $^{18}O_2$ or CD_4) are recommended to trace the origin of oxygen and hydrogen atoms in the methyl hydroperoxide product and to validate the proposed reaction mechanism.

Response: We appreciate your insightful suggestion to strengthen the mechanistic evidence for CH_3OOH formation. To definitively trace the origin of oxygen in CH_3OOH, we conducted isotope experiments using ^{18}O-labeled oxygen ($^{18}O_2$) or ^{18}O-labeled water ($H_2{}^{18}O$) in the reaction and analyzed the oxygenated products by gas chromatography-mass spectrometry (GC-MS). To prevent CH_3OOH thermal decomposition during GC-MS analysis under aerobic conditions, we reduced CH_3OOH to CH_3OH with $NaBH_4$ before analysis. As shown in Fig. 6f, the use of $^{18}O_2$ and $H_2{}^{16}O$ resulted in strong signal peaks for $CH_3{}^{18}OH$, whereas no $CH_3{}^{18}OH$ was detected in reactions carried out with $^{16}O_2$ and $H_2{}^{18}O$. This provides strong evidence that O_2 is the source of oxygen for CH_3OOH. Moreover, control experiments conducted

in the absence of O_2 yielded no product formation, which conclusively rules out any contribution from lattice oxygen (Supplementary **Fig. 34**). We also conducted isotope labeling experiments with CD_4+H_2O, For CD_3OH/ CD_3OD derived from CD_4, indicate the CH_3 in CH_3OOH clearly originates from CH_4.

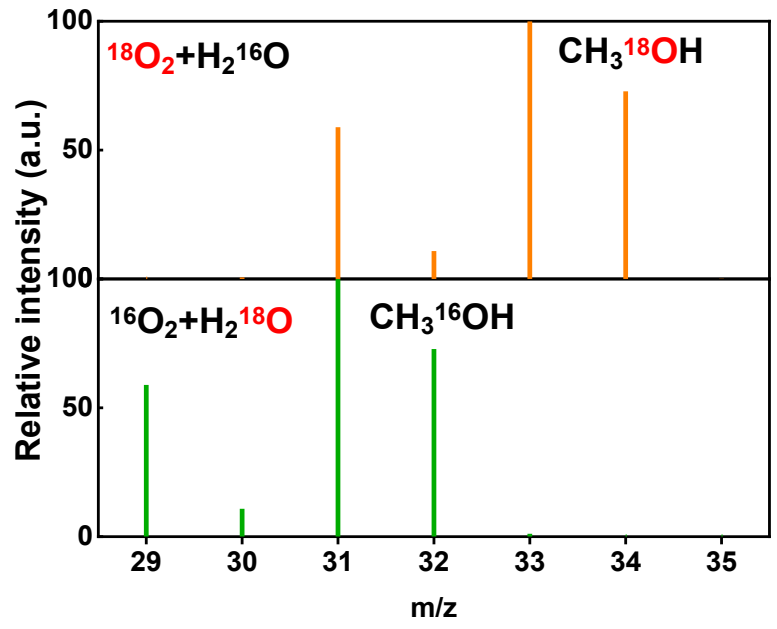

Figure 6f. GC-MS spectra of CH_3OH reduced CH_3OOH over Ru_1/PSC ZB-ZnO with $^{18}O_2$ + $H_2{}^{16}O$ or $^{16}O_2$ + $H_2{}^{18}O$ in photocatalytic CH_4 oxidation.

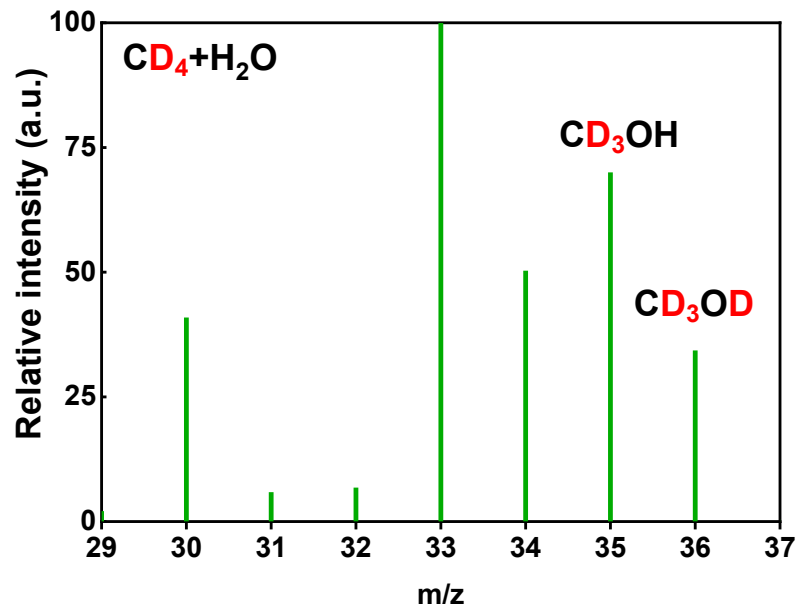

Figure R2. GC-MS spectra of CH_3OH generated over Ru_1/PSC ZB-ZnO with CD_4 + $H_2{}^{16}O$ in photocatalytic CD_4 oxidation.

Comment 7: The photocatalytic performance measurements in this manuscript were conducted under a light intensity of 1000 mW/cm², which is ten times the intensity of sunlight. Therefore, the light source should not be described as AM 1.5G (100 mW/cm²). Under such high-intensity illumination, significant heating of the catalyst surface is expected, which raises concerns about potential thermal contributions to the observed reactivity. As a result, it becomes difficult to distinguish whether the catalytic process is purely photocatalytic or involves photo-thermal effects. To clarify the reaction mechanism, direct measurement of the catalyst surface temperature during irradiation is essential. Furthermore, a rigorous evaluation should be provided to demonstrate that the observed methane oxidation is driven solely by photocatalytic processes, rather than by thermally activated pathways.

Response: We sincerely thank you for raising this critical point regarding the experimental light intensity and its potential implications for distinguishing photocatalytic from photothermal effects. Your concerns are highly valid, and we appreciate the opportunity to clarify our experimental conditions and provide additional evidence supporting a predominantly photocatalytic mechanism.

(1) In our experiments, we employed a 300 W Xenon lamp equipped with an AM 1.5G filter to simulate the solar spectral distribution. **Figure S23** shows the Xenon lamp light source spectral distribution with AM 1.5G filter. The power density reaching the catalyst bed was indeed set to 1000 mW/cm^2 by adjusting the lamp output and reactor geometry, which exceeds the standard intensity for the AM 1.5G solar spectrum (100 mW/cm^2). We acknowledge that describing this condition simply as "AM 1.5G" in the

main text was imprecise, as it conflates spectral quality with intensity. We have amended the manuscript to accurately state: "…under simulated AM 1.5G spectral irradiation with a power density of 1000 mW cm^{-2}."

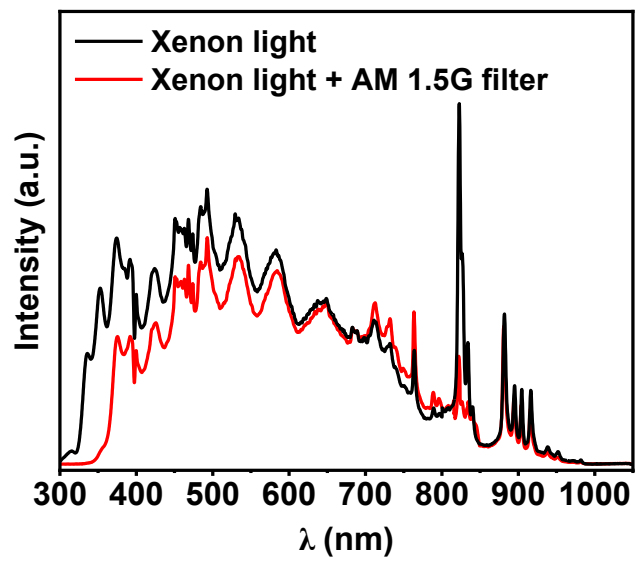

Figure S23. The spectrum of Xenon lamp light with or without AM 1.5G filter.

(2) **Catalyst surface temperature:** The reaction was conducted in a continuous-flow gas-solid reactor, which is integrated with a precise circulating water system. This could effectively dissipate any incidental heat, preventing significant thermal heating of the catalyst. The measured catalyst bed temperature stabilized at 32 °C under illumination and remained constant over 120 minutes of testing (**Figure S24**).

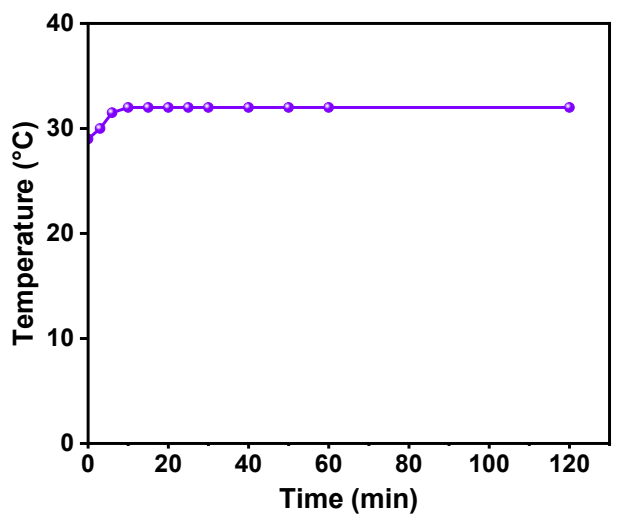

Figure S24. Catalyst surface temperature during reaction. The temperature of catalysts detected by a thermometer under light at different reaction time during photothermal catalytic oxidation of CH_4. The temperature on the surface of catalysts is accurately controlled via a circulating water system.

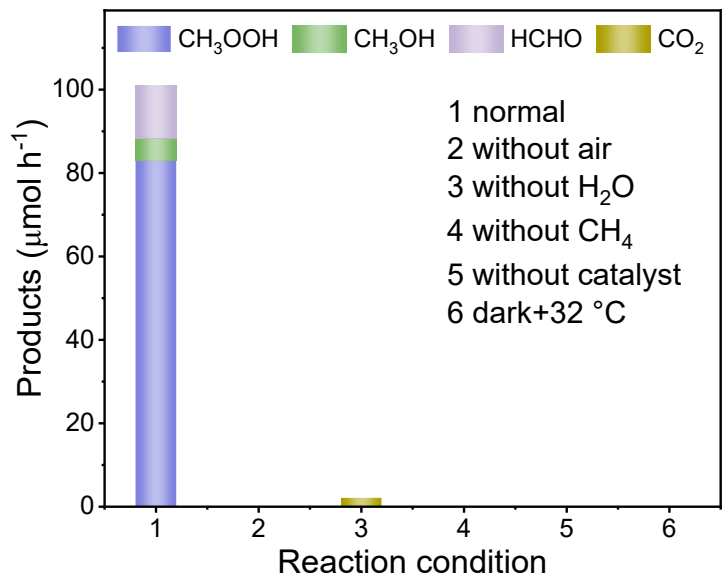

Figure S34. Control experiments screening the necessity of key reaction components. Production rates obtained over Ru_1/ZnO under different conditions. Reaction conditions: 45 mL min^{-1} CH_4 and 5 mL min^{-1} air (25 vol.% O_2/N_2), RH = 20%, 50 °C, 300 W Xe lamp with AM 1.5G filter, 1000 mW cm^{-2}. The results demonstrate that significant product formation occurs exclusively under light irradiation with all reaction components present. The negligible activity observed in the dark (even with system temperature rise to 32 °C), under visible light, in the absence of methane, or without catalyst, systematically rules out thermal effects. O_2 and H_2O is essential for initiating methane oxidation.

(3) Evidence for a predominantly photocatalytic mechanism: Control experiments conducted at the same temperature (32°C) under dark conditions showed no product formation (**Supplementary Figure**

S34), definitively confirming that the reaction is fundamentally light-dependent and ruling out any thermal contribution. Moreover, the yield exhibited a near-linear dependence on light intensity between 200 and 1000 mW/cm². The linear dependence indicates a photocatalytic mechanism limited by photon flux, rather than a photothermal one which would typically show a nonlinear response due to heat accumulation.

Comment 8: Please provide a clear photo of the gas-phase continuous-flow photoreactor used in this research so that readers can have a more intuitive understanding of the application situation.

Response: Thank you for this valuable suggestion regarding the visualization of continuous-flow gas–solid photoreactor in our experiment. As requested, we have included clear photographs of the custom-designed continuous-flow gas-solid photoreactor in **Supplementary Figure S22**. This image provides an intuitive overview of the reactor configuration, clearly showing the reaction chamber, gas inlet/outlet ports, and quipped with a circulating water system to maintain the reaction at room temperature. The reactor's design ensures that the reaction gas passes through the catalyst bed, making the catalytic reaction proceed more fully.

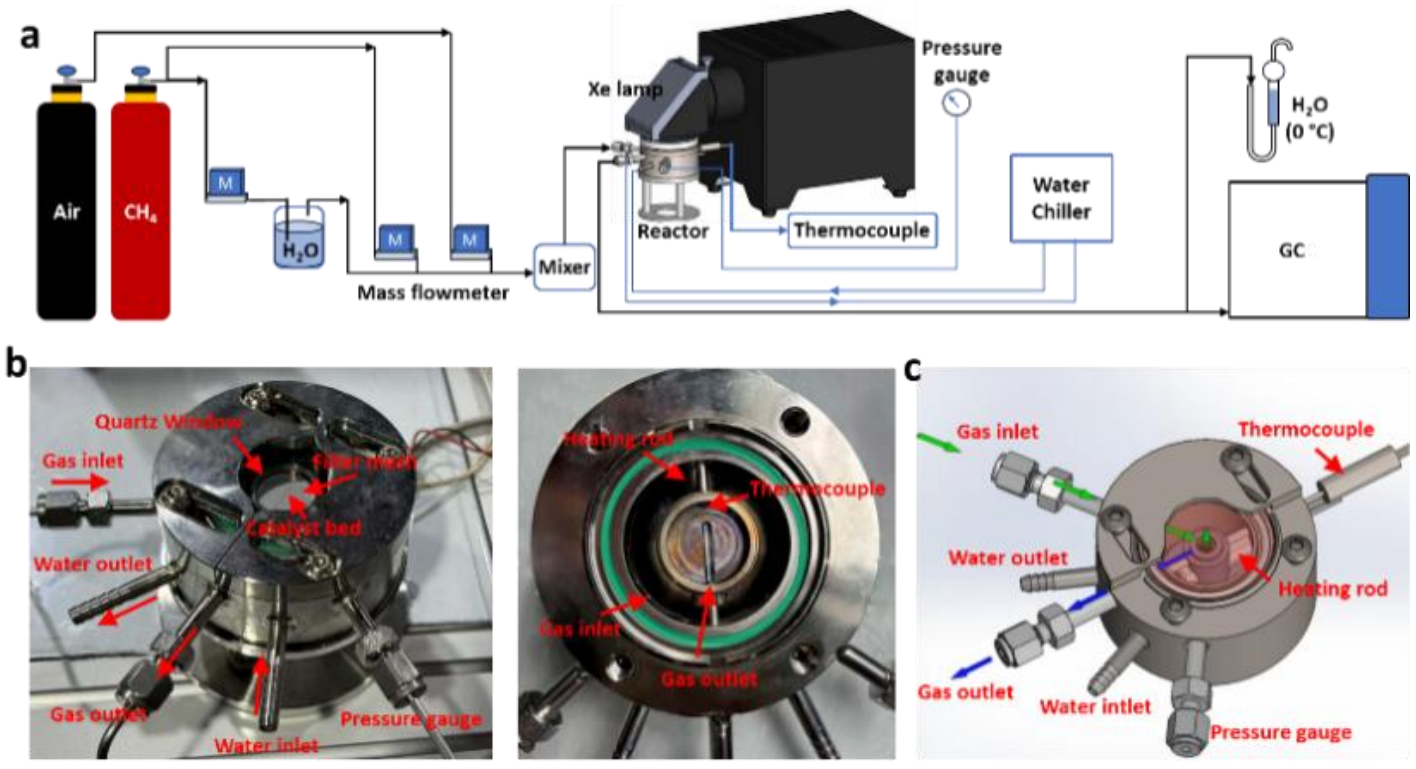

Figure S22. Equipment diagram. **a,** Schematic diagram of the experimental setup. **b,** Physical photo of the continuous-flow gas-solid photoreactor for photocatalytic oxidation of CH_4. **c,** Schematic diagram of the gas-phase continuous-flow photoreactor.

Comment 9: The most valuable products in methane selective oxidation are typically multi-carbon compounds formed via C-C coupling reactions. In contrast, all products reported in this study are single-carbon (C1) species, and the formation rates of the three detected products under 10-sun illumination are all below 100 μmol/h. Does this level of performance demonstrate competitiveness under comparable irradiation conditions? To enable a rigorous comparison, please provide a detailed summary table of relevant studies, including key experimental parameters such as light intensity, reaction temperature, catalyst loading, photocatalytic reactor configuration, and gas composition.

Response: Thank you for raising this important point regarding product value and performance benchmarking. We respectfully contend that while C-C coupling to multi-carbon products is a highly desirable long-term goal, the selective and stable photocatalytic production of single-carbon oxygenates under mild conditions remains a fundamental and technologically significant challenge. The central achievement of our work is the steering of methane partial oxidation toward methyl hydroperoxide (CH_3OOH) with high selectivity (82.5%) and exceptional stability (>40 h) at near-ambient temperature, which represents a distinct and valuable pathway. CH_3OOH is a valuable propellant and a versatile

precursor in organic synthesis; its direct, selective formation from CH_4 and O_2 via photocatalysis is particularly challenging due to the tendency for over-oxidation to methanol or formaldehyde. Our system addresses this by utilizing atomically dispersed Ru sites on a porous single-crystalline ZnO matrix to precisely control the oxygen reduction pathway, favoring •OOH intermediates which couple with $•CH_3$ to form CH_3OOH, effectively suppressing deeper oxidation.

Regarding performance under 10-sun illumination (1000 mW cm^{-2}), a yield of 84.6 μmol h^{-1} for CH_3OOH with the stated selectivity and stability is highly competitive. The key advantage lies not in raw yield alone, but in the combination of high selectivity for a challenging product under continuous flow, and operation at mild temperature (catalyst bed 32°C), which minimizes thermal energy input. We fully agree that a rigorous comparison is essential. As requested, we have provided detailed supplementary tables summarizing key parameters from relevant studies, including light intensity, reaction temperature, catalyst loading, reactor type, gas composition, and performance metrics. These table will clearly contextualize our work, highlighting its contributions in achieving selective CH_3OOH production under illumination in a gas-solid flow system. We appreciate your suggestion to enhance the comparative analysis of our work.

Table S4: Performance comparison of photocatalysis. Representative works on the selective photocatalytic conversion of CH_4 to CH_3OOH.

Catalysts	Reactor type	Reactants	T (°C)	Light	CH_3OOH (μmol/h)	Selectivity (%)	Ref.
Ru_1/PSCZB-ZnO	flow	45 mL min^{-1} CH_4 and 5 mL min^{-1} air (25 vol.% O_2/N_2), RH = 20%; Cat.: 100 mg	32	1000 mW cm^{-2}	83.0	82.3	**This work**
b-TiO_2	batch	P_{CH4} = 0.1 MPa; 20 mL 50 mM H_2O_2, 1 h; Cat.: 2 mg	25	300 mW cm^{-2}	1.5	100	8
Ru_1O_x/ZnO	batch	P_{CH4} = 2 MPa; P_{O2} = 0.1 MPa; 10 mL of H_2O, 1 h; Cat.: 10 mg	25	150 mW cm^{-2}	3.2	90.9	9
0.5Ru-ZnO	batch	P_{CH4} = 2 MPa; P_{O2} = 0.1 MPa; 180 mL of H_2O, 1h; Cat.: 10 mg	30	365 nm LED (75 mW cm^{-2})	100.2	86.1	10
$Pd_{0.5}$-def-WO_3	batch	P_{CH4} = 1.9 MPa; P_{O2} = 0.1 MPa; 100 mL of H_2O, 2h; Cat.: 20 mg	25	100 mW cm^{-2}	92.9	80.3	11
Pd-def-In_2O_3	batch	P_{CH4} = 1.9 MPa; P_{O2} = 0.1 MPa; 50 mL of H_2O, 3 h; Cat.: 20 mg	25	LED lamp (421 nm)	36	60	12
ZnO(Zni)-350	batch	P_{CH4} = 1.9 MPa; P_{O2} = 0.2 MPa; 180 mL of H_2O, 2 h; Cat.: 10 mg	30	550 mW cm^{-2}	18.6	60.4	13
$Au_{0.2}Cu_{0.15}$-ZnO	batch	P_{CH4} = 1.9 MPa; P_{O2} = 0.1 MPa; 100 mL of H_2O, 2h; Cat.: 20 mg	25	150 mW cm^{-2}	124.7	56.9	14
Au/ZnO	batch	P_{CH4} = 2.9 MPa r; P_{O2} = 0.1 MPa; 30 mL of H_2O; 2 h; Cat.: 10 mg	25	320 nm - 780 nm; 300 mW·cm^{-2}	59.4	54	15
$Au_{0.15}$/ZnO	batch	P_{CH4} = 1.5 MPa; P_{O2} = 0.5 MPa;100 mL of H_2O; 2 h; Cat.: 10 mg	30	100 mW cm^{-2}	5.4	52.8	16
Cu-W-TiO_2	batch	P_{CH4} = 2 MPa; P_{O2} = 0.2 MPa; 80 mL of H_2O, 2 h; Cat.: 10 mg	30	350 - 760 nm, 200 mW cm^{-2})	77.5	47	17

0.1 wt % Au/ZnO	batch	P_{CH4} = 2 MPa; P_{O2} = 0.1 MPa;100 mL of H_2O; 2 h; Cat.: 10 mg	25	300-500 nm, 100 mW cm^{-2}	62.7	47	18
1%Cu/ZnO	batch	P_{CH4} = 1.9 MPa; P_{O2} = 0.1 MPa; 80 mL of H_2O; 2 h; Cat.: 5 mg	30	16 A	40.5	42	19
Au/CoO_x/TiO_2	batch	P_{CH4} = 2 MPa; P_{O2} = 0.1 MPa; 180 mL of H_2O; 2 h; Cat.: 10 mg	25	450 mW cm^{-2}	10.4	39	20
Au_1Pd_9-ZnO	batch	P_{CH4} = 1.9 MPa; P_{O2} = 0.1 MPa; 75 mL of H_2O; 1h; Cat.: 10 mg	25	350 mW cm^2	40	34.8	21
Au-Pd/TiO_2	batch	P_{CH4} = 3 MPa; P_{O2} = 1 MPa; 30 mL of H_2O, 1h; Cat.: 5 mg	42	250 mW cm^{-2}	31	31	22

Table S5: Performance comparison of photocatalysis. Representative works on the selective photocatalytic oxidation of CH_4 to C_1 oxygenates at high density light.

Catalysts	Reactor type	Reactants	T (°C)	Light	Oxygenates (μmol/h)	Selectivity (%)	Ref.
Ru_1/PSCZB-ZnO	flow	45 mL min^{-1} CH_4 and 5 mL min^{-1} air (25 vol.% O_2/N_2), RH = 20%; Cat.: 100 mg	32	350-1000 nm; 1000 mW cm^{-2}	100.9	99.9	**This work**
FeO_x/ZnO	batch	P_{CH4} = 3 MPa; 5 mM H_2O_2; 90 mL of H_2O, 3 h; Cat.: 5 mg	R.T.	full spectrum, 1500 mW cm^{-2}	20.7	99.9	23
Ag_1-ZnO	flow	45 mL min^{-1} CH_4 and 5 mL min^{-1} air (25 vol.% O_2/N_2), RH = 20%; Cat.: 4 mg	228	200 - 1200 nm; 1000 mW cm^{-2}	124	73.9	24
1wt.%Pd-ZnO	flow	50 mL min^{-1} CH_4 and 10 mL min^{-1} air (25 vol.% O_2/N_2); Cat.: 0.5 mg	35	full spectrum, 600 mW cm^{-2}	3.3	100	25
0.5Ru-ZnO	batch	P_{CH4} = 2 MPa; P_{O2} = 0.1 MPa; 180 mL of H_2O, 1h; Cat.: 10 mg	150	365 nm LED (75 mW cm^{-2})	476.2	95.4	10
ZnO(Zni)-350	batch	P_{CH4} = 1.9 MPa; P_{O2} = 0.2; 180 mL of H_2O, 2 h; Cat.: 10 mg	30	550 mW cm^{-2}	28.9	98.6	13
Au/CoO_x/TiO_2	batch	P_{CH4} = 2 MPa; P_{O2} = 0.1; 180 mL of H_2O, 2 h; Cat.: 10 mg	25	450 mW cm^{-2}	25.4	95	20
Ni-NC/TiO_2	batch	P_{CH4} = 2 MPa; P_{O2} = 0.1 MPa; 100 mL of H_2O, 4h; Cat.: 10 mg	25	300-500 nm, 450 mW cm^{-2})	50.2	93	26
3.2%Ag/TiO_2 {001}	batch	P_{CH4} = 2 MPa; P_{O2} = 0.1 MPa; 100 mL of H_2O, 2h; Cat.: 10 mg	25	300-500 nm, 450 mW cm^{-2}	54.1	90	27
Au_1Pd_9-ZnO	batch	P_{CH4} = 1.9 MPa; P_{O2} = 0.1 MPa; 75 mL of H_2O, 1h; Cat.: 10 mg	25	350 mW cm^2	101.3	86.75	21

Au/ZnO	batch	P_{CH4} = 29 bar; P_{O2} = 1 bar; 30 mL of H_2O; 2 h; Cat.: 10 mg	25	320-780 nm; 300 mW·cm^{-2}	106.2	96.6	15
Au_1/In_2O_3	batch	P_{CH4} = 2 MPa; P_{O2} = 1 MPa; 180 mL of H_2O, 7h; Cat.: 10 mg	25	300-1100 nm, 250 mW cm^{-2}	18.8	95	28

Table S6. Comparison of catalytic performance. Representative works on photocatalytic CH_4 conversion to C_1 oxygenates in flow reactor.

Catalysts	Reactants	T/°C	Light	Oxygenates (μmol/h)	Selectivity (%)	Ref.
Ru_1/PSC ZB-ZnO	45 mL min^{-1} CH_4 and 5 mL min^{-1} air (25 vol.% O_2/N_2), RH = 20%; Cat.: 100 mg	32	350 - 1000 nm; 1000 mW cm^{-2}	100.9	99.9	**This work**
Ag_1-ZnO	45 mL min^{-1} CH_4 and 5 mL min^{-1} air (25 vol.% O_2/N_2), RH = 20%; Cat.: 4 mg	228	200 - 1200 nm; 1000 mW cm^{-2}	124	73.9	24
PMOF-RuFe(OH)	$O_2 + H_2O$, ~1 bar, 3ml H_2O; Cat.: 10 mg	R.T.	400–780 nm	88.1	100	29
1wt.%Pd-ZnO	50 mL min^{-1} CH_4 and 10 mL min^{-1} air (25 vol.% O_2/N_2); Cat.: 0.5 mg	35.3	full spectrum, 600 mW cm^{-2}	3.3	100	25
90% A-TiO_2	Cat.: 10 mg; P_{CH4} = 20 bar; P_{O2} = 5 bar; 180 mL of H_2O, 3 h	25	300-1100 nm, 130 mW cm^{-2}	34	99.6	30
MoO_3 (5 wt.%)/SiO_2	Cat.: 25 mg; 7.4 mmol h^{-1} CH_4, (CH_4: O_2: He= 6: 2: 25);	220	high-pressure mercury vapour lamp (200 W)	6	100	31
MoO_3 (5 wt.%)/ZnO	Cat.: 250 mg; 7.5 mmol h^{-1} CH_4, (CH_4: O_2: He = 3:1:10);	220	high-pressure mercury vapour lamp (200 W)	4.9	79.2	32

Comment 10: What specific reactions do photogenerated electrons and holes undergo at the surface of the photocatalyst during the photocatalytic process? While theoretical calculations can provide valuable insights, they are insufficient as standalone evidence. It is essential to support mechanistic interpretations with robust experimental data to reliably elucidate the reaction pathways of charge carriers.

Response: We sincerely thank you for raising this critical point regarding the specific reactions of photogenerated charge carriers. Based on our band-structure analysis, in situ XPS, EPR, DRIFTS, scavenger tests, and DFT calculations, we can now describe these surface processes explicitly as follows:

Light absorption and charge generation: $ZnO + h\nu \rightarrow e^-_{CB} + h^+_{VB}$

(1) Reactions of photogenerated electrons

Electrons are preferentially transferred from ZnO to isolated Ru sites and then used for selective O_2 reduction to the •OOH intermediate.

a) Electron trapping on Ru single atoms:

- $e_{CB}^{-} + Ru^{n+} \rightarrow Ru^{(n-1)+}$,

This is directly evidenced by the negative shift in Ru 3d binding energy under illumination in in situ XPS, confirming Ru sites as electron acceptors (**Fig. 6a**).

b) Stepwise O_2 reduction at Ru sites to •OOH (dominant pathway)

- $Ru^{(n-1)+} + O_2(ads) \rightarrow Ru^{n+} + O_2^{-}(ads)$
- $O_2^{-}(ads) + H^{+} \rightarrow {\cdot}OOH$

DFT calculations show that on Ru_1/ZB-ZnO(222), O_2 binds in an end-on configuration with favorable adsorption energy and a thermodynamically accessible pathway to •OOH, while further O–O bond cleavage to •OH is kinetically hindered (higher ΔG for •OOH → •OH), thereby stabilizing the •OOH intermediate (**Fig. 6i**). This is consistent with: Strong •OOH signals in EPR on Ru-containing samples (**Fig. 6d–e**), and the characteristic •OOH band at ~870 cm^{-1} in in situ DRIFTS on Ru_1/ZB-ZnO(222) (**Fig. 6g**). Crucially, the severe suppression of CH_3OOH formation upon adding $AgNO_3$ directly confirms that electrons are indispensable for the reduction step that generates the key •OOH intermediate (**Supplementary Figure S46**).

c) Coupling of •OOH with $•CH_3$ to form CH_3OOH

- ${\cdot}CH_3 + {\cdot}OOH \rightarrow CH_3OOH$

The oxygen source of CH_3OOH is confirmed by ^{18}O-labeling (**Fig. 6f**): $^{18}O_2 + H_2{}^{16}O$ leads to $CH_3{}^{18}OH$ after $NaBH_4$ reduction of CH_3OOH, whereas $^{16}O_2 + H_2{}^{18}O$ does not, proving that O_2, reduced by photoelectrons, provides the oxygen in CH_3OOH.

(2) Reactions of photogenerated holes

a) Hole localization and lattice oxygen involvement

In situ XPS shows a positive shift of the O 1s peak under light, indicating that holes accumulate on lattice/adsorbed oxygen species (O^{2-}/O^{-}), forming highly oxidizing O-centered holes:

- $h_{VB}^{+} + O_{lattice/surf}^{2-} \rightarrow O_{lattice/surface}^{-}$

b) H_2O oxidation to •OH (secondary but important pathway)

Holes can oxidize adsorbed water/hydroxyls to •OH:

- $O_{surf}^{-} + H_2O \rightarrow {\cdot}OH + H^{+} + O_{surf}^{2-}$

The role of •OH is confirmed by scavenger tests: isopropanol (•OH scavenger) reduces the liquid product yield to ≈45% of the original (**Supplementary Figure S46**), showing that •OH contributes significantly, though not exclusively, to CH_4 activation.

c) Direct CH_4 activation by holes to surface $•CH_3$ (dominant for C–H activation)

Our DRIFTS data under CH_4 (**Fig. 6g–h** and **Supplementary Fig. S47**) show clear signatures of $*CH_3$ and $*OCH_3$ intermediates, indicating direct oxidative C–H bond activation at ZnO surface sites:

- $CH_4(ads) + h_{VB}^{+} \rightarrow {\cdot}CH_3 + H^{+}$

and subsequent stabilization/partial oxidation:

- $CH_3 + O_{surf} \rightarrow {\cdot}OCH_3(ads)$

The nearly complete suppression of reaction by $Na_2C_2O_4$ (hole scavenger) in quenching experiments (**Supplementary Fig. S46**) demonstrates that holes are the primary oxidants responsible for methane activation, with •OH playing an assisting role.

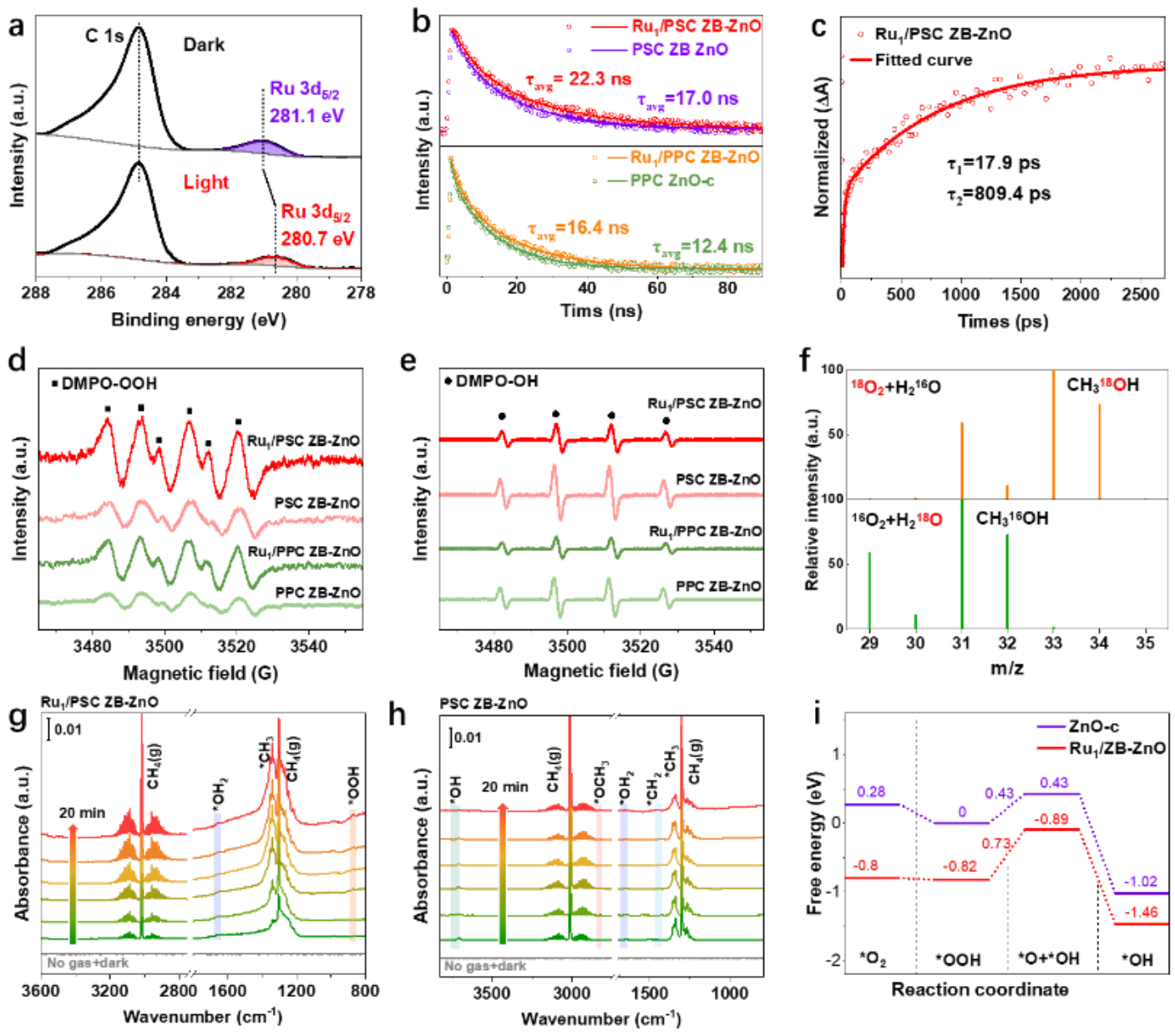

<u>**Fig. 6.** Mechanism of the selective CH_4 photo-oxidation over the Ru_1/PSC ZB-ZnO catalyst. **a,** In situ Ru $3d_{5/2}$ XPS spectra of Ru/PSC ZB-ZnO in the dark and under light irradiation. **b,** Time-resolved PL decay of Ru_1/PSC ZB-ZnO and PSC ZB-ZnO. **c,** The normalized TAS decay kinetics of Ru_1/PSC ZB-ZnO probed at 412 nm excited by pump 360 nm. **d-e,** EPR spectra of Ru_1/PSC ZB-ZnO, PSC ZB-ZnO, Ru_1/PPC ZB-ZnO and PPC ZB-ZnO in air-saturated water under light irradiation for 5 min. **f,** GC-MS spectra of CH_3OH reduced CH_3OOH over Ru_1/PSC ZB-ZnO with $^{18}O_2$ + $H_2{}^{16}O$ or $^{16}O_2$ + $H_2{}^{18}O$ in photocatalytic CH_4 oxidation. **g-h,** In situ DRIFTS spectra for CH_4 photooxidation over Ru_1/PSC ZB-ZnO and PSC ZB-ZnO during light irradiation. **i,** Calculated reaction energy of O_2 adsorption and activation over Ru_1/ZB-ZnO and ZB-ZnO catalysts.</u>

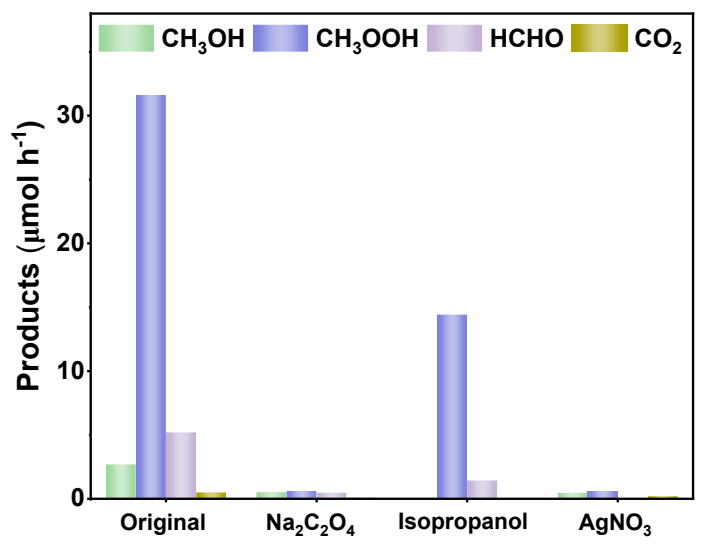

<u>**Figure S46.** Active species trapping experiments for photocatalytic CH_4 oxidation. Influence of the scavengers of the photocatalytic CH_4 oxidation over PSC Ru_1/ZB-ZnO. $Na_2C_2O_4$ as h^+ capture agent, isopropanol as ·OH capture agent, $AgNO_3$ as e^- capture agent. Batch reactor, 0.1 MPa air (25 vol.% O_2 and N_2), 1.9 MPa CH_4, 50 mL H_2O, 1 h reaction time.</u>

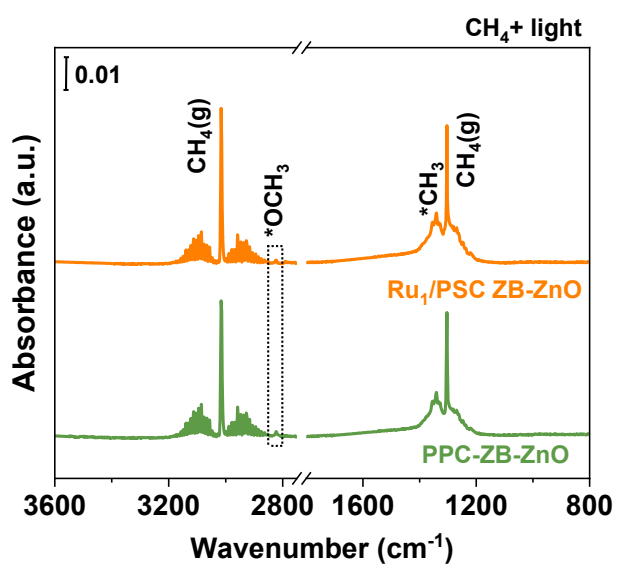

<u>**Figure S47.** In situ diffuse reflectance infrared Fourier transform spectroscopy (DRIFTS) for CH_4 photooxidation over Ru_1/PSC ZB-ZnO and PSC ZB-ZnO under light and CH_4.</u>

Comment 11: What roles do Ru and ZnO play individually in the photocatalytic process? Is there

evidence of a synergistic interaction between Ru and ZnO under illumination? Furthermore, what are the specific adsorption sites on the catalyst surface for CH_4, H_2O, and O_2, respectively? To gain mechanistic insight into the reaction pathways and surface processes, comprehensive in-situ spectroscopic investigations—such as in-situ DRIFTS, XPS, or Raman spectroscopy—should be conducted to dynamically monitor the evolution of surface species and intermediate states during the catalytic cycle.

Response: We sincerely thank you for raising these insightful questions regarding the roles of Ru and ZnO, their potential synergy, and the specific surface adsorption sites—all of which are central to understanding the photocatalytic mechanism. We have performed a series of spectroscopic investigations, including in situ DRIFTS, XPS, EPR, and complementary TPD and DFT calculations. These experiments collectively provide clear evidence for the distinct functions of each component, their synergistic interaction under illumination, and the dynamic evolution of key surface species and intermediates during the reaction.

1. Individual roles of Ru and ZnO in the photocatalytic process

(1) Role of ZnO (PSC ZB-ZnO framework): light absorber, bulk charge-transport medium, and CH_4 adsorption/oxidation site

- Light absorption and charge generation: UV–vis DRS, UPS, VB-XPS, and Mott–Schottky analyses (**Supplementary Figs. 35–39**) show that PSC ZB-ZnO determines the fundamental band structure. Its band positions allow both H_2O oxidation to •OH and O_2 reduction to •OOH. Ru loading only slightly shifts the band edges.
- Efficient charge transport: PL and time-resolved PL (**Fig. 6b**, **Supplementary Fig. 41**) indicate that PSC ZB-ZnO exhibits lower recombination and longer lifetimes than the porous polycrystalline control, due to the absence of grain boundaries. EIS (**Supplementary Fig. 42**) shows lower charge-transfer resistance in the PSC framework. Thus, PSC ZnO provides a highly conductive, low-defect pathway for photogenerated carriers.
- CH_4 adsorption and oxidation sites: CH_4-TPD (**Supplementary Fig. S45**) shows a strong desorption peak at ~470 °C for both PSC ZB-ZnO and Ru_1/PSC ZB-ZnO, assigned to CH_4 adsorbed on ZnO lattice sites. Ru loading introduces only a weak additional peak at ~288 °C. This indicates that CH_4 adsorbs predominantly on Zn-O lattice sites of ZnO, not on Ru. In-situ DRIFTS (**Supplementary Fig. S47**) further reveals surface $*CH_3$ and $*OCH_3$ species under CH_4 and light, confirming that holes localized on lattice oxygen (O^-) in ZnO are responsible for C–H bond activation and formation of $•CH_3$ and subsequent intermediates.

(2) Role of Ru single atom: selectron trapping, O_2 activation

- Electron acceptor / reduction cocatalyst: In-situ XPS under illumination (**Fig. 6a**) shows a negative shift of the Ru 3d5/2 binding energy (281.1 → 280.7 eV) while the O 1s peak shifts to higher binding energy. This demonstrates that photoexcited electrons migrate to Ru, whereas holes accumulate on lattice oxygen in ZnO. Therefore, Ru acts as an electron sink/O_2 reduction site, leaving oxidizing holes on ZnO.
- Enhancing charge separation and lifetime: Steady-state PL and time-resolved PL (**Fig. 6b; Supplementary Fig. 41**) show that Ru loading significantly quenches emission and extends carrier lifetimes (τ_{avg}: PSC ZB-ZnO 14.33 ns → Ru_1/PSC ZB-ZnO 17.40 ns), evidencing improved charge

separation. Fs-TAS (**Fig. 6c**, **Supplementary Figs. 43–44** and **Table S7**) shows that Ru accelerates electron trapping and prolongs radiative recombination times, i.e., more electrons are funneled to Ru sites and remain available for surface reduction reactions.

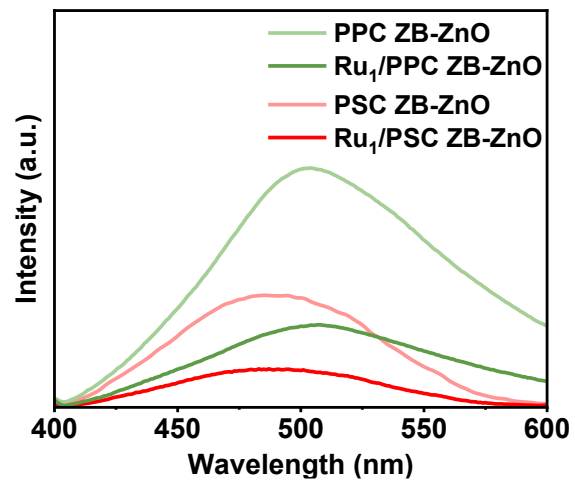

Figure S41. Steady-state PL characterizations of ZnO samples, with and without Ru single-atom modification. Ru modification enhances the charge separation efficiency of ZnO-based photocatalysts, while the single-crystalline structure synergistically suppresses the recombination of photo-generated carriers.

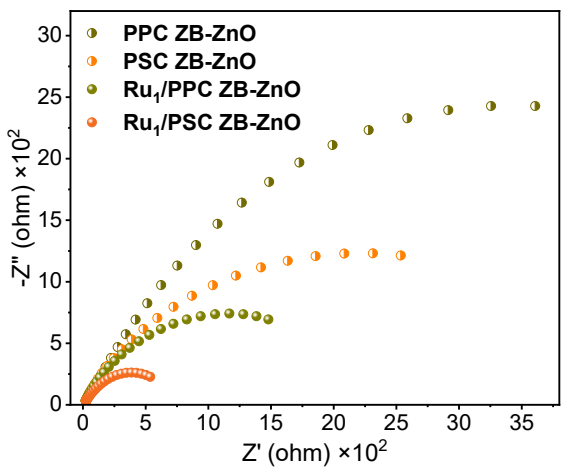

Figure S42. Electrochemical impedance spectroscopy (EIS) of single-crystal and polycrystalline ZnO with and without Ru single-atom modification. EIS were performed in a 0.1 M Na_2SO_4.aqueous. The analysis demonstrates that that single-crystalline ZnO (PSC ZB-ZnO) possesses a substantially lower charge-transfer resistance than its polycrystalline counterparts (PPS ZnO-c). Moreover, this advantage is further accentuated upon Ru modification. Specifically, the Ru-modified systems display a notably smaller semicircle radius in the Nyquist plots, evidencing a significant further reduction in charge-transfer resistance. This underscores a synergistic interplay, where the single-crystal morphology provides an efficient conductive pathway, and the atomic-level Ru incorporation actively enhances the interfacial charge-transfer kinetics.

- Selective O_2 activation to •OOH:
  O_2-TPD (Supplementary Fig. S45) clearly shows enhanced O_2 adsorption on Ru_1/PSC ZB-ZnO compared to bare PSC ZB-ZnO, indicating that Ru atoms are preferential O_2 adsorption sites. In-situ EPR with DMPO (Fig. 6d–e) reveals a marked shift from •OH-dominated radicals on pristine ZnO to •OOH-dominated signals on Ru-loaded samples. This evidences that Ru–O_4 sites preferentially drive $O_2 \rightarrow$ •OOH rather than deep reduction to •OH. DFT calculations on Ru_1/ZB-ZnO(222) vs. ZB-ZnO(222) (**Fig. 6i**, **Supplementary Figs. 48–50**) support this: Ru_1/ZB-ZnO stabilizes an end-on O_2 configuration and lowers the barrier for •OOH formation while increasing the barrier for O–O bond cleavage to •OH. Thus Ru kinetically and thermodynamically biases the oxygen reduction pathway toward •OOH.

2. Evidence for synergistic Ru–ZnO interaction under illumination

- Performance comparison beyond simple additivity: Ru_1/PSC ZB-ZnO shows a 4.6-fold increase in CH_3OOH production over PSC ZB-ZnO, and none of the evaluated RuO_2 or other Ru_1O_x/MO_x catalysts demonstrated a significant yield of any oxidative products. (**Supplementary Fig. 33**). The performance of Ru_1/PSC ZnO is significantly superior to that of either PSC ZnO alone or RuO_2 alone, indicating a synergistic effect between Ru and ZnO.

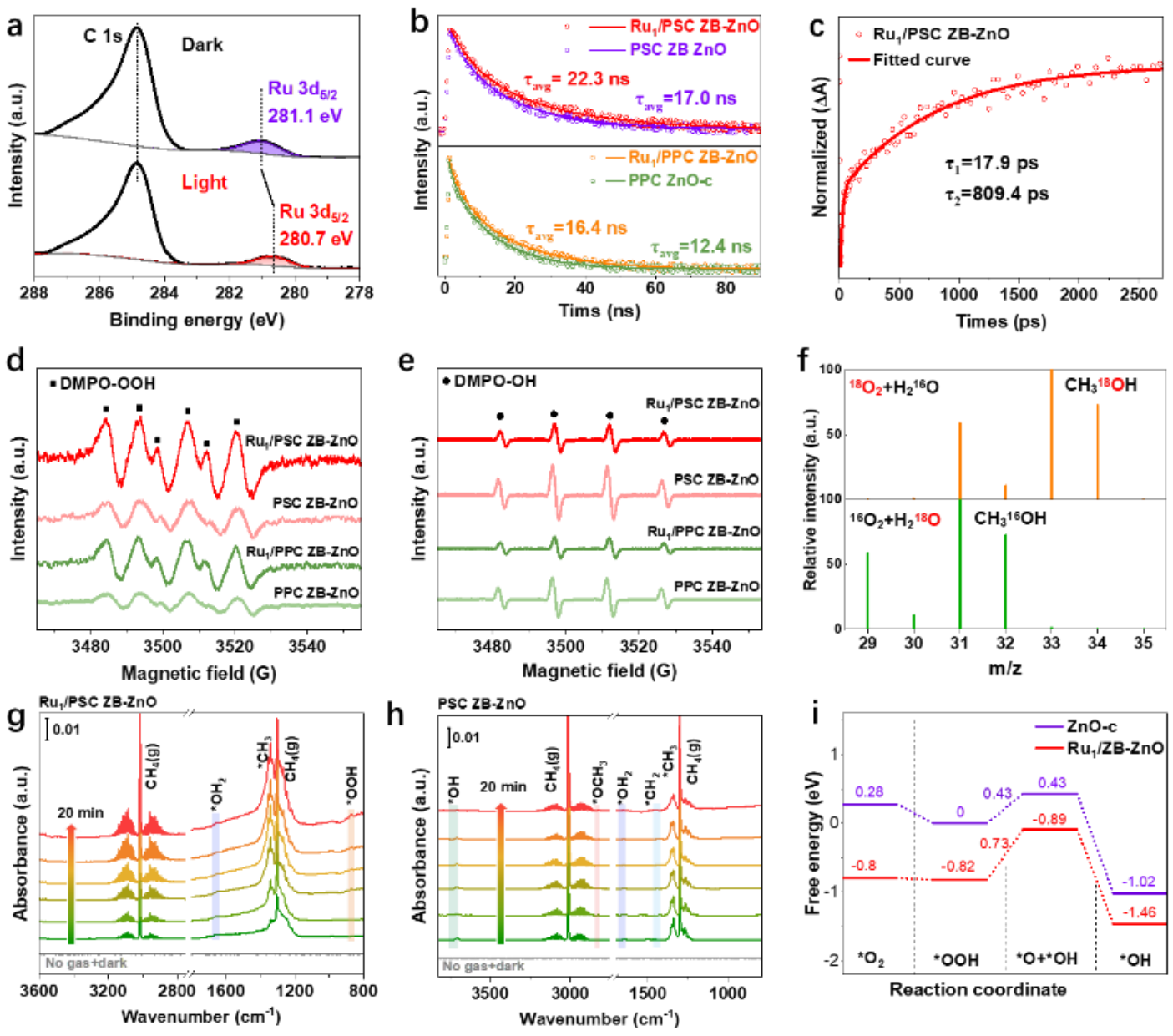

Fig. 6. Mechanism of the selective CH_4 photo-oxidation over the Ru_1/PSC ZB-ZnO catalyst. **a,** In situ Ru $3d_{5/2}$ XPS spectra of Ru/PSC ZB-ZnO in the dark and under light irradiation. **b,** Time-resolved PL decay of Ru_1/PSC ZB-ZnO and PSC ZB-ZnO. **c,** The normalized TAS decay kinetics of Ru_1/PSC ZB-ZnO probed at 412 nm excited by pump 360 nm. **d-e,** EPR spectra of Ru_1/PSC ZB-ZnO, PSC ZB-ZnO, Ru_1/PPC ZB-ZnO and PPC ZB-ZnO in air-saturated water under light irradiation for 5 min. **f,** GC-MS spectra of CH_3OH reduced CH_3OOH over Ru_1/PSC ZB-ZnO with $^{18}O_2$ + $H_2{}^{16}O$ or $^{16}O_2$ + $H_2{}^{18}O$ in photocatalytic CH_4 oxidation. **g-h,** In situ DRIFTS spectra for CH_4 photooxidation over Ru_1/PSC ZB-ZnO and PSC ZB-ZnO during light irradiation. **i,** Calculated reaction energy of O_2 adsorption and activation over Ru_1/ZB-ZnO and ZB-ZnO catalysts.

- Cooperative charge-separation pathway: In-situ XPS (**Fig. 6a** and O 1s shift in Supplementary **Fig. 40**) shows that under illumination, electrons flow specifically to Ru while holes accumulate on ZnO lattice oxygen. This spatial separation is only possible because Ru is electronically coupled to the conductive single-crystal ZnO network. Thus, Ru and ZnO jointly create a vectorial charge-separation pathway:
  ZnO: light absorption → e^-/h^+ generation → h^+ on O_lattice (CH_4 oxidation)
  Ru: e^- trapping (O_2 → •OOH).
  The two components therefore cooperate rather than acting independently.

- Coupled radical pathway control:
  In-situ EPR (**Fig. 6d–e**) and in-situ DRIFTS (**Fig. 6g–h**) show that, on Ru_1/PSC ZB-ZnO, the surface chemistry is collectively reshaped:
  – DRIFTS on Ru_1/PSC ZB-ZnO shows simultaneous buildup of $*CH_3$ and •OOH bands and formation of CH_3OOH (**Fig. 6g**),
  – While on bare PSC ZB-ZnO, strong •OH bands and deeper-oxidation intermediates ($*CH_2$, $*OCH_3$) dominate (**Fig. 6h**).
  These observations indicate that CH_4 activation (on ZnO) and O_2 activation (on Ru) are mechanistically coupled: Ru changes the fate of electrons and O_2, which in turn changes the fate of the $*CH_3$ radicals that are generated on ZnO.

3. Specific adsorption sites for CH_4, H_2O, and O_2

(1) CH_4 adsorption: CH_4-TPD (**Supplementary Fig. S45**) shows a dominant desorption peak at ~470 °C for both PSC ZB-ZnO and Ru_1/PSC ZB-ZnO, assigned to CH_4 bound on ZnO lattice sites. Ru introduces only a minor additional low-temperature peak (~288 °C), confirming its limited role as a CH_4 adsorption site. In-situ DRIFTS (**Fig. 6g–h, Supplementary Fig. S47**) detects $*CH_3$ and $*OCH_3$ species on both catalysts under illumination. These intermediates are associated with oxidation on Zn–O lattice sites. CH_4 adsorbs predominantly on ZnO surface sites ($Zn^{\delta+}$–$O^{\delta-}$ pairs / lattice O), where photogenerated holes on $O_{lattice}$ abstract H atoms to form $*CH_3$.

(2) O_2 preferentially adsorbs on Ru single sites on the ZnO surface, where it is reduced by Ru-trapped electrons to •OOH.

O_2-TPD shows substantially increased O_2 adsorption capacity upon Ru introduction (Supplementary Fig. S45), indicating that Ru centers are the preferred O_2 binding sites. In-situ EPR reveals that Ru loading switches the predominant radical from •OH (ZnO) to •OOH (Ru_1/ZnO), consistent with O_2 reduction occurring at Ru sites. DFT (Fig. 6i, Supplementary Figs. 48–50) explicitly models O_2 adsorption on Ru_1/ZB-ZnO(222) and bare ZB-ZnO(222), showing that Ru stabilizes a strongly chemisorbed end-on O_2 configuration and facilitates •OOH formation.

(3) H_2O primarily adsorbs on ZnO surface sites (terminal Zn–OH or O–H species), and these sites are responsible for •OH generation in the absence of Ru.

H_2O-TPD (**Supplementary Fig. S45**) shows almost identical desorption profiles for PSC ZB-ZnO and Ru_1/PSC ZB-ZnO, demonstrating that Ru does not significantly alter water adsorption. The H_2O adsorption is therefore governed by ZnO's surface hydroxyls and coordinatively unsaturated Zn/O sites. In-situ DRIFTS on PSC ZB-ZnO (Fig. 6h) shows an OH stretching band at 3703 cm^{-1} assigned to surface •OH species formed from H_2O under illumination, again linked to ZnO lattice oxygen.

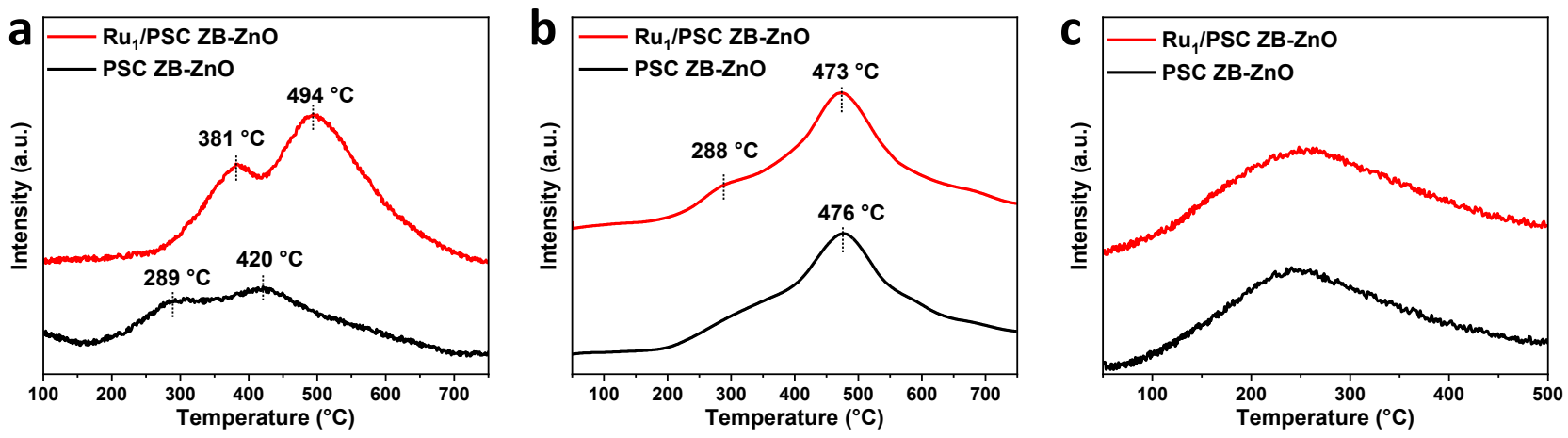

Figure S45. Temperature-programmed desorption (TPD) analyses. **a-c,** O_2-TPD, CH_4-TPD, H_2O-TPD spectra of Ru_1/PSC ZB-ZnO and PSC ZB-ZnO.

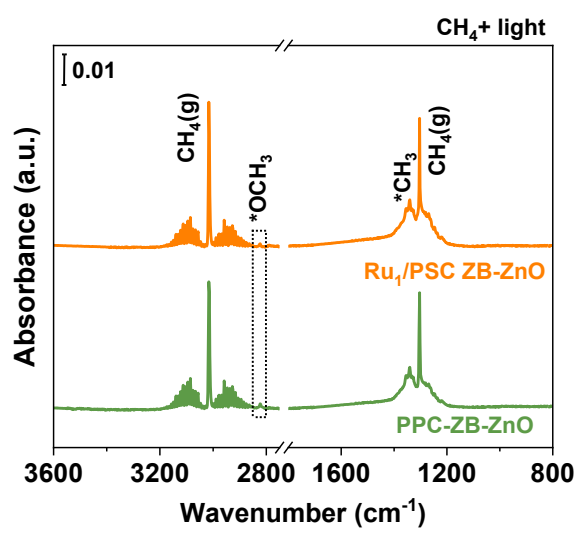

Figure S47. In situ diffuse reflectance infrared Fourier transform spectroscopy (DRIFTS) for CH_4 photooxidation over Ru_1/PSC ZB-ZnO and PSC ZB-ZnO under light and CH_4.

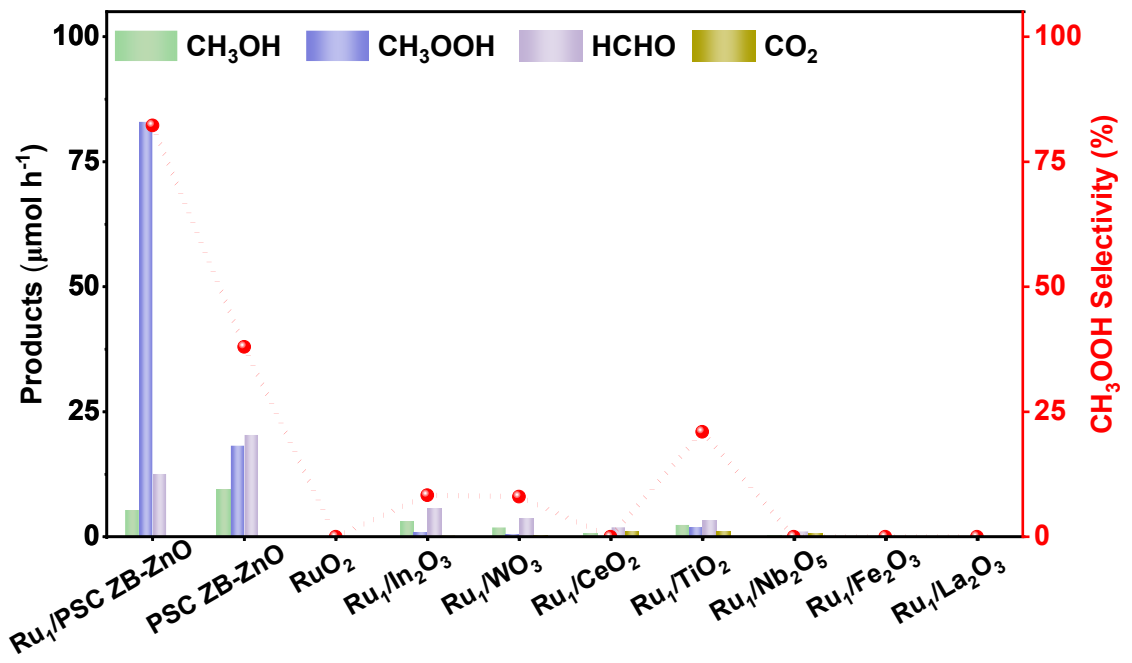

Figure S33. Performance evaluation of Ru_1/PSC ZnO and other Ru-based catalysts in the photocatalytic methane oxidation reaction.

Comment 12: Please provide the original gas chromatography (GC) data and gas chromatography-mass spectrometry (GC-MS) data for the photocatalytic gaseous products to ensure that all potential by-products are fully accounted for and not overlooked.

Response: We thank you for carefully reviewing our manuscript. For the gaseous products, as shown in **Figure S27**, no gaseous products peaks were observed after 2 hours of reaction in the gas chromatography (GC). After 10 hours, only a trace CO_2 peak was observed, with no other gaseous products found. GC-MS total ion chromatograms (TICs) confirmed no co-eluting peaks or unresolved species.

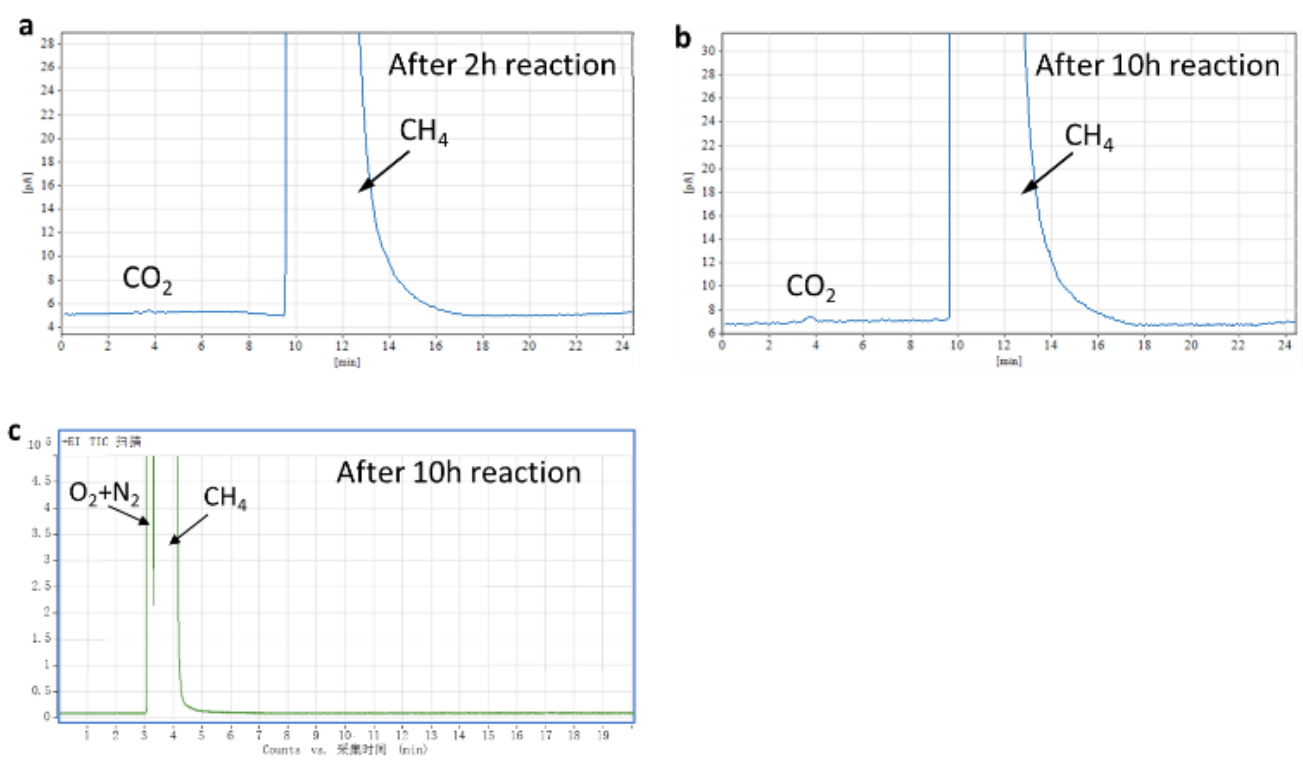

Figure S28. Original GC-FID spectras for outlet gas after photocatalytic methane oxidation over Ru_1/PSC ZB-ZnO for (a) 2 h and (b) 10 h reaction. (c) GC-MS chromatogram GC-MS total ion chromatograms (TICs) confirmed no co-eluting peaks or unresolved species after 10 hours reaction.

Comment 13: In Figure 6 and Table S3, the normalized TAS decay kinetics fitting lifetimes of ZB-ZnO and Ru_1/ZB-ZnO are opposite.

Response: We sincerely appreciate your keen observation regarding the issue of the incorrectly labeled normalized TAS decay kinetics fitting lifetimes for PSC ZB-ZnO and Ru_1/PSC ZB-ZnO in **Figures 6b-c**. This error occurred during the figure assembly process, where the sample identifiers for the transient absorption spectroscopy (TAS) decay kinetics were accidentally labeled in reverse. **Figures 6b-c** have been relabeled as ZB-ZnO and Ru_1/ZnO, maintaining consistency with the results in **Table 3**.

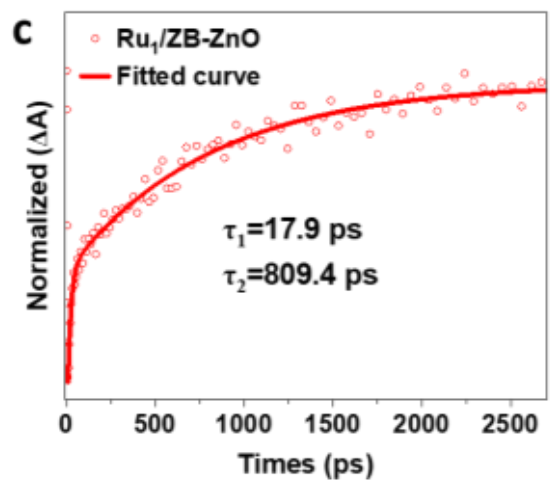

Fig. 6. c, The normalized TAS decay kinetics of Ru$_1$/PSC ZB-ZnO probed at 412 nm excited by pump 360 nm.

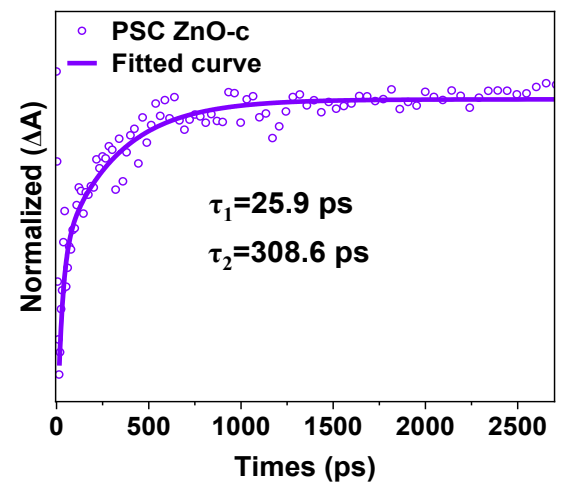

Figure S44. The normalized TAS decay kinetics of PSC ZB-ZnO probed at 412 nm excited by pump 360 nm.

Table 7. Fitted lifetime from normalized TAS decay kinetics of ZB-ZnO and Ru$_1$/ZB-ZnO.

Sample	A_1	τ_1 (ps)	A_2	τ_2 (ps)	τ_{avg} (ps)
PSC ZB-ZnO	1.2	25.9	0.56	308.6	265.5
Ru$_1$/PSC ZB-ZnO	0.48	17.9	0.48	809.4	792.3

Response to reviewers

Response to Reviewer #1

General comments: The authors have addressed most of the concerns. The light intensity is 1000 mW/cm^2, which is relatively high. Evaluating the photocatalytic performance in terms of apparent quantum efficiency is more reasonable. How about the apparent quantum efficiency of Ru_1/PSC ZB-ZnO?

Response: We sincerely appreciate your comment, which strengthens our performance evaluation. We have conducted additional experiments to evaluate the apparent quantum efficiency (AQE) of Ru_1/PSC ZB-ZnO.

Table S3. The amount of formed liquid oxygenates, the light intensity, and calculated AQE at different wavelengths.

Wavelength (nm)	Light Intensity (W/cm^2)	CH_3OOH (μmol)	CH_3OH (μmol)	HCHO (μmol)	AQE (%)
365	0.018	55.6	4.6	5.1	15.3
380	0.021	58.8	3.8	5.6	13.0
420	0.026	2.1	0.2	0.1	0.3

The AQE of Ru_1/ZnO photocatalyst was measured at different wavenumbers using a Xe lamp equipped with band-pass filter as the light source. AQE was determined using the following equation:

$$\mathrm{AQE}=\frac{N_\mathrm{e}}{N(\mathrm{photons})}\times 100\%$$

Where N_e and N(photons) represent the number of reacted electrons and the number of incident photons, respectively. The number of reacted electrons $N_e = N(CH_3OOH) + 3 \times N(CH_3OH) + 5 \times N(HCHO)$. Where $N(CH_3OOH)$, $N(CH_3OH)$, N(HCHO) denote the molar quantities of synthesized methyl hydroperoxide, methanol, and formaldehyde, respectively. The number of incident photons is calculated as $N(\mathrm{photons}) = I \cdot A \cdot t / E_\lambda$, where I, A, t and E_λ denote wavelength-specific irradiance (W cm^{-2}), illuminated catalyst area (cm^2). light incident time (s) and photon energy (J), respectively. $E_\lambda = hc/\lambda$ (λ = 365, 380 or 420 nm).

Response to Reviewer #2

General comments: The revision dramatically improves the quality of this work and clarity of the presentation. The reviewer does not have other comment and would suggest to accept it.

Response: We sincerely appreciate your time and positive comment.

Response to Reviewer #3

General comments: The authors have responded well to the majority of comments from reviewers. And the manuscript is nearly ready for acceptance. Please address one remaining point:

Generally, photocatalyst surface temperature will rises significantly under strong light irradiation (1000 mW/cm^2). Thus, the observed proportionality between photocatalytic efficiency and irradiation intensity

alone does not confirm a purely photocatalytic mechanism (without photothermal effect). Please clarify how surface temperature was measured. A thermocouple measures ambient temperature near the catalyst, not the actual irradiated surface temperature. Use an infrared thermometer to measure the transient surface temperature immediately after prolonged illumination, values often exceed 32 °C. This clarification will strengthen the mechanistic interpretation without affecting the manuscript's quality or impact.

Response: We sincerely appreciate your insightful comment. We fully acknowledge that significant photothermal heating can occur under intense light, which may interfere with the interpretation of photocatalytic mechanisms. In our experimental design, to minimize such thermal effects, we employed a photoreactor equipped with a circulating water system, which continuously remove heat during irradiation.

Accurately measuring the surface temperature of catalysts during photothermal catalysis remains a methodological challenge. As demonstrated in prior temperature detection in gas−solid photocatalytic systems *via* thermocouples, the temperature at the edge of the quartz vessel loaded with catalyst was designated as the catalyst surface's isothermal temperature parameter (**Figure R,** *Nature Communications, 2025, 16, 2550*). Thus, in our preliminary experiments, we used thermocouple to measure the temperature at the interface between the catalyst and the reactor wall (Position 1, **Figure S24**). The temperature at position 1 stabilized at 32°C after more than ten minutes, indicating that the circulating water effectively reduces the temperature around the reactor wall to a low level.

Nevertheless, as you rightly pointed out, in practical gas−solid flow photoreactors, thermal gradients can arise due to combined photoexcitation, gas flow, and reaction heat effects. The temperature at position 1 may not accurately represent the average temperature on the catalyst surface under intense light irradiation. Therefore, we conducted additional measurements near the upper surface at the center of the catalyst bed (Position 2). With the cooling system in operation under our photooxidation conditions, the temperature at Position 2 stabilized at 69 °C. In contrast, without active cooling, the temperature rapidly exceeded 170 °C. This marked temperature reduction with the cooling circulation is attributed not only to the efficient heat dissipation of the system itself, but may also to the favorable thermal conductivity of the catalyst.

To further rule out contributions from thermal pathways in catalytic activity, we conducted a dark control experiment at an elevated temperature (**Figure S34**). Even when the catalyst was heated to 120 °C in the absence of light, no measurable product formation was detected. Thus, the catalytic activity under illumination—with the catalyst surface temperature at 69 °C—originate from a genuine photo-driven catalytic process, rather than from a photothermal effects.

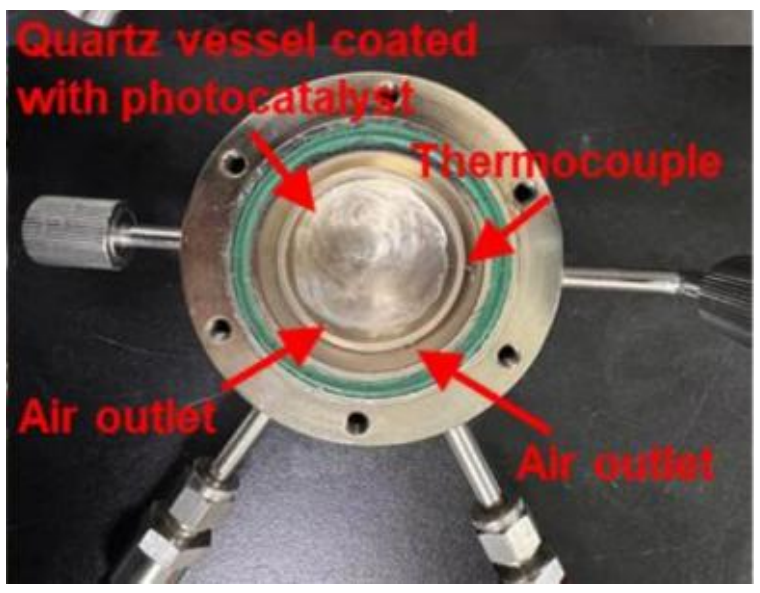

Figure R. Physical photo of the reaction device for photothermal catalytic selective oxidation of CH_4 (*Nature Communications, 2025, 16, 2550*).

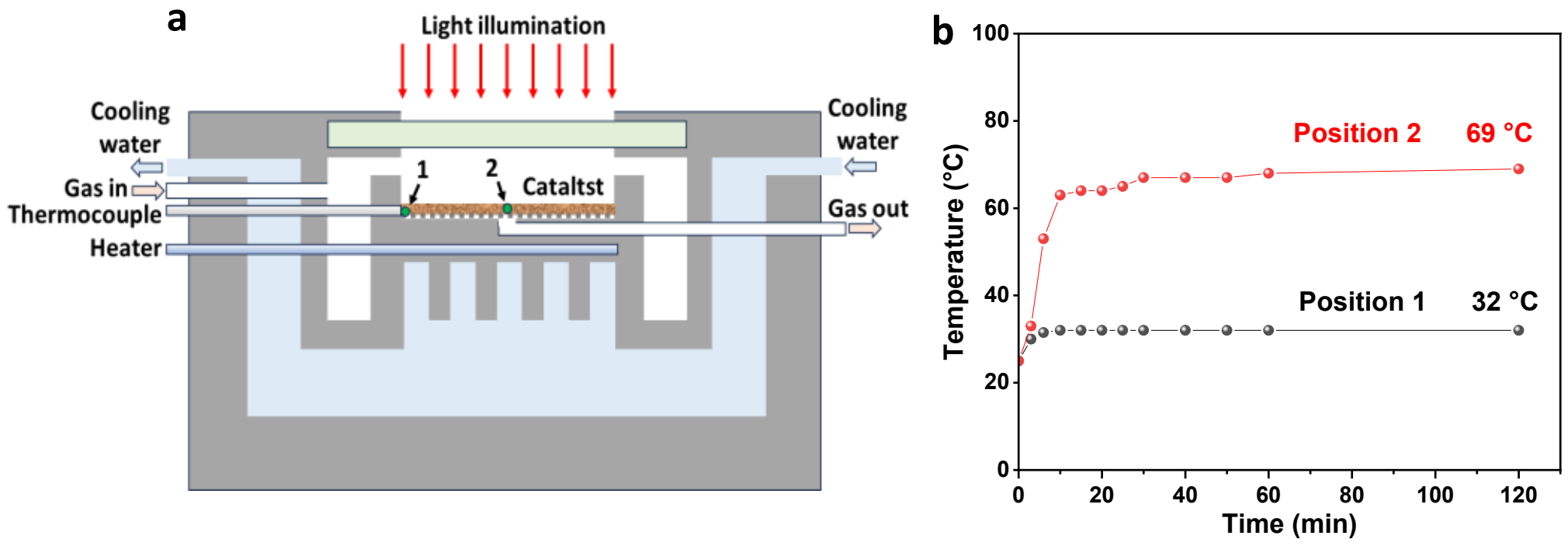

Figure S24. Temperature detection in gas−solid photoreactor equipped with a circulating water system via thermocouple. **a,** Schematic diagram of test positions. **b,** The temperature of different positions detected by a thermometer under light at different reaction time during photothermal catalytic oxidation. The temperature at the interface between the catalyst and the reactor wall (position 1) stabilized at 32°C after more than ten minutes, indicating that the circulating water effectively reduces the temperature around the reactor wall to a low level. The temperature near the upper surface at the center of the catalyst bed (Position 2) stabilized at 69 °C with the cooling system in operation under our photooxidation conditions.

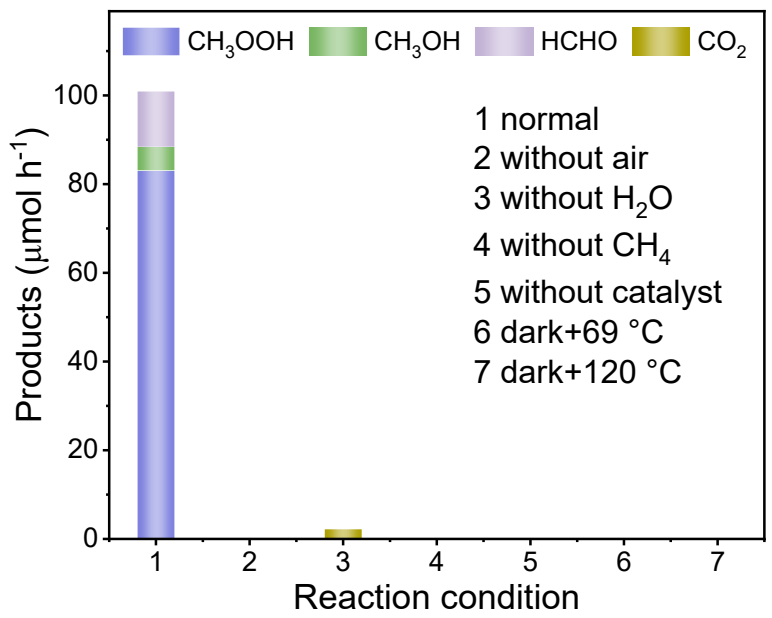

Figure 34. Control experiments screening the necessity of key reaction components. Production rates obtained over Ru_1/PSC ZB-ZnO under different conditions. Reaction conditions: 45 mL min^{-1} CH_4 and 5 mL min^{-1} air (25 vol.% O_2/N_2), RH = 20%, 50 °C, 300 W Xe lamp with AM 1.5G filter, 1000 mW cm^{-2}. The results demonstrate that significant product formation occurs exclusively under light irradiation with all reaction components present. The negligible activity observed in the dark (even with system temperature rise to 120 ℃), under visible light, in the absence of methane, or without catalyst, systematically rules out thermal effects. O_2 and H_2O is essential for initiating methane oxidation.
